# Directed Evolution of Threonine Aldolases for the Photobiocatalytic Synthesis of β-Branched-α-Tetrasubstituted and Other Non-Canonical Amino Acids

**DOI:** 10.1021/jacs.6c03303

**Published:** 2026-05-21

**Authors:** Tian-Ci Wang, Xiangyu Zhang, Zheng Zhang, Guodong Rao, Zhiyu Bo, Pei-Pei Xie, Jiedong Li, Qiang Yang, Xiaoyong Li, R. David Britt, Jiang Wang, Peng Liu, Yang Yang

**Affiliations:** 1 Department of Chemistry and Biochemistry, University of California Santa Barbara, Santa Barbara, California 93106; 2 Department of Chemistry, University of Pittsburgh, Pittsburgh, Pennsylvania 15260; 3 Department of Chemistry, University of California Davis, Davis, California 95616; 4 Synthetic Molecule Design and Development, Lilly Research Laboratories, Eli Lilly and Company, Indianapolis, Indiana 46285; 5 Department of Bioengineering, University of California Santa Barbara, Santa Barbara, California 93106; 6 Biomolecular Science and Engineering (BMSE) Program, University of California Santa Barbara, Santa Barbara, California 93106; 7 Howard Hughes Medical Institute, University of California Santa Barbara, Santa Barbara, California 93106

## Abstract

The convergent, stereoselective, and protecting-group-free synthesis of non-canonical amino acids, particularly those bearing a tetrasubstituted α-stereogenic center, remains a challenging task. By leveraging pyridoxal radical biocatalysis, we developed photobiocatalytic oxidative coupling methods for the assembly of α-tetrasubstituted amino acids, including β-branched variants, in a diastereo- and enantioselective fashion. Through repurposing and directed evolution of *Thermotoga maritima* threonine aldolases, we transformed a traditionally two-electron pyridoxal phosphate (PLP) dependent enzyme into a highly active and stereoselective biocatalyst for single-electron C–C bond formation, enabling enantioconvergent conversion of a broad range of racemic organoboron substrates. Mechanistic studies using radical clock probes and electron paramagnetic resonance (EPR) spectroscopy uncovered an unexpected role of free PLP in oxidative radical generation under photochemical conditions, highlighting the unique photochemical properties of this natural cofactor. Molecular dynamics (MD) simulations further elucidated the origin of enantio- and diastereoselectivity in the key radical addition step between the enzymatic quinonoid and the carbon-centered radical. Collectively, these results underscore the power of pyridoxal radical biocatalysis to access a broad spectrum of valuable non-canonical amino acid products via intermolecular, enzyme-controlled asymmetric radical chemistry, a transformation that remains elusive to both state-of-the-art small-molecule catalysis and native enzymology.

## Graphical Abstract

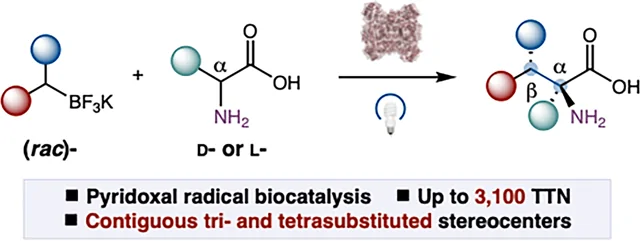

## Introduction

Non-canonical amino acids (ncAAs) constitute fundamental building blocks in biologically active natural products and clinically indispensable peptide therapeutics.^1^ With the advent of powerful genetic code expansion tools, a range of non-canonical amino acids can now be conveniently and precisely introduced into proteins with biological, therapeutic, and biocatalytic significance.^2–3^ Driven by the recent clinical success of macrocyclic peptide and glucagon-like peptide-based therapeutics,^4^ new technologies to allow for the efficient, stereoselective, and sustainable synthesis of underexploited ncAAs have been extensively sought after by the pharmaceutical industry. In this context, biocatalysis represents a promising alternative to conventional chemical synthesis,^5^ offering excellent stereocontrol and obviating the need for amino and carboxylate protecting groups during the synthesis of complex amino acids.^6^

Compared to traditional α-substituted α-amino acids, non-canonical α,α-disubstituted α-amino acids bearing a tetrasubstituted α-stereocenter offer enhanced chemical and proteolytic stability, along with improved potency and conformational rigidity.^7^ Among them, despite their added synthetic complexity, β-branched-α-tetrasubstituted ncAAs featuring contiguous tetra- and trisubstituted stereocenters at the α and β positions represent a class of highly promising but underexploited ncAA building blocks.^8^ Synthetic methods allowing the assembly of these challenging chemical entities rely on the use of tedious protection-deprotection sequences, with the majority of catalytic asymmetric methods hinging on transition-metal-catalyzed asymmetric allylic substitution reactions with protected amino acid equivalents.^7a, 8^ Despite the excellent potential, naturally occurring amino acid producing enzymes, including 4-methylideneimidazole-5-one (MIO)-dependent C–N lyases,^9^ pyridoxal phosphate (PLP)-dependent transaminases^10–11^ and the vast majority of PLP-dependent C–C bond forming enzymes,^12–17^ are generally incapable of producing non-canonical amino acids with a tetrasubstituted α-carbon. Important studies by Griengl and Fesko demonstrated that a subset of PLP-dependent threonine aldolases could allow the synthesis of α-tetrasubstituted amino acids via the native aldolase activity.^18^ However, the reversible nature and the undesirable thermodynamics of this two-electron PLP aldolase process, which favor C–C bond cleavage, result in modest yields and diastereoselectivities for the desired β-hydroxy-α-amino acids.^17^

In 2023, through the repurposing and engineering of naturally occurring PLP-dependent tryptophan synthases that use a two-electron mechanism for substrate activation, our group introduced the first examples of unnatural pyridoxal radical enzyme activity, allowing intermolecular C–C bond forming reactions to occur with excellent stereocontrol.^19a,b^ Subsequently, our lab^19c,d^ and the Hyster lab^19e^ independently reprogrammed PLP-dependent threonine aldolases to catalyze intermolecular C–C bond formation at the α-position of amino acid donors via a radical mechanism. Our recent work demonstrates that the concept of non-native pyridoxal radical biocatalysis is applicable to a wider range of PLP enzymes with diverse activation mechanisms.^19f,g^ Together, these novel modes of pyridoxal radical biocatalysis,^19^ which were previously unknown in both organic chemistry and enzymology, expand the rapidly growing repertoire of enzyme-catalyzed stereoselective radical transformations.^20–27,28^ Mechanistically, these cooperative photobiocatalytic radical C–C coupling reactions represent some of the earliest demonstrations that transient, externally generated radicals can enter the enzyme and engage with persistent enzymatic intermediates to enable stereoselective C–C bond formation, heralding a new class of cooperative catalytic reactions involving enzymes. Although prior studies from our laboratory^19c,d^ and that of Hyster^19e^ enabled access to α-tetrasubstituted amino acids, the preparation of such α-tetrasubstituted amino acids with adjacent stereocenters was unknown. In particular, although the protocols from our lab^19c^ and Hyster^19e^ allowed the biocatalytic synthesis of β-branched-α-amino acids with a trisubstituted α carbon with up to 76% yield (91:9 d.r., 99:1 e.r.)^19c^ and 10% yield (3.9:1 d.r., >99:1 e.r.)^19e^, respectively, these approaches do not extend to the synthesis of β-branched-α,α-disubstituted α-amino acids, further underscoring the challenge in the stereoselective assembly of these densely substituted molecular architectures.

We questioned whether this radical PLP enzyme platform could be leveraged to enable efficient, diastereo- and enantioselective C–C coupling for constructing contiguous tri- and tetrasubstituted stereocenters, thereby enabling the convergent and stereocontrolled synthesis of challenging β-branched-α-tetrasubstituted amino acids. The abbreviated mechanism is described in Scheme 1. Transimination of the enzymatic internal aldimine (**I**) with a non-native α-substituted α-amino acid substrate furnishes an external aldimine (**II**). Subsequent α-deprotonation would generate the key persistent quinonoid species (**III**). At this stage, the externally formed prochiral secondary alkyl radical **IV**, generated via single-electron oxidation of the racemic organoboron reagent ((*rac*)-**1**), would add to the Cα of quinonoid **III**, furnishing a delocalized *N*-centered radical intermediate (**V**). Further single-electron oxidation of **V** would provide a new external aldimine (**VI**), which would undergo transimination with the conserved lysine residue would release the ncAA product **3** and regenerate the enzyme.

We envisioned that photoredox-mediated generation of prochiral free radicals outside the enzyme active site would allow both (*S*)- and (*R*)-**1** to be converted, thus enabling the enantioconvergent transformation of racemic secondary alkylboron reagents (**1**). Critically, the radical addition step involving prochiral radical **IV** to quinonoid **III** constitutes the enantio- and diastereoselectivity-determining step, setting two vicinal stereocenters in a single bond forming event. We postulated that directed evolution could afford improved radical PLP enzyme variants capable of imposing excellent stereocontrol over the newly formed stereochemical dyad. Herein, we report the development of engineered threonine aldolase variants to enable the stereoselective synthesis of β-branched-α-tetrasubstituted amino acids. Variants evolved for this transformation also display enhanced activity toward a broader range of α-tri- and α-tetrasubstituted amino acids.

## Results and discussion

To commence this study, we performed oxidative photobiocatalytic coupling of racemic secondary alkyltrifluoroborate salt (**(*rac*)**-**1a**) and d-alanine (**2a**) using Co(NH_3_)_6_Cl_3_ as the stoichiometric oxidant^19c^ and evaluated a panel of *Thermotoga maritima* threonine aldolase (*Tm*TA) variants (Table 1). In the presence of 0.5 mol% wild-type *Tm*TA, the desired oxidative coupling product **3a** formed in 41% yield, 88:12 d.r. and >99:1 e.r., confirming feasibility of enzymatic construction of sterically hindered contiguous stereocenters (Table 1, entry 1). Use of previously engineered *Tm*TA variants, including *Tm*TA W86N, *Tm*TA W86E and *Tm*TA W86Q resulted in similar yields and stereoselectivities as wt *Tm*TA (Table 1, entries 2–4). It was found that *Tm*TA E88T furnished **3a** with an enhanced yield of 59% and similar diastereoselectivity (89:11 d.r. and >99:1 e.r., entry 5). Furthermore, the E88T H83F double mutant catalyzed the production of **3a** with simultaneously improved activity and diastereoselectivity (58% yield, 92:8 d.r., >99:1 e.r., entry 6). However, using *Tm*TA E88T H83F as the template, subsequent mutagenesis and screening did not lead to further improvements, indicating that this double mutant represented a local optimum in the fitness landscape.^19a–19b^

We next combined beneficial mutations W86Q and E88T by site-directed mutagenesis. The double mutant TmTA W86Q E88T provided 3a in 58% yield, 92:8 d.r. and >99:1 .e.r. (Table 1, entry 7). To further improve enzyme activity and diastereoselectivity, using TmTA W86Q E88T as the parent, we turned to an in-house built, high-throughput photobiocatalysis screening platform consisting of a 24-tip horn-based high throughput cell lysis and a 96-position LED array-based parallel photoreactor. Using the 22c-trick method,^30^ site-saturation mutagenesis (SSM) libraries were generated and screened. A total of 90 clones were assessed in a 96-well plate using our high-throughput platform. From the TmTA W86Q E88T H83X library, the most active variant was found to be a double mutant TmTA W86Q E88A carrying an unexpected mutation E88A, resulting in significantly enhanced enzyme activity and diastereoselectivity (Table 1, entry 8, 73% yield, 93:7 d.r. and >99:1 e.r.). Using TmTA W86Q E88A as the parent, another round of SSM and screening led to the identification of F85M as the beneficial mutation, affording TmTA W86Q E88A F85M (TmTA^QAM^) with further improved activity and diastereoselectivity (entry 9). Using 0.5 mol % triple mutant TmTA^QAM^, 3a formed in 75% yield, 94:6 d.r. and >99:1 e.r. (entry 9). Importantly, carrying out this photobiocatalytic C–C coupling with 0.1 mol% TmTA^QAM^ afforded 3a in 53% yield and identical diastereo- and enantioselectivity (94:6 d.r. and >99:1 e.r.). At the same enzyme loading, TmTA W86Q E88A provided 3a in 49% yield, 92:8 d.r. and >99:1 e.r. These low biocatalyst loading results further confirmed the improved activity and diastereoselectivity of the final triple mutant TmTA^QAM^. Using L-alanine (L-2a) in lieu of d-alanine (d-2a), 3a still formed in 55% yield, 94:6 d.r. and >99:1 e.r. (entry 10), highlighting the ability of TmTA^QAM^ to transform either enantiomeric form of alanine. Finally, to shed light on the effects of newly introduced beneficial mutations E88A and F85M, we prepared single mutants of wt TmTA (entries 11 and 12). It was found that both TmTA E88A and TmTA F85M provided improved yields and slightly enhanced diastereoselectivities relative to wt TmTA, thus confirming their role in promoting this radical C–C coupling.

The photocatalyst effect in this PLP enzyme-catalyzed construction of contiguous tri- and tetrasubstituted stereocenter is also noteworthy (Table 2). While 4CzIPN remained an effective photocatalyst when used at a 10 mol % loading (70% yield, 94:6 d.r. and >99:1 e.r., Table 2, entry 2), Ru-based photosensitizer Ru(bpy)_3_Cl_2_ was found to be the most efficient, giving rise to **3a** in 75% yield, 94:6 d.r. and >99:1 e.r. (Table 2, entry 1). Previously used Ir-based photocatalyst *fac*-Ir(ppy)_3_ only provided **3a** in 50% yield and 93:7 d.r. (entry 3). Other commonly used organic photosensitizer, such as rhodamine B, fluorescein and rose bengal also provided inferior results (entries 4–6). In the absence of PLP enzyme *Tm*TA^QAM^ or visible light irradiation, formation of the oxidative coupling product **3a** was not observed, confirming the dual photobiocatalytic nature of this transformation (entries 7–8). Interestingly, further studies showed that in the absence of Ru(bpy)_3_Cl_2_, **3a** still formed in 13% yield, albeit with a reduced d.r. of 89:11 (entry 9). This result indicated a minor light-driven pathway leading to **3a** independent of the added photocatalyst is operative under these conditions. In the absence of Co(NH_3_)_6_Cl_3_, **3a** also formed in 15% yield, 94:6 d.r. and >99:1 e.r. (entry 10), indicating the presence of an alternative oxidant. Furthermore, in the absence of added 10 mol% free PLP, the yield of **3a** was drastically reduced to 43%, although the diastereo- and enantioselectivity remained unchanged (entry 11). Together, results in entries 9–11 suggested a previously underappreciated role of added PLP under these photobiocatalytic conditions. Finally, further lowering the enzyme loading to 0.1 mol%, **3a** still formed in 53% yield, 94:6 d.r. and >99:1 e.r. (entry 12), indicating a turnover number of 530 and an improved enzyme activity. Slightly different diastereoselectivities were observed with photocatalysts detailed in Table 2 and further studies to shed light on this effect are underway.


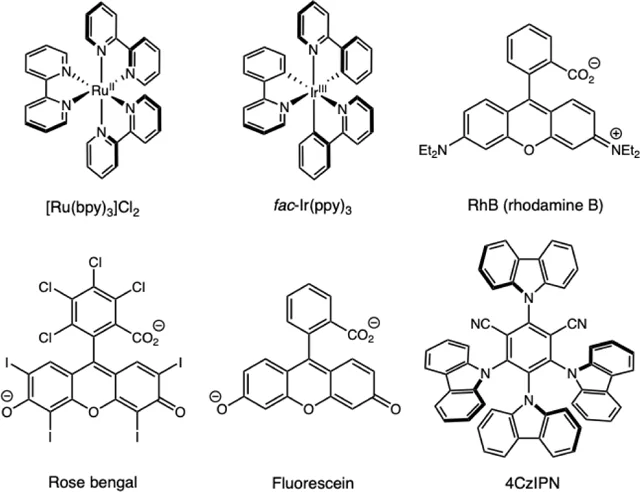


With the optimized reaction conditions in hand, we examined the substrate scope of this diastereo- and enantioselective photobiocatalytic coupling using 0.5 mol% *Tm*TA^QAM^ (Table 3). It was found that substrates bearing an *ortho*- (**3b**), a *meta*-(**3c**) and a *para*-substituent (**3d**) on the aromatic ring were well tolerated, providing the corresponding ncAAs in good yields and stereoselectivities. Among these, the inclusion of an *ortho*-substituent (**3b**) resulted in further improved diastereocontrol (99:1 d.r.). Electro-donating groups such as a methoxy group (**3e**), as well as electro-withdrawing groups such as a fluorine (**3f**), a chlorine (**3g**), and a bromine (**3h**) were also tolerated by the engineered PLP enzyme. Larger aromatic substituents such as the 2-naphthyl group (**3i**) was also readily accepted by *Tm*TA^QAM^. Furthermore, ncAAs possessing an extended β-alkyl substituents including an ethyl (**3j**) and a propyl (**3k**) could also be readily synthesized with good diastereo- and enantioselectivities. Cyclic secondary alkyl radical precursors, including an indanyl (**3l**), a 1-tetrahydronaphthyl (**3m**) and a chromanyl (**3n**) group, were also readily transformed under the current photobiocatalytic conditions, furnishing the corresponding ncAA products with a cyclic substituent with excellent diastereo- and enantioselectivities. Commercially available 2-aminobutanoic acid (**2o**), a non-native amino acid donor featuring a larger α-ethyl group, was also accepted by *Tm*TA^QAM^, giving rise to the ncAA product with modest initial activity and high levels of diastereo- and enantioselectivity. Finally, this photobiocatalytic amino acid synthesis could be conveniently scaled up to a 1.0 mmol scale, providing the ncAA product **3a** in 67% yield (153 mg), 93:7 d.r. and >99:1 e.r., further showcasing the synthetic utility of the current method.

To date, unnatural photobiocatalytic activities are usually characterized by modest turnover efficiency. To enhance the synthetic utility of these novel enzymatic functions, it is imperative to further improve their turnover efficiency. As our newly engineered PLP biocatalyst variant *Tm*TA^QAM^ displayed increased activity in the stereoselective construction of ncAAs possessing vicinal tri- and tetrasubstituted stereocenters, we posited that *Tm*TA^QAM^ might exhibit enhanced catalytic activity towards a wider range of substrates. To this end, we evaluated *Tm*TA^QAM^-catalyzed conversion of primary benzyl substrate **4a** and glycine (**2c)** into phenylalanine **5a** at varying enzyme loadings (Table 4). Indeed, at 1 mol% enzyme loading, *Tm*TA^QAM^ afforded **5a** in 85% yield and 98.5:1.5. e.r. (Table 4, entry 1). These results are superior to our previously reported *Tm*TA W86N, which gave rise to **5a** in 78% yield and 97:3 e.r. (entry 2), suggesting increased enzyme activity of our newly evolved triple mutant *Tm*TA^QAM^. Further lowering the loading of *Tm*TA^QAM^ from 1.0 mol% to 0.01 mol%, a turnover number of 3,100 was observed (entries 2–4). To our knowledge, this enzyme turnover efficiency represents the highest TTN in new-to-nature photobiocatalytic processes. We note that higher TTNs were observed with α-monosubstituted α-amino acids. For more challenging α,α-disubstituted α-amino acids, TTN values determined under turnover limiting conditions were found to be 500–910 (*vide infra*).

We next surveyed the catalytic efficiency of *Tm*TA^QAM^ across a broader range of substrates (Table 5). Benzyltrifluoroborate substrates possessing an *ortho*- (**4b**), a *meta*-(**4c**) and a *para*-(**4d**) substituent were all successful converted to the corresponding ncAA products with a TTN ranging from 2,200 to 2,890. Benzyltrifluoroborate with an electron-donating methoxy group (**4e**) and an electro-withdrawing trifluoromethyl group (**4f**) at the *para*-positions were also readily accommodated, delivering the corresponding ncAA product with 1,900 and 1,260 TTN, respectively. 2-Naphthylmethylboron substrate was also a viable substrate, providing **4g** with 1,280 TTN and 97:3 e.r. Furthermore, when d-alanine (**2a**) was used as the amino acid donor, the same set of organoboron substrates could be transformed to their corresponding α-tetrasubstituted ncAA products with a TTN of 500–910.

To further probe the mechanism of this photobiocatalytic oxidative coupling, we prepared a cyclopropyl containing radical clock substrate (*rac*)-**1p** and subjected it to standard photobiocatalytic reaction conditions (Scheme 2). Both the cyclopropyl ring opening product **7a** and the cyclopropyl retained product **7b** were obtained, with a **7a**:**7b** ratio determined to be 5:1 based on ^1^H NMR analysis of unpurified reaction mixture. The formation of the ring opened homoallyl product **7a** is consistent with the involvement of radical intermediate.^31^ Our local coupled-cluster (DLPNO-CCSD(T)) calculations suggested a low activation barrier of 13.5 kcal/mol for the ring opening of cyclopropyl substituted radical **8b** intermediate, leading to the ring-opened homoallyl radical **8a** that is 5.1 kcal/mol higher in energy than **8b**. Based on these results, we hypothesized that the product distribution of **7a** and **7b** is likely controlled by the enzyme and/or the intrinsic activity differences of **7a** and **7b** in the C–C bond forming radical addition step.

Our studies in Table 2 revealed the underappreciated role of added free PLP under these oxidative coupling conditions. To further probe the role of PLP, we first performed TEMPO trapping experiment for the reaction of **1a** and PLP under visible light irradiation at 440 nm (Table 6). In the presence of 2 equiv TEMPO, TEMPO trapping product **9** formed in 87% yield as determined by LC-MS analysis using 2-phenylbutyric acid as an internal standard. In the absence of visible light irradiation, the formation of **9** was not observed. Together, these results indicated that benzyl radical could form directly from **1a** and PLP under visible light irradiation.

We further employed spin trap EPR spectroscopy to investigate radicals generated under catalytically relevant conditions (Figure 1, see the SI for further details). A set of samples were subjected to irradiation at 440 nm at the presence of 5,5-dimethyl-1-pyrroline-*N*-oxide (DMPO). Strong spin-trapped radical signals were detected for the reaction mixtures containing **1a**, PLP and DMPO under irradiation. The presence of d-alanine (**D-2a**) does not affect the EPR signal intensity, indicating that either free PLP or the PLP aldimine adduct derived from **D-2a** could trigger benzyl radical generation from **1a** under photochemical conditions. This finding is in contrast to our previous results on reductive radical generation from benzyl 2,4,6-triphenylpyridinium salt, in which only the PLP aldimine species and not free PLP could induce benzyl radical formation upon visible light irradiation. The EPR parameters of the spin-trapped radical, simulated to be *g* = 2.00723, *A* ^14^N = 59.1 MHz (or 2.10 mT) and *A* ^1^H = 40.8 MHz (or 1.45 mT), are consistent with a carbon centered radical being trapped and almost identical to the same species in our previous report.^19b, 32–33^ Together, these studies unrevealed the unexpected role of free PLP in the generation of benzyl radicals when irradiated at 440 nm.

To gain insights into the origin of enantio- and diastereoselectivity in the C–C bond forming radical addition step, we performed classical molecular dynamics (MD) simulations to investigate interactions between key active site residues, the quinonoid, and the benzyl radical. Since the PLP cofactor in wt *Tm*TA resides at the interface of two polypeptide chains,^34^ we used a dimeric *Tm*TA^QAM^ model for MD simulations. Three independent 500 ns MD replicas were performed using the quinonoid intermediate derived from alanine **2a** (Figure 2A).

Throughout the MD simulations, PLP remained tightly bound in the active site, with a mass-weighted average RMSF (root-mean-square fluctuation) of just 0.18 Å for the quinonoid, indicating structural rigidity. Multiple hydrogen bonding interactions were observed, including those between the PLP 5’-phosphate and K199/R231’, the substrate carboxylate and R171/R316, and the PLP pyridine nitrogen and D168 (see Figures S8–S11 for details). The (*Re*)-face of the prochiral quinonoid C⍺ is sterically shielded by K199, R171, and R231’ (see Figure S12 for the distance distributions from C⍺ to these residues). As a result, the addition of the benzyl radical occurs at the exposed (*Si*)-face of the quinonoid, consistent with the experimentally observed enantioselectivity favoring the formation of L-ncAA products.

To elucidate the origin of diastereoselectivity in the radical addition step, we modeled near-attack conformations (NACs) of the prochiral α-methyl benzyl radical derived from **1a** approaching the C⍺ of the quinonoid intermediate. These simulations were performed by restraining the C(benzyl)⋯C⍺(quinonoid) distance to 2.8–3.0 Å using a harmonic potential of 100 kcal·mol^−1^·Å^−2^ (Figure 2(B)). We separately analyzed NAC frames in which the (*Si*)- or (*Re*)-face of the benzyl radical was oriented toward the quinonoid, corresponding to formation of the major and minor diastereomers (2*S*,3*S*)-**3a** and (2*S*,3*R*)-**3a**, respectively. A consistent arginine/π interaction^35^ was observed between the side chain of R316 and the Ph ring of the benzyl radical. This interaction is stronger in the (*Si*)-face addition trajectories, as evidenced by a shorter median distance between the benzene ring centroid and the guanidinium carbon of R316 (π^radical^⋯C_Gdm_^R316^ = 4.2 Å, compared to 4.4 Å in the (*Re*)-face addition) and a higher frequency of this distance being within 5 Å (78.9% vs. 58.2 %). The weaker interaction in the (*Re*)-face addition pathway is attributed to unfavorable eclipsed repulsion between the ⍺-methyl group of the benzyl radical and the methyl group at the quinonoid C⍺. In contrast, the (*Si*)-face addition places the less sterically demanding benzylic hydrogen coplanar with the quinonoid methyl group, avoiding this steric clash and favoring the formation of the major diastereomer.

## Conclusion

In summary, through cooperative catalysis between PLP enzymes and photosensitizers, we developed a protecting-group-free, biocatalytic oxidative coupling strategy for the enantioselective synthesis of α-tetrasubstituted non-canonical amino acids. Directed evolution of a thermophilic threonine aldolase provided a highly efficient and diastereoselective radical C–C bond forming enzyme, enabling access to β-branched-α-tetrasubstituted amino acids possessing contiguous stereocenters. Furthermore, this evolved triple mutant PLP enzyme achieved a total turnover number of up to 3,100 under photobiocatalytic reaction, representing a record for unnatural photobiocatalytic transformations and highlighting the feasibility of engineering highly active radical enzymes. Classical MD simulations revealed that C⍺ stereochemistry is governed by selective radical addition to the exposed (*Si*)-face of the quinonoid intermediate. Diastereocontrol arises from a key arginine/π interaction, which orients the sterically least hindered benzylic C–H of the radical toward ⍺-substituent of the quinonoid, favoring the formation of the major diastereomer. Additionally, TEMPO trapping and EPR experiments revealed an unexpected role of free PLP in promoting carbon-centered radical generation from organoboron substrates under photochemical conditions, underscoring the rich and previously underexplored photochemistry of this naturally occurring pyridoxal cofactor. Together, this study showcases the potential of pyridoxal radical biocatalysis to enable the synthesis of valuable non-canonical amino acids through reaction mechanisms previously unknown in both synthetic organic chemistry and native enzymology. Ongoing efforts in our laboratory aim to expand this strategy to a broader range of traditionally two-electron PLP enzymes for novel asymmetric intermolecular C–C bond-forming transformations.

## Supplementary Material

SI

ASSOCIATED CONTENT

Supporting Information

Experimental procedures, DNA and protein sequences, characterization data, HPLC traces and NMR spectra (PDF).

The Supporting Information is available free of charge on the ACS Publications website.

## Figures and Tables

**Figure 1.**
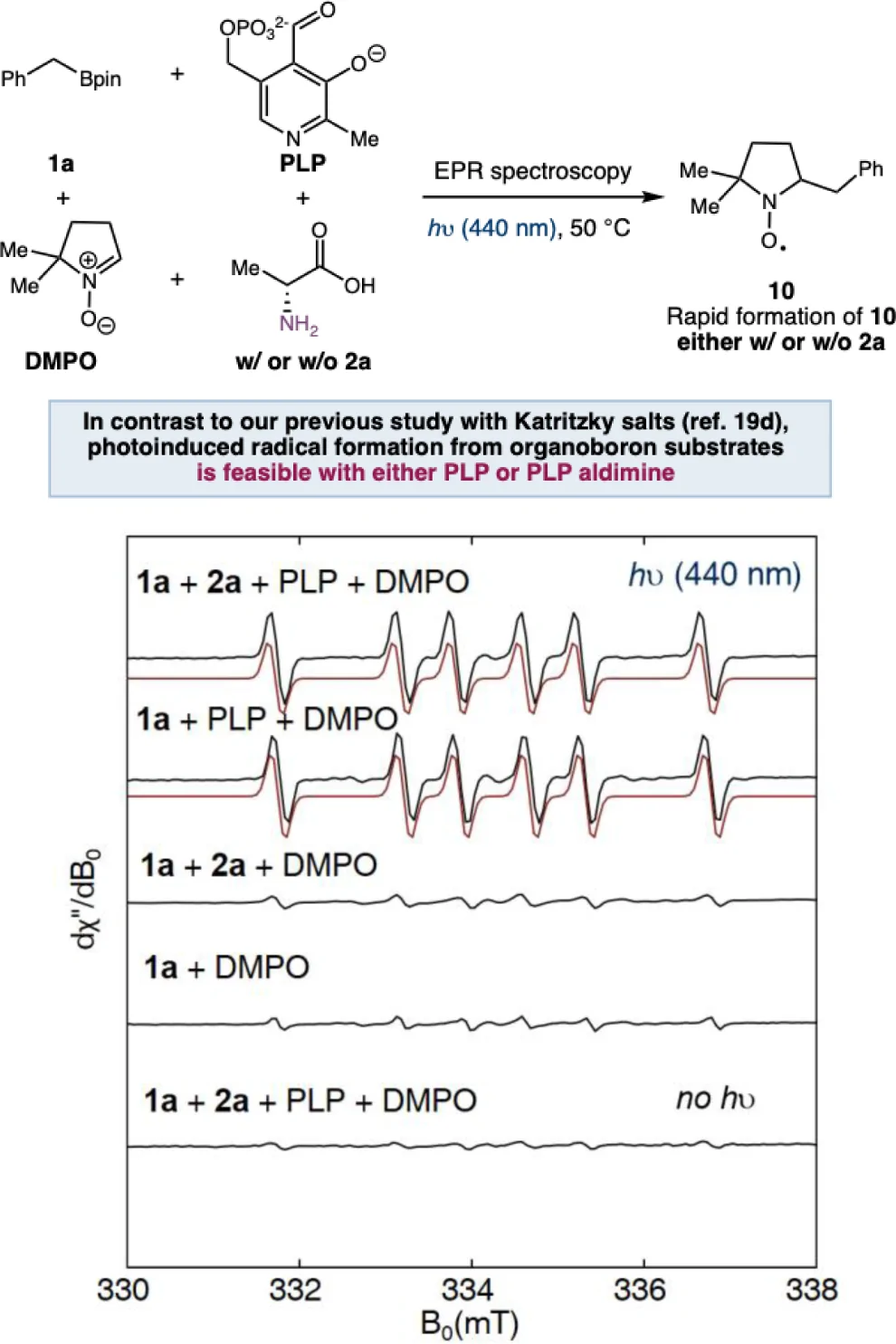
X-band CW EPR (298 K, 2 mW) spectra of DMPO-spin trapped reaction mixtures before and after blue light irradiation. Red trace: simulation of the spin trapped benzyl radical species (**10**) using *g* = 2.00723, *A* ^14^N = 59.1 MHz (or 2.10 mT), *A* ^1^H = 40.8 MHz (or 1.45 mT).

**Figure 2.**
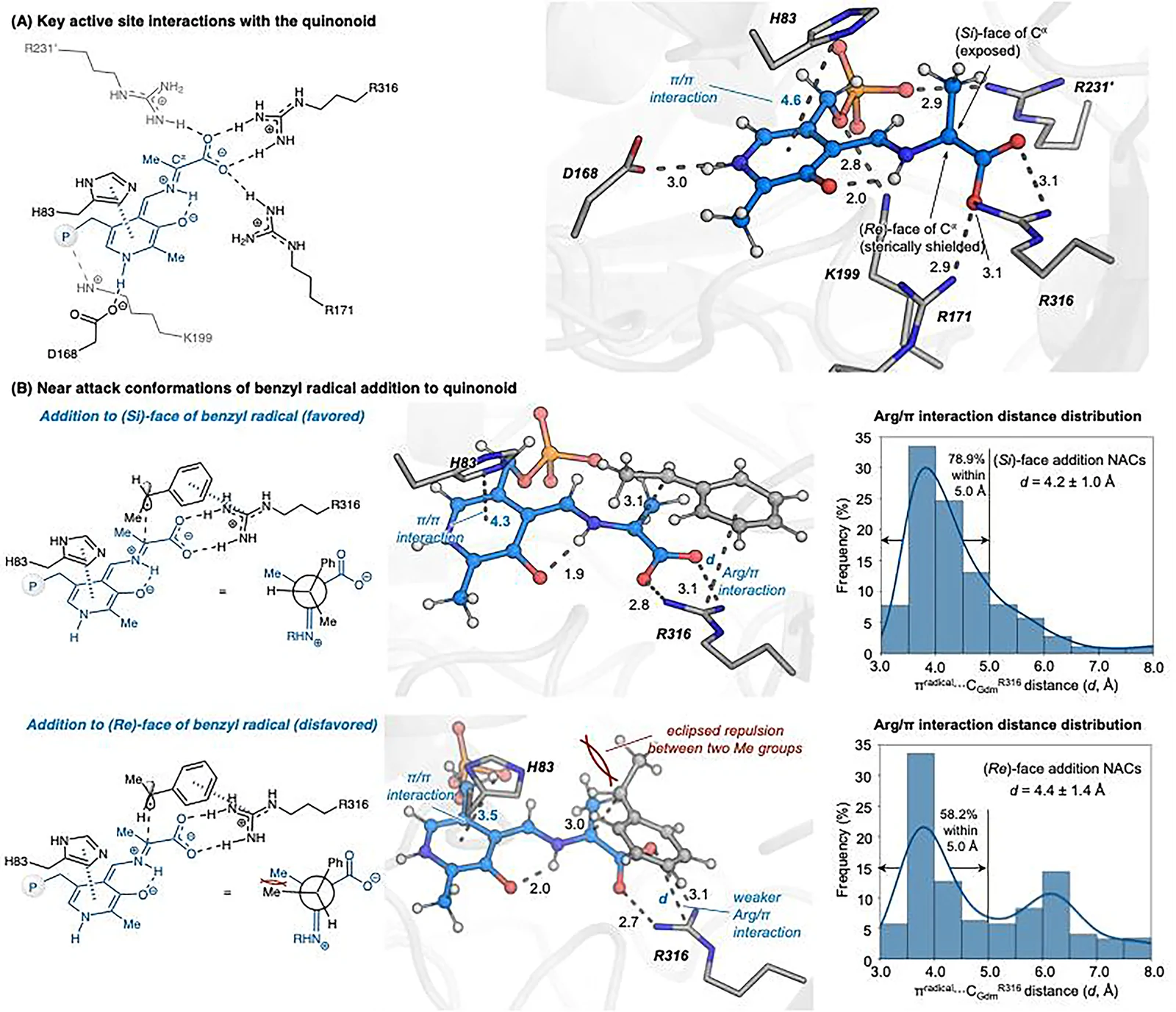
Computational studies on photobiocatalytic radical coupling: Understanding the origin of enzymatic stereocontrol in the radical addition using classical molecular dynamics (MD) simulations.

**Scheme 1.**
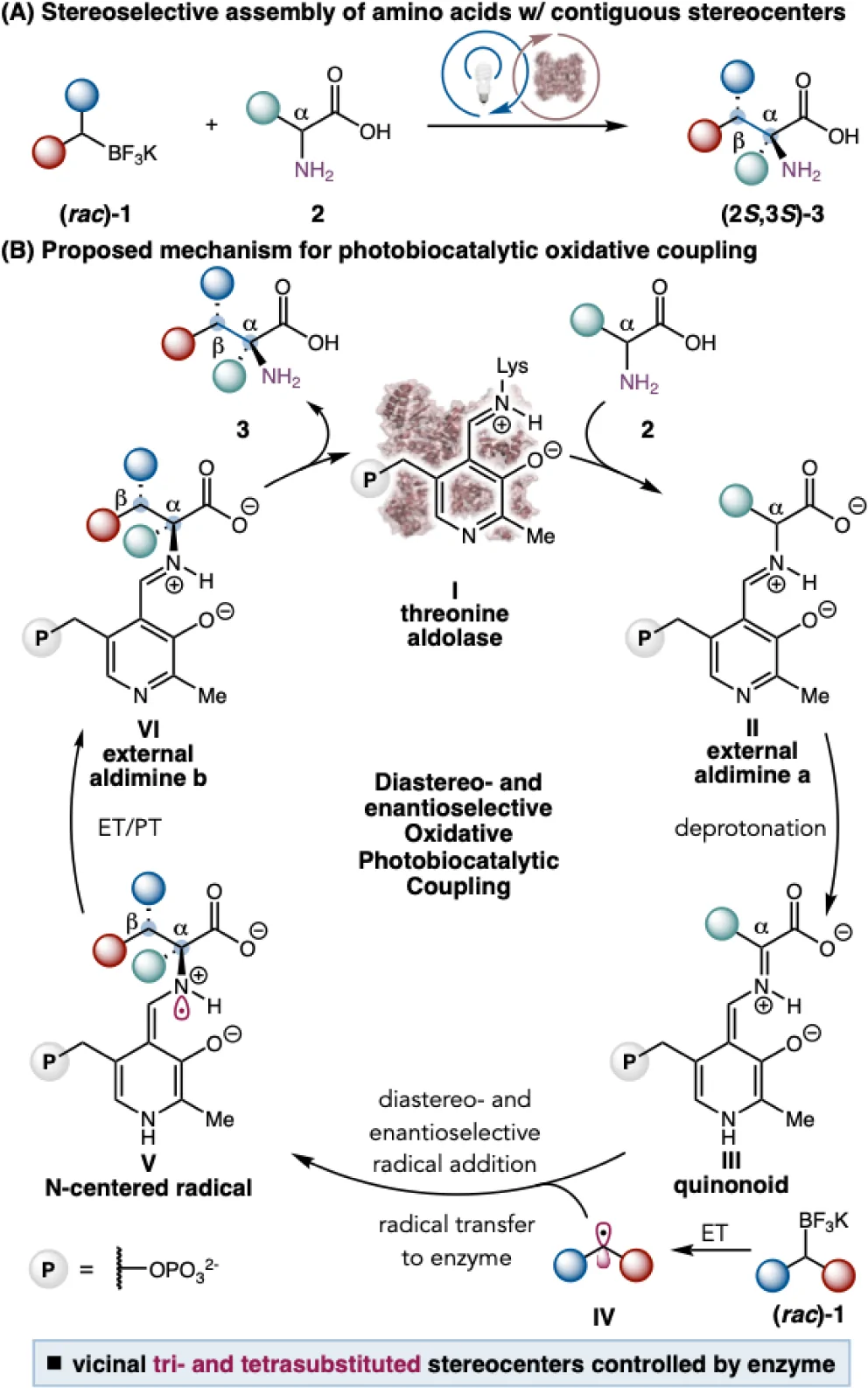
PLP-Dependent Threonine Aldolase-Catalyzed Oxidative Coupling for the Stereoselective, Protecting-Group-Free Synthesis of Non-Canonical Amino Acids with Contiguous Tri- and Tetrasubstituted Stereocenters.

**Scheme 2.**
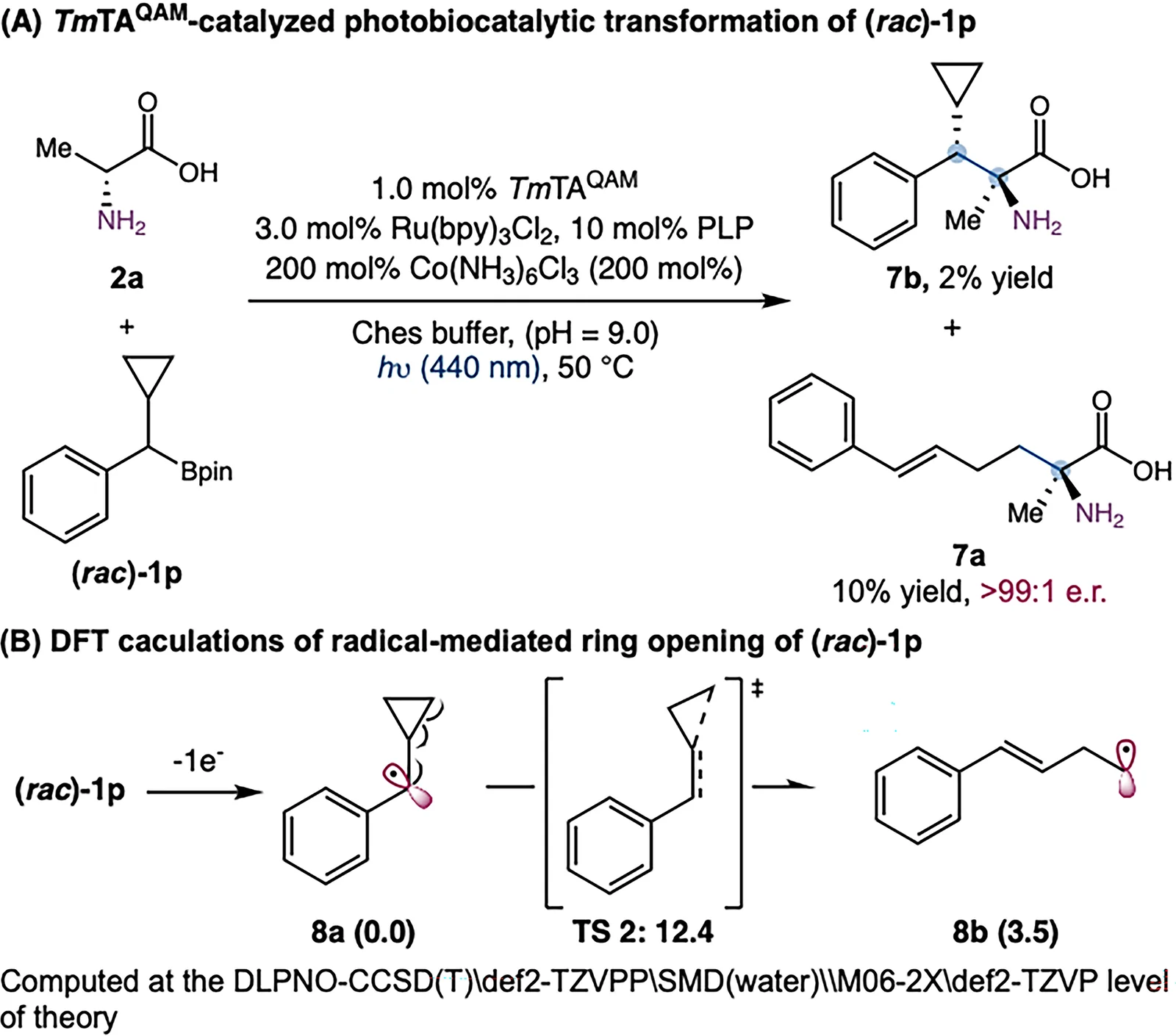
Photobiocatalytic Transformation of Radical Clock Substrates

**Table 1.** Discovery and Enzyme Engineering of *Tm*TA of Oxidative Photobiocatalytic Enantioselective Coupling Reactions^a^

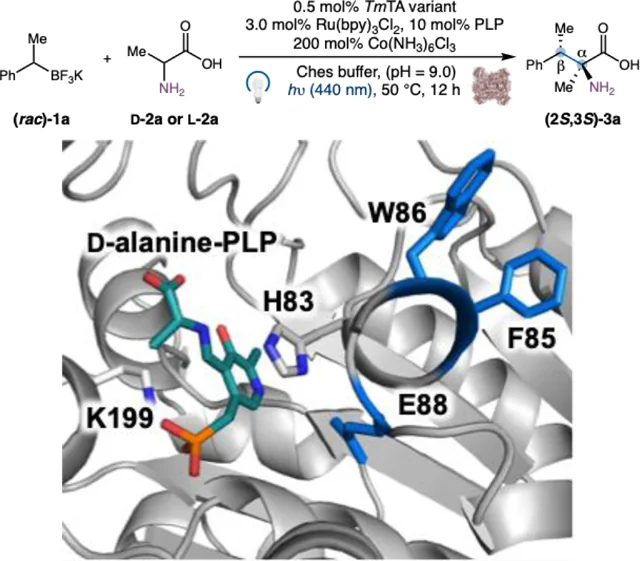					
entry	2a	*Tm*TA variant	yield (%)	d.r.	e.r.
1	**D-2a**	wt *Tm*TA	41%	88:12	>99:1
2	**D-2a**	*Tm*TA W86N	44%	89:11	>99:1
3	**D-2a**	*Tm*TA W86E	46%	90:10	>99:1
4	**D-2a**	*Tm*TA W86Q	46%	90:10	>99:1
5	**D-2a**	*Tm*TA E88T	59%	89:11	>99:1
6	**D-2a**	*Tm*TA E88T H83F	58%	92:8	>99:1
7	**D-2a**	*Tm*TA W86Q E88T	58%	92:8	>99:1
8	**D-2a**	*Tm*TA W86Q E88A	73% (49%) ^b^	93:7 (92:8) ^b^	>99:1 (>99:1) ^b^
9	**D-2a**	*Tm*TA W86Q E88A F85M (*Tm*TA^QAM^)	75% (53%)^b^	94:6 (94:6) ^b^	>99:1 (>99:1) ^b^
10	**L-2a**	*Tm*TA^QAM^	55%	94:6	>99:1
11	**D-2a**	*Tm*TA E88A	73%	90:10	>99:1
12	**D-2a**	*Tm*TA F85M	66%	90:10	>99:1

a Reaction conditions: **(*rac*)-1a** (6.0 mM), **D**-**2a** (30.0 mM), 0.5 mol% *Tm*TA (30 μM), 3.0 mol% Ru(bpy)_3_Cl_2_ (0.18 mM), 200 mol% Co(NH_3_)_6_Cl_3_ (12.0 mM), PLP (0.60 mM), 100 mM Ches buffer (pH = 9.0), 50 °C, *hν* (440 nm, 45 W), 12 h.

b 0.1 mol% *Tm*TA variant was used. *Tm*TA active site structure was made using PDB # 1LW5.

**Table 2.** Further Evaluation of Oxidative Photobiocatalytic Enantioselective Coupling Reactions^a^

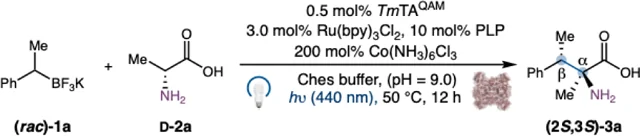					
entry	deviation from standard conditions	yield (%)	TTN	d.r.	e.r.
1	none	75	150	94:6	>99:1
2	10 mol% 4CzIPN	70	140	94:6	>99:1
3	2 mol% *fac*-Ir(ppy)_3_	50	100	93:7	>99:1
4	10% rhodamine B	68	136	91:9	>99:1
5	10 mol% fluorescein	64	128	93:7	>99:1
6	10 mol% rose bengal	51	152	95:5	>99:1
7	no *Tm*TA^QAM^	0	0	-	-
8	no *hν*	0	0	-	-
9	no Ru(bpy)_3_Cl_2_	13	26	89:11	>99:1
10	no Co(NH_3_)_6_Cl_3_	15	30	94:6	>99:1
11	no PLP	43	86	94:6	>99:1
12	0.1 mol% *Tm*TA^QAM^	53	530	94:6	>99:1

a Reaction conditions: **(*rac*)-1a** (6.0 mM), **2a** (30 mM), 0.5 mol% *Tm*TA^QAM^ (30 μM), 3.0 mol% Ru(bpy)_3_Cl_2_ (0.18 mM), 200 mol% Co(NH_3_)_6_Cl_3_ (12.0 mM), PLP (0.60 mM), 100 mM Ches buffer (pH = 9.0), *hν* (440 nm, 45 W), 50 °C, 12 h.

**Table 3.** Substrate Scope of *Tm*TA^QAM^ Catalyzed-Diastereo- and Enantioselective Oxidative Coupling Reactions for the Synthesis of Non-Canonical Amino Acids Bearing Contiguous Tri- and Tetrasubstituted Stereocenters^a^

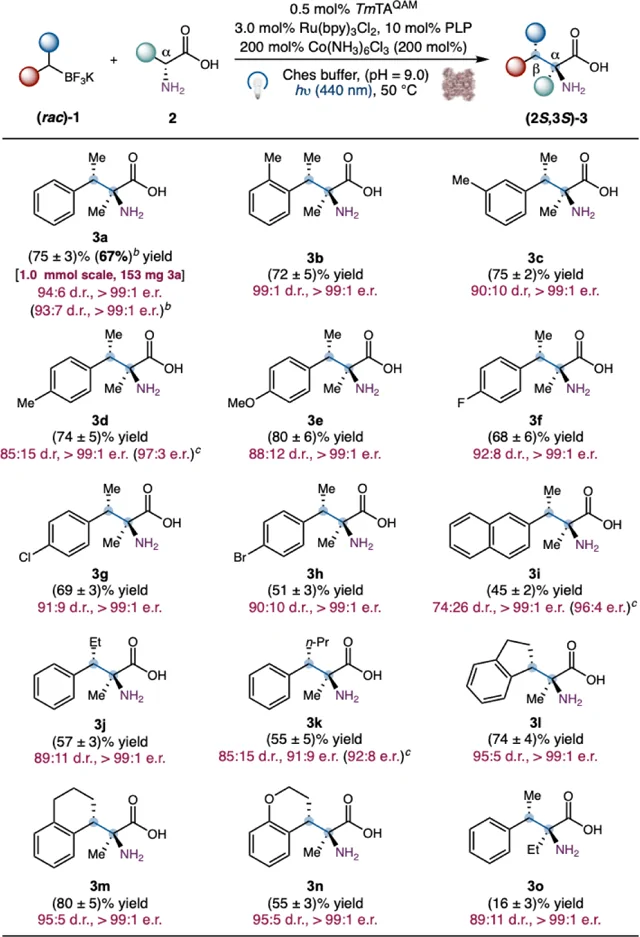

a Reaction conditions: **(*rac*)**-**1** (6.0 mM), **2** (30 mM), 0.5 mol% *Tm*TA^QAM^ (30 μM), 3.0 mol% Ru(bpy)_3_Cl_2_ (0.18 mM), 200 mol% Co(NH_3_)_6_Cl_3_ (12.0 mM), PLP (0.60 mM), 100 mM Ches buffer (pH = 9.0), *hν* (440 nm, 45 W), 50 °C, 12 h.

**Table 4.** *Tm*TA^QAM^ Turnover Number in Photobiocatalytic Asymmetric Oxidative α-Benzylation of Glycine^a^

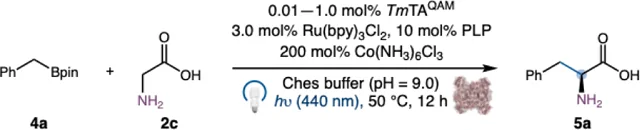				
entry	*Tm*TA^QAM^ loading	yield (%)	TTN	e.r.
1	1.0 mol%	85	85	98.5:1.5
2	0.5 mol%	78	156	98.5:1.5
3	0.1 mol%	69	690	98.5:1.5
4	0.01 mol%	31	3,100	98:2

a Reaction conditions: **4a** (6.0 mM), **2c** (60 mM), 0.01–1 mol% *Tm*TA^QAM^ (0.6–60 μM), 3.0 mol% Ru(bpy)_3_Cl_2_ (0.18 mM), 200 mol% Co(NH_3_)_6_Cl_3_ (12.0 mM), PLP (0.60 mM), 100 mM Ches buffer (pH = 9.0), *hν* (440 nm, 45 W), 50 °C, 12 h.

**Table 5.** Improved Total Turnover Numbers in Stereoselective Oxidative Photobiocatalytic Coupling with *Tm*TA^QAMa^

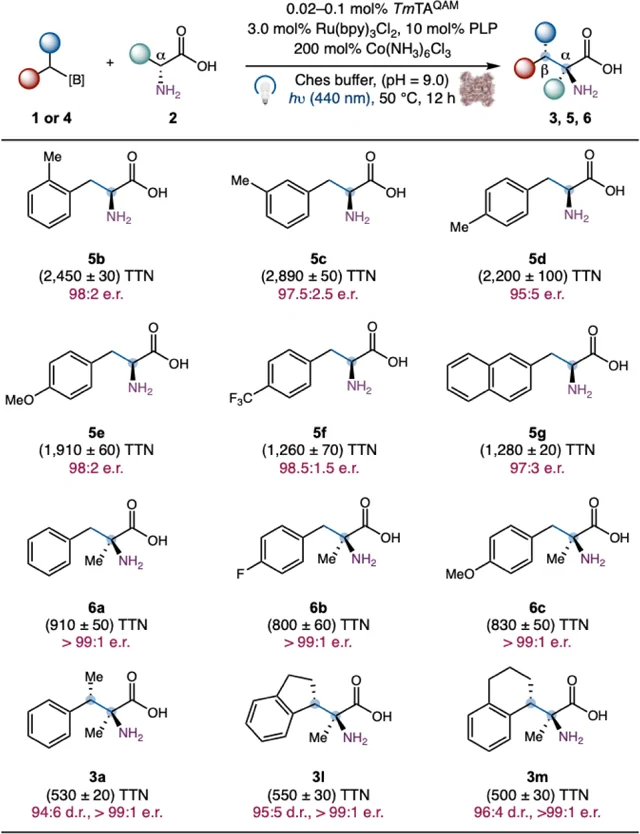

a Reaction conditions: **4** (6.0 mM), **2** (60 mM), 0.02–0.1 mol% *Tm*TA^QAM^ (1.2–6 μM), 3.0 mol% Ru(bpy)_3_Cl_2_ (0.18 mM), 200 mol% Co(NH_3_)_6_Cl_3_ (12.0 mM), PLP (0.60 mM), 100 mM Ches buffer (pH = 9.0), *hν* (440 nm, 45 W), 50 °C, 12 h.

**Table 6.** TEMPO-Trapping Experiments^a^

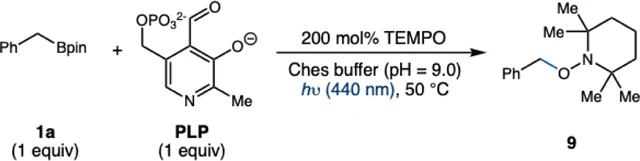		
entry	variation from the standard conditions	yield of 9
1	none	87%
2	5.0 equiv **2a** added	84%
3	no PLP	<1%
4	no PLP, 5.0 equiv **2a** added	<1%
5	no *h*ν (440 nm)	0%

a Reaction conditions: **1a** (6.0 mM), PLP (6.0 mM), TEMPO (12.0 mM), 100 mM Ches buffer (pH = 9.0), *hν* (440 nm, 45 W), 50 °C, 12 h.
