# Supplementary material for: Directed Evolution of Threonine Aldolases for the Photobiocatalytic Synthesis of β-Branched-α-Tetrasubstituted and Other Non-Canonical Amino Acids
Source: J Am Chem Soc. Author manuscript; Available in PMC 2026 Oct 20. (PMC13679966; doi:10.1021/jacs.6c03303)

Supporting Information for

Directed Evolution of Threonine Aldolases as Radical C–C Bond Forming Enzymes for Photobiocatalytic Synthesis of β-Branched-α-Tetrasubstituted and Other Non-Canonical Amino Acids

Tian-Ci Wang,[1,‡] Xiangyu Zhang,[2,‡] Zheng Zhang,[1,‡] Guodong Rao,[3] Zhiyu Bo,[1] Pei-Pei Xie,[2] Jiedong Li,[1] Qiang Yang,[4] Xiaoyong Li,[4] R. David Britt,[3] Jiang Wang,[4] Peng Liu[2,*] and Yang Yang[1,5,*]

[1]Department of Chemistry and Biochemistry, University of California Santa Barbara, Santa Barbara, California 93106

[2]Department of Chemistry, University of Pittsburgh, Pittsburgh, Pennsylvania 15260

[3]Department of Chemistry, University of California Davis, Davis, California 95616

[4]Synthetic Molecule Design and Development, Lilly Research Laboratories, Eli Lilly and Company, Indianapolis, Indiana 46285

[5]Department of Bioengineering, University of California Santa Barbara, Santa Barbara, California 93106

[6]Biomolecular Science and Engineering Program, University of California Santa Barbara, Santa Barbara, California 93106

[7]Howard Hughes Medical Institute, University of California Santa Barbara, Santa Barbara, California 93106

‡These authors contributed equally.

*Corresponding author.

Email: yang@chem.ucsb.edu, pengliu@pitt.edu, wang_jiang@lilly.com

Table of contents

I. General methods 3

II. General experimental and analytical procedures 8

III. Selected further results for reaction development 13

IV. DNA and protein sequences 19

V. UV-Vis spectroscopic analysis 29

VI. DMPO spin-trapping EPR experiment 31

VII. Radical clock reaction 32

VIII. Pierce™ BCA protein assay 34

IX. Synthesis and characterization of substrates 35

X. Synthesis and characterization of products 38

XI. HPLC calibration curves of amino acid products 56

XII. HPLC traces for Marfey's analysis and Fmoc chloride derivatization 69

XIII. Computational Studies 95

XIV. References 114

XV. ^{1}H, ^{19}F and ^{13}C NMR spectra of compounds 119

I. General methods

General. All biocatalytic reactions were performed in a reaction tube (13 × 100 mm, 8 mL, Fisherbrand) with magnetic stirring (500 rpm) using Heidolph Hei-Tec stir plates. Kessil LED PR160L-440 nm (max 45 W) was used as the visible light source. Unless otherwise noted, all chemicals and reagents were obtained from commercial suppliers (Sigma Aldrich, VWR, Alfa Aesar, Combi Blocks, Ambeed, Oakwood, and Enamine) and used without further purification. Silica gel chromatography was carried out using AMD Silica Gel 60, 230-400 mesh. ^{1}H and ^{13}C NMR spectra were recorded on a Bruker 400 or 500 MHz nuclear magnetic resonance spectrometer in DMSO-d_6, D_2O or methanol-d_4 (CD_3OD). ^{19}F NMR and ^{11}B NMR spectra (where applicable) were recorded on a Bruker 400 or 500 MHz instrument (^{1}H decoupled). Data for ^{1}H NMR are reported as follows: chemical shift (δ ppm), multiplicity (s = singlet, d = doublet, t = triplet, q = quartet, p = pentet, sext = sextet, m = multiplet, dd = doublet of doublets, dt = doublet of triplets, ddd = doublet of doublet of doublets, brs = broad singlet), coupling constant (Hz), integration. Sonication on a small scale was performed using a BioLogics ultrasonic homogenizer (model 150VT) equipped with a stepped microtip. Sonication on a large scale was performed using a Qsonica Q500 sonicator equipped with a standard probe (tip diameter = ½’’). All IR spectra were recorded on a Thermo Scientific Nicolet iS5 spectrometer (iD5 ATR, diamond). High-resolution mass spectrometry data was obtained at the Mass Spectrometry Facilities at the University of California Santa Barbara. High-resolution accurate mass (HRAM) ESI data was analyzed on a Waters LCT Premier mass spectrometer with a LEAP PAL autosampler with isocratic MeOH flow (no column; direct injection). Molecular formulas (MF) were validated by lock mass calibration to sodiated polyethylene glycol polymer or sodiated monoethyl ether polyethylene glycol polymer standards. High-resolution accurate mass (HRAM) EI data was acquired using a Waters LCT Premier time-of-flight (TOF) mass spectrometer. Masses of positively charged ions were calibrated using a methanol solution of polyethylene glycol or polyethylene glycol monomethyl ether as an internal standard. All samples were dissolved in methanol and were directly infused unless otherwise noted. Synthetic reactions were monitored by thin layer chromatography (TLC, Silicycle TLG-R10014BK-323 gel plates) using a UV-lamp or an appropriate TLC stain for visualization. UV-vis spectra were collected on a UV1800 Shimadzu spectrophotometer.

E. coli cells were grown using Luria-Bertani medium (LB) with 0.05 mg/mL kanamycin (LB_{kan}). Primer sequences for site-directed mutagenesis and site-saturation mutagenesis are provided below. T5 exonuclease, Phusion DNA polymerase, and *Taq* DNA ligase were purchased from New England Biolabs (NEB, Ipswich, MA). Potassium Phosphate Buffer (abbreviated as KPi buffer) was used as a buffering system for protein purification and storage unless otherwise specified.

Chromatography. Analytical reverse phase high performance liquid chromatography-mass spectrometry (HPLC-MS) was carried out using a Shimazu-2040C 3D Plus with a 2020 MS detector and a Kromasil 100-5-C18 column (4.6 × 50 mm, 5 μm) with water (0.1% formic acid) and acetonitrile (0.1% formic acid) as the mobile phase. Yields were determined by calibration curves using 2-phenylbutyric acid as the internal standard. The enantiomeric ratios (e.r.'s) of non-canonical amino acid products were determined by HPLC after derivation with Fmoc-Cl as described below. The diastereomeric ratios (d.r.'s) of non-canonical amino acid products were determined by NMR after the combination of 20 analytical reactions and purification using a reverse phase C18 column.

Reverse-phase HPLC calibration curve development. Stock solutions of authentic products (30 mM in CH_3CN/1 M aq. HCl (v:v = 1:1)) and internal standard (2-phenylbutyric acid) (100 mM in CH_3CN/1 M aq. HCl (v:v = 1:1)) were freshly prepared. To a 1.5 mL microcentrifuge tube were added 30 μL of internal standard stock solution (3 μmol), 20, 40, 60, 80 and 100 μL of product stock solution was added to each tube, respectively, followed by the addition of 950, 930, 910, 890 and 870 μL CH_3CN/1 M aq. HCl to make the total volume to be 1.00 mL. The mixture was vortexed (20 s for 3 times) to ensure good mixing. The mixture was transferred to a 1.5 mL glass vial for HPLC analysis. The calibration curves detailed below plot product yield (y-axis) against the ratio of the peak area of the product to the peak area of the internal standard from HPLC analysis (x-axis). In the development of calibration curves, care was taken such that the concentration of calibration curve samples was close to analyte samples.

Cloning, site-directed mutagenesis and site-saturation mutagenesis. pET-28a(+) was used as the cloning and expression vector for L-threonine aldolase (TA) from *Thermotoga maritima* (*Tm*TA), including *Tm*TA W86Q E88A, *Tm*TA W86Q E88A F85M and other mutants of pyridoxal

phosphate (PLP)-dependent enzymes described in this study.[1-2] Genes of PLP-dependent enzymes were codon optimized and purchased from GeneralBiol as plasmids using pET-28a(+) as the cloning vector. The gene of interest (GOI) was cloned into pET-28a(+) between restriction sites *Nde*I and *Hind*III to include a 6X His tag and a thrombin site at the N terminus. Site-directed mutagenesis was carried out using the overlap extension polymerase chain reaction (PCR). Site-saturation mutagenesis was performed using the "22c-trick" method as described by Kille et al[3]. The PCR products were DpnI digested, gel purified, and ligated using the in-house prepared Gibson mix[4]. This Gibson mix was prepared using 5X isothermal (ISO) reaction buffer (25% PEG-8000, 500 mM Tris-HCl pH 7.5, 50 mM $MgCl_2$, 50 mM DTT, 1 mM each of the dNTPs, and 5 mM NAD), T5 exonuclease, Phusion DNA polymerase, and *Taq* DNA ligase. The ligation mixture was used directly to transform electrocompetent *E. coli* strain *E. cloni* BL21(DE3) cells (Lucigen).

Expression and purification of *Thermotoga maritima* threonine aldolase (*Tm*TA): A single colony from LB_{kan} agar plate was picked using a sterile toothpick and cultured in LB_{kan} media (25 mL) at 37 °C and 230 rpm overnight. This preculture was used to inoculate 1 L of TB_{kan} media (v/v = 2.5%) in a 4 L Erlenmeyer flask, and the expression culture was incubated at 37 °C and 200 rpm for ca. 3 h until the OD_{600} reached ca. 2.0. The culture was cooled on ice for 30 min and induced with 0.5 mM isopropyl-*β*-D-thiogalatopyranoside (IPTG) (final concentration). Protein expression was conducted at 22 °C and 180 rpm for 20 h. *E. coli* cells were harvested by centrifugation at 4 °C and 6,000 g for 10 min using a Thermo Scientific Sorvall Lynx 6000 superspeed centrifuge. The cell pellet was then flash frozen with liquid nitrogen and stored until further use in a –80 °C freezer or directly used for cell lysis and protein purification.

For protein purification, frozen cells were suspended in HisTrap buffer A (25 mM KPi, 100 mM NaCl, 20 mM imidazole, pH = 8.0, ca. 2–3 mL buffer A was added to 1 g of wet cell pellet), followed by the addition of a PLP stock solution (10 mM in 50 mM KPi buffer, pH = 8.0) to a final PLP concentration of 0.2 mM. The cell suspension was transferred to a 120 mL stainless steel beaker and lysed by sonication using a Qsonica Q500 sonicator equipped with a standard probe (tip diameter = ½''). Cell lysis conditions: 45% amplitude, 2 secs on, 4 secs off, 36 min in total (sonication time: 12 min). To pellet the cell debris, lysates were centrifuged using a Lynx 6000 superspeed centrifuge (15,000 g, 60 min, 4 °C). Threonine aldolases containing an N-terminal

6×His tag were purified with a Ni-NTA column (5 mL HisTrap HP, GE Healthcare, Piscataway, NJ) using an AKTA Start protein purification system (GE healthcare). Proteins were eluted on a linear gradient from Histrap buffer A (25 mM KPi, 100 mM NaCl, 20 mM imidazole, pH 8.0) to Histrap buffer B (25 mM KPi, 100 mM NaCl, 500 mM imidazole, pH 8.0) over 10 column volumes (CVs). Proteins eluted at an imidazole concentration of ca. 200 mM. Fractions containing the threonine aldolase variant were combined, followed by the addition of a PLP stock solution (10 mM PLP in 50 mM KPi buffer, pH 8.0) to a final PLP concentration of 0.1 mM. The enzyme solution was concentrated and subjected to three rounds of buffer exchange using the storage buffer (buffer C, 100 mM Ches, pH = 8.0) using an ultracentrifugal filter (10 kDa molecular weight cutoff, Amicon Ultra, Sigma Millipore) to remove excess salt and imidazole. Protein concentration was measured with the Nanodrop A280 method and the bicinchoninic acid (BCA) assay and normalized to ca. 30 mg/mL for storage. Typically, 1 L of TB_{kan} expression culture provides ca. 60 mg *Tm*TA variant. Concentrated proteins were aliquoted and flash-frozen in liquid N_2 and stored at –80 °C until further use. 10% glycerol (final concentration) was added to the final storage buffer to enhance long-term protein stability upon storage.

PLP concentration determination. The concentration of holo PLP enzymes was also determined by PLP-specific UV-visible assays. 75 µL 0.2 M NaOH solution was added into asolution of purified PLP enzyme (5 µL) and carefully mixed. The solution was transformed into a quartz cuvette for UV-vis spectroscopic analysis. PLP concentration was determined using the D equation: $\varepsilon^{PLP} = 6600\ M^{-1}cm^{-1}$ at 390 nm. For PLP enzymes prepared using the procedure described above, the PLP cofactor loading (holo enzyme content) was (100 ± 10)%.

Expression of *Tm*TA variants in 96-well plates. Single colonies from LB_{kan} agar plates were picked using sterile toothpicks and cultured in deep-well 96-well plates containing LB_{kan} (400 µL/well) at 37 °C and 250 rpm shaking (12–14 h) in an Eppendorf Innova 44R shaker. TB_{kan} (950 µL/well) in a deep-well 96-well plate was then inoculated with an aliquot (50 µL/well) of these overnight cultures and allowed to shake for 3.0 h at 37 °C and 250 rpm. The plates were cooled on ice for 20 min and the cultures were induced with 0.5 mM isopropyl β-D-1-thiogalactopyranoside (IPTG). Expression was conducted at 22 ºC, 220 rpm for 20 h.

Reaction screening in 96-well plate format. *E. coli* (*E. cloni* BL21(DE3)) cells in deep-well 96-well plates were pelleted (3,000 *g*, 3 min, 4 °C) using an Eppendorf tabletop centrifuge 5910R and resuspended in Ches buffer (400 µL/well) by gentle shaking using a Fisher Scientific microplate shaker (800 rpm, 3–5 min). Then a PLP stock solution (15 µL per well, 10 mM in 100 mM Ches buffer, pH = 7.5) was added. The cell suspension was lysed by sonication using a Qsonica Q500 sonicator equipped with a 24-tip horn (Figure S1). In each round of sonication, cell suspensions in a total of 24 wells in a 96-well plate were lysed. For example, in first round of sonication, wells A1, A3, A5, …, A11; C1, C3, C5, …, C11; E1, E3, E5, …, E11; G1, G3, G5, …, and G11 were sonicated. To lyse the cell suspensions in the 96-well plate, four iterations of sonication using this 24-tip horn were required. Cell lysis conditions: 45% amplitude, 2 secs on, 4 secs off, 3 min in total (sonication time: 1 min). To pellet the cell debris, lysates were centrifuged using an Eppendorf 5910R tabletop centrifuge (4000 *g*, 20 min, 4 °C). Using an Eppendorf Xplorer 12-channel electronic pipette (50–1000 µL), the clarified cell-free lysates (400 µL per well) were then added to shell vials (400 µL) in a Paradox 96-position reactor from Analytical Sales (Lumidox® II 96-Position Discovery LED Array 445nm-Indigo) and transferred into a Coy anaerobic chamber. In the Coy chamber, 15 µL amino acid substrate (e.g., D-alanine) (0.5 M in degassed 100 mM Ches buffer, pH = 8.0) and 15 µL of the oganoboron substrate (100 mM in degassed DMSO) were added into each well using an Eppendorf Xplorer 12-channel pipette (final concentration of the oganoboron substrate was 3.0 mM). The 96-position Paradox parallel reactor was illuminated with a 445 nm Lumidox 96-position LED array from Analytical Sales (75 mW) and shaken on a Corning digital microplate shaker at room temperature and 380 rpm (Figure S2). After 6 h, the 96-well plate was taken out of the anaerobic chamber and the analytical scale reactions were worked up following the appropriate method below.[1-2]

Activity screening and enantioselectivity screening using LC-MS. After 6 h, a solution of 3.0 mM 2-phenylbutanoic acid (internal standard) in a mixed solvent system (1 M, HCl/MeCN = 1:1, 500 µL) was added using an Eppendorf Xplorer 12-channel, 50–1000 µL electronic pipette. The reaction mixture was pipetted up and down using the Eppendorf 12-channel pipette several times to ensure complete mixing. This mixture (1000 µL per well) was then transferred to a new deep-well 96-well plate and spun down using an Eppendorf 5910R tabletop centrifuge (4500 *g*, 20 min) to completely precipitate the solid from the liquid layer. The supernatant (200 µL per well) was

transferred to 500 μL vial inserts using an Eppendorf Xplorer, 12-channel, 15–300 μL electronic pipette. The inserts were then placed in 1.5 mL vials and analyzed by reverse-phase LC-MS analysis. A representative LC-MS method is as follows: Agilent Poroshell 120, EC-C18 column (4.6 × 50 mm, 4 μm), flow rate = 0.70 mL/min, hold at 5% CH_3CN (0.1% formic acid) in ddH_2O (0.1% formic acid) for 3.0 min, ramp up to 95% CH_3CN (0.1% formic acid) in ddH_2O (0.1% formic acid) over 1.0 min, hold at 95% CH_3CN (0.1% formic acid) in H_2O (0.1% formic acid) for 2.0 min.

II. General experimental and analytical procedures

General procedure A. The threonine aldolase variant *Tm*TA W86Q E88A F85M (*Tm*TA^{QAM} ca. 30 mg/mL, 40 μL) was allowed to thaw and kept on top of ice. In a Coy anaerobic chamber, the following stock solutions were prepared: Organoboron substrate (100 mM in degassed DMSO), amino acid substrate (500 mM in degassed 100 mM Ches buffer, pH = 8.0), PLP (15 mM in 100 mM Ches buffer, pH = 7.5), $Ru(bpy)_3Cl_2$ (9.0 mM in degassed DMSO) and $Co(NH_3)_6Cl_3$ (200 mM in degassed water). To a Fisher brand reaction tube (13 × 100 mm, 8 mL) containing a stir bar were added Ches buffer (720 μL, 100 mM, pH = 9.0), 60 μL organoboron stock solution, 60 μL amino acid substrate stock solution, 40 μL PLP stock solution, 20 μL $Ru(bpy)_3Cl_2$ stock solution, 60 μL $Co(NH_3)_6Cl_3$ stock solution and ca. 40 μL *Tm*TA W86Q E88A F85M solution (the volume of the enzyme stock solution is dependent on the concentration of the specific enzyme sample). The total reaction volume was 1.0 mL; the final concentrations of each reaction component were as follows: 6.0 mM organoboron substrate, 30.0 mM amino acid substrate, 600 μM PLP, 180 μM $Ru(bpy)_3Cl_2$, 12.0 mM $Co(NH_3)_6Cl_3$, and 30 μM *Tm*TA W86Q E88A F85M. The reaction tube was then sealed and removed from the Coy anaerobic chamber and submerged in a water bath at 50 °C. The reaction mixture was allowed to stir at 500 rpm and 50 °C and illuminated with two 45 W Kessil LED lamps (440 nm, 50% intensity) for 12 h.

Yield determination. Upon completion, the reaction mixture was quenched with 1 mL of CH_3CN/1 M aq. HCl (v:v = 1:1). 30 μL of a stock solution of 2-phenylbutyric acid (100 mM in CH_3CN/1 M aq. HCl) was then added as the internal standard. After vigorous mixing, ca. 2.0 mL of reaction mixture was transferred into a 2.0 mL Eppendorf tube and centrifuged at 15,000 rpm for 15 min. The supernatant was analyzed by LC-MS. The yield was determined by LC-MS using

2-phenylbutyric acid as the internal standard using a freshly determined calibration curve. The remaining reaction mixture was then used for the determination of enantiomeric ratio by Marfey's analysis for α-trisubstituted ncAAs, and Fmoc chloride derivatization was applied to α,α-disubstituted α-amino acid products as detailed below. A representative LC-MS analysis method is as follows: Agilent Poroshell 120, EC-C18 column (4.6 × 50 mm, 4 μm), flow rate = 0.70 mL/min, hold at 5% CH_3CN (0.1% formic acid) in H_2O (0.1% formic acid) for 12.0 min, ramp up to 95% CH_3CN (0.1% formic acid) in H_2O (0.1% formic acid) over 1.0 min, hold at 95% CH_3CN (0.1% formic acid) in H_2O (0.1% formic acid) for 2.0 min.

Determination of absolute stereochemistry. The absolute stereochemistry of α,α-disubstituted α-amino acids product (2*S*,3*S*)-2-amino-2-methyl-3-phenylbutanoic acid **3a** was determined by comparison with previous reported stereochemistry[5-7] (*vide infra*). We measured the optical rotation of the biocatalytically produced product (major diastereomer) and obtained a negative value ($[\alpha]^{20}_D$ = –20.2 (*c* = 0.43, MeOH)). Based on literature precedents, we assign this product to either (2*S*,3*S*)-**3a** or (2*R*,3*S*)-**3a**. To further identify which diastereomer was produced, we conducted comparative ^{1}H NMR analysis. The major product showed a characteristic signal at 3.07 ppm while minor product at 2.98 ppm. Prior literature results revealed that (2*S*,3*S*)-**3a** has a signal at 3.07 pm.

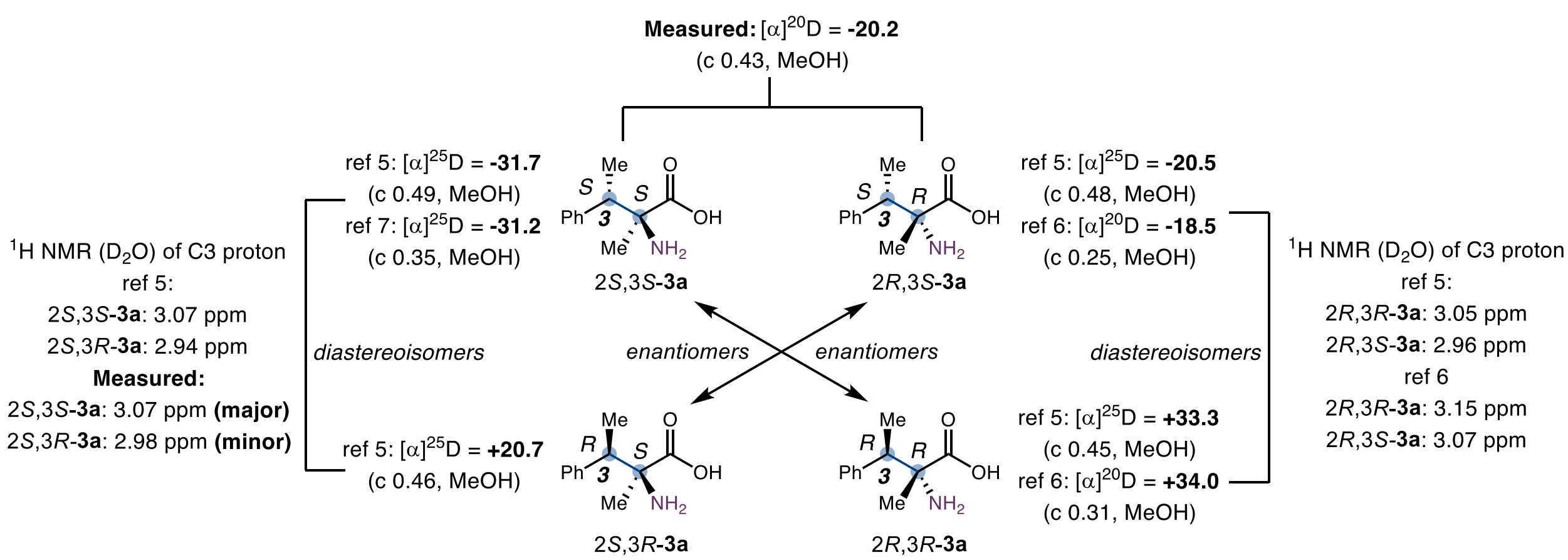

Comparison of Spectroscopic data

	3a·HCl (major)	Lit[5]	Lit[6]
^{1}H NMR	7.27-6.61 (m, 5H), 3.07 (q, *J* = 7.3 Hz, 1H), 1.16 (s, 3H), 1.06 (d, *J* = 7.4Hz, 3H).	7.09 (m, 5H), 3.07 (q, 1H, *J* = 7.4Hz), 1.14 (s, 3H),1.11 (d, *J* = 7.4Hz, 3H);	7.18 (m, 5H), 3.15 (q, 1H, *J* = 7.5 Hz), 1.24 (s, 3H), 1.21 (d, 3H, *J* = 7.4 Hz).
$[\alpha]_D^{20}$	–20.2 (*c* = 0.43, MeOH)	–31.7 (*c* = 0.49, MeOH)	
	**3a·HCl (minor)**	**Lit[5]**	**Lit[6]**
^{1}H NMR	7.27-6.61 (m, 5H), 2.98 (q, *J* = 7.2 Hz, 1H), 1.14 (s, 3H), 1.03 (d, *J* = 7.4Hz, 3H).	7.08(m, 5H), 2.94 (q, 1H, *J* = 7.3Hz), 1.03 (d, 3H, *J* = 7.3Hz), 0.96 (s, 3H);	7.17 (m, 5H), 3.07 (q, 1H, *J* = 7.0 Hz), 1.19 (s, 3H), 1.18 (d, 3H, *J* = 7.5 Hz)

The absolute stereochemistry of other non-canonical amino acid products (**3b**-**3p**) was inferred by analogy to **3a**. The absolute stereochemistry of phenylalanine derivatives was determined from commercially available **L-5a**. The absolute stereochemistry of quaternary stereocenter reaction product (*S*)-2-amino-2-methyl-3-phenylpropanoic acid **6a** was determined by comparison with literature[8] (*vide infra*). The absolute stereochemistry of other non-canonical amino acid products (**6b**-**6c**) was inferred by analogy to **6a**.

Determination of enantiomeric ratio (e.r.) of α,α-disubstituted α-amino acids using Fmoc chloride derivatization and normal phase chiral HPLC analysis. Marfey's analysis was found to be ineffective in the transformation of α-tetrasubstituted ncAAs and low conversions were usually observed despite our attempts to optimize this process. We thus applied Fmoc chloride derivatization to these α,α-disubstituted α-amino acid products and resolved the enantiomers of these derivatization products by chiral normal phase HPLC. Ten analytical scale reactions were combined and purified to accurately determine the enantiomeric ratio of the product.

Experimental procedure. To a one-dram vial containing a stir bar were added the respective α-tetrasubstituted amino acid (50 µmol), 300 mM aq. NaOH (3 equiv, 500 µL) and a stock solution of fluorenylmethyloxycarbonyl chloride (Fmoc–Cl, 1.2 equiv, 600 µL, 100 mM in acetonitrile). The reaction mixture was allowed to stir at room temperature for 12 h. The organic solvent was removed *in vacuo* and the aqueous layer was washed with Et_2O (3 mL). The aqueous layer was then acidified with saturated aqueous citric acid solution (2.0 mL, final pH = 2–3) and extracted with EtOAc (3 × 3 mL). The combined organic layer was dried over Na_2SO_4, filtered, and

evaporated *in vacuo* with the aid of a rotary evaporator. The residue was subjected to flash column chromatography on silica gel to furnish the desired derivatized compound as a white solid, which was dissolved in 1:1 *i*-PrOH/hexanes (1.0 mL) and analyzed by chiral HPLC.

A representative chiral HPLC analysis method is as follows: CHIRALCEL IC column (4.6 mm × 25 cm, 5 micron), 95:5 hexanes: EtOH (0.1% trifluoroacetic acid (TFA)), flow rate = 1.0 mL/min, monitoring wavelength = 265 nm, 40 °C, 30 min.

General procedure for the preparation of non-canonical amino acids on a 1 mmol scale

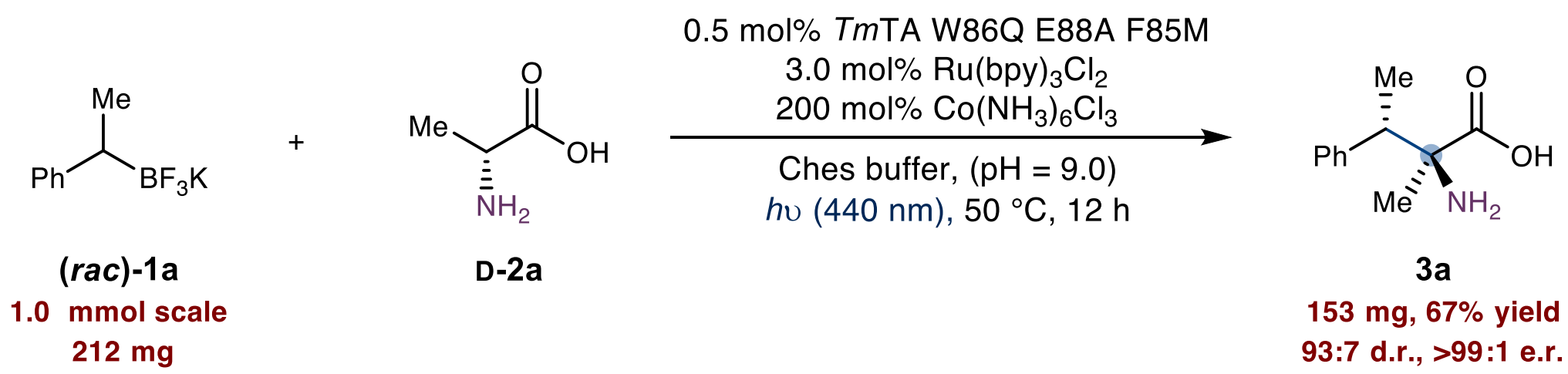

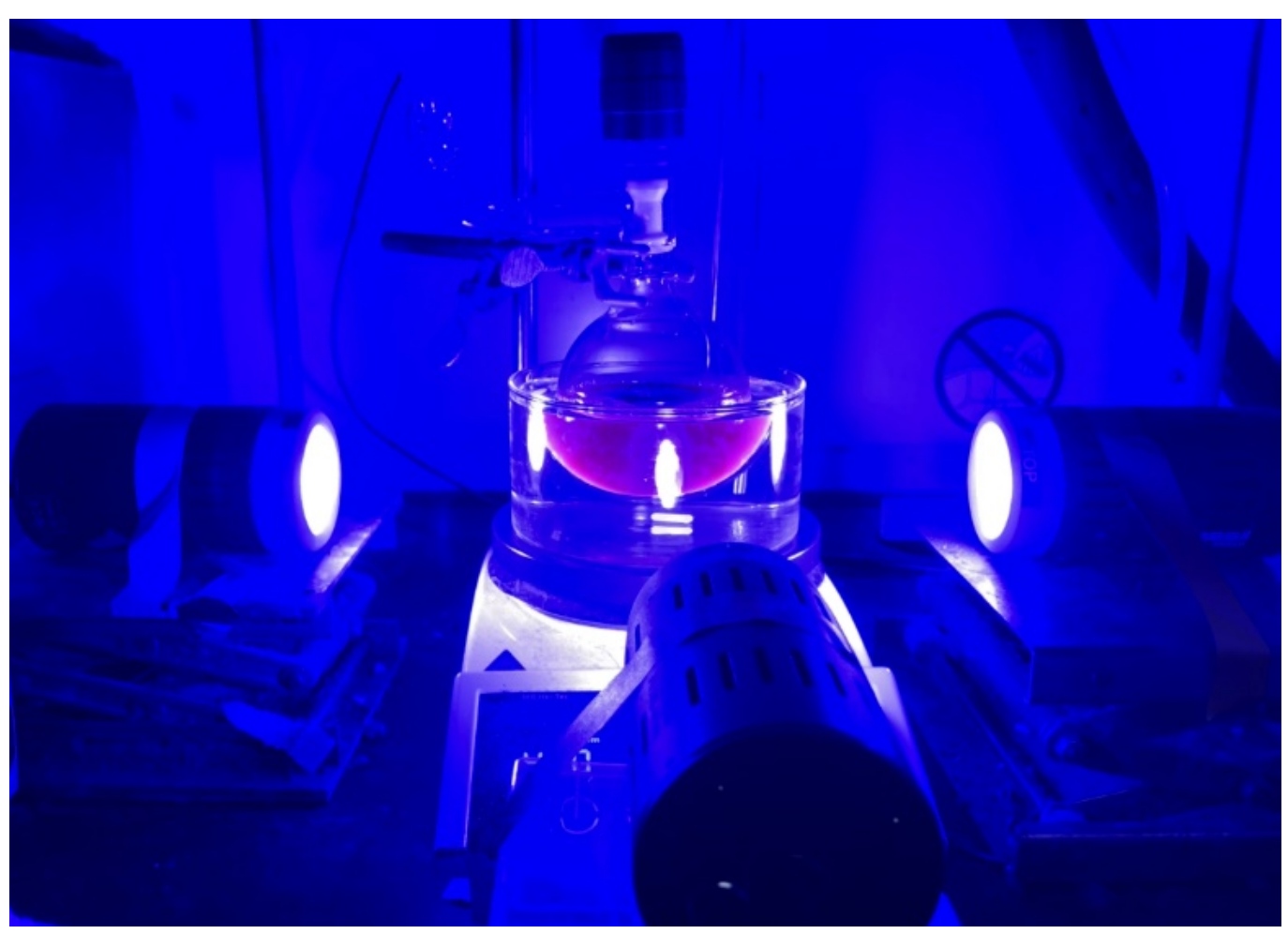

Figure. S1. 1.0 mmol scale photobiocatalytic synthesis of α,α-disubstituted-α-amino acid **3a**.

General Procedure. *Tm*TA^{QAM} (*Tm*TA W86Q E88A F85M, ca. 30 mg/mL, 1 mL/tube) was thawed from –80 °C freezer and kept on ice. In a Coy anaerobic chamber, the following stock solutions were prepared: organoboron substrate (200 mM in degassed DMSO), $Ru(bpy)_3Cl_2$ (20.0 mM in degassed DMSO), D-alanine (500 mM in degassed 100 mM Ches buffer), PLP (50 mM in 100 mM Ches buffer, pH = 7.5), $Co(NH_3)_6Cl_3$ (200 mM in degassed water). To a 250 mL round

bottom flask equipped with a stir bar were added 100 mM Ches buffer (80 mL, pH = 9.0), 5 mL organoboron substrate stock solution, 20 mL D-alanine stock solution, 2 mL PLP stock solution, 10 mL $Co(NH_3)_6Cl_3$ stock solution, 1.5 mL $Ru(bpy)_3Cl_2$ stock solution, and ca. 6.0 mL *Tm*TA^{QAM} enzyme solution (30 mg/mL). The total reaction volume was 125.0 mL; the final concentrations of each reaction component were as follows: 8.0 mM organoboron substrate, 80.0 mM D-alanine, 16.0 mM $Co(NH_3)_6Cl_3$, 0.8 mM PLP, 40 μM *Tm*TA^{QAM} and 0.24 mM $Ru(bpy)_3Cl_2$. The reaction tube was then sealed and removed from the Coy anaerobic chamber and submerged in a water bath preheated to 50 °C. The reaction mixture was allowed to stir at 500 rpm and 50 °C and illuminated with three Kessil LED lamps (440 nm, 45 W, 50% intensity) for 12 h. The reaction mixture was then quenched with 1 M HCl (100 mL) and MeCN (100 mL) and centrifuged (4 ˚C, 30 min, 4500 rpm). The supernatant was concentrated *in vacuo* with the aid of a rotary evaporator until the total volume was less than 50 mL. The residue was centrifuged (30 min, 4500 rpm) again to separate the insoluble fraction. The supernatant was further concentrated *in vacuo* with the aid of a rotary evaporator to close to dryness. Approximately 40 mL of water was added to dissolve the residue. The resulting mixture was filtered through a 4.5 μm membrane filter and loaded onto a C18 column (12 g catridge) that had been equilibrated to water with 0.1% HCOOH. The column was first washed with 10 column volumes (CV) of water (0.1% HCOOH) to remove excessive salts. Then, the product was eluted with a gradient of 0% to 20% MeCN in water over 12 CVs. Product-containing fractions were combined and concentrated *in vacuo* to remove all the organic solvents. The residual aqueous solution was frozen in liquid nitrogen and lyophilized using a Christ Alpha 1-2 LDplus lyophilizer to give the amino acid product. Product **3a** was isolated as an HCl adduct as a white solid (153 mg, 67% yield).

III. Selected further results for reaction development

Table S1. Evaluation of photoredox catalysts for diastereo- and enantioselective synthesis of **3a**[a]

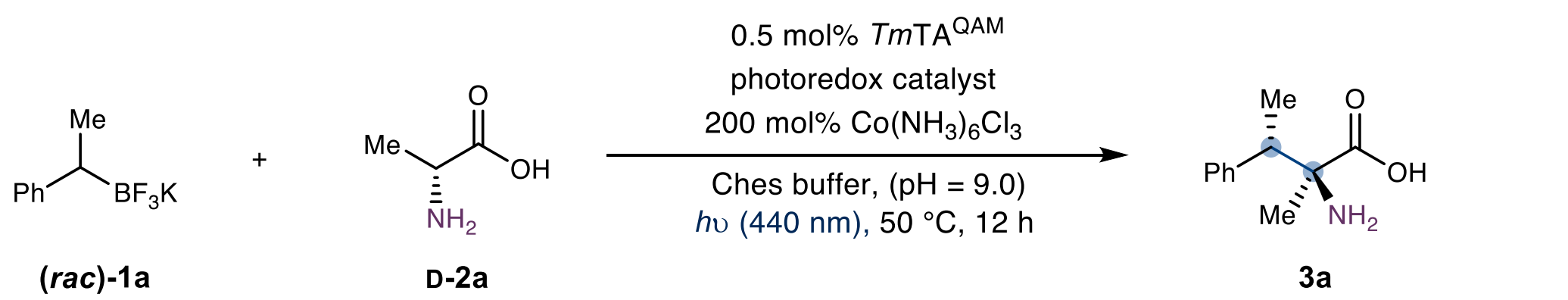

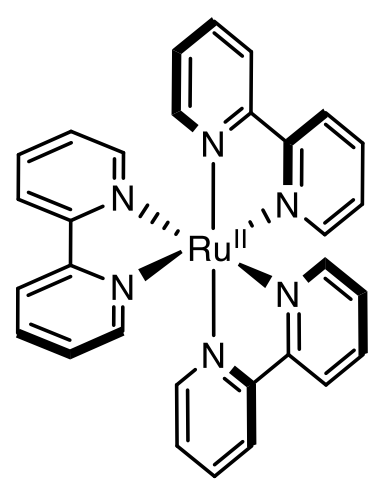

$[Ru(bpy)_3]^{2+}$

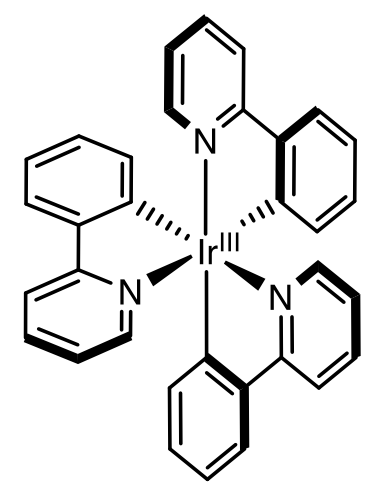

(*fac*)-$Ir(ppy)_3$

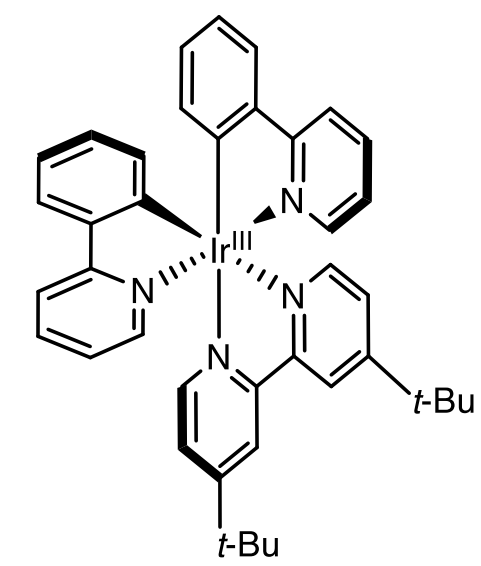

$Ir(ppy)_2dtbpy$

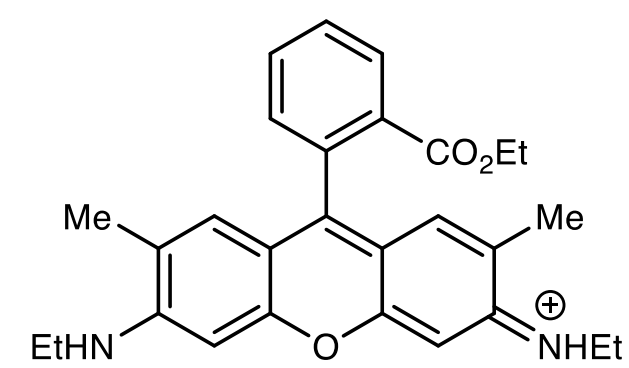

Rh 6G (rhodamine 6G)

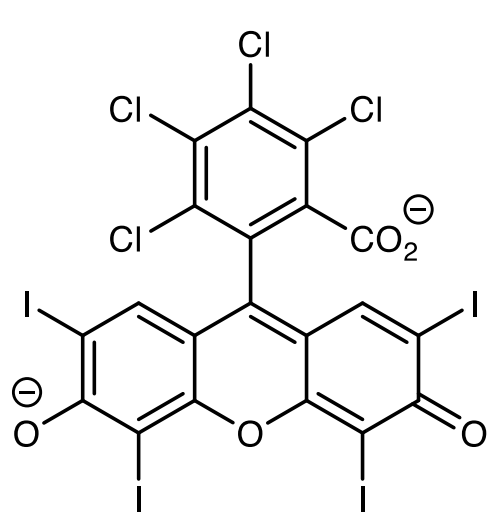

Rose bengal

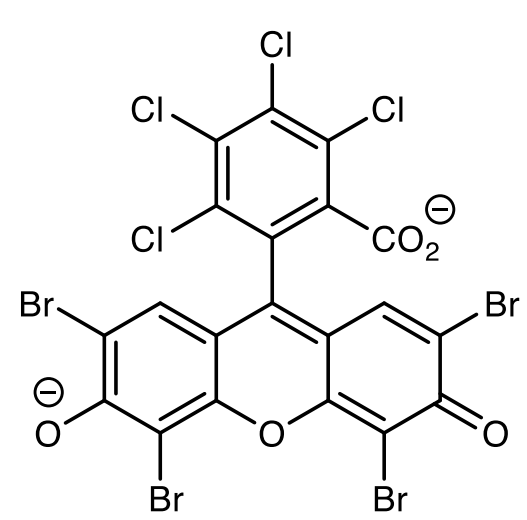

Phloxime B

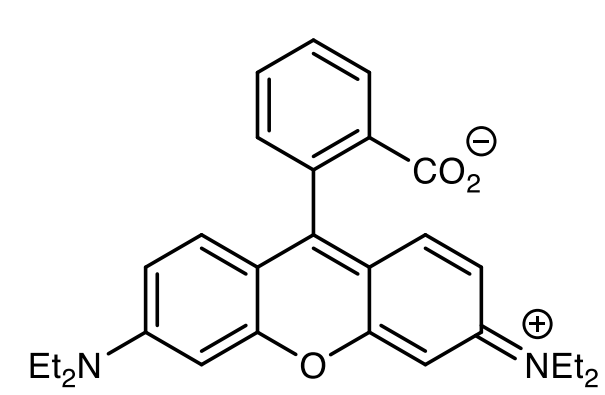

Rh B (rhodamine B)

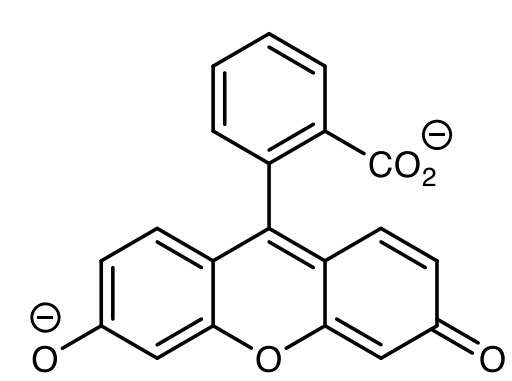

Fluorescein

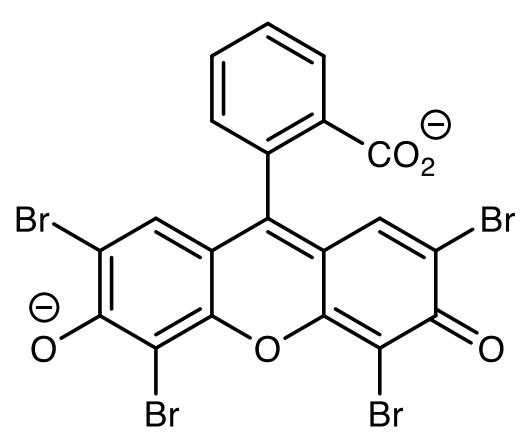

Eosin Y

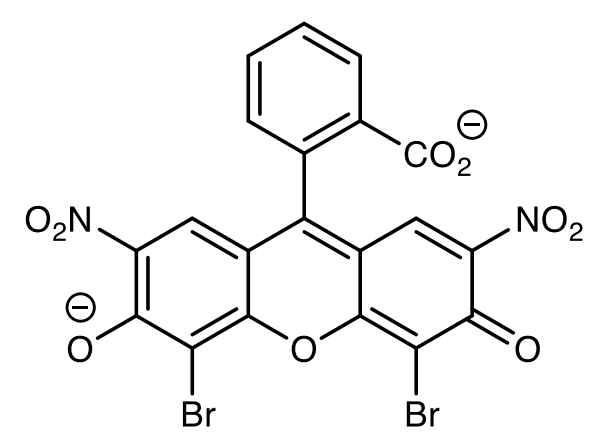

Eosin B

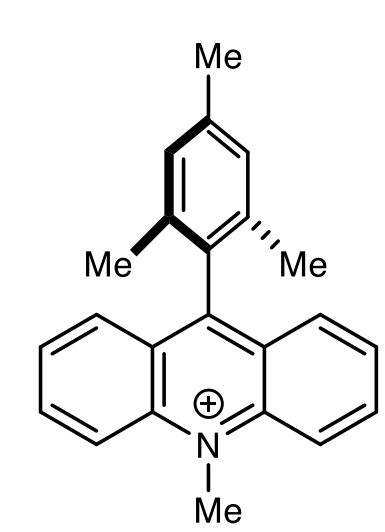

9-mesityl-10-methylacridinium

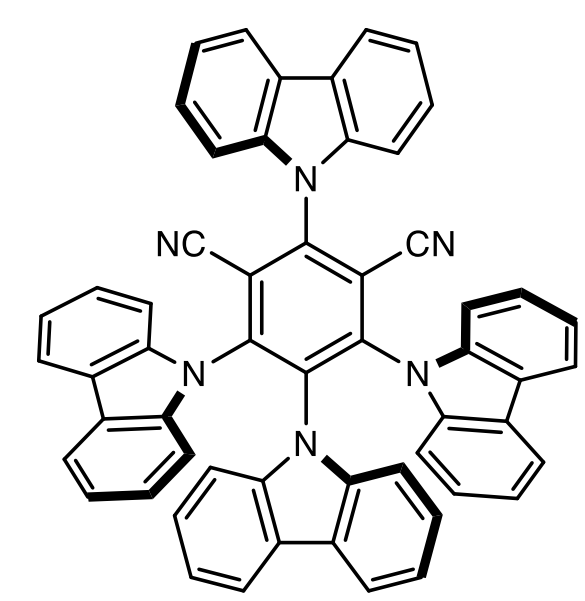

4CzIPN

entry	photoredox catalyst	yield of **3a**	d.r. of **3a**	e.r. of **3a**
1	4CzIPN (10 mol%)	70%	94:6	>99:1
2	Eosin Y (10 mol%)	63%	95:5	>99:1
3	9-mesityl-10-methylacridinium (10 mol%)	56%	94:6	>99:1
4	Rhodamine B (10 mol%)	68%	91:9	>99:1
5	Phloxime B (10 mol%)	60%	93:7	>99:1
6	Rhodamine 6G (10 mol%)	62%	94:6	>99:1
7	Rose bengal (10 mol%)	51%	95:5	>99:1
8	Fluorescein (10 mol%)	64%	93:7	>99:1
9	Eosin B (10 mol%)	58%	94:6	>99:1
10	$Ru(bpy)_3Cl_2$ (2 mol%)	73%	94:6	>99:1
11	*fac*-$Ir(ppy)_3$ (2 mol%)	50%	93:7	>99:1
12	$Ir(ppy)_2(dtbpy)PF_6$ (2 mol%)	23%	93:7	>99:1

[a]**(*rac*)-1a** (6.0 mM, 1.0 equiv), **2a** (30 mM, 5.0 equiv), 0.50 mol% *Tm*TA W86Q E88A F85M (*Tm*TA^{QAM}) (30 μM), 2–10 mol% photocatalyst (120–600 μM), 200 mol% $Co(NH_3)_6Cl_3$ (12.0 mM), PLP (0.60 mM), *hν* (440 nm, 45 W, 50% intensity), 100 mM Ches buffer (pH = 9.0), DMSO (8% v/v), 50 °C, 12 h.

Table S2. Buffer evaluation for biocatalytic diastereo- and enantioselective synthesis of **3a**[a]

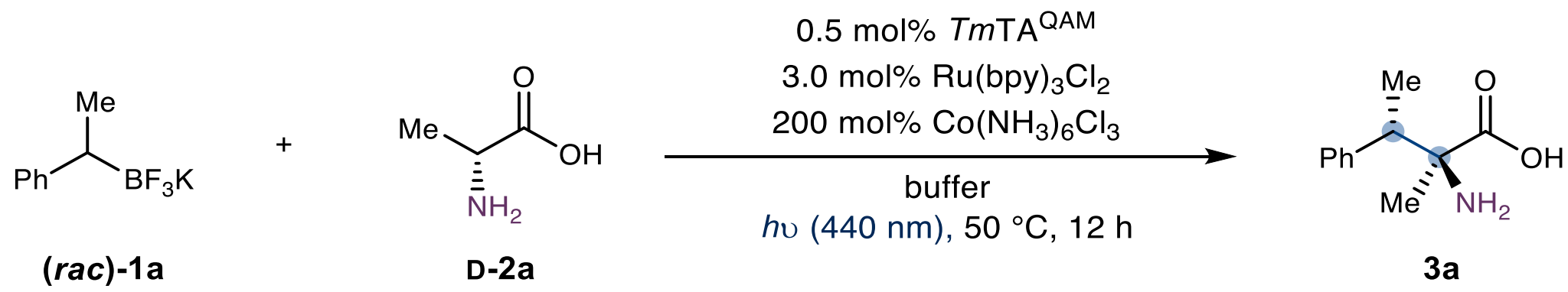

entry	Buffer	yield of **3a**	d.r. of **3a**	e.r. of **3a**
1	Ches (100 mM, pH 7.0)	29%	92:8	>99:1
2	Ches (100 mM, pH 7.5)	41%	93:7	>99:1
3	Ches (100 mM, pH 8.0)	52%	93:7	>99:1
4	Ches (100 mM, pH 8.5)	68%	94:6	>99:1
5	Ches (100 mM, pH 9.0)	75%	94:6	>99:1
6	Ches (100 mM, pH 9.5)	74%	94:6	>99:1
7	KPi (200 mM, pH 9.0)	63%	94:6	>99:1
8	Tris (100 mM, pH 9.0)	66%	94:6	>99:1
9	HEPES (100 mM, pH 9.0)	61%	93:7	>99:1

[a]**(*rac*)-1a** (6.0 mM), **2a** (30 mM), 0.5 mol% *Tm*TA^{QAM} (30 μM), 3.0 mol% $Ru(bpy)_3Cl_2$ (0.18 mM), 200 mol% $Co(NH_3)_6Cl_3$ (12.0 mM), PLP (0.60 mM), $h\nu$ (440 nm, 45 W, 50% intensity), buffer, DMSO (8% v/v), 50 °C, 12 h.

Table S3. *Tm*TA variant evaluation for diastereo- and enantioselective synthesis of **3a**[a]

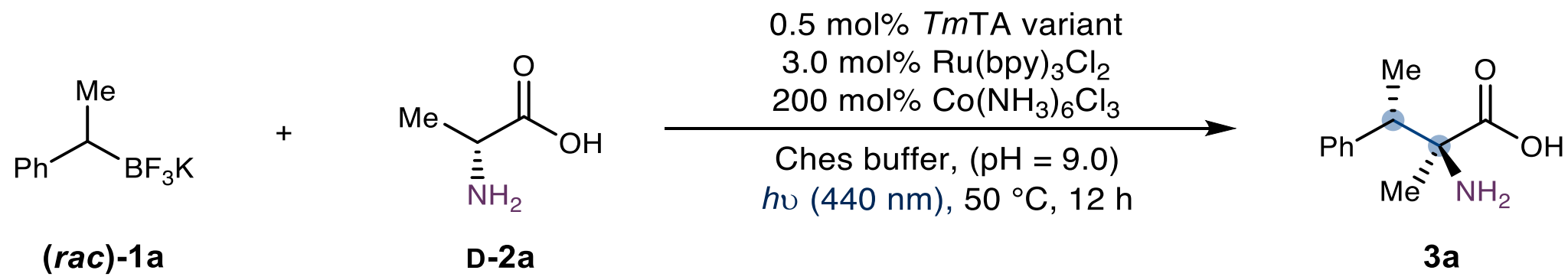

entry	*Tm*TA variant	yield of **3a**	d.r. of **3a**	e.r. of **3a**
1	wild type	41%	88:12	>99:1
2	*Tm*TA W86N	44%	89:11	>99:1
3	*Tm*TA W86E	46%	90:10	>99:1
4	*Tm*TA W86Q	46%	90:10	>99:1
5	*Tm*TA E88T	59%	89:11	>99:1
6	*Tm*TA H83F	47%	88:12	>99:1
7	*Tm*TA E88T H83F	58%	92:8	>99:1
8	*Tm*TA W86Q E88T	58%	92:8	>99:1
9	*Tm*TA W86Q E88A	73%	93:7	>99:1
10	*Tm*TA W86Q E88A F85M	75%	94:6	>99:1
11	*Tm*TA W86Q E88A	49%[b]	92:8	>99:1
12	*Tm*TA W86Q E88A F85M	53%[b]	94:6	>99:1

[a]**(*rac*)-1a** (6.0 mM), **2a** (30 mM), 0.5 mol% *Tm*TA (30 μM), 3.0 mol% $Ru(bpy)_3Cl_2$ (0.18 mM), 200 mol% $Co(NH_3)_6Cl_3$ (12.0 mM), PLP (0.60 mM), $h\nu$ (440 nm, 45 W, 50% intensity), Ches buffer, pH = 9.0, DMSO (8% v/v), 50 °C, 12 h. [b]0.1 mol% *Tm*TA (6 μM) was used.

Table S4. Further studies on biocatalytic diastereo- and enantioselective synthesis of **3a**[a]

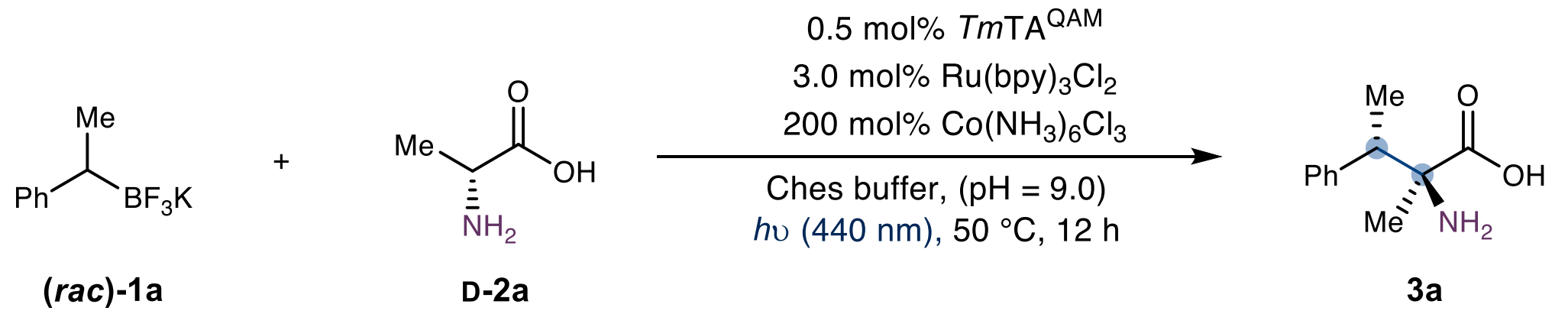

entry	variation from the standard conditions	yield of **3a** (%)	TTN	d.r. of **3a**	e.r. of **3a**
1	None	75%	150	94:6 (93:7)[b]	>99:1
2	5% PLP	56%	112	94:6	>99:1
3	w/o PLP	43%	86	94:6	>99:1
4	w/o enzyme	0%	-	-	>99:1
5	w/o *hν*	0%	-	-	>99:1
6	w/o $Co(NH_3)_6Cl_3$	15%	30	94:6	>99:1
7	w/o $Ru(bpy)_3Cl_2$	13%	26	89:11	>99:1
8	0.2 mol% *Tm*TA^{QAM}	62%	310	94:6	>99:1
9	0.1 mol% *Tm*TA^{QAM}	53%	530	94:6	>99:1
10	10 equiv (*rac*)-**2a**	54%	108	94:6	>99:1
11	10 equiv D-**2a**	78%	156	94:6	>99:1
12	10 equiv L-**2a**	55%	110	93:7	>99:1
13	*hν* (390 nm)	65%	130	94:6	>99:1
14	*hν* (467 nm)	68%	128	94:6	>99:1
15	*hν* (525 nm)	53%	106	94:6	>99:1

[a]**(*rac*)-1a** (6.0 mM), **2a** (30 mM), 0.5 mol% *Tm*TA^{QAM} (30 μM), 3.0 mol% $Ru(bpy)_3Cl_2$ (0.18 mM), 200 mol% $Co(NH_3)_6Cl_3$ (12.0 mM), PLP (0.60 mM), *hν* (440 nm, 45 W, 50% intensity), Ches buffer, pH = 9.0, DMSO (8% v/v), 50 °C, 12 h. [b]Diastereoisomeric ratio determined by ^1H NMR analysis of unpurified reaction mixture.

Table S5. Turnover efficiency of biocatalytic enantioselective synthesis of **6a**[a]

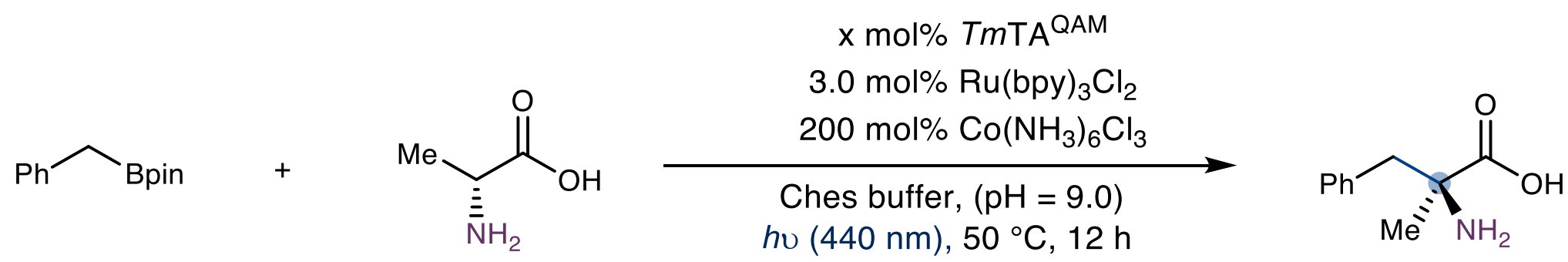

1a **D-2a** **6a**

entry	enzyme loading	yield of **6a** (%)	TON	e.r. of **6a**
1	1.0 mol%	87%	87	>99:1
2	0.5 mol%	81%	162	>99:1
3	0.2 mol%	74%	370	>99:1
4	0.1 mol%	68%	680	>99:1
5	0.05 mol%	46%	920	>99:1
6	0.05 mol%	43%[b]	860	>99:1
7	0.02 mol%	28%	1400	>99:1
8	0.01 mol%	13%	1300	>99:1

[a]**1a** (6.0 mM), **2a** (60 mM), x mol% *Tm*TA^{QAM}, 3.0 mol% $Ru(bpy)_3Cl_2$ (0.18 mM), 200 mol% $Co(NH_3)_6Cl_3$ (12.0 mM), PLP (0.60 mM), *hν* (440 nm, 45 W, 50% intensity), Ches buffer, pH = 9.0, DMSO (8% v/v), 50 °C, 12 h. [b]2.0 mol% $Ru(bpy)_3Cl_2$ was used.

IV. DNA and protein sequences

Wild-type *Tm*TA

DNA sequence

ATGGGCAGCAGCCATCATCATCATCATCACAGCAGCGGCCTGGTGCCGCGCGGCAGCCATATGA
TCGATCTGCGCAGCGATACCGTGACCAAACCGACAGAAGAAATGCGTAAAGCAATGGCTCAGGC
CGAAGTTGGCGATGATGTTTATGGCGAAGATCCGACCATTAATGAACTGGAACGTCTGGCCGCC
GAAACCTTTGGTAAAGAAGCCGCCCTGTTTGTGCCGAGCGGTACAATGGGTAATCAGGTGAGCA
TTATGGCCCATACCCAGCGCGGTGACGAAGTTATTCTGGAAGCAGATAGTCATATTTTCTGGTA
TGAAGTTGGTGCAATGGCTGTTCTGAGCGGTGTGATGCCGCATCCTGTTCCTGGTAAAAATGGT
GCAATGGACCCTGATGATGTTCGTAAAGCAATTCGTCCGCGTAATATTCATTTTCCGCGTACCA
GTCTGATTGCAATTGAAAATACCCATAATCGTAGCGGTGGTCGCGTTGTTCCGCTGGAAAATAT
TAAGGAAATTTGTACCATCGCCAAAGAACATGGTATTAATGTGCATATTGACGGTGCCCGTATT
TTTAATGCAAGCATTGCAAGTGGTGTTCCGGTGAAAGAATATGCCGGTTATGCCGATAGTGTTA
TGTTTTGTCTGAGTAAAGGTCTGTGTGCCCCGGTTGGTAGCGTGGTTGTTGGTGACCGTGATTT
TATTGAACGTGCACGTAAAGCACGTAAAATGCTGGGTGGTGGTATGCGTCAGGCAGGTGTTCTG
GCAGCTGCAGGTATTATTGCACTGACCAAAATGGTGGATCGCCTGAAAGAAGATCATGAAAATG
CACGCTTTCTGGCACTGAAACTGAAAGAAATTGGCTATAGTGTTAACCCGGAAGATGTGAAAAC
CAATATGGTTATTCTGCGTACCGATAATCTGAAAGTTAATGCCCACGGTTTTATTGAAGCACTG
CGCAATAGTGGCGTGCTGGCAAATGCCGTTAGCGATACCGAAATTCGCCTGGTTACCCATAAAG
ATGTTAGTCGTAATGATATTGAGGAAGCCCTGAATATTTTTGAAAAACTGTTTCGTAAGTTCAG
CTAA

Protein sequence

MGSSHHHHHHSSGLVPRGSHMIDLRSDTVTKPTEEMRKAMAQAEVGDDVYGEDPTINELERLAA
ETFGKEAALFVPSGTMGNQVSIMAHTQRGDEVILEADSHIFWYEVGAMAVLSGVMPHPVPGKNG
AMDPDDVRKAIRPRNIHFPRTSLIAIENTHNRSGGRVVPLENIKEICTIAKEHGINVHIDGARI
FNASIASGVPVKEYAGYADSVMFCLSKGLCAPVGSVVVGDRDFIERARKARKMLGGGMRQAGVL

AAAGIIALTKMVDRLKEDHENARFLALKLKEIGYSVNPEDVKTNMVILRTDNLKVNAHGFIEAL
RNSGVLANAVSDTEIRLVTHKDVSRNDIEEALNIFEKLFRKFS

***Tm*TA W86N**

DNA sequence

ATGGGCAGCAGCCATCATCATCATCATCACAGCAGCGGCCTGGTGCCGCGCGGCAGCCATATGA
TCGATCTGCGCAGCGATACCGTGACCAAACCGACAGAAGAAATGCGTAAAGCAATGGCTCAGGC
CGAAGTTGGCGATGATGTTTATGGCGAAGATCCGACCATTAATGAACTGGAACGTCTGGCCGCC
GAAACCTTTGGTAAAGAAGCCGCCCTGTTTGTGCCGAGCGGTACAATGGGTAATCAGGTGAGCA
TTATGGCCCATACCCAGCGCGGTGACGAAGTTATTCTGGAAGCAGATAGTCATATTTTC**AAT**TA
TGAAGTTGGTGCAATGGCTGTTCTGAGCGGTGTGATGCCGCATCCTGTTCCTGGTAAAAATGGT
GCAATGGACCCTGATGATGTTCGTAAAGCAATTCGTCCGCGTAATATTCATTTTCCGCGTACCA
GTCTGATTGCAATTGAAAATACCCATAATCGTAGCGGTGGTCGCGTTGTTCCGCTGGAAAATAT
TAAGGAAATTTGTACCATCGCCAAAGAACATGGTATTAATGTGCATATTGACGGTGCCCGTATT
TTTAATGCAAGCATTGCAAGTGGTGTTCCGGTGAAAGAATATGCCGGTTATGCCGATAGTGTTA
TGTTTTGTCTGAGTAAAGGTCTGTGTGCCCCGGTTGGTAGCGTGGTTGTTGGTGACCGTGATTT
TATTGAACGTGCACGTAAAGCACGTAAAATGCTGGGTGGTGGTATGCGTCAGGCAGGTGTTCTG
GCAGCTGCAGGTATTATTGCACTGACCAAAATGGTGGATCGCCTGAAAGAAGATCATGAAAATG
CACGCTTTCTGGCACTGAAACTGAAAGAAATTGGCTATAGTGTTAACCCGGAAGATGTGAAAAC
CAATATGGTTATTCTGCGTACCGATAATCTGAAAGTTAATGCCCACGGTTTTATTGAAGCACTG
CGCAATAGTGGCGTGCTGGCAAATGCCGTTAGCGATACCGAAATTCGCCTGGTTACCCATAAAG
ATGTTAGTCGTAATGATATTGAGGAAGCCCTGAATATTTTTGAAAAACTGTTTCGTAAGTTCAG
CTAA

Protein sequence

MGSSHHHHHHSSGLVPRGSHMIDLRSDTVTKPTEEMRKAMAQAEVGDDVYGEDPTINELERLAA
ETFGKEAALFVPSGTMGNQVSIMAHTQRGDEVILEADSHIF**N**YEVGAMAVLSGVMPHPVPGKNG
AMDPDDVRKAIRPRNIHFPRTSLIAIENTHNRSGGRVVPLENIKEICTIAKEHGINVHIDGARI
FNASIASGVPVKEYAGYADSVMFCLSKGLCAPVGSVVVGDRDFIERARKARKMLGGGMRQAGVL

AAAGIIALTKMVDRLKEDHENARFLALKLKEIGYSVNPEDVKTNMVILRTDNLKVNAHGFIEAL
RNSGVLANAVSDTEIRLVTHKDVSRNDIEEALNIFEKLFRKFS

*Tm*TA E88T

DNA sequence

ATGGGCAGCAGCCATCATCATCATCATCACAGCAGCGGCCTGGTGCCGCGCGGCAGCCATATGA
TCGATCTGCGCAGCGATACCGTGACCAAACCGACAGAAGAAATGCGTAAAGCAATGGCTCAGGC
CGAAGTTGGCGATGATGTTTATGGCGAAGATCCGACCATTAATGAACTGGAACGTCTGGCCGCC
GAAACCTTTGGTAAAGAAGCCGCCCTGTTTGTGCCGAGCGGTACAATGGGTAATCAGGTGAGCA
TTATGGCCCATACCCAGCGCGGTGACGAAGTTATTCTGGAAGCAGATAGTCATATTTTCTGGTA
T**ACG**GTTGGTGCAATGGCTGTTCTGAGCGGTGTGATGCCGCATCCTGTTCCTGGTAAAAATGGT
GCAATGGACCCTGATGATGTTCGTAAAGCAATTCGTCCGCGTAATATTCATTTTCCGCGTACCA
GTCTGATTGCAATTGAAAATACCCATAATCGTAGCGGTGGTCGCGTTGTTCCGCTGGAAAATAT
TAAGGAAATTTGTACCATCGCCAAAGAACATGGTATTAATGTGCATATTGACGGTGCCCGTATT
TTTAATGCAAGCATTGCAAGTGGTGTTCCGGTGAAAGAATATGCCGGTTATGCCGATAGTGTTA
TGTTTTGTCTGAGTAAAGGTCTGTGTGCCCCGGTTGGTAGCGTGGTTGTTGGTGACCGTGATTT
TATTGAACGTGCACGTAAAGCACGTAAAATGCTGGGTGGTGGTATGCGTCAGGCAGGTGTTCTG
GCAGCTGCAGGTATTATTGCACTGACCAAAATGGTGGATCGCCTGAAAGAAGATCATGAAAATG
CACGCTTTCTGGCACTGAAACTGAAAGAAATTGGCTATAGTGTTAACCCGGAAGATGTGAAAAC
CAATATGGTTATTCTGCGTACCGATAATCTGAAAGTTAATGCCCACGGTTTTATTGAAGCACTG
CGCAATAGTGGCGTGCTGGCAAATGCCGTTAGCGATACCGAAATTCGCCTGGTTACCCATAAAG
ATGTTAGTCGTAATGATATTGAGGAAGCCCTGAATATTTTTGAAAAACTGTTTCGTAAGTTCAG
CTAA

Protein sequence

MGSSHHHHHHSSGLVPRGSHMIDLRSDTVTKPTEEMRKAMAQAEVGDDVYGEDPTINELERLAA
ETFGKEAALFVPSGTMGNQVSIMAHTQRGDEVILEADSHIFWY**T**VGAMAVLSGVMPHPVPGKNG
AMDPDDVRKAIRPRNIHFPRTSLIAIENTHNRSGGRVVPLENIKEICTIAKEHGINVHIDGARI
FNASIASGVPVKEYAGYADSVMFCLSKGLCAPVGSVVVGDRDFIERARKARKMLGGGMRQAGVL

AAAGIIALTKMVDRLKEDHENARFLALKLKEIGYSVNPEDVKTNMVILRTDNLKVNAHGFIEAL
RNSGVLANAVSDTEIRLVTHKDVSRNDIEEALNIFEKLFRKFS

*Tm*TA H83F

DNA sequence

<u>ATGGGCAGCAGCCATCATCATCATCATCACAGCAGCGGCCTGGTGCCGCGCGGCAGCCAT</u>ATGA
TCGATCTGCGCAGCGATACCGTGACCAAACCGACAGAAGAAATGCGTAAAGCAATGGCTCAGGC
CGAAGTTGGCGATGATGTTTATGGCGAAGATCCGACCATTAATGAACTGGAACGTCTGGCCGCC
GAAACCTTTGGTAAAGAAGCCGCCCTGTTTGTGCCGAGCGGTACAATGGGTAATCAGGTGAGCA
TTATGGCCCATACCCAGCGCGGTGACGAAGTTATTCTGGAAGCAGATAGT**TTT**ATTTTCTGGTA
TGAAGTTGGTGCAATGGCTGTTCTGAGCGGTGTGATGCCGCATCCTGTTCCTGGTAAAAATGGT
GCAATGGACCCTGATGATGTTCGTAAAGCAATTCGTCCGCGTAATATTCATTTTCCGCGTACCA
GTCTGATTGCAATTGAAAATACCCATAATCGTAGCGGTGGTCGCGTTGTTCCGCTGGAAAATAT
TAAGGAAATTTGTACCATCGCCAAAGAACATGGTATTAATGTGCATATTGACGGTGCCCGTATT
TTTAATGCAAGCATTGCAAGTGGTGTTCCGGTGAAAGAATATGCCGGTTATGCCGATAGTGTTA
TGTTTTGTCTGAGTAAAGGTCTGTGTGCCCCGGTTGGTAGCGTGGTTGTTGGTGACCGTGATTT
TATTGAACGTGCACGTAAAGCACGTAAAATGCTGGGTGGTGGTATGCGTCAGGCAGGTGTTCTG
GCAGCTGCAGGTATTATTGCACTGACCAAAATGGTGGATCGCCTGAAAGAAGATCATGAAAATG
CACGCTTTCTGGCACTGAAACTGAAAGAAATTGGCTATAGTGTTAACCCGGAAGATGTGAAAAC
CAATATGGTTATTCTGCGTACCGATAATCTGAAAGTTAATGCCCACGGTTTTATTGAAGCACTG
CGCAATAGTGGCGTGCTGGCAAATGCCGTTAGCGATACCGAAATTCGCCTGGTTACCCATAAAG
ATGTTAGTCGTAATGATATTGAGGAAGCCCTGAATATTTTTGAAAAACTGTTTCGTAAGTTCAG
CTAA

Protein sequence

<u>MGSSHHHHHHSSGLVPRGSH</u>MIDLRSDTVTKPTEEMRKAMAQAEVGDDVYGEDPTINELERLAA
ETFGKEAALFVPSGTMGNQVSIMAHTQRGDEVILEADS**F**IFWYEVGAMAVLSGVMPHPVPGKNG
AMDPDDVRKAIRPRNIHFPRTSLIAIENTHNRSGGRVVPLENIKEICTIAKEHGINVHIDGARI

FNASIASGVPVKEYAGYADSVMFCLSKGLCAPVGSVVVGDRDFIERARKARKMLGGGMRQAGVL
AAAGIIALTKMVDRLKEDHENARFLALKLKEIGYSVNPEDVKTNMVILRTDNLKVNAHGFIEAL
RNSGVLANAVSDTEIRLVTHKDVSRNDIEEALNIFEKLFRKFS

*Tm*TA E88T H83F

DNA sequence

ATGGGCAGCAGCCATCATCATCATCATCACAGCAGCGGCCTGGTGCCGCGCGGCAGCCATATGA
TCGATCTGCGCAGCGATACCGTGACCAAACCGACAGAAGAAATGCGTAAAGCAATGGCTCAGGC
CGAAGTTGGCGATGATGTTTATGGCGAAGATCCGACCATTAATGAACTGGAACGTCTGGCCGCC
GAAACCTTTGGTAAAGAAGCCGCCCTGTTTGTGCCGAGCGGTACAATGGGTAATCAGGTGAGCA
TTATGGCCCATACCCAGCGCGGTGACGAAGTTATTCTGGAAGCAGATAGT**TTT**ATTTTCTGGTA
T**ACG**GTTGGTGCAATGGCTGTTCTGAGCGGTGTGATGCCGCATCCTGTTCCTGGTAAAAATGGT
GCAATGGACCCTGATGATGTTCGTAAAGCAATTCGTCCGCGTAATATTCATTTTCCGCGTACCA
GTCTGATTGCAATTGAAAATACCCATAATCGTAGCGGTGGTCGCGTTGTTCCGCTGGAAAATAT
TAAGGAAATTTGTACCATCGCCAAAGAACATGGTATTAATGTGCATATTGACGGTGCCCGTATT
TTTAATGCAAGCATTGCAAGTGGTGTTCCGGTGAAAGAATATGCCGGTTATGCCGATAGTGTTA
TGTTTTGTCTGAGTAAAGGTCTGTGTGCCCCGGTTGGTAGCGTGGTTGTTGGTGACCGTGATTT
TATTGAACGTGCACGTAAAGCACGTAAAATGCTGGGTGGTGGTATGCGTCAGGCAGGTGTTCTG
GCAGCTGCAGGTATTATTGCACTGACCAAAATGGTGGATCGCCTGAAAGAAGATCATGAAAATG
CACGCTTTCTGGCACTGAAACTGAAAGAAATTGGCTATAGTGTTAACCCGGAAGATGTGAAAAC
CAATATGGTTATTCTGCGTACCGATAATCTGAAAGTTAATGCCCACGGTTTTATTGAAGCACTG
CGCAATAGTGGCGTGCTGGCAAATGCCGTTAGCGATACCGAAATTCGCCTGGTTACCCATAAAG
ATGTTAGTCGTAATGATATTGAGGAAGCCCTGAATATTTTTGAAAAACTGTTTCGTAAGTTCAG
CTAA

Protein sequence

MGSSHHHHHHSSGLVPRGSHMIDLRSDTVTKPTEEMRKAMAQAEVGDDVYGEDPTINELERLAA
ETFGKEAALFVPSGTMGNQVSIMAHTQRGDEVILEADS**F**IFWY**T**VGAMAVLSGVMPHPVPGKNG
AMDPDDVRKAIRPRNIHFPRTSLIAIENTHNRSGGRVVPLENIKEICTIAKEHGINVHIDGARI
FNASIASGVPVKEYAGYADSVMFCLSKGLCAPVGSVVVGDRDFIERARKARKMLGGGMRQAGVL

AAAGIIALTKMVDRLKEDHENARFLALKLKEIGYSVNPEDVKTNMVILRTDNLKVNAHGFIEAL
RNSGVLANAVSDTEIRLVTHKDVSRNDIEEALNIFEKLFRKFS

*Tm*TA W86E

DNA sequence

ATGGGCAGCAGCCATCATCATCATCATCACAGCAGCGGCCTGGTGCCGCGCGGCAGCCATATGA
TCGATCTGCGCAGCGATACCGTGACCAAACCGACAGAAGAAATGCGTAAAGCAATGGCTCAGGC
CGAAGTTGGCGATGATGTTTATGGCGAAGATCCGACCATTAATGAACTGGAACGTCTGGCCGCC
GAAACCTTTGGTAAAGAAGCCGCCCTGTTTGTGCCGAGCGGTACAATGGGTAATCAGGTGAGCA
TTATGGCCCATACCCAGCGCGGTGACGAAGTTATTCTGGAAGCAGATAGTCATATTTTC**GAA**TA
TGAAGTTGGTGCAATGGCTGTTCTGAGCGGTGTGATGCCGCATCCTGTTCCTGGTAAAAATGGT
GCAATGGACCCTGATGATGTTCGTAAAGCAATTCGTCCGCGTAATATTCATTTTCCGCGTACCA
GTCTGATTGCAATTGAAAATACCCATAATCGTAGCGGTGGTCGCGTTGTTCCGCTGGAAAATAT
TAAGGAAATTTGTACCATCGCCAAAGAACATGGTATTAATGTGCATATTGACGGTGCCCGTATT
TTTAATGCAAGCATTGCAAGTGGTGTTCCGGTGAAAGAATATGCCGGTTATGCCGATAGTGTTA
TGTTTTGTCTGAGTAAAGGTCTGTGTGCCCCGGTTGGTAGCGTGGTTGTTGGTGACCGTGATTT
TATTGAACGTGCACGTAAAGCACGTAAAATGCTGGGTGGTGGTATGCGTCAGGCAGGTGTTCTG
GCAGCTGCAGGTATTATTGCACTGACCAAAATGGTGGATCGCCTGAAAGAAGATCATGAAAATG
CACGCTTTCTGGCACTGAAACTGAAAGAAATTGGCTATAGTGTTAACCCGGAAGATGTGAAAAC
CAATATGGTTATTCTGCGTACCGATAATCTGAAAGTTAATGCCCACGGTTTTATTGAAGCACTG
CGCAATAGTGGCGTGCTGGCAAATGCCGTTAGCGATACCGAAATTCGCCTGGTTACCCATAAAG
ATGTTAGTCGTAATGATATTGAGGAAGCCCTGAATATTTTTGAAAAACTGTTTCGTAAGTTCAG
CTAA

Protein sequence

MGSSHHHHHHSSGLVPRGSHMIDLRSDTVTKPTEEMRKAMAQAEVGDDVYGEDPTINELERLAA
ETFGKEAALFVPSGTMGNQVSIMAHTQRGDEVILEADSHIF**E**YEVGAMAVLSGVMPHPVPGKNG
AMDPDDVRKAIRPRNIHFPRTSLIAIENTHNRSGGRVVPLENIKEICTIAKEHGINVHIDGARI
FNASIASGVPVKEYAGYADSVMFCLSKGLCAPVGSVVVGDRDFIERARKARKMLGGGMRQAGVL

AAAGIIALTKMVDRLKEDHENARFLALKLKEIGYSVNPEDVKTNMVILRTDNLKVNAHGFIEAL
RNSGVLANAVSDTEIRLVTHKDVSRNDIEEALNIFEKLFRKFS

***Tm*TA W86Q**

DNA sequence

<u>ATGGGCAGCAGCCATCATCATCATCATCACAGCAGCGGCCTGGTGCCGCGCGGCAGCCAT</u>ATGA
TCGATCTGCGCAGCGATACCGTGACCAAACCGACAGAAGAAATGCGTAAAGCAATGGCTCAGGC
CGAAGTTGGCGATGATGTTTATGGCGAAGATCCGACCATTAATGAACTGGAACGTCTGGCCGCC
GAAACCTTTGGTAAAGAAGCCGCCCTGTTTGTGCCGAGCGGTACAATGGGTAATCAGGTGAGCA
TTATGGCCCATACCCAGCGCGGTGACGAAGTTATTCTGGAAGCAGATAGTCATATTTTC**CAG**TA
TGAAGTTGGTGCAATGGCTGTTCTGAGCGGTGTGATGCCGCATCCTGTTCCTGGTAAAAATGGT
GCAATGGACCCTGATGATGTTCGTAAAGCAATTCGTCCGCGTAATATTCATTTTCCGCGTACCA
GTCTGATTGCAATTGAAAATACCCATAATCGTAGCGGTGGTCGCGTTGTTCCGCTGGAAAATAT
TAAGGAAATTTGTACCATCGCCAAAGAACATGGTATTAATGTGCATATTGACGGTGCCCGTATT
TTTAATGCAAGCATTGCAAGTGGTGTTCCGGTGAAAGAATATGCCGGTTATGCCGATAGTGTTA
TGTTTTGTCTGAGTAAAGGTCTGTGTGCCCCGGTTGGTAGCGTGGTTGTTGGTGACCGTGATTT
TATTGAACGTGCACGTAAAGCACGTAAAATGCTGGGTGGTGGTATGCGTCAGGCAGGTGTTCTG
GCAGCTGCAGGTATTATTGCACTGACCAAAATGGTGGATCGCCTGAAAGAAGATCATGAAAATG
CACGCTTTCTGGCACTGAAACTGAAAGAAATTGGCTATAGTGTTAACCCGGAAGATGTGAAAAC
CAATATGGTTATTCTGCGTACCGATAATCTGAAAGTTAATGCCCACGGTTTTATTGAAGCACTG
CGCAATAGTGGCGTGCTGGCAAATGCCGTTAGCGATACCGAAATTCGCCTGGTTACCCATAAAG
ATGTTAGTCGTAATGATATTGAGGAAGCCCTGAATATTTTTGAAAAACTGTTTCGTAAGTTCAG
CTAA

Protein sequence

<u>MGSSHHHHHHSSGLVPRGSH</u>MIDLRSDTVTKPTEEMRKAMAQAEVGDDVYGEDPTINELERLAA
ETFGKEAALFVPSGTMGNQVSIMAHTQRGDEVILEADSHIF**Q**YEVGAMAVLSGVMPHPVPGKNG
AMDPDDVRKAIRPRNIHFPRTSLIAIENTHNRSGGRVVPLENIKEICTIAKEHGINVHIDGARI
FNASIASGVPVKEYAGYADSVMFCLSKGLCAPVGSVVVGDRDFIERARKARKMLGGGMRQAGVL

AAAGIIALTKMVDRLKEDHENARFLALKLKEIGYSVNPEDVKTNMVILRTDNLKVNAHGFIEAL
RNSGVLANAVSDTEIRLVTHKDVSRNDIEEALNIFEKLFRKFS

_Tm_TA W86Q E88A

DNA sequence

<u>ATGGGCAGCAGCCATCATCATCATCATCACAGCAGCGGCCTGGTGCCGCGCGGCAGCCAT</u>ATGA
TCGATCTGCGCAGCGATACCGTGACCAAACCGACAGAAGAAATGCGTAAAGCAATGGCTCAGGC
CGAAGTTGGCGATGATGTTTATGGCGAAGATCCGACCATTAATGAACTGGAACGTCTGGCCGCC
GAAACCTTTGGTAAAGAAGCCGCCCTGTTTGTGCCGAGCGGTACAATGGGTAATCAGGTGAGCA
TTATGGCCCATACCCAGCGCGGTGACGAAGTTATTCTGGAAGCAGATAGTCATATTTTC**CAG**TA
T**GCG**GTTGGTGCAATGGCTGTTCTGAGCGGTGTGATGCCGCATCCTGTTCCTGGTAAAAATGGT
GCAATGGACCCTGATGATGTTCGTAAAGCAATTCGTCCGCGTAATATTCATTTTCCGCGTACCA
GTCTGATTGCAATTGAAAATACCCATAATCGTAGCGGTGGTCGCGTTGTTCCGCTGGAAAATAT
TAAGGAAATTTGTACCATCGCCAAAGAACATGGTATTAATGTGCATATTGACGGTGCCCGTATT
TTTAATGCAAGCATTGCAAGTGGTGTTCCGGTGAAAGAATATGCCGGTTATGCCGATAGTGTTA
TGTTTTGTCTGAGTAAAGGTCTGTGTGCCCCGGTTGGTAGCGTGGTTGTTGGTGACCGTGATTT
TATTGAACGTGCACGTAAAGCACGTAAAATGCTGGGTGGTGGTATGCGTCAGGCAGGTGTTCTG
GCAGCTGCAGGTATTATTGCACTGACCAAAATGGTGGATCGCCTGAAAGAAGATCATGAAAATG
CACGCTTTCTGGCACTGAAACTGAAAGAAATTGGCTATAGTGTTAACCCGGAAGATGTGAAAAC
CAATATGGTTATTCTGCGTACCGATAATCTGAAAGTTAATGCCCACGGTTTTATTGAAGCACTG
CGCAATAGTGGCGTGCTGGCAAATGCCGTTAGCGATACCGAAATTCGCCTGGTTACCCATAAAG
ATGTTAGTCGTAATGATATTGAGGAAGCCCTGAATATTTTTGAAAAACTGTTTCGTAAGTTCAG
CTAA

Protein sequence

<u>MGSSHHHHHHSSGLVPRGSH</u>MIDLRSDTVTKPTEEMRKAMAQAEVGDDVYGEDPTINELERLAA
ETFGKEAALFVPSGTMGNQVSIMAHTQRGDEVILEADSHIF**Q**Y**A**VGAMAVLSGVMPHPVPGKNG
AMDPDDVRKAIRPRNIHFPRTSLIAIENTHNRSGGRVVPLENIKEICTIAKEHGINVHIDGARI
FNASIASGVPVKEYAGYADSVMFCLSKGLCAPVGSVVVGDRDFIERARKARKMLGGGMRQAGVL

AAAGIIALTKMVDRLKEDHENARFLALKLKEIGYSVNPEDVKTNMVILRTDNLKVNAHGFIEAL
RNSGVLANAVSDTEIRLVTHKDVSRNDIEEALNIFEKLFRKFS

*Tm*TA W86Q E88T

DNA sequence

ATGGGCAGCAGCCATCATCATCATCATCACAGCAGCGGCCTGGTGCCGCGCGGCAGCCATATGA
TCGATCTGCGCAGCGATACCGTGACCAAACCGACAGAAGAAATGCGTAAAGCAATGGCTCAGGC
CGAAGTTGGCGATGATGTTTATGGCGAAGATCCGACCATTAATGAACTGGAACGTCTGGCCGCC
GAAACCTTTGGTAAAGAAGCCGCCCTGTTTGTGCCGAGCGGTACAATGGGTAATCAGGTGAGCA
TTATGGCCCATACCCAGCGCGGTGACGAAGTTATTCTGGAAGCAGATAGTCATATTTTC**CAG**TA
T**ACG**GTTGGTGCAATGGCTGTTCTGAGCGGTGTGATGCCGCATCCTGTTCCTGGTAAAAATGGT
GCAATGGACCCTGATGATGTTCGTAAAGCAATTCGTCCGCGTAATATTCATTTTCCGCGTACCA
GTCTGATTGCAATTGAAAATACCCATAATCGTAGCGGTGGTCGCGTTGTTCCGCTGGAAAATAT
TAAGGAAATTTGTACCATCGCCAAAGAACATGGTATTAATGTGCATATTGACGGTGCCCGTATT
TTTAATGCAAGCATTGCAAGTGGTGTTCCGGTGAAAGAATATGCCGGTTATGCCGATAGTGTTA
TGTTTTGTCTGAGTAAAGGTCTGTGTGCCCCGGTTGGTAGCGTGGTTGTTGGTGACCGTGATTT
TATTGAACGTGCACGTAAAGCACGTAAAATGCTGGGTGGTGGTATGCGTCAGGCAGGTGTTCTG
GCAGCTGCAGGTATTATTGCACTGACCAAAATGGTGGATCGCCTGAAAGAAGATCATGAAAATG
CACGCTTTCTGGCACTGAAACTGAAAGAAATTGGCTATAGTGTTAACCCGGAAGATGTGAAAAC
CAATATGGTTATTCTGCGTACCGATAATCTGAAAGTTAATGCCCACGGTTTTATTGAAGCACTG
CGCAATAGTGGCGTGCTGGCAAATGCCGTTAGCGATACCGAAATTCGCCTGGTTACCCATAAAG
ATGTTAGTCGTAATGATATTGAGGAAGCCCTGAATATTTTTGAAAAACTGTTTCGTAAGTTCAG
CTAA

Protein sequence

MGSSHHHHHHSSGLVPRGSHMIDLRSDTVTKPTEEMRKAMAQAEVGDDVYGEDPTINELERLAA
ETFGKEAALFVPSGTMGNQVSIMAHTQRGDEVILEADSHIF**Q**Y**T**VGAMAVLSGVMPHPVPGKNG
AMDPDDVRKAIRPRNIHFPRTSLIAIENTHNRSGGRVVPLENIKEICTIAKEHGINVHIDGARI

FNASIASGVPVKEYAGYADSVMFCLSKGLCAPVGSVVVGDRDFIERARKARKMLGGGMRQAGVL
AAAGIIALTKMVDRLKEDHENARFLALKLKEIGYSVNPEDVKTNMVILRTDNLKVNAHGFIEAL
RNSGVLANAVSDTEIRLVTHKDVSRNDIEEALNIFEKLFRKFS

***Tm*TA W86Q E88A F85M (*Tm*TA^{QAM})**

DNA sequence

ATGGGCAGCAGCCATCATCATCATCATCACAGCAGCGGCCTGGTGCCGCGCGGCAGCCATATGA
TCGATCTGCGCAGCGATACCGTGACCAAACCGACAGAAGAAATGCGTAAAGCAATGGCTCAGGC
CGAAGTTGGCGATGATGTTTATGGCGAAGATCCGACCATTAATGAACTGGAACGTCTGGCCGCC
GAAACCTTTGGTAAAGAAGCCGCCCTGTTTGTGCCGAGCGGTACAATGGGTAATCAGGTGAGCA
TTATGGCCCATACCCAGCGCGGTGACGAAGTTATTCTGGAAGCAGATAGTCATATT**ATGCAG**TA
T**GCG**GTTGGTGCAATGGCTGTTCTGAGCGGTGTGATGCCGCATCCTGTTCCTGGTAAAAATGGT
GCAATGGACCCTGATGATGTTCGTAAAGCAATTCGTCCGCGTAATATTCATTTTCCGCGTACCA
GTCTGATTGCAATTGAAAATACCCATAATCGTAGCGGTGGTCGCGTTGTTCCGCTGGAAAATAT
TAAGGAAATTTGTACCATCGCCAAAGAACATGGTATTAATGTGCATATTGACGGTGCCCGTATT
TTTAATGCAAGCATTGCAAGTGGTGTTCCGGTGAAAGAATATGCCGGTTATGCCGATAGTGTTA
TGTTTTGTCTGAGTAAAGGTCTGTGTGCCCCGGTTGGTAGCGTGGTTGTTGGTGACCGTGATTT
TATTGAACGTGCACGTAAAGCACGTAAAATGCTGGGTGGTGGTATGCGTCAGGCAGGTGTTCTG
GCAGCTGCAGGTATTATTGCACTGACCAAAATGGTGGATCGCCTGAAAGAAGATCATGAAAATG
CACGCTTTCTGGCACTGAAACTGAAAGAAATTGGCTATAGTGTTAACCCGGAAGATGTGAAAAC
CAATATGGTTATTCTGCGTACCGATAATCTGAAAGTTAATGCCCACGGTTTTATTGAAGCACTG
CGCAATAGTGGCGTGCTGGCAAATGCCGTTAGCGATACCGAAATTCGCCTGGTTACCCATAAAG
ATGTTAGTCGTAATGATATTGAGGAAGCCCTGAATATTTTTGAAAAACTGTTTCGTAAGTTCAG
CTAA

Protein sequence

MGSSHHHHHHSSGLVPRGSHMIDLRSDTVTKPTEEMRKAMAQAEVGDDVYGEDPTINELERLAA
ETFGKEAALFVPSGTMGNQVSIMAHTQRGDEVILEADSHI**MQ**Y**A**VGAMAVLSGVMPHPVPGKNG
AMDPDDVRKAIRPRNIHFPRTSLIAIENTHNRSGGRVVPLENIKEICTIAKEHGINVHIDGARI
FNASIASGVPVKEYAGYADSVMFCLSKGLCAPVGSVVVGDRDFIERARKARKMLGGGMRQAGVL

AAAGIIALTKMVDRLKEDHENARFLALKLKEIGYSVNPEDVKTNMVILRTDNLKVNAHGFIEAL
RNSGVLANAVSDTEIRLVTHKDVSRNDIEEALNIFEKLFRKFS

MIDLRSDTVTKPTEEMRKAMAQAEVGDDVYGEDPTINELERLAAETFGKEAALFVPSGTMGNQV
SIMAHTQRGDEVILEADSHI**MW**Y**E**VGAMAVLSGVMPHPVPGKNGAMDPDDVRKAIRPRNIHFPR
TSLIAIENTHNRSGGRVVPLENIKEICTIAKEHGINVHIDGARIFNASIASGVPVKEYAGYADS
VMFCLSKGLCAPVGSVVVGDRDFIERARKARKMLGGGMRQAGVLAAAGIIALTKMVDRLKEDHE
NARFLALKLKEIGYSVNPEDVKTNMVILRTDNLKVNAHGFIEALRNSGVLANAVSDTEIRLVTH
KDVSRNDIEEALNIFEKLFRKFS

Table S6. Primers used for site-saturation mutagenesis for the engineering of *Tm*TA.

DNA template	Primer name	Primer sequence
***Tm*TA**	W86X_fwd_TGG	CTGGAAGCAGATAGTCATATTTTC**TGG**TATGAAGTTGG
	W86X_fwd_NDT	CTGGAAGCAGATAGTCATATTTTC**NDT**TATGAAGTTGG
	W86X_fwd_VHG	CTGGAAGCAGATAGTCATATTTTC**VHG**TATGAAGTTGG
	W86X_rev	GAAAATATGACTATCTGCTTCCAGAATAACTTCG
***Tm*TA W86Q**	E88X_fwd_TGG	GATAGTCATATTTTCCAGTAT**TGG**GTTGGTGCAATGGCTG
	E88X_fwd_NDT	GATAGTCATATTTTCCAGTAT**NDT**GTTGGTGCAATGGCTG
	E88X_fwd_VHG	GATAGTCATATTTTCCAGTAT**VHG**GTTGGTGCAATGGCTG
	E88X_rev	ATACTGGAAAATATGACTATCTGCTTCCAGAATAACTTCG
***Tm*TA**	H83X_fwd_TGG	GTTATTCTGGAAGCAGATAGT**TGG**ATTTTCTGGTATACGGTTGGTGC

**E88T**	H83X_fwd_NDT	GTTATTCTGGAAGCAGATAGT**NDT**ATTTTCTGGTATACGGTTGGTGC
	H83X_fwd_VHG	GTTATTCTGGAAGCAGATAGT**VHG**ATTTTCTGGTATACGGTTGGTGC
	H83X_rev	ACTATCTGCTTCCAGAATAACTTCGTCACCGCGCTG
***Tm*TA W86Q E88T**	H83X_fwd_TGG	GTTATTCTGGAAGCAGATAGT**TGG**ATTTTCCAGTATACGGTTGGTG
	H83X_fwd_NDT	GTTATTCTGGAAGCAGATAGT**NDT**ATTTTCCAGTATACGGTTGGTG
	H83X_fwd_VHG	GTTATTCTGGAAGCAGATAGT**VHG**ATTTTCCAGTATACGGTTGGTG
	H83X_rev	ACTATCTGCTTCCAGAATAACTTCGTC
***Tm*TA W86Q E88A**	F85X_fwd_TGG	CTGGAAGCAGATAGTCATATT**TGG**CAGTATGCGGTTGGTG
	F85X _fwd_NDT	CTGGAAGCAGATAGTCATATT**NDT**CAGTATGCGGTTGGTG
	F85X _fwd_VHG	CTGGAAGCAGATAGTCATATT**VHG**CAGTATGCGGTTGGTG
	F85X _rev	AATATGACTATCTGCTTCCAGAATAACTTCGTCAC

V. UV-Vis spectroscopic analysis

UV-visible spectra (300–600 nm) were recorded on a Shimadzu UV1800 spectrophotometer using a quartz cuvette. In this UV-vis spectroscopic analysis, 4 µL *Tm*TA W86Q (30 mg/mL in Ches buffer, pH = 8.0) in 100 mM Ches buffer (196 µL) was used (final *Tm*TA W86Q concentration: 15 µM) (Figure S4**)**. First, the UV-vis spectrum of *Tm*TA W86Q was recorded. A strong absorption band at 425 nm is characteristic of the internal aldimine formed between the PLP cofactor and the conserved lysine residue K199 of *Tm*TA. 6 µL stock solution of D- or L-alanine **2a** (500 mM in Ches buffer, 100 mM, pH= 8.0) was then added to the *Tm*TA W86Q solution (final alanine **2a** concentration: 15 mM). Upon mixing, a new signal at 500 nm quickly emerged, indicating the formation of the quinonoid species. This UV-vis measurement was carried out with *Tm*TA W86Q at pH 8.0 and 9.0 and the spectra are provided in Fig. S2. Overall, a stronger quinonoid absorption band (501 nm) was observed under more basic conditions, indicating a higher quinonoid population is present at a higher pH. Furthermore, a stronger quinonoid signal was observed when D-alanine was added. This result shows that the formation of quinonoid species is much more facile with D-alanine than with L-alanine.

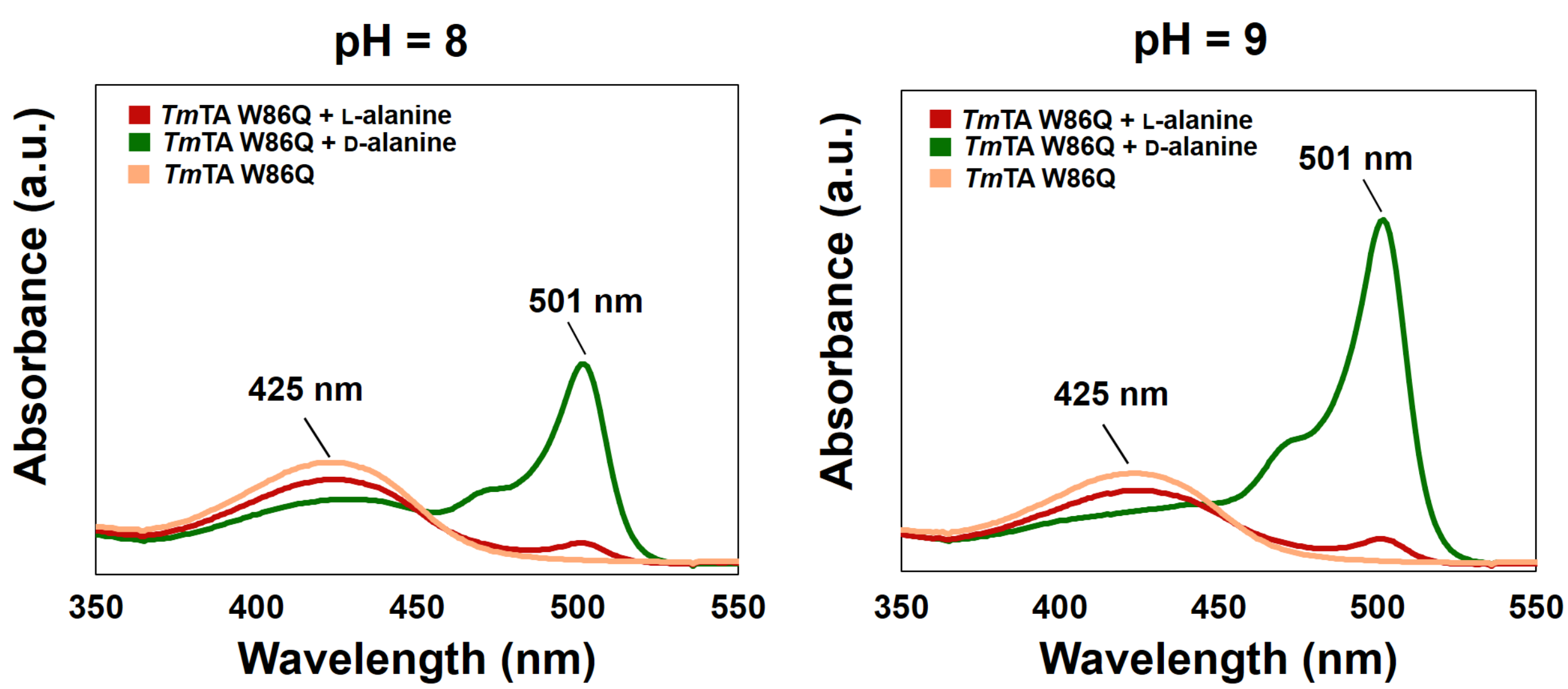

Figure S2. UV-vis spectra of *Tm*TA W86Q.

Similar UV-vis measurements were also carried out with *Tm*TA W86Q E88A and *Tm*TA W86Q E88A F85M (*Tm*TA^{QAM}) (Fig. S3 and S4**)**. With all the three *Tm*TA variants, characteristic

signals of the quinonoid species were observed when D- or L-alanine was added. The quinonoid signals at 501 nm were stronger at pH 9.0 than pH 8.0.

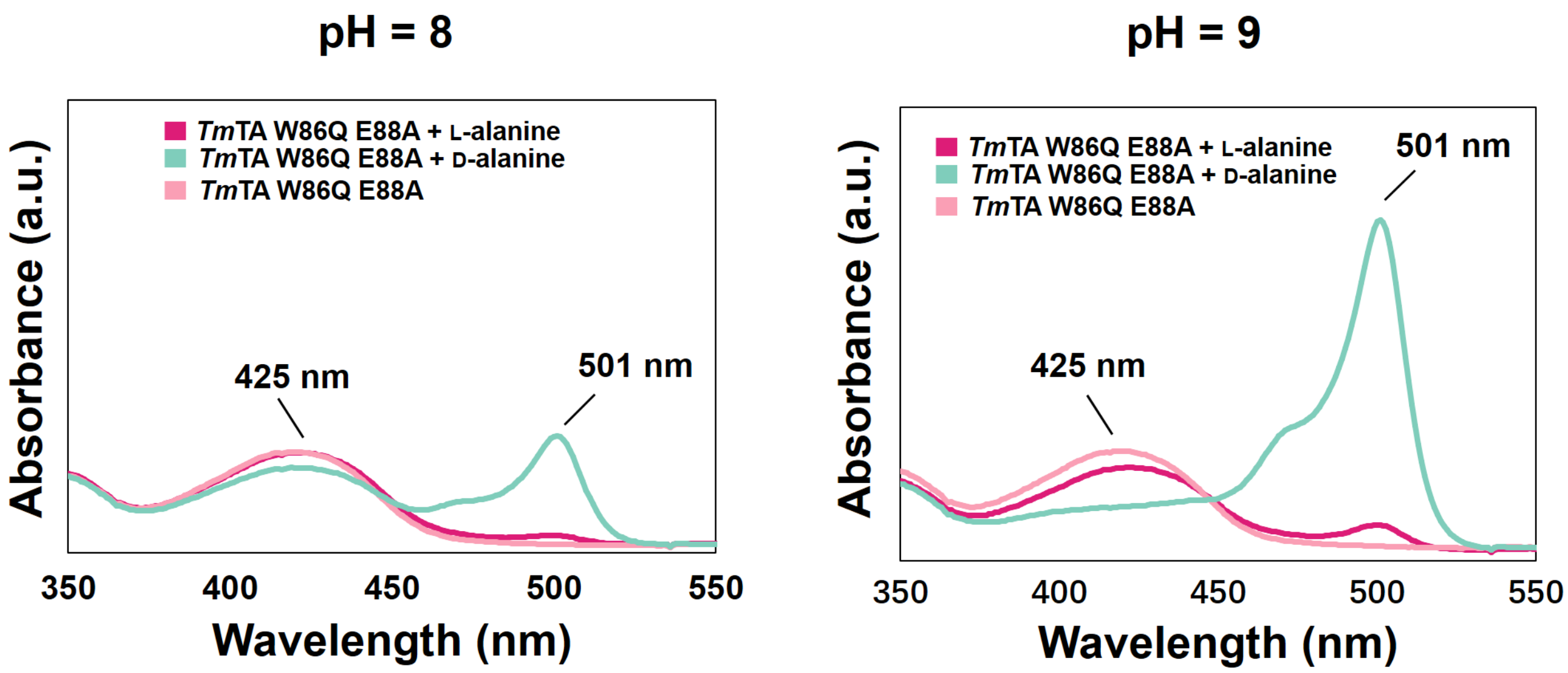

Figure S3. UV-vis spectra of *Tm*TA W86Q E88A.

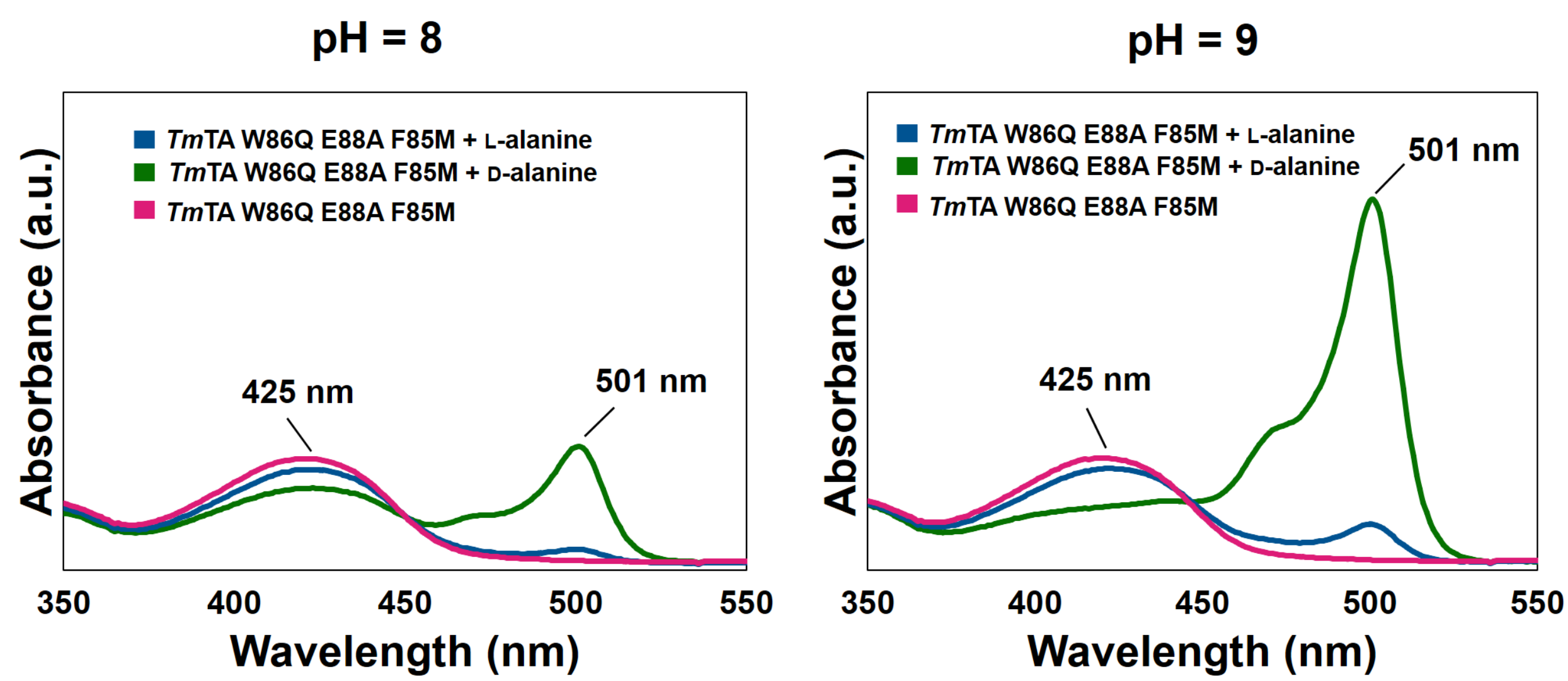

Figure S4. UV-vis spectra of the *Tm*TA W86Q E88A F85M (*Tm*TA^{QAM}).

VI. DMPO spin-trapping EPR experiment

EPR Spectroscopy Methods

Electron paramagnetic resonance spectroscopy was performed at the CalEPR center in the Department of Chemistry at the University of California, Davis. X-band (9.4 GHz) continuous wave EPR spectra were recorded at room temperature on a Bruker BioSpin E500 spectrometer equipped with a super high Q resonator (ER4122SHQE). All spectra were recorded under slow-passage, non-saturating conditions with the following settings: microwave frequency = 9.39 GHz, microwave power = 2 mW, conversion time = 60 s, modulation frequency = 100 kHz, modulation amplitude = 1 gauss. Spectral simulations were performed in MATLAB 2023b with EasySpin 6.0.0-dev package.[9]

For spin trap experiments, typical samples contain 20 mL reaction mixture of 5.0 mM PLP, 5.0 mM alanine (**2a**), 5.0 mM benzylboronic acid pinacol ester (**1a**) and 40 mM 5,5-dimethyl-1-pyrroline-*N*-oxide (DMPO) in KPi buffer (pH = 8.5). For control experiments, the specific individual component as indicated was omitted. The mixture was transferred to a glass capillary tube made from the 9-inch disposable glass pipette with the tip flame-sealed. The samples were then illuminated under 440 nm LED light for 1 min when necessary. A mixture of CH_2Cl_2 and EtOAc was added to the capillary tube to extract the spin trap adduct. EPR spectra were then recorded to analyze the spin-trapped product.

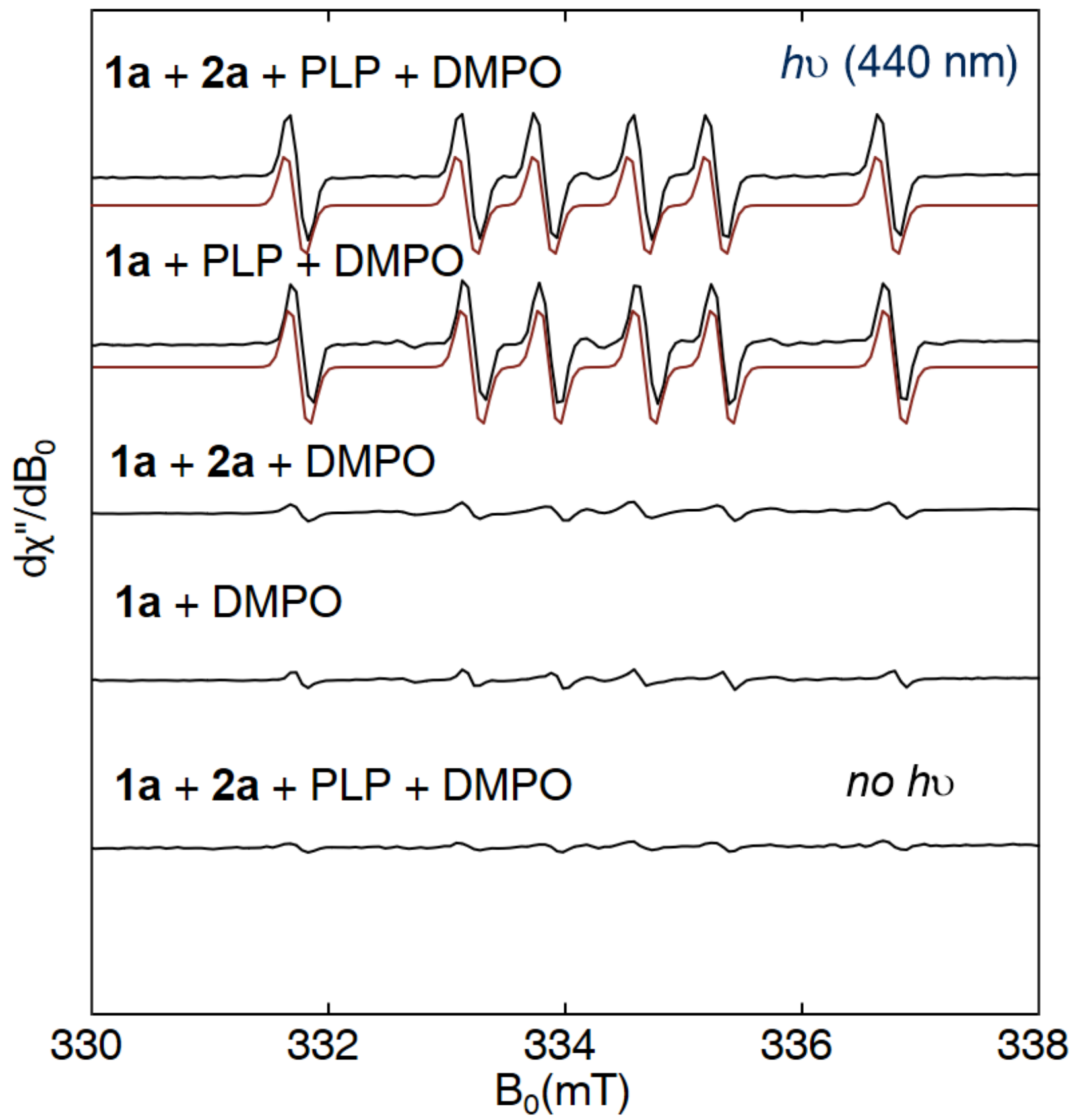

Figure S5. EPR studies to understand the mechanism of photoinduced radical generation.

VII. Radical clock reaction

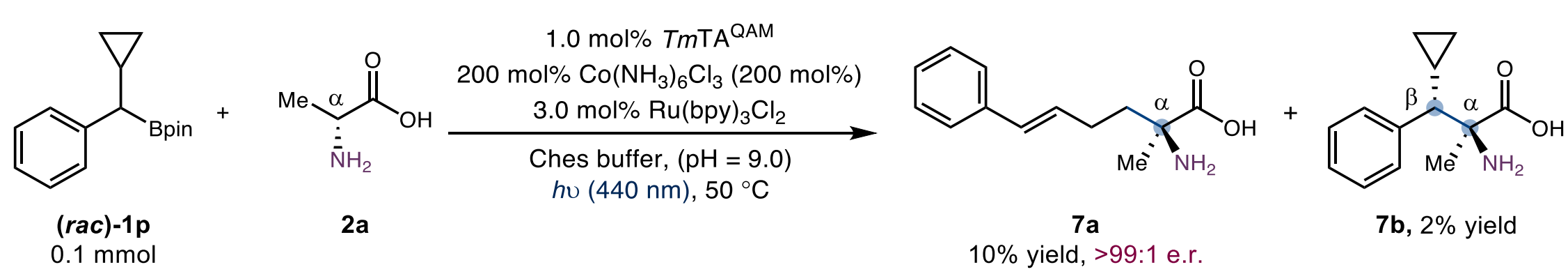

Experimental Procedure. *Tm*TA^{QAM} (ca. 30 mg/mL, 1 mL/tube) was thawed from –80 °C freezer and kept on ice. In a Coy anaerobic chamber, the following stock solutions were prepared: organoboron substrate (*rac*)-**1p** (200 mM in degassed DMSO), $Ru(bpy)_3Cl_2$ (20.0 mM in degassed DMSO), D-alanine (500 mM in degassed 100 mM Ches buffer), PLP (50 mM in 100 mM Ches buffer, pH = 7.5), $Co(NH_3)_6Cl_3$ (200 mM in degassed water). To a 50 mL round bottom flask equipped with a stir bar were added Ches buffer (8 mL, pH = 9.0), 0.5 mL organoboron substrate stock solution, 2 mL D-alanine stock solution, 0.2 mL PLP stock solution, 1 mL $Co(NH_3)_6Cl_3$ stock

solution, 0.15 mL $Ru(bpy)_3Cl_2$ stock solution, and ca. 1.2 mL *Tm*TA^{QAM} enzyme solution (the volume of the enzyme stock solution is dependent on the specific concentration of the enzyme sample used). The total reaction volume was 12.5 mL; the final concentrations of each reaction component were as follows: 8.0 mM organoboron substrate, 80.0 mM D-alanine, 16.0 mM $Co(NH_3)_6Cl_3$, 0.8 mM PLP, 80 μM *Tm*TA^{QAM} and 0.24 mM $Ru(bpy)_3Cl_2$. The reaction tube was then sealed and removed from the Coy anaerobic chamber and submerged in a water bath preheated to 50 °C. The reaction mixture was allowed to stir at 500 rpm and 50 °C and illuminated with three Kessil LED lamps (440 nm, 45 W, 50% intensity) for 12 h. The reaction mixture was then quenched with 1 M HCl (100 mL) and MeCN (100 mL) and centrifuged (4 ˚C, 30 min, 4500 rpm). The supernatant was concentrated *in vacuo* with the aid of a rotary evaporator until the total volume was less than50 mL. The residue was centrifuged (30 min, 4500 rpm) again to separate the insoluble fraction. The supernatant was further concentrated *in vacuo* with the aid of a rotary evaporator to close to dryness. Deuterium oxide (D_2O, 4 mL) was added to dissolve the residue, 4-Dimethylaminopyridine (DMAP, 9.0 mg) was added as the internal standard. The yield and the ratio of **7a**:**7b** = 5:1 were determined by crude ^{1}HNMR. LC-MS yield was determined on 6 μM scale reaction using 2-phenylbutyric acid (100 mM in CH_3CN/1 M aq. HCl) as internal standard.

Approximately 10 mL of water was added to the mixture. The resulting mixture was filtered through a 4.5 μm membrane filter and loaded onto a C18 column (12 g catridge) that had been equilibrated to water with 0.1% HCOOH. The column was first washed with 10 column volumes (CV) of water (0.1% HCOOH) to remove excessive salts. Then, the product was eluted with a gradient of 0% to 20% MeCN in water over 12 CVs. Product-containing fractions were combined and concentrated *in vacuo* to remove all the organic solvents. The residual aqueous solution was frozen in liquid nitrogen and lyophilized using a Christ Alpha 1-2 LDplus lyophilizer to give the amino acid product. Product **7a** was isolated as an HCl adduct as a white solid. Determination of enantiomeric ratio (e.r.) of **7a** using Fmoc chloride derivatization and normal phase chiral HPLC analysis.

VIII. Pierce™ BCA protein assay

Pierce™ BCA protein assay kits was purchased from Thermo Scientific. Following the method described by Thermo Scientific, we prepare the bovine serum albumin (BSA) standards on varying concentrations (125, 250, 500, 750, and 1000 μg/mL). The PLP enzyme *Tm*TA^{QAM} was diluted to 500–1500 μg/mL. Measurements were made by following the standard protocol from Thermo Scientific. We found that the *Tm*TA concentrations determined by the BCA method to be consistently larger than the Nanodrop A280 method. The BCA method was used to have a conservative estimation of the enzyme total turnover numbers.

Figure S6. BCA assay calibration curve using the BSA standard

entry	BSA (mg/mL)	Absorbance of BSA
1	125	0.19
2	250	0.33
3	500	0.62
4	750	0.8
5	1000	1.2

IX. Synthesis and characterization of substrates

Compounds (***rac***)-**1a, 1b, 1c, 1d, 1e', 1f, 1g, 1h, 1i, 1j, 1l, 1m** and **1o** are known compounds.[1, 10-14] Compounds (***rac***)-**1k** and (***rac***)-**1n** were synthesized according to **General Procedure B** as detailed below[13].

Compound (***rac***)-**1k** was prepared following **General Procedure B**.

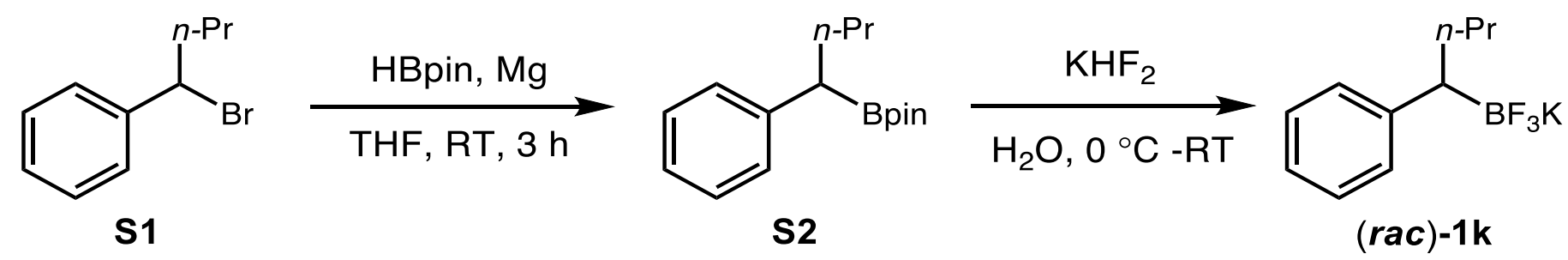

General Procedure B. A 100 mL round-bottom flask equipped with a stir bar was charged with magnesium turnings (290 mg, 12 mmol, 1.2 equiv) purchased from Strem. The flask was evacuated and backfilled with nitrogen and this process was repeated for a total of three times. THF (40 mL, 0.3 M) was added, followed by the addition of a very small iodine crystal. The mixture was allowed to stir until the color of I_2 disappeared. Pinacolborane (1.2 mL, 12.0 mmol, 1.2 equiv) was added. Benzyl bromide **S1** (10 mmol, 1.0 equiv) in 5 mL THF was added slowly over 10 min at room temperature to allow the Grignard reagent to form gently. The reaction mixture was allowed to stir at room temperature for 3 h, cooled to 0 °C, slowly acidified with 3 M HCl (15 mL) (Caution! hydrogen evolution), and allowed to stir at room temperature for 30 min. The reaction mixture was then transferred to a separatory funnel and extracted with diethyl ether (3 × 15 mL). The combined organic layers were dried over anhydrous $MgSO_4$, filtered, and concentrated *in vacuo* with the aid of a rotary evaporator.[15]

S2 was dissolved in MeOH (50 mL) and cooled to 0 °C. Saturated aqueous KHF_2 (122 mmol, 8.1 equiv) was added dropwise over 30 min and the solution was allowed to warm to room temperature. The resulting suspension was concentrated *in vacuo* with the aid of a rotary evaporator. The mixture was resuspended in toluene (150 mL), and pinacol and H_2O were removed azeotropically with the aid of a rotary evaporator at 40 °C. The remaining solid was dried under high vacuum, dissolved in hot acetone (3 × 100 mL) and filtered to remove inorganic salts. The filtrate was concentrated to a minimal volume (5–20 mL) and hexanes (500 mL) was added to provide a white solid. The solid product was isolated by filtration and washed with hexanes (50

mL) and CH_2Cl_2 (30 mL) to afford the desired benzyltrifluoroborate salt (***rac***)-**1k** as a white powder.[16]

Compound (***rac***)-**1n** was prepared following **General Procedure C**.

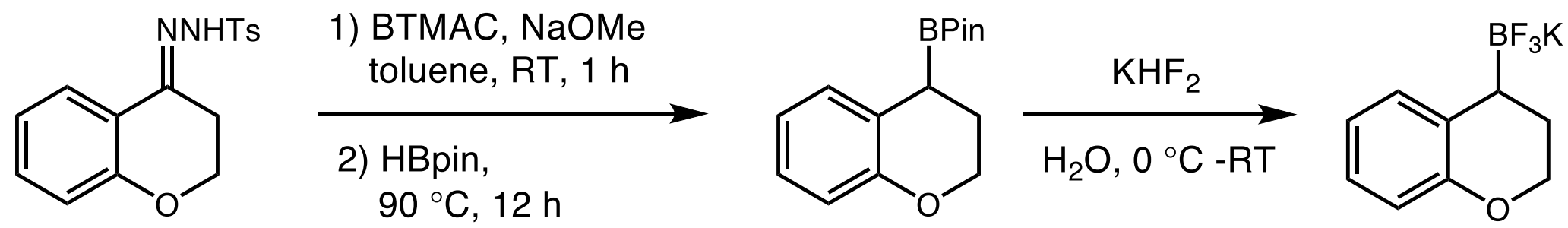

General Procedure C. A Schlenk tube was charged with *N*-tosylhydrazone **S3** (10 mmol, 1.0 equiv), NaOMe (10 mmol, 1.0 equiv) and benzyltrimethylammonium chloride (1.0 mmol, 1.0 equiv). The Schlenk tube was evacuated and backfilled with N_2 and the procedure was repeated for a total of three times. Toluene (80 mL) was added, and the mixture was allowed to stir at room temperature for 1 h under a N_2 atmosphere. Pinacolborane (30 mmol, 4.35 mL) was added and the mixture was stirred for 5 min at room temperature before it was heated to 90 ºC for another 12 h. After the reaction was cooled down to room temperature, a mixture of hexanes/ethyl acetate (1:1, 60 mL) and several drops of methanol were added. The mixture was stirred for 5 min and filtered through a short plug of silica gel eluting with ethyl acetate (60 mL). Organic solvents were removed *in vacuo* with the aid of a rotary evaporator. 20 mL pentane was added to the residue and the mixture was sonicated for 10 min. The mixture was filtered through a celite plug eluting with pentane (3 × 20 mL). The filtrate was combined, dried over $MgSO_4$, and the solvents were removed *in vacuo* with the aid of a rotary evaporator. This crude product was purified by silica gel chromatography (hexanes : EtOAc = 10 : 1) to afford the borylation product, which was subsequently converted to the trifluoroborate salt using the same procedure described in General Procedure B.

Characterization data for secondary benzyltrifluoroborates

Potassium trifluoro (1-phenylbutyl)- borate ((*rac*)-1k)

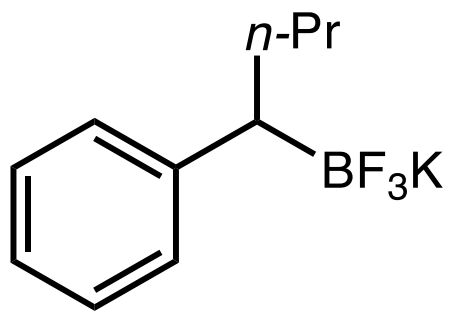

^{1}H NMR (400 MHz, DMSO-d_6) δ: 7.10 – 6.95 (m, 4H), 6.86 (m, 1H), 1.57 (td, *J* = 9.4, 6.9 Hz, 1H), 1.46 (dq, *J* = 8.5, 4.7 Hz, 2H), 1.16 – 0.95 (m, 2H), 0.74 (t, *J* = 7.3 Hz, 3H) ppm. ^{13}C NMR (101 MHz, DMSO-d_6) δ: 150.8, 128.3, 126.74, 122.1, 34.1, 25.3, 22.1, 14.4 ppm. ^{19}F NMR (376 MHz, DMSO-d_6) δ: -141.4 ppm. ^{11}B NMR (128 MHz, DMSO) δ: 3.9 ppm. IR: 2952.54, 2924.47, 2866.87, 1599.08, 1491.46, 1451.09, 1292.6, 1221.43, 1115.33, 1070.92, 937.72, 872.22, 856.14, 773.05, 751.22, 707.51, 608.96, 585.25, 547.79 cm^{-1}. HRMS (ESI) (m/z) for [M-K]$^{-}$ $C_{10}H_{13}BF_3$ requires 201.1064, observed 201.1068.

Chroman-4-yltrifluoroboronate, potassium salt ((***rac***)**-1n**)

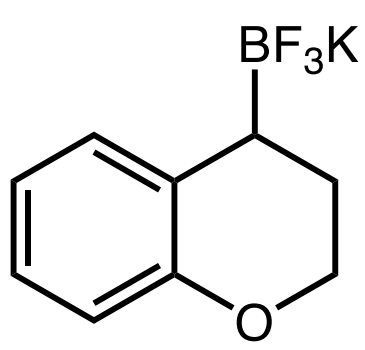

^{1}H NMR (400 MHz, DMSO-d_6) δ: 7.11 (d, *J* = 7.5 Hz, 1H), 6.80 – 6.71 (m, 1H), 6.59 (td, *J* = 7.5, 1.4 Hz, 1H), 6.51 (dd, *J* = 8.0, 1.4 Hz, 1H), 4.16 (td, *J* = 9.5, 2.9 Hz, 1H), 3.92 (dt, *J* = 9.5, 4.3 Hz, 1H), 1.91 – 1.65 (m, 2H), 1.61 (m, 1H) ppm. ^{13}C NMR (101 MHz, DMSO-d_6) δ: 13C NMR (126 MHz, DMSO) δ 154.2, 130.7, 130.2, 123.4, 118.6, 115.0, 65.2, 27.1, 24.7 ppm. ^{19}F NMR (376 MHz, DMSO-d_6) δ: -140.5 ppm. ^{11}B NMR (128 MHz, DMSO) δ: 4.3 ppm. IR: 2969.85, 1571.02, 1483.65, 1448.94, 1299, 1229.22, 1214.05, 1184.15, 1117.75, 1084.62, 1058, 986.64, 922.23, 826.75, 767.18, 755.12, 695.17, 576.64, 495.75 cm^{-1}. HRMS (ESI) (m/z) for [M-K]$^{-}$ $C_9H_9BF_3O$ requires 201.0704, observed 201.0707.

X. Synthesis and characterization of products

Racemic amino acids **3a-3o** were synthesized using **General Procedure D**.[7] Racemic Ni complex **S5** was synthesized and characterized according to a previously described procedure[17]. Amino acids **4a-4e** and **5a-5c** were known compounds.[1-2] Racemic amino acid **7a** was synthesized using **General Procedure E.**[18] Amino acids **7b** and **7b-dia** were synthesized using **General Procedure F.**[19-20]

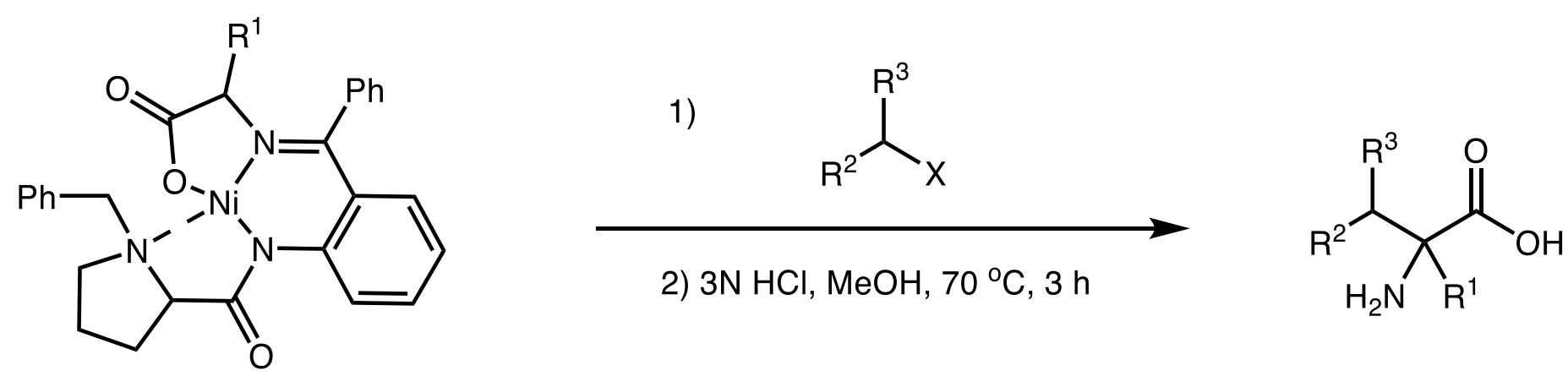

S5 **3**

General Procedure D. A 100 mL oven-dried round-bottom flask equipped with a magnetic stir bar was charged with the Ni complex **S5** (2.0 mmol, 1 equiv) and NaOH (20 mmol, 10 equiv). The reaction mixture was then evacuated and backfilled with nitrogen. This cycle was repeated for three times. Anhydrous DMF (10 mL) was then added. The corresponding bromide (5.0 mmol, 2.5 equiv) was dissolved in anhydrous DMF (10 mL) and added dropwise into reaction mixture via a syringe, and the resulting mixture was stirred at 25 °C for 24 h. The reaction mixture was quenched by the addition of 200 mL ice water and extracted with CH_2Cl_2 (3 × 50 mL). The combined organic layers were washed with water (3 × 200 mL) and brine (200 mL), dried over anhydrous Na_2SO_4. The mixture was concentrated in vacuo with the aid of a rotary evaporator, and the residue was used for next step without further purification.

To a 100 mL round bottom flask was added 3 M HCl/MeOH (1:1, 30 mL). The residue was dissolved in MeOH (10 mL) and added dropwise into this flask using a syringe. The resulting mixture was stirred at 70 °C for 3 h and allowed to cool to room temperature. Organic solvent was removed *in vacuo* with the aid of a rotary evaporator. The remaining aqueous residue was further diluted with water (20 mL) and washed with ethyl acetate (3 × 20 mL). The obtained aqueous solution was concentrated to a final volume of 10–15 mL with the aid of a rotary evaporator and directly purified by reverse phase chromatography with the aid of a Biotage Isolera using a C18

column. The product was acidified with 6 M HCl, concentrated in vacuo with the aid of a rotary evaporator, and further lyophilized to give the corresponding amino acid HCl adduct **3**.

Amino acids **5a**-**5e** and **6a**-**6c** were known compounds.[1-2]

Amino acid **7a** was synthesized using **General Procedure E**[18].

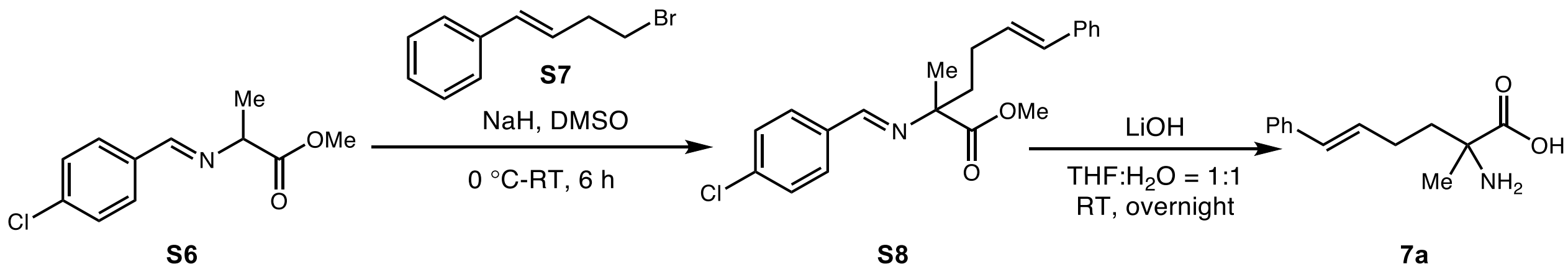

General Procedure E. In a 100 mL round bottom flask, NaH (60% in mineral oil, 330 mg, 5.5 mmol, 1.1 equiv) was weighed and flushed with argon. NaH was washed with hexanes and dried under vacuum. A solution of imine **S6** (5.0 mmol, 1.0 equiv) in dry DMSO (10.0 mL) was added under argon. The mixture was allowed to stir for 30 min, and alkyl bromide **S7** (5.0 mmol, 1.0 equiv) was added slowly. The resulting mixture was allowed to stir for 72 h. After the reaction went to completion as indicated by TLC analysis, the mixture was diluted with brine and Et_2O. The organic phase was separated, and the aqueous phase was extracted with Et_2O (3 × 20.0 mL). The combined organic layers were washed with water (5 × 100 mL), dried over $MgSO_4$, filtered, and dried *in vacuo* to afford alkylated imine **S8**. This imine was dissolved in THF (7.5 mL) and cooled to 0 °C. At 0 °C, aq. 1M HCl (7.5 mL) was added, and the resulting mixture was stirred at 0 ºC for 90 min. The reaction mixture was diluted with Et_2O and further extracted with Et_2O (3 × 20 mL). The aqueous layer was saved, and the resulting organic layer was extracted with water (50 mL). The aqueous layers were combined and concentrated *in vacuo* with the aid of a rotary evaporator. The resulting crude amino ester hydrochloride was used for the next step without further purification.

To a 100 mL round bottom flask equipped with a magnetic stir bar were added the crude amino ester hydrochloride (5 mmol, 1.0 equiv) and 1:1 THF/water (20 mL). LiOH (15.0 mmol, 720 mg, 3.0 equiv) was added in one portion. The resulting mixture was stirred at room temperature for 12 h. After completion, the organic solvent was removed *in vacuo* with the aid of a rotary evaporator. The aqueous solution was diluted with water (20 mL) and washed with ethyl acetate (3 × 20 mL). The obtained aqueous layer was concentrated *in vacuo* in to a final volume of 10–15 mL. This residue was purified by reverse phase column chromatography with the aid of

a Biotage Isolera (12 g C18 cartridge). The purified product was further acidified with 6 M HCl, concentrated in vacuo with the aid of a rotary evaporator and lyophilized to give the corresponding amino acid HCl adduct **7a**.

Amino acids **7b** and **7b-*dia*** was synthesized using **General Procedure F.** Compound **S9** was synthesized according to a previously reported procedure.[19-20]

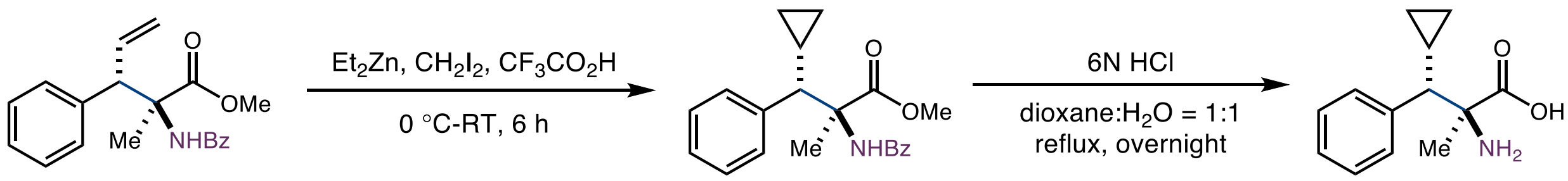

General Procedure F. A 100 mL oven-dried round-bottom flask equipped with a magnetic stir bar was evacuated and backfilled with nitrogen and this procedure was repeated for a total of three times. Et_2Zn (1.0 M in hexanes, 5.0 mL, 5.0 equiv) was added, and the mixture was cooled to 0 ℃. CH_2I_2 (4 mL, 5 equiv) was added dropwise into this mixture over 5 min at 0 ℃. The reaction mixture was stirred at 0 ℃ for 1 h. CF_3COOH (1.9 mL, 5 mmol, 5.0 equiv) was dissolved in anhydrous CH_2Cl_2 (10 mL) and the resulting solution was added dropwise into the reaction mixture at 0 ℃ over 10 min. The mixture was stirred at 0 °C for 1 h. **S9** (1 mmol, 323 mg) was dissolved in anhydrous CH_2Cl_2 (10 mL) and added dropwise into the reaction mixture at 0 ℃ over 5 min. The resulting mixture was stirred at room temperature for 6 h. The mixture was quenched with 200 mL sat. NH_4Cl aqueous solution and extracted with dichloromethane (3 × 50 mL). The combined organic layers were washed with water (3 × 200 mL) and brine (200 mL), dried over Na_2SO_4 and concentrated *in vacuo* with the aid of a rotary evaporator. The residue was directly used for the next step without further purification.

To a 100 mL round bottom flask equipped with magnetic stir bar were added **S10** (0.5 mmol) and a 1:1 mixture of 6M HCl/dioxane (20 mL). The resulting mixture was stirred at 100 °C for 12 h. After cooling down to room temperature, the organic solvent was removed *in vacuo* with the aid of a rotary evaporator. The aqueous solution was diluted with water (20 mL) and washed with ethyl acetate (3 × 20 mL). The aqueous solution was further concentrated to a final volume of 10–15 mL with the aid of a rotary evaporator. This mixture was directly purified by reverse phase chromatography with the aid of a Biotage Isolera and a C18 column (12 g cartridge) and further

acidified with 6M HCl, concentrated in vacuo with the aid of a rotary evaporator and lyophilized to give the corresponding amino acid HCl adduct **7b** and **7b-*dia***.

Characterization data for non-canonical amino acids

(2*S*,3*S*)-2-amino-2-methyl-3-phenylbutanoic acid (3a)

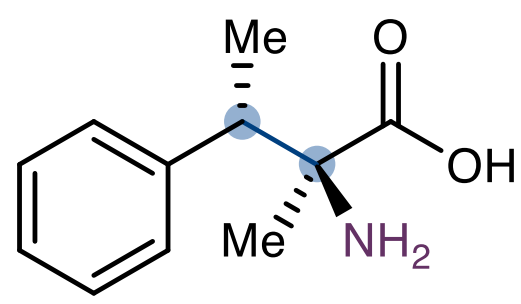

^{1}H NMR (400 MHz, D_2O) for major diastereomer (**3a**) δ: 7.49 – 7.39 (m, 3H), 7.38 – 7.33 (m, 2H), 3.46 (q, *J* = 7.3 Hz, 1H), 1.56 (s, 3H), 1.45 (d, *J* = 7.3 Hz, 3H) ppm. ^{13}C NMR (101 MHz, D_2O) for major diastereomer (**3a**) δ: 174.3, 138.7, 128.9, 128.7, 128.2, 64.0, 44.3, 18.7, 13.8 ppm. IR: 3377, 2914, 1963, 1721, 1603, 1523, 1454, 1391, 1216, 1143, 1007, 949, 875, 778, 704 cm^{-1}. HRMS (ESI) (m/z) for $[M+H]^+$ $C_{11}H_{16}NO_2$ requires 194.1176, observed 194.1183. $[\alpha]^{20}_{D}$ = -20.2 (c = 0.43, MeOH). [lit[5, 7] -31.7 (c = 0.49, MeOH)].

(2*S*,3*S*)-3a was prepared according to General Procedure A using *Tm*TA^{QAM} (0.5 mol%), derivatized using Fmoc-Cl, and analyzed by chiral HPLC.

HPLC analysis conditions: CHIRALCEL IC column (4.6 mm × 25 cm, 5 micron), 97:3 hexanes: iPrOH (0.1% TFA), flow rate = 1.0 mL/min, wavelength = 265 nm, 40 °C. t_R = 20.2 min (major), t_R = 20.8 min (minor), t_R = 27.3 min (minor), t_R = 33.6 min (major).

Yield with D-alanine: run 1: 72%; run 2: 76%; run 3: 76%; average yield: 75%.

Yield with D-alanine using 0.5 mol% *Tm*TA^{QAM}: run 1: 51%; run 2: 54%; run 3: 55%; average yield: 53%.

Diastereoselectivity determined by NMR analysis: 93:7 d.r.

Enantioselectivity of the major diastereomer determined by HPLC: >99:1 e.r.

Enantioselectivity of the minor diastereomer determined by HPLC: 96:4 e.r.

(2*S*,3*S*)-2-amino-2-methyl-3-(o-tolyl)butanoic acid (3b)

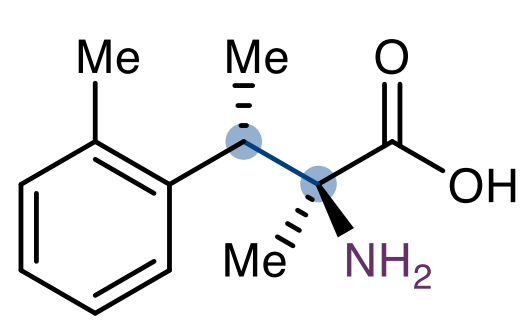

^{1}H NMR (400 MHz, D_2O) for major diastereomer (**3b**) δ: 7.29 (m, 4H), 3.76 (q, *J* = 7.2 Hz, 1H), 2.38 (s, 3H), 1.59 (s, 3H), 1.42 (d, *J* = 7.2 Hz, 3H) ppm. ^{13}C NMR (101 MHz, D_2O) for major diastereomer (**3b**) δ: 173.6, 137.8, 137.1, 131.1, 127.8, 127.5, 126.3, 63.7, 39.5, 19.7, 19.1, 15.1 ppm. IR: 3337, 3138, 2939, 2170, 1917, 1714, 1597, 1577, 1525, 1492, 1456, 1405, 1383, 1284, 1262, 1196, 1159, 1049, 995, 781, 750 cm^{-1}. HRMS (ESI) (m/z) for $[M+H]^+$ $C_{12}H_{18}NO_2$ requires 208.1338, observed 208.1333.

(2*S*,3*S*)-3b was prepared according to General Procedure A using *Tm*TA^{QAM} (0.5 mol%) derivatized using Fmoc-Cl and analyzed by chiral HPLC.

HPLC analysis conditions: CHIRALCEL IC column (4.6 mm × 25 cm, 5 micron), 97:3 hexanes: iPrOH (0.1% TFA), flow rate = 1.0 mL/min, wavelength = 265 nm, 40 °C. t_R = 8.5 min (major), t_R = 11.7 min (minor).

Yield with D-alanine: run 1: 67%; run 2: 76%; run 3: 73%; average yield: 72%.

Diastereoselectivity determined by NMR analysis: 99:1 d.r.

Enantioselectivity of the major diastereomer determined by HPLC: >99:1 e.r.

(2*S*,3*S*)-2-amino-2-methyl-3-(m-tolyl)butanoic acid (3c)

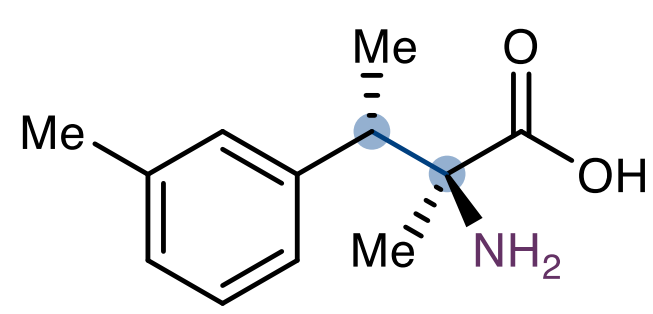

^{1}H NMR (400 MHz, D_2O) for major diastereomer (**3c**) δ: 7.33 (m, 1H), 7.23 (m, 1H), 7.19 – 7.09 (m, 2H), 3.43 (q, J = 7.3 Hz, 1H), 2.34 (s, 3H), 1.56 (s, 3H), 1.43 (d, J = 7.3 Hz, 3H) ppm. ^{13}C NMR (101 MHz, D_2O) for major diastereomer (**3c**) δ: 173.6, 139.1, 138.4, 129.3, 128.9, 125.7, 63.6, 44.2, 20.4, 18.5, 13.7 ppm. IR: 3381, 2897, 1747, 1715, 1591, 1525, 1504, 1455, 1382, 1285, 1223, 1191, 1144, 1062, 848, 794, 761, 724, 703, 644 617 cm^{-1}. HRMS (ESI) (m/z) for $[M+H]^+$ $C_{12}H_{18}NO_2$ requires 208.1338, observed 208.1333.

(2*S*,3*S*)-3c was prepared according to General Procedure A using *Tm*TA^{QAM} (0.5 mol%) derivatized using Fmoc-Cl and analyzed by chiral HPLC.

HPLC analysis conditions: CHIRALCEL IC column (4.6 mm × 25 cm, 5 micron), 95:5 hexanes: EtOH (0.1% TFA), flow rate = 1.0 mL/min, wavelength = 265 nm, 40 °C. t_R = 8.5 min (major), t_R = 8.9 min (minor), t_R = 10.9 min (minor), t_R = 13.8 min (major).

Yield with D-alanine: run 1: 73%; run 2: 76%; run 3: 75%; average yield: 75%.

Diastereoselectivity determined by NMR analysis: 90:10 d.r.

Enantioselectivity of the major diastereomer determined by HPLC: >99:1 e.r.

Enantioselectivity of the minor diastereomer determined by HPLC: 96:4 e.r.

(2*S*,3*S*)-2-amino-2-methyl-3-(p-tolyl)butanoic acid (3d)

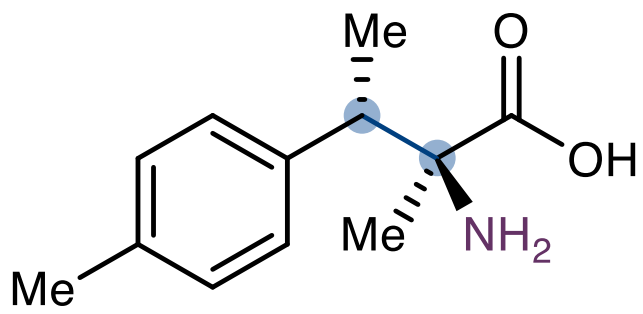

^{1}H NMR (400 MHz, D_2O) for major diastereomer (**3d**) δ: 7.33 – 7.18 (m, 4H), 3.43 (q, J = 7.3 Hz, 1H), 2.33 (s, 3H), 1.56 (s, 3H), 1.43 (d, J = 7.3 Hz, 3H) ppm. ^{13}C NMR (101 MHz, D_2O) for major diastereomer (**3d**) δ: 173.8, 138.5, 135.3, 129.5, 128.7, 63.8, 43.9, 20.0, 18.6, 13.8 ppm. IR: 2920, 1721, 1591, 1516, 1487, 1385, 1187, 1126, 866, 822, 796, 761, 722 cm^{-1}. HRMS (ESI) (m/z) for $[M+H]^+$ $C_{12}H_{18}NO_2$ requires 208.1338, observed 208.1333.

(2*S*,3*S*)-3d was prepared according to General Procedure A using *Tm*TA^{QAM} (0.5 mol%) derivatized using Fmoc-Cl and analyzed by chiral HPLC.

HPLC analysis conditions: CHIRALCEL IC column (4.6 mm × 25 cm, 5 micron), 95:5 hexanes: EtOH (0.1% TFA), flow rate = 1.0 mL/min, wavelength = 265 nm, 40 °C. t_R = 8.4 min (major), t_R = 9.5 min (minor), t_R = 12.2 min (minor), t_R = 14.2 min (major).

Yield with D-alanine: run 1: 73%; run 2: 79%; run 3: 71%; average yield: 75%.

Diastereoselectivity determined by NMR analysis: 85:15 d.r.

Enantioselectivity of the major diastereomer determined by HPLC: >99:1 e.r.

Enantioselectivity of the minor diastereomer determined by HPLC: 97:3 e.r.

(2*S*,3*S*)-2-amino-3-(4-methoxyphenyl)-2-methylbutanoic acid (3e)

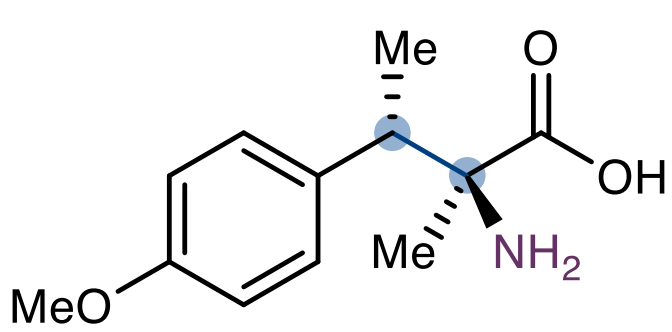

^{1}H NMR (400 MHz, D_2O) for major diastereomer (**3e**) δ: 7.45 – 7.12 (m, 2H), 7.11 – 6.83 (m, 2H), 3.83 (s, 3H), 3.42 (q, J = 7.4 Hz, 1H), 1.55 (s, 3H), 1.41 (d, J = 7.4 Hz, 3H) ppm. ^{13}C NMR (101 MHz, D_2O) for major diastereomer (**3e**) δ: 173.8, 158.6, 130.9, 130.0, 114.3, 63.8, 55.3, 43.5, 18.5, 13.9 ppm. IR: 3367, 3070, 2937, 1728, 1645, 1610, 1562, 1513, 1443, 1401, 1248, 1215, 1183, 1143, 1063, 1028, 999, 941, 836, 806, 744 cm^{-1}. HRMS (ESI) (m/z) for $[M+H]^+$ $C_{12}H_{18}NO_3$ requires 224.1287, observed 224.1282.

(2*S*,3*S*)-3e was prepared according to General Procedure A using *Tm*TA^{QAM} (0.5 mol%) derivatized using Fmoc-Cl and analyzed by chiral HPLC.

HPLC analysis conditions: CHIRALCEL IC column (4.6 mm × 25 cm, 5 micron), 95:5 hexanes: EtOH (0.1% TFA), flow rate = 1.0 mL/min, wavelength = 265 nm, 40 °C. t_R = 12.2 min (major), t_R = 14.5 min (minor), t_R = 19.3 min (minor), t_R = 21.8 min (major).

Yield with D-alanine: run 1: 71%; run 2: 83%; run 3: 73%; average yield: 76%.

Diastereoselectivity determined by NMR analysis: 88:12 d.r.

Enantioselectivity of the major diastereomer determined by HPLC: >99:1 e.r.

Enantioselectivity of the minor diastereomer determined by HPLC: >99:1 e.r.

(2*S*,3*S*)-2-amino-3-(4-fluorophenyl)-2-methylbutanoic acid (3f)

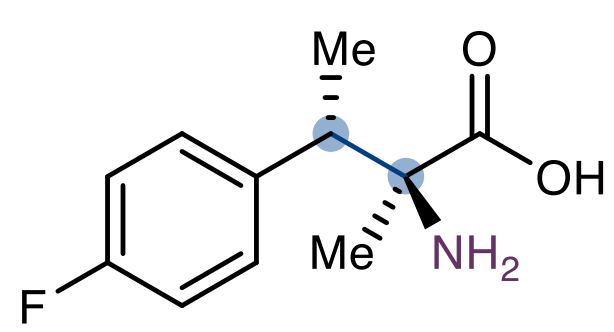

^{1}H NMR (400 MHz, D_2O) for major diastereomer (**3f**) δ: 7.39 – 7.30 (m, 2H), 7.23 – 7.10 (m, 2H), 3.47 (q, *J* = 7.4 Hz, 1H), 1.55 (s, 3H), 1.43 (d, *J* = 7.4 Hz, 3H) ppm. ^{13}C NMR (101 MHz, D_2O) for major diastereomer (**3f**) δ: 173.6, 162.2 (d, *J* = 244.3 Hz), 134.2 (d, *J* = 3.3 Hz), 130.5 (d, *J* = 8.3 Hz), 115.5 (d, *J* = 21.5 Hz), 63.7, 43.6, 18.6, 13.9 ppm. ^{19}F NMR (376 MHz, D_2O) for major diastereomer (**3e**) δ: -114.8 ppm. IR: 2929, 2186, 1741, 1654, 1605, 1510, 1455, 1414, 1383, 1223, 1191, 1161, 1131, 1066, 1016, 836, 796, 763, 738, 722, 702, 644 cm^{-1}. HRMS (ESI) (m/z) for $[M+H]^+$ $C_{11}H_{15}FNO_2$ requires 212.1087, observed 212.1083.

(2*S*,3*S*)-3f was prepared according to General Procedure A using *Tm*TA^{QAM} (0.5 mol%) derivatized using Fmoc-Cl and analyzed by chiral HPLC.

HPLC analysis conditions: CHIRALCEL IB N-5 column (4.6 mm × 25 cm, 5 micron), 97:3 hexanes: EtOH (0.1% TFA), flow rate = 1.0 mL/min, wavelength = 265 nm, 40 °C. t_R = 22.4 min (major), t_R = 37.8 min (minor).

Yield with D-alanine: run 1: 68%; run 2: 64%; run 3: 73%; average yield: 68%.

Diastereoselectivity determined by NMR analysis: 92:8 d.r.

Enantioselectivity of the major diastereomer determined by HPLC: >99:1 e.r.

(2*S*,3*S*)-2-amino-3-(4-chlorophenyl)-2-methylbutanoic acid (3g)

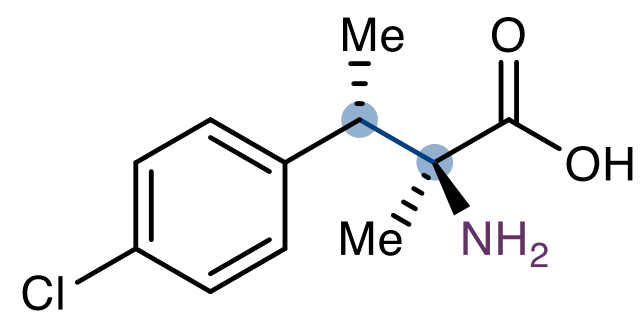

^{1}H NMR (400 MHz, D_2O) for major diastereomer (**3g**) δ: 7.50 – 7.41 (m, 2H), 7.31 (m, 2H), 3.45 (q, *J* = 7.3 Hz, 1H), 1.54 (s, 3H), 1.43 (d, *J* = 7.3, 3H) ppm. ^{13}C NMR (101 MHz, D_2O) for major diastereomer (**3g**) δ: 173.7, 137.1, 133.4, 130.3, 128.8, 128.8, 63.7, 43.8, 18.8, 13.7 ppm. IR: 2882, 1909, 1714, 1591, 1520, 1492, 1385, 1353, 1283, 1193, 1149, 1093, 1014, 870, 831, 718, 670 cm^{-1}. HRMS (ESI) (m/z) for $[M+H]^+$ $C_{11}H_{15}ClNO_2$ requires 228.0791, observed 212.0786.

(2*S*,3*S*)-3g was prepared according to General Procedure A using *Tm*TA^{QAM} (0.5 mol%) derivatized using Fmoc-Cl and analyzed by chiral HPLC.

HPLC analysis conditions: CHIRALCEL IC column (4.6 mm × 25 cm, 5 micron), 95:5 hexanes: EtOH (0.1% TFA), flow rate = 1.0 mL/min, wavelength = 265 nm, 40 °C. t_R = 8.0 min (major), t_R = 9.0 min (minor), t_R = 14.0 min (major), t_R = 15.4 min (minor).

Yield with D-alanine: run 1: 72%; run 2: 67%; run 3: 67%; average yield: 69%.

Diastereoselectivity determined by NMR analysis: 91:9 d.r.

Enantioselectivity of the major diastereomer determined by HPLC: >99:1 e.r.

Enantioselectivity of the minor diastereomer determined by HPLC: >99:1 e.r.

(2*S*,3*S*)-2-amino-3-(4-bromophenyl)-2-methylbutanoic acid (3h)

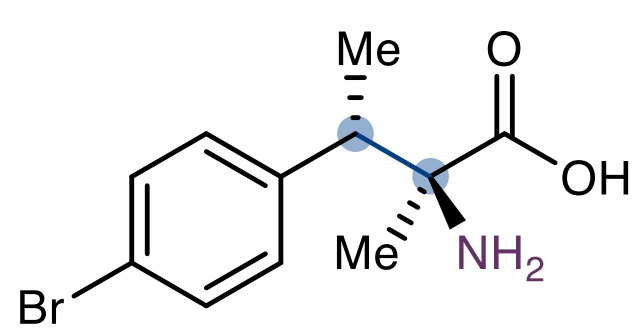

^{1}H NMR (400 MHz, D_2O) for major diastereomer (**3h**) δ: 7.56 (m, 2H), 7.22 (m, 2H), 3.42 (q, *J* = 7.3 Hz, 1H), 1.53 (s, 3H), 1.40 (d, *J* = 7.3 Hz, 3H) ppm. ^{13}C NMR (101 MHz, D_2O) for major diastereomer (**3h**) δ: 173.1, 137.4, 131.8, 130.6, 121.6, 63.3, 43.8, 18.7, 13.7 ppm. IR: 2890, 1735, 1489, 1078, 1010, 826 cm^{-1}. HRMS (ESI) (m/z) for $[M-H]^-$ $C_{11}H_{13}BrNO_2$ requires 270.0130, observed 270.0135.

(2*S*,3*S*)-3h was prepared according to General Procedure A using *Tm*TA^{QAM} (0.5 mol%) derivatized using Fmoc-Cl and analyzed by chiral HPLC.

HPLC analysis conditions: CHIRALCEL IC column (4.6 mm × 25 cm, 5 micron), 95:5 hexanes: EtOH (0.1% TFA), flow rate = 1.0 mL/min, wavelength = 265 nm, 40 °C. t_R = 9.7 min (major), t_R = 11.1 min (minor), t_R = 17.2 min (major), t_R = 18.8 min (minor).

Yield with D-alanine: run 1: 48%; run 2: 51%; run 3: 53%; average yield: 51%.

Diastereoselectivity determined by NMR analysis: 90:10 d.r.

Enantioselectivity of the major diastereomer determined by HPLC: >99:1 e.r.

Enantioselectivity of the minor diastereomer determined by HPLC: 98:2 e.r.

(2*S*,3*S*)-2-amino-2-methyl-3-(naphthalen-2-yl)butanoic acid (3i)

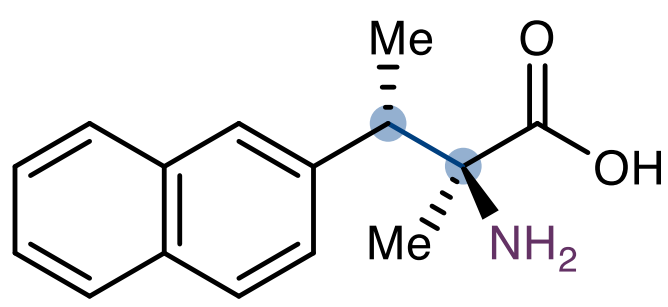

^{1}H NMR (400 MHz, D_2O) for major diastereomer (**3i**) δ: 7.97 – 7.90 (m, 3H), 7.83 (m, 1H), 7.64 – 7.54 (m, 2H), 7.44 (ddd, *J* = 7.2, 5.2, 1.8 Hz, 1H), 3.59 (q, *J* = 7.3 Hz, 1H), 1.59 (s, 3H), 1.52 (d, *J* = 7.3 Hz, 3H) ppm. ^{13}C NMR (101 MHz, D_2O) for major diastereomer (**3i**) δ: 173.9, 136.2, 135.9, 132.9, 132.6, 128.4, 127.9, 127.8, 127.6, 126.7, 126.6, 63.9, 44.4, 18.9, 13.9 ppm. IR: 3374, 2885, 1951, 1726, 1599, 1507, 1454, 1391, 1138, 1125, 949, 888, 857, 821, 745, 654, 623, 598, 511, 477 cm^{-1}. HRMS (ESI) (m/z) for $[M+Na]^+$ $C_{15}H_{17}NO_2Na$ requires 266.1157, observed 266.1159.

(2*S*,3*S*)-3i was prepared according to General Procedure A using *Tm*TA^{QAM} (0.5 mol%) derivatized using Fmoc-Cl and analyzed by chiral HPLC.

HPLC analysis conditions: CHIRALCEL IC column (4.6 mm × 25 cm, 5 micron), 95:5 hexanes: EtOH (0.1% TFA), flow rate = 1.0 mL/min, wavelength = 265 nm, 40 °C. t_R = 12.3 min (major), t_R = 17.3 min (minor), t_R = 19.5 min (minor), t_R = 22.5 min (major).

Yield with D-alanine: run 1: 42%; run 2: 44%; run 3: 47%; average yield: 45%.

Diastereoselectivity determined by NMR analysis: 75:25 d.r.

Enantioselectivity of the major diastereomer determined by HPLC: >99:1 e.r.

Enantioselectivity of the minor diastereomer determined by HPLC: 96:4 e.r.

(2*S*,3*S*)-2-amino-2-methyl-3-phenylpentanoic acid (3j)

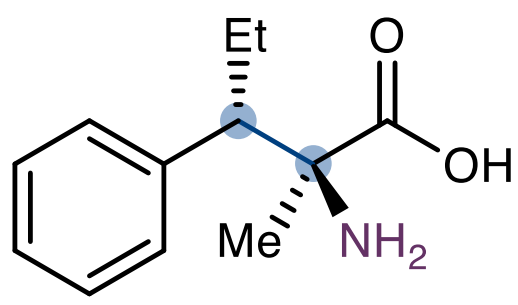

^{1}H NMR (400 MHz, D_2O) for major diastereomer (**3j**) δ: 7.45 (m, 3H), 7.35 (m, 2H), 3.15 (dd, *J* = 11.8, 4.0 Hz, 1H), 2.06 – 1.81 (m, 2H), 1.56 (s, 3H), 0.73 (t, *J* = 7.2 Hz, 3H) ppm. ^{13}C NMR (101 MHz, D_2O) for major diastereomer (**3j**) δ: 174.4, 136.7, 130.4, 129.7, 129.0, 64.2, 53.0, 21.2, 19.2, 12.1 ppm. IR: 2873, 2559, 1718, 1587, 1506, 1452, 1401, 1220, 1199, 1185, 1144, 849, 799, 754, 730, 695, 652 cm^{-1}. HRMS (ESI) (m/z) for $[M+H]^+$ $C_{12}H_{18}NO_2$ requires 208.1338, observed 208.1338.

(2*S*,3*S*)-3j was prepared according to General Procedure A using *Tm*TA^{QAM} (0.5 mol%) derivatized using Fmoc-Cl and analyzed by chiral HPLC.

HPLC analysis conditions: CHIRALCEL IC column (4.6 mm × 25 cm, 5 micron), 95:5 hexanes: EtOH (0.1% TFA), flow rate = 1.0 mL/min, wavelength = 265 nm, 40 °C. t_R = 7.9 min (major), t_R = 21.5 min (minor).

Yield with D-alanine: run 1: 59%; run 2: 59%; run 3: 54%; average yield: 57%.

Diastereoselectivity determined by NMR analysis: 89:11 d.r.

Enantioselectivity of the major diastereomer determined by HPLC: >99:1 e.r.

(2*S*,3*S*)-2-amino-2-methyl-3-phenylhexanoic acid (3k)

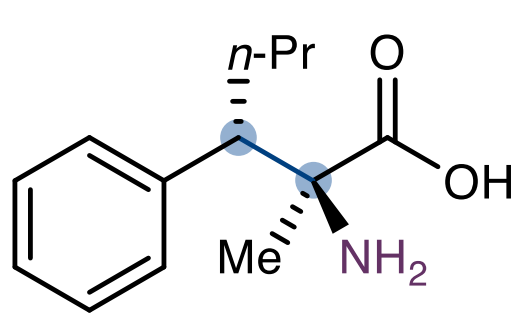

^{1}H NMR (400 MHz, D_2O) for major diastereomer (**3k**) δ: 7.50 – 7.38 (m, 3H), 7.38 – 7.29 (m, 2H), 3.25 (dd, *J* = 12.4, 3.4 Hz, 1H), 1.91 (tdd, *J* = 13.1, 9.0, 4.7 Hz, 1H), 1.78 (dddd, *J* = 13.1, 9.8, 6.9, 3.4 Hz, 1H), 1.56 (s, 3H), 1.21 – 0.99 (m, 2H), 0.82 (t, *J* = 7.4 Hz, 3H) ppm. ^{13}C NMR (101 MHz, D_2O) for major diastereomer (**3k**) δ: 173.7, 136.2, 129.6, 128.9, 128.3, 63.5, 50.1, 29.3, 19.9, 18.5, 12.7 ppm. IR: 2872, 1726, 1600, 1495, 1454, 1380, 1135, 800, 753, 730, 700, 654, 599, 515 cm^{-1}. HRMS (ESI) (m/z) for $[M+H]^+$ $C_{13}H_{20}NO_2$ requires 222.1494, observed 222.1504.

(2*S*,3*S*)-3k was prepared according to General Procedure A using *Tm*TA^{QAM} (0.5 mol%) derivatized using Fmoc-Cl and analyzed by chiral HPLC.

HPLC analysis conditions: CHIRALCEL IC column (4.6 mm × 25 cm, 5 micron), 97:3 hexanes: EtOH (0.1% TFA), flow rate = 1.0 mL/min, wavelength = 265 nm, 40 °C. t_R = 12.8 min (major), t_R = 16.6 min (minor), t_R = 26.6 min (major), t_R = 27.6 min (minor).

Yield with D-alanine: run 1: 51%; run 2: 53%; run 3: 61%; average yield: 55%.

Diastereoselectivity determined by NMR analysis: 85:15 d.r.

Enantioselectivity of the major diastereomer determined by HPLC: 91:9 e.r.

Enantioselectivity of the minor diastereomer determined by HPLC: 92:8 e.r.

(*S*)-2-amino-2-((*S*)-2,3-dihydro-1H-inden-1-yl)propanoic acid (3l)

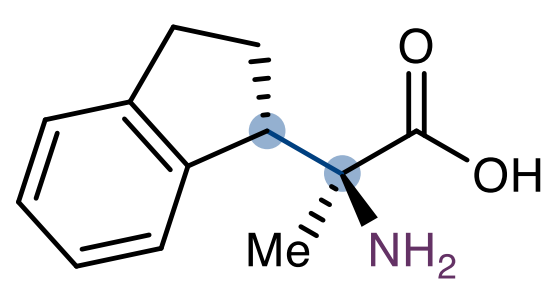

^{1}H NMR (400 MHz, D_2O) for major diastereomer (**3l**) δ: 7.41 – 7.30 (m, 2H), 7.29 – 7.20 (m, 2H), 3.88 (dd, *J* = 9.3, 4.4 Hz, 1H), 3.13 – 2.90 (m, 2H), 2.58 – 2.32 (m, 1H), 2.14 (ddt, *J* = 13.9, 7.7, 4.4 Hz, 1H), 1.69 (s, 3H) ppm. ^{13}C NMR (101 MHz, D_2O) for major diastereomer (**3l**) δ: 175.0, 146.2, 139.4, 129.3, 127.5, 126.0, 125.8, 64.2, 50.5, 31.1, 26.2, 21.6 ppm. IR: 3384, 2897, 2557, 1741, 1584, 1507, 1480, 1459, 1376, 1226, 1203, 1157, 1142, 1125, 833, 757, 653 cm^{-1}. HRMS (ESI) (m/z) for $[M+H]^+$ $C_{12}H_{16}NO_2$ requires 206.1176, observed 206.1171.

(2*S*,3*S*)-3l was prepared according to General Procedure A using *Tm*TA^{QAM} (0.5 mol%) derivatized using Fmoc-Cl and analyzed by chiral HPLC.

HPLC analysis conditions: CHIRALCEL IC column (4.6 mm × 25 cm, 5 micron), 95:5 hexanes: EtOH (0.1% TFA), flow rate = 1.0 mL/min, wavelength = 265 nm, 40 °C. t_R = 11.6 min (minor), t_R = 12.3 min (major), t_R = 15.5 min (major), t_R = 18.7 min (minor).

Yield with D-alanine: run 1: 78%; run 2: 71%; run 3: 73%; average yield: 74%.

Yield with D-alanine using *Tm*TA W86Q E88A F85M (0.1 mol%): run 1: 52%; run 2: 57%; run 3: 57%; average yield: 55%.

Diastereoselectivity determined by NMR analysis: 95:5 d.r.

Enantioselectivity of the major diastereomer determined by HPLC: >99:1 e.r.

Enantioselectivity of the minor diastereomer determined by HPLC: >99:1 e.r.

(*S*)-2-amino-2-((*S*)-1,2,3,4-tetrahydronaphthalen-1-yl)propanoic acid (3m)

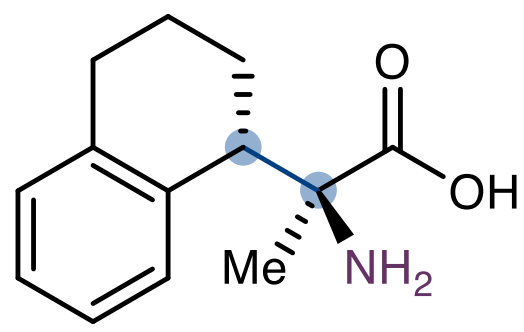

^{1}H NMR (400 MHz, D_2O) for major diastereomer (**3m**) δ: 7.32 – 7.26 (m, 1H), 7.23 (m, 3H), 3.73 (t, *J* = 7.7 Hz, 1H), 2.71 (dddd, *J* = 26.1, 16.3, 10.5, 5.1 Hz, 2H), 2.29 – 2.11 (m, 1H), 2.04 – 1.90 (m, 1H), 1.78 (dddd, *J* = 14.3, 11.3, 7.7, 4.1 Hz, 1H), 1.64 (s, 3H), 1.54 (ddt, *J* = 19.2, 9.9, 4.9 Hz, 1H) ppm. ^{13}C NMR (101 MHz, D_2O) for major diastereomer (**3m**) δ: 175.4, 140.9, 132.7, 129.7, 129.0, 127.6, 126.3, 64.6, 42.0, 29.2, 23.7, 20.9, 20.9 ppm. IR: 3368, 2906, 1744, 1586, 1505, 1452, 1369, 1218, 1191, 1125.29, 1057, 823, 805, 775, 753, 739, 652 cm^{-1}. HRMS (ESI) (m/z) for $[M+H]^+$ $C_{13}H_{18}NO_2$ requires 220.1338, observed 220.1340.

(2*S*,3*S*)-3m was prepared according to General Procedure A using *Tm*TA^{QAM} (0.5 mol%) derivatized using Fmoc-Cl and analyzed by chiral HPLC.

HPLC analysis conditions: CHIRALCEL IC column (4.6 mm × 25 cm, 5 micron), 95:5 hexanes: EtOH (0.1% TFA), flow rate = 1.0 mL/min, wavelength = 265 nm, 40 °C. t_R = 8.7 min (minor), t_R = 9.4 min (major), t_R = 10.8 min (major), t_R = 14.4 min (minor).

Yield with D-alanine: run 1: 75%; run 2: 81%; run 3: 85%; average yield: 80%.

Yield with D-alanine using *Tm*TA W86Q E88A F85M (0.1 mol%): run 1: 52%; run 2: 50%; run 3: 47%; average yield: 50%.

Diastereoselectivity determined by NMR analysis: 95:5 d.r.

Enantioselectivity of the major diastereomer determined by HPLC: >99:1 e.r.

Enantioselectivity of the minor diastereomer determined by HPLC: 99:1 e.r.

(*S*)-2-amino-2-((*S*)-chroman-4-yl)propanoic acid (3n)

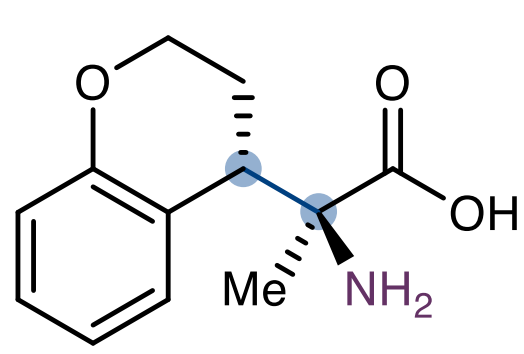

^{1}H NMR (400 MHz, D_2O) for major diastereomer (**3n**) δ: 7.34 – 7.24 (m, 1H), 7.19 (m, 1H), 7.05 – 6.96 (m, 1H), 6.92 (m, 1H), 4.32 (ddd, *J* = 11.5, 6.4, 4.3 Hz, 1H), 4.07 (ddd, *J* = 11.5, 8.5, 3.7 Hz, 1H), 3.77 (t, *J* = 7.4 Hz, 1H), 2.29 (dtd, *J* = 14.0, 6.8, 3.6 Hz, 1H), 2.21 – 2.09 (m, 1H), 1.67 (s, 3H) ppm. ^{13}C NMR (101 MHz, D_2O) for major diastereomer (**3n**) δ: 175.5, 156.5, 130.2, 129.8, 122.4, 120.4, 118.3, 65.3, 64.0, 38.9, 24.0, 21.9 ppm. IR: 2912, 1742, 1581, 1509, 1490, 1453, 1385, 1369, 1300, 1270, 1222, 1192, 1157, 1134, 1119, 1071, 1021, 981, 951, 855, 829, 755, 700,

660, 588, 522 cm^{-1}. HRMS (ESI) (m/z) for $[M+H]^+$ $C_{12}H_{16}NO_3$ requires 222.1130, observed 222.1134.

(2*S*,3*S*)-3n was prepared according to General Procedure A using *Tm*TA^{QAM} (0.5 mol%) derivatized using Fmoc-Cl and analyzed by chiral HPLC.

HPLC analysis conditions: CHIRALCEL IC column (4.6 mm × 25 cm, 5 micron), 98:2 hexanes: EtOH (0.1% TFA), flow rate = 1.0 mL/min, wavelength = 265 nm, 40 °C. t_R = 14.9 min (minor), t_R = 16.4 min (major), t_R = 17.1 min (major).

Yield with D-alanine: run 1: 51%; run 2: 58%; run 3: 55%; average yield: 55%.

Diastereoselectivity determined by NMR analysis: 95:5 d.r.

Enantioselectivity of the major diastereomer determined by HPLC: >99:1 e.r.

(2*S*,3*S*)-2-amino-2-ethyl-3-phenylbutanoic acid (3o)

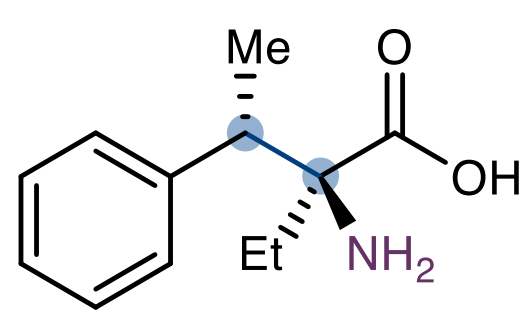

^{1}H NMR (400 MHz, D_2O) for major diastereomer (**3o**) δ: 7.46 – 7.38 (m, 1H), 7.36 (m, 1H), 3.52 (q, *J* = 7.4 Hz, 1H), 2.08 (dd, *J* = 15.0, 7.6 Hz, 1H), 1.93 (dq, *J* = 14.6, 7.3 Hz, 1H), 1.46 (d, *J* = 7.4 Hz, 3H), 0.98 (t, *J* = 7.5 Hz, 3H) ppm. ^{13}C NMR (101 MHz, D_2O) for major diastereomer (**3o**) δ: 173.2, 138.8, 128.9, 128.7, 128.2, 43.8, 26.3, 13.5, 7.4, 1.0 ppm. IR: 2882, 1719, 1584, 1497, 1454, 1393, 1206, 1138, 774, 702, 535 cm^{-1}. HRMS (ESI) (m/z) for $[M+H]^+$ $C_{12}H_{18}NO_2$ requires 208.1338, observed 208.1337.

(2*S*,3*S*)-3o was prepared according to General Procedure A using *Tm*TA E88T H83F (1.0 mol%) derivatized using Fmoc-Cl and analyzed by chiral HPLC.

HPLC analysis conditions: CHIRALCEL IA column (4.6 mm × 25 cm, 5 micron), 99:1 hexanes: iPrOH (0.1% TFA), flow rate = 1.0 mL/min, wavelength = 265 nm, 40 °C. t_R = 25.0 min (major), t_R = 26.8 min (minor), t_R = 31.0 min (minor), t_R = 32.8 min (major).

Yield with (*S*)-2-aminobutanoic acid: run 1: 13%; run 2: 17%; run 3: 17%; average yield: 16%.

Diastereoselectivity determined by NMR analysis: 89:11 d.r.

The ratio of C-alkylation to N-alkylation determined by NMR analysis: 83:17 r.r.

Enantioselectivity of the major diastereomer determined by HPLC: 97:3 e.r.

Enantioselectivity of the minor diastereomer determined by HPLC: 94:6 e.r.

L-phenylalanine (4a)

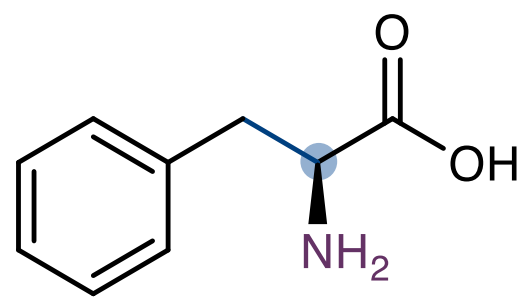

L-4a is a known compound. Spectral data match those reported previously[1-2].

L-4a was prepared according to General Procedure A using *Tm*TA^{QAM} (0.02 mol%) and derivatized using (*R*)-FDAA and analyzed by LC-MS.

Yield with glycine: run 1: 53.1%; run 2: 50.0%; run 3: 52.6%; average yield: 51.9%.

Enantioselectivity was determined by LC-MS: run 1: 99:1 e.r.; run 2: 98.5:1.5 e.r.; run 3: 98.5:1.5 e.r.; average e.r.: 98.5:1.5.

L-2-amino-3-(o-tolyl)propanoic acid (4b)

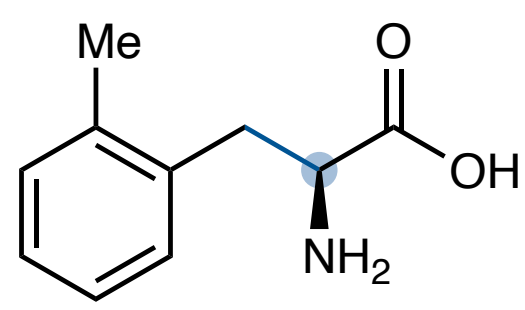

L-4a is a known compound. Spectral data match those reported previously [1-2]. **L-4b** was prepared according to General Procedure A using *Tm*TA^{QAM} (0.02 mol%) and derivatized using (*R*)-FDAA and analyzed by LC-MS.

Yield: run 1: 49.6%; run 2: 49.0%; run 3: 48.5%; average yield: 49%.

Enantioselectivity: run 1: 98:2 e.r.; run 2: 98:2 e.r.; run 3: 98:2 e.r.; average e.r.: 98:2.

L-2-amino-3-(m-tolyl)propanoic acid (4c)

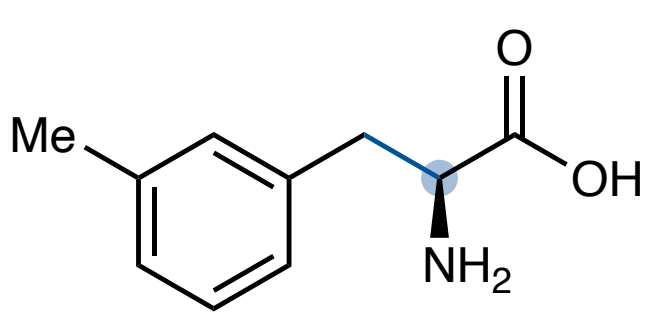

L-4c is a known compound. Spectral data match those reported previously[1-2]. **L-4c** was prepared according to General Procedure A using *Tm*TA^{QAM} (0.02 mol%) and derivatized using (*R*)-FDAA and analyzed by LC-MS.

Yield: run 1: 57.8%; run 2: 56.7%; run 3: 58.7%; average yield: 57.7%.

Enantioselectivity: run 1: 97.5:2.5 e.r.; run 2: 97:3 e.r.; run 3: 97.5:2.5 e.r.; average e.r.: 97.5:2.5.

L-2-amino-3-(p-tolyl)propanoic acid (4d)

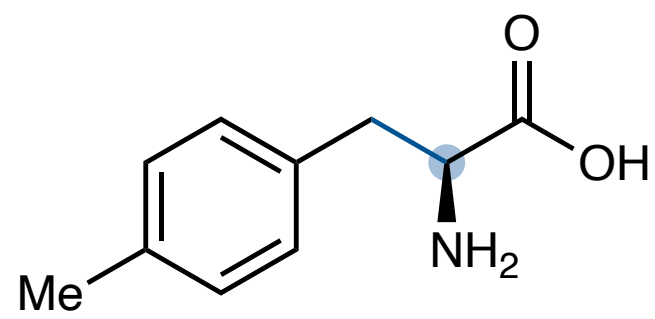

L-4d is a known compound. Spectral data match those reported previously[1-2].

L-4d was prepared according to General Procedure A using *Tm*TA[QAM] (0.02 mol%) and derivatized using (*R*)-FDAA and analyzed by LC-MS.

Yield: run 1: 41.9%; run 2: 44.0%; run 3: 45.8%; average yield: 43.9%.

Enantioselectivity: run 1: 96:4 e.r.; run 2: 95:5 e.r.; run 3: 95:5 e.r.; average e.r.: 95:5.

L-2-amino-3-(4-methoxyphenyl)propanoic acid (4e)

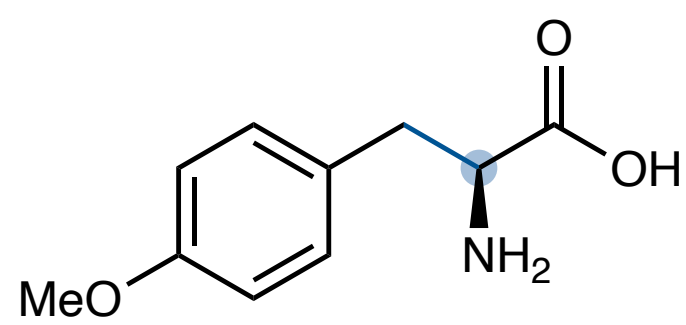

L-4e is a known compound. Spectral data match those reported previously[1-2].

L-4e was prepared according to General Procedure A using *Tm*TA[QAM] (0.02 mol%) and derivatized using (*R*)-FDAA and analyzed by LC-MS.

Yield: run 1: 37.0%; run 2: 39.5%; run 3: 37.8%; average yield: 38.1%.

Enantioselectivity: run 1: 98:2 e.r.; run 2: 98:2 e.r.; run 3: 97.5:2.5 e.r.; average e.r.: 97:3.

L-2-amino-3-(4-(trifluoromethyl)phenyl)propanoic acid (4f)

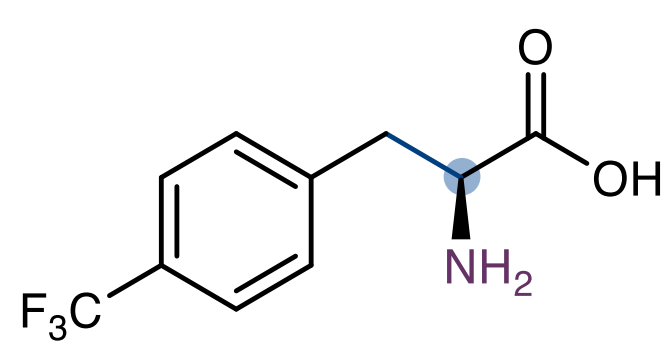

L-4f is a known compound. Spectral data match those reported previously[1-2].

L-4f was prepared according to General Procedure A using *Tm*TA[QAM] (0.02 mol%) and derivatized using (*R*)-FDAA and analyzed by LC-MS.

Yield with glycine: run 1: 26.5%; run 2: 23.6%; run 3: 25.5%; average yield: 25.2%.

Enantioselectivity was determined by LC-MS: run 1: 98:2 e.r.; run 2: 98.5:1.5 e.r.; run 3: 99:1 e.r.; average e.r.: 98.5:1.5.

L-2-amino-3-(naphthalen-2-yl)propanoic acid (4g)

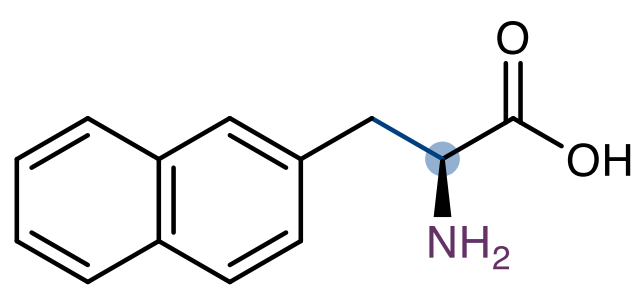

L-4g is a known compound. Spectral data match those reported previously[1-2].

L-4g was prepared according to General Procedure A using *Tm*TA^{QAM} (0.02 mol%) and derivatized using (*R*)-FDAA and analyzed by LC-MS.

Yield: run 1: 25.8%; run 2: 25.0%; run 3: 25.8%; average yield: 25.5%.

Enantioselectivity: run 1: 97:3 e.r.; run 2: 96:4 e.r.; run 3: 97:3 e.r.; average e.r.: 97:3.

(*S*)-2-amino-2-methyl-3-phenylpropanoic acid (5a)

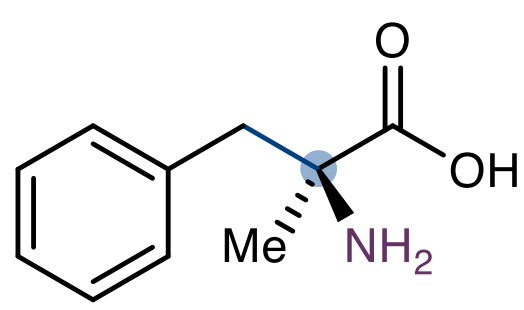

(*S*)-5a a known compound. Spectral data match those reported previously[1-2].

(*S*)-5a was prepared according to General Procedure A using *Tm*TA^{QAM} (0.05 mol%) derivatized using Fmoc-Cl and analyzed by chiral HPLC.

HPLC analysis conditions: CHIRALCEL IG column (4.6 mm × 25 cm, 5 micron), 97:3 hexanes: *i*-PrOH (0.1% TFA), flow rate = 1.0 mL/min, wavelength = 265 nm, 40 °C. t_R = 23.3 min (major).

Yield with D-alanine: run 1: 48%; run 2: 46%; run 3: 43%; average yield: 46%.

Enantioselectivity was determined by HPLC: >99:1 e.r.

(*S*)-2-amino-3-(4-fluorophenyl)-2-methylpropanoic acid (5b)

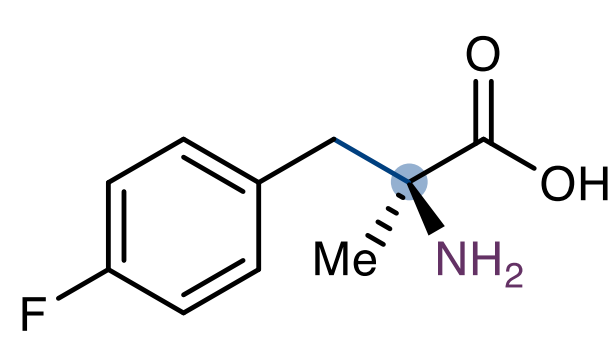

(*S*)-5b a known compound. Spectral data match those reported previously[1-2].

(*S*)-5b was prepared according to General Procedure A using *Tm*TA^{QAM} (0.05 mol%) derivatized using Fmoc-Cl and analyzed by chiral HPLC.

HPLC analysis conditions: CHIRALCEL IG column (4.6 mm × 25 cm, 5 micron), 97:3 hexanes: *i*-PrOH (0.1% TFA), flow rate = 1.0 mL/min, wavelength = 265 nm, 40 °C. t_R = 23.3 min (major).

Yield with D-alanine: run 1: 43%; run 2: 37%; run 3: 40%; average yield: 40 %.

Enantioselectivity was determined by HPLC: >99:1 e.r.

(*S*)-2-amino-3-(4-methoxyphenyl)-2-methylpropanoic acid (5c)

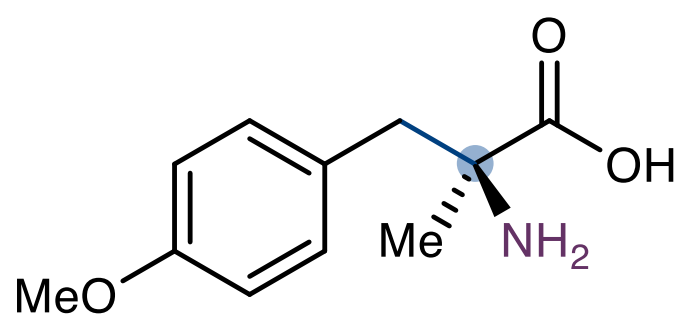

(*S*)-5c a known compound. Spectral data match those reported previously[1-2].

(*S*)-5c was prepared according to General Procedure A using *Tm*TA^{QAM} (0.05 mol%) derivatized using Fmoc-Cl and analyzed by chiral HPLC.

HPLC analysis conditions: CHIRALCEL IG column (4.6 mm × 25 cm, 5 micron), 97:3 hexanes: *i*-PrOH (0.1% TFA), flow rate = 1.0 mL/min, wavelength = 265 nm, 40 °C. t_R = 23.3 min (major).

Yield with D-alanine: run 1: 39%; run 2: 43%; run 3: 43%; average yield: 42%.

Enantioselectivity was determined by HPLC: >99:1 e.r.

(*S*,*E*)-2-amino-2-methyl-6-phenylhex-5-enoic acid (7a)

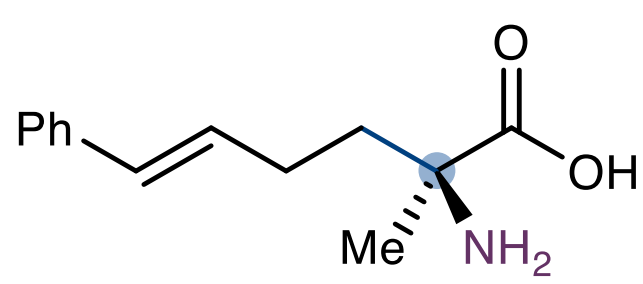

^{1}H NMR (400 MHz, D_2O) δ: 7.46 (m, 2H), 7.40 (m, 2H), 7.31 (m, 1H), 6.56 (d, *J* = 16.0 Hz, 1H), 6.32 (dt, *J* = 16.0, 6.7 Hz, 1H), 2.41 (ddd, *J* = 16.9, 12.1, 6.0 Hz, 1H), 2.28 (dq, *J* = 14.2, 6.7 Hz, 1H), 2.18 (ddd, *J* = 14.4, 10.9, 5.6 Hz, 1H), 2.07 (ddd, *J* = 14.4, 10.8, 5.4 Hz, 1H), 1.64 (s, 3H) ppm. ^{13}C NMR (101 MHz, D_2O) δ: 174.0, 137.2, 130.8, 128.9, 128.6, 127.5, 126.0, 59.9, 36.2, 26.6, 21.8 ppm. IR: 2959, 2197, 1742, 1440, 1406, 1377, 1195, 960, 740, 687 cm^{-1}. HRMS (ESI) (m/z) for $[M+Na]^+$ $C_{13}H_{17}NO_2Na$ requires 242.1157, observed 242.1162.

(*S*, *E*)-7awas prepared according to General Procedure A using *Tm*TA^{QAM} (1.0 mol%) derivatized using Fmoc-Cl and analyzed by chiral HPLC.

HPLC analysis conditions: CHIRALCEL IF column (4.6 mm × 25 cm, 5 micron), 95:5 hexanes: EtOH (0.1% TFA), flow rate = 1.0 mL/min, wavelength = 265 nm, 40 °C. t_R = 9.5 min (major), t_R = 11.7 min (minor).

Yield with D-alanine: run 1: 8%; run 2: 11%; run 3: 12%; average yield: 10%.

Enantioselectivity was determined by HPLC: >99:1 e.r.

***anti*-Methyl-2-benzamido-2-methyl-3-phenylpent-4-enoate (S9)**

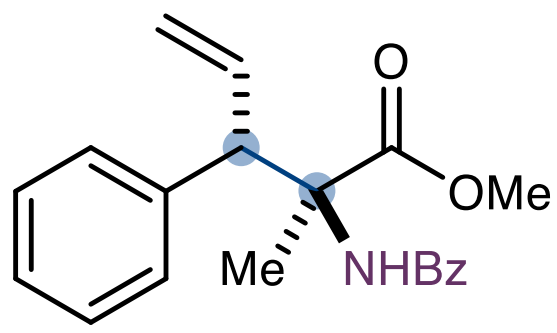

S9

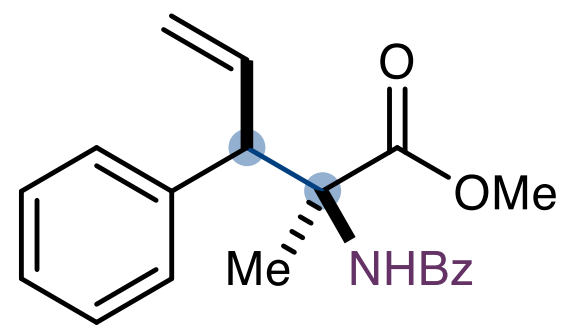

S9-*dia*

^{1}H NMR (400 MHz, $CDCl_3$) for major diastereomer (**S9**) δ: 7.70 – 7.62 (m, 2H), 7.54 – 7.45 (m, 1H), 7.44 – 7.35 (m, 4H), 7.34 – 7.31 (m, 1H), 7.29 – 7.26 (m, 2H), 6.69 (s, 1H), 6.36 (dt, *J* = 16.8, 9.6 Hz, 1H), 5.38 – 5.09 (m, 2H), 3.89 (d, *J* = 9.6 Hz, 1H), 3.72 (s, 3H), 1.71 (s, 3H) ppm. ^{13}C NMR (101 MHz, $CDCl_3$) for major diastereomer (**S9**) δ: 173.2, 166.4, 138.5, 135.6, 134.5, 131.8, 129.2, 128.9, 128.7, 128.0, 127.0, 119.3, 62.7, 57.8, 52.5, 19.7 ppm. ^{1}H NMR (400 MHz, $CDCl_3$) for minor diastereomer (**S9-*dia***) δ: 7.71 – 7.64 (m, 2H), 7.53 – 7.46 (m, 1H), 7.44 – 7.38 (m, 2H), 7.26 (m, 3H), 7.16 – 7.10 (m, 2H), 6.91 (s, 1H), 6.44 (dt, *J* = 16.8, 10.1 Hz, 1H), 5.72 – 5.10 (m, 2H), 4.20 (d, *J* = 10.1 Hz, 1H), 3.72 (s, 3H), 1.75 (s, 3H) ppm. ^{13}C NMR (101 MHz, $CDCl_3$) for minor diastereomer (**S9-*dia***) δ: 173.3, 166.4, 138.6, 136.1, 134.8, 131.7, 128.7, 128.6, 128.6, 127.6, 126.9, 119.8, 63.2, 56.2, 52.6, 19.6. ppm. Spectra was matched previously[19].

anti-2-Amino-3-cyclopropyl-2-methyl-3-phenylpropanoic acid (7b)

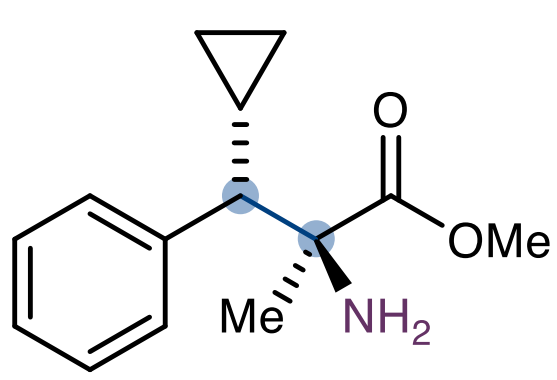

7b

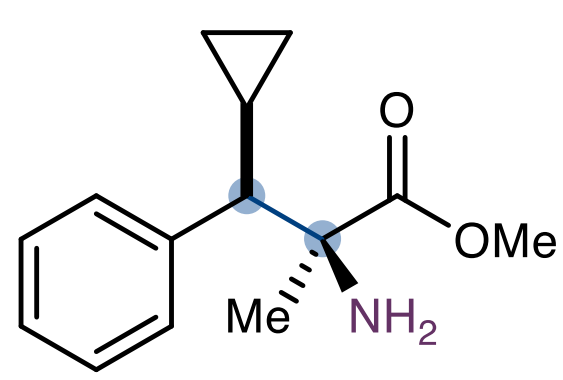

7b-*dia*

^{1}H NMR (400 MHz, D_2O) for major diastereomer (**7b**) δ: 7.69 – 7.27 (m, 5H), 2.51 (d, *J* = 11.0 Hz, 1H), 1.66 (s, 3H), 1.41 (dq, *J* = 12.4, 6.3 Hz, 1H), 0.89 (q, *J* = 9.4 Hz, 1H), 0.61 – 0.48 (m, 2H), -0.03 (t, *J* = 5.9 Hz, 1H) ppm. ^{13}C NMR (101 MHz, D_2O) for major diastereomer (**7a**) δ: 174.0, 138.2, 129.3, 128.7, 128.1, 64.2, 55.3, 19.1, 10.4, 8.0, 2.5 ppm. ^{1}H NMR (400 MHz, D_2O) for minor diastereomer (**7b-*dia***) δ: 7.39 (m, 5H), 2.21 (d, *J* = 10.8 Hz, 1H), 1.37 (d, *J* = 14.9 Hz, 1H), 1.11 (s, 3H), 0.70 (q, *J* = 7.4 Hz, 1H), 0.51 – 0.26 (m, 2H), -0.15 (dd, *J* = 9.9, 5.3 Hz, 1H) ppm. ^{13}C NMR (101 MHz, D_2O) for minor diastereomer (**7b-*dia***) δ: 176.1, 140.4, 131.8, 131.4, 130.7, 66.4, 57.9, 22.5, 13.2, 10.1, 5.3 ppm. IR: 3381, 3305, 1574, 1536, 1454, 1397, 1282, 922, 797, 596 cm^{-1}. HRMS (ESI) (m/z) for $[M-H]^-$ $C_{13}H_{16}NO_2$ requires 218.1181, observed 218.1172.

(*S*,*S*)-7bwas prepared according to General Procedure A using *Tm*TA^{QAM} (1.0 mol%) derivatized using Fmoc-Cl and analyzed by chiral HPLC.

Yield with D-alanine: run 1: 2%; run 2: 2%; run 3: 2%; average yield: 2%.

XI. HPLC calibration curves of amino acid products

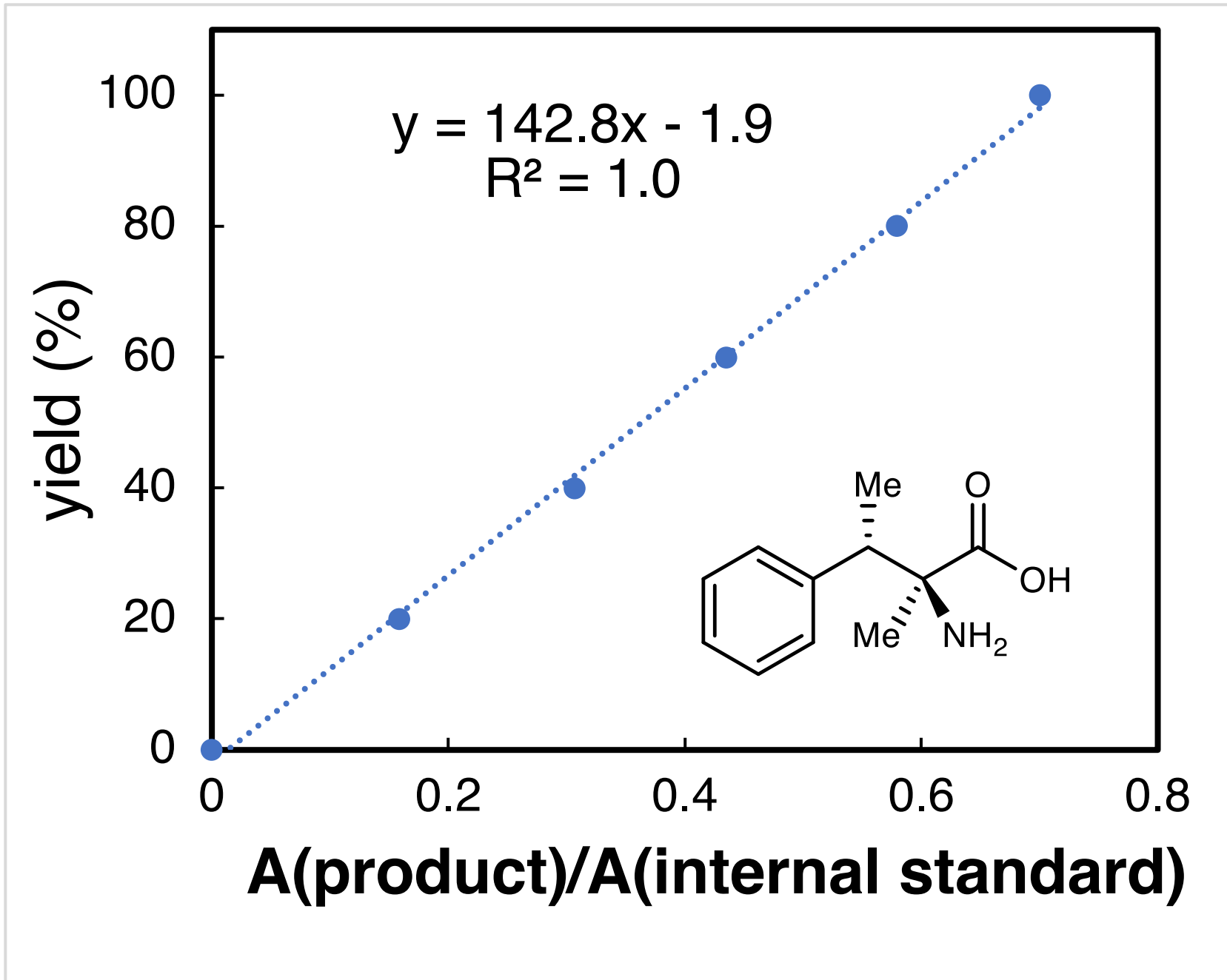

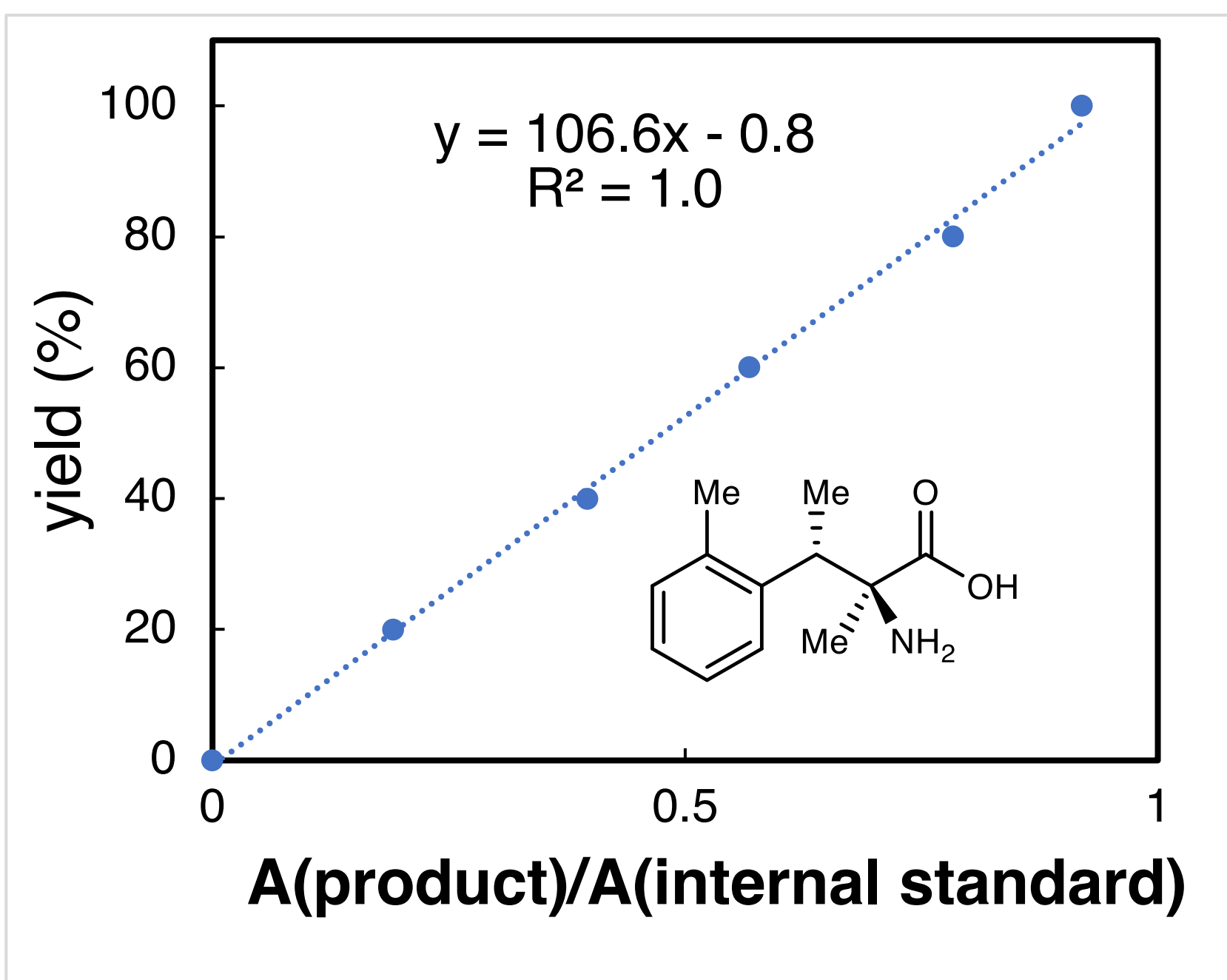

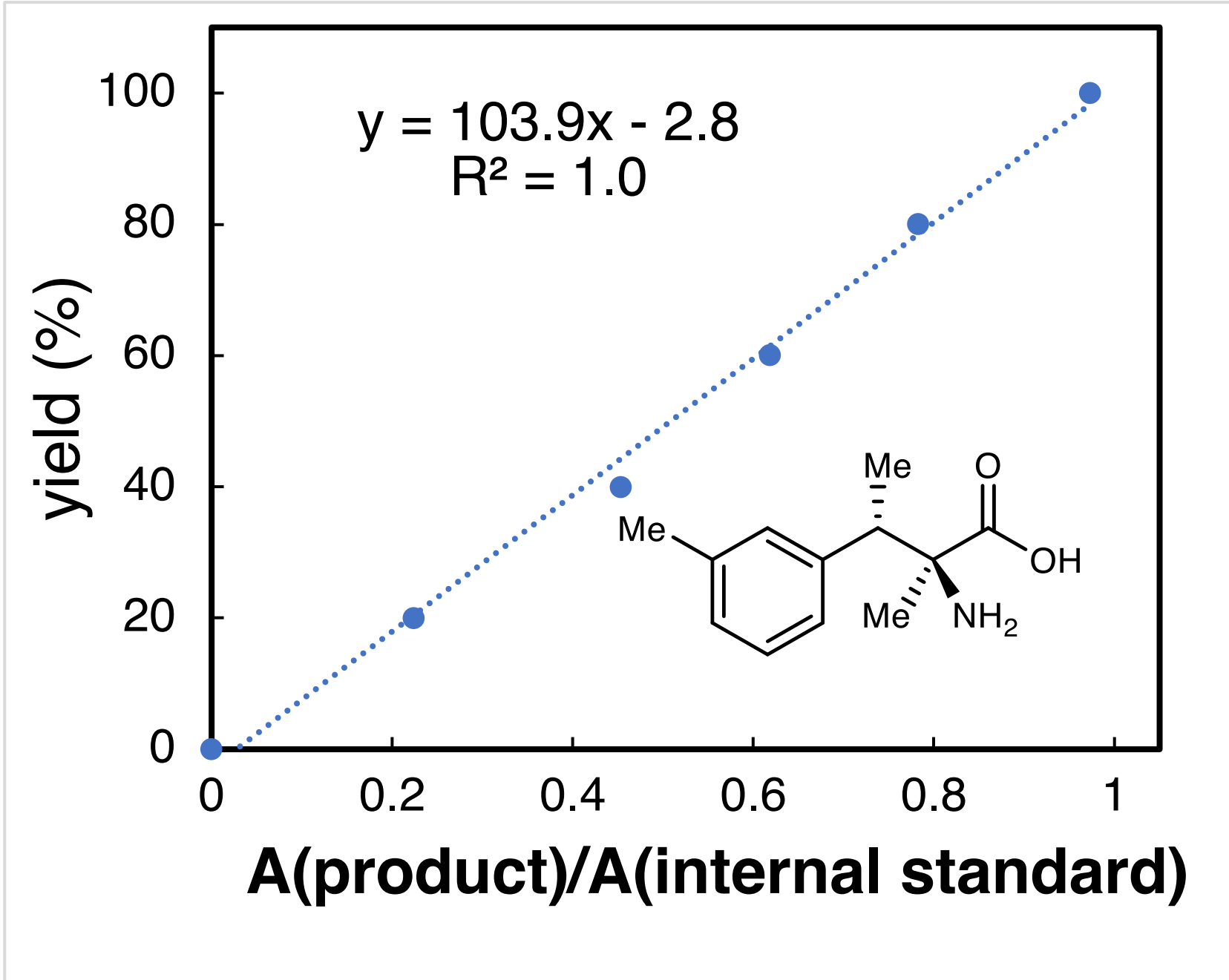

y = 103.9x - 2.8
R² = 1.0
yield (%)
100
80
60
40
20
0
0
0.2
0.4
0.6
0.8
1
A(product)/A(internal standard)
Me
Me
O
OH
Me
NH2

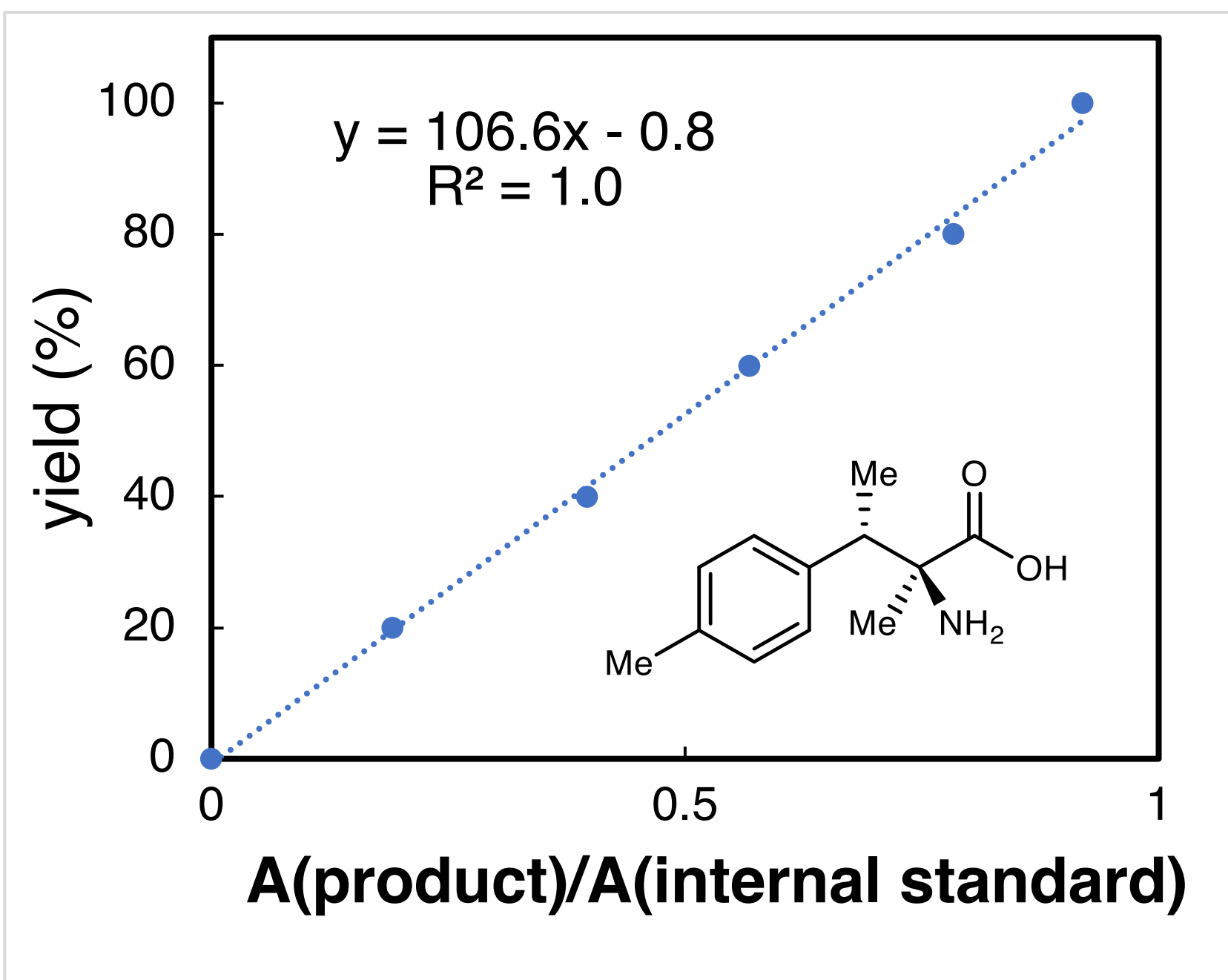

y = 106.6x - 0.8
R² = 1.0
yield (%)
100
80
60
40
20
0
0
0.5
1
A(product)/A(internal standard)
Me
O
OH
Me
NH2
Me

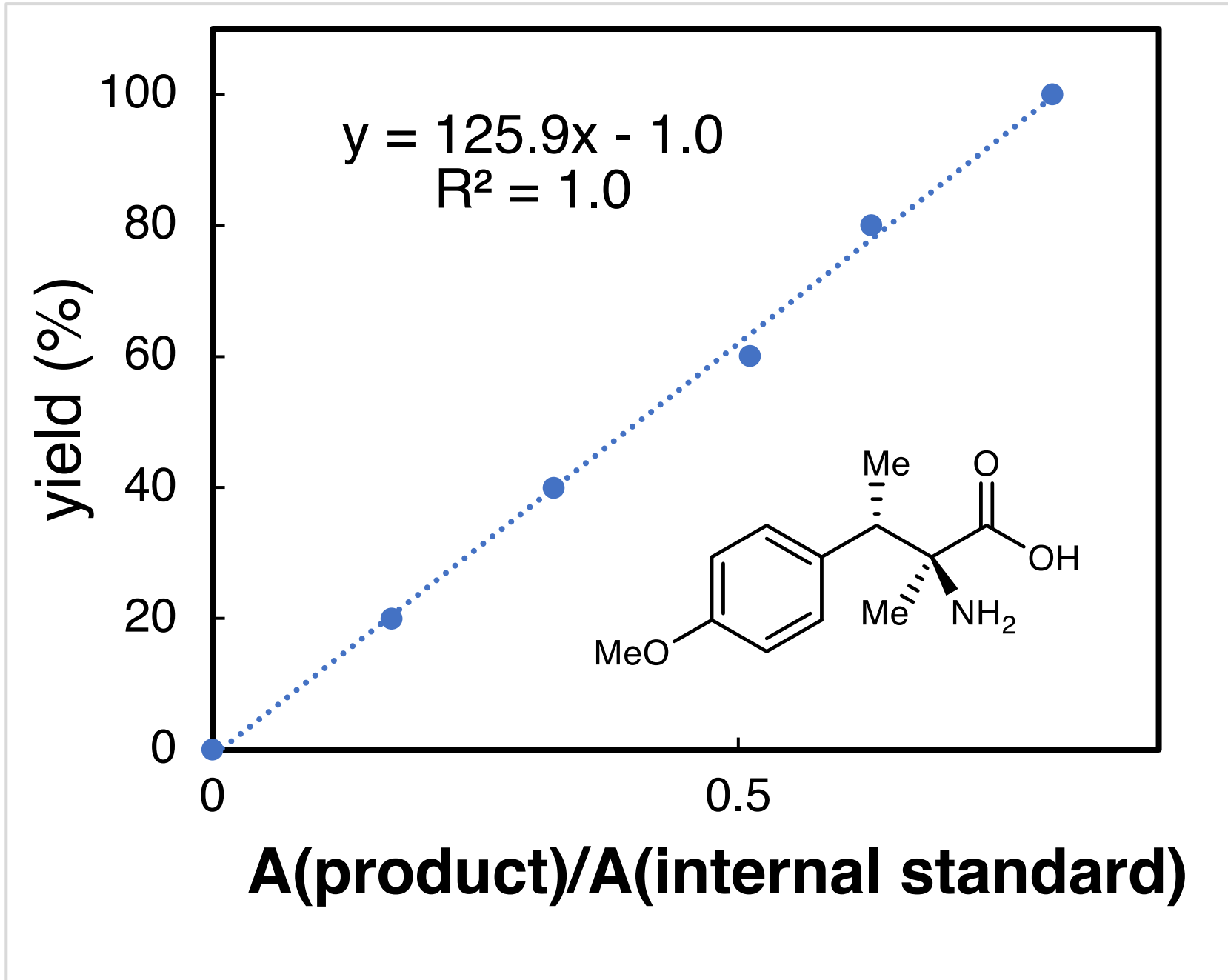

y = 125.9x - 1.0
R² = 1.0
100
80
60
40
20
0
0
0.5
yield (%)
A(product)/A(internal standard)
Me
O
OH
Me
NH2
MeO

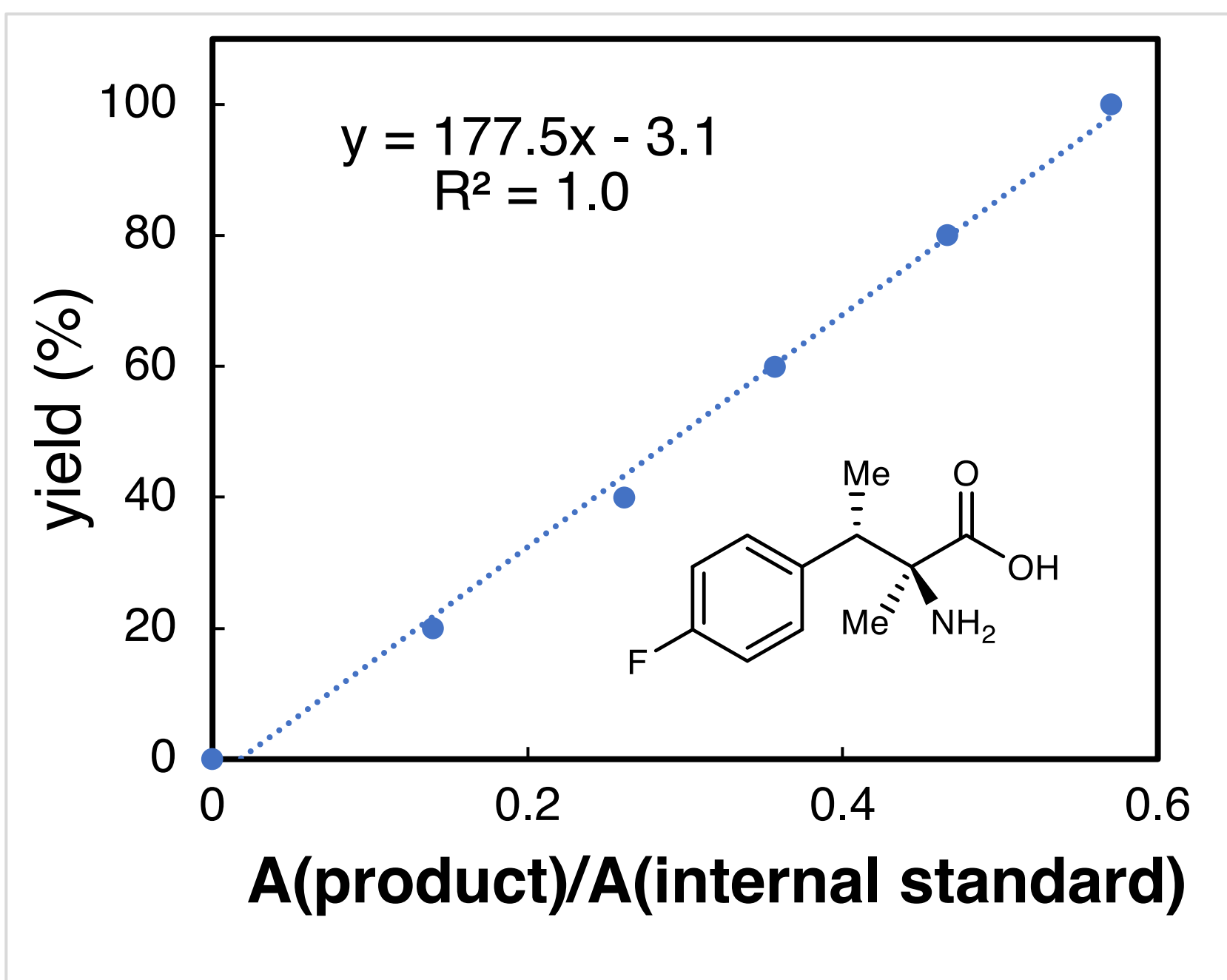

y = 177.5x - 3.1
R² = 1.0
100
80
60
40
20
0
0
0.2
0.4
0.6
yield (%)
A(product)/A(internal standard)
Me
O
OH
Me
NH2
F

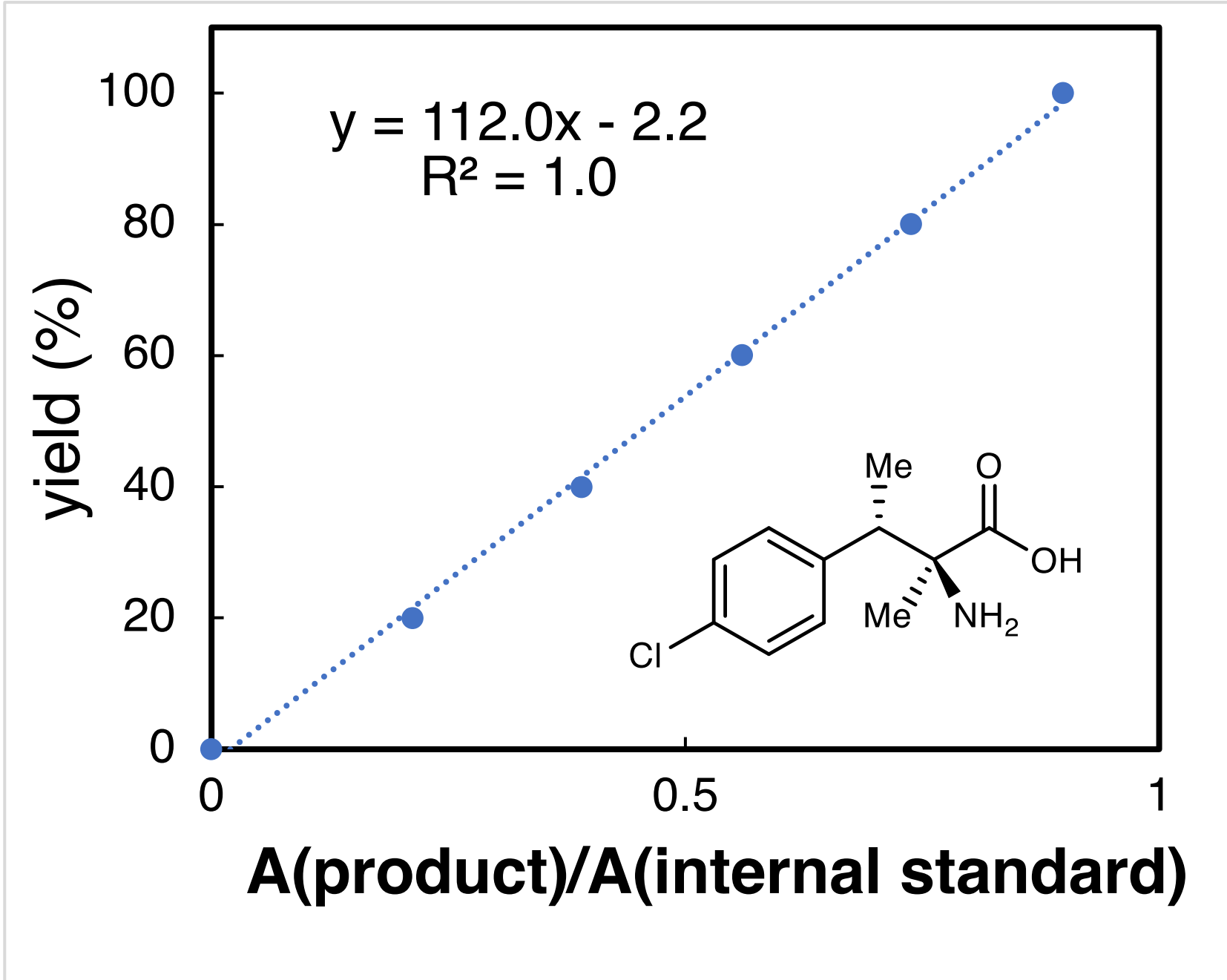

y = 112.0x - 2.2
R² = 1.0
yield (%)
A(product)/A(internal standard)
100
80
60
40
20
0
0
0.5
1
Me
O
OH
Me
NH2
Cl

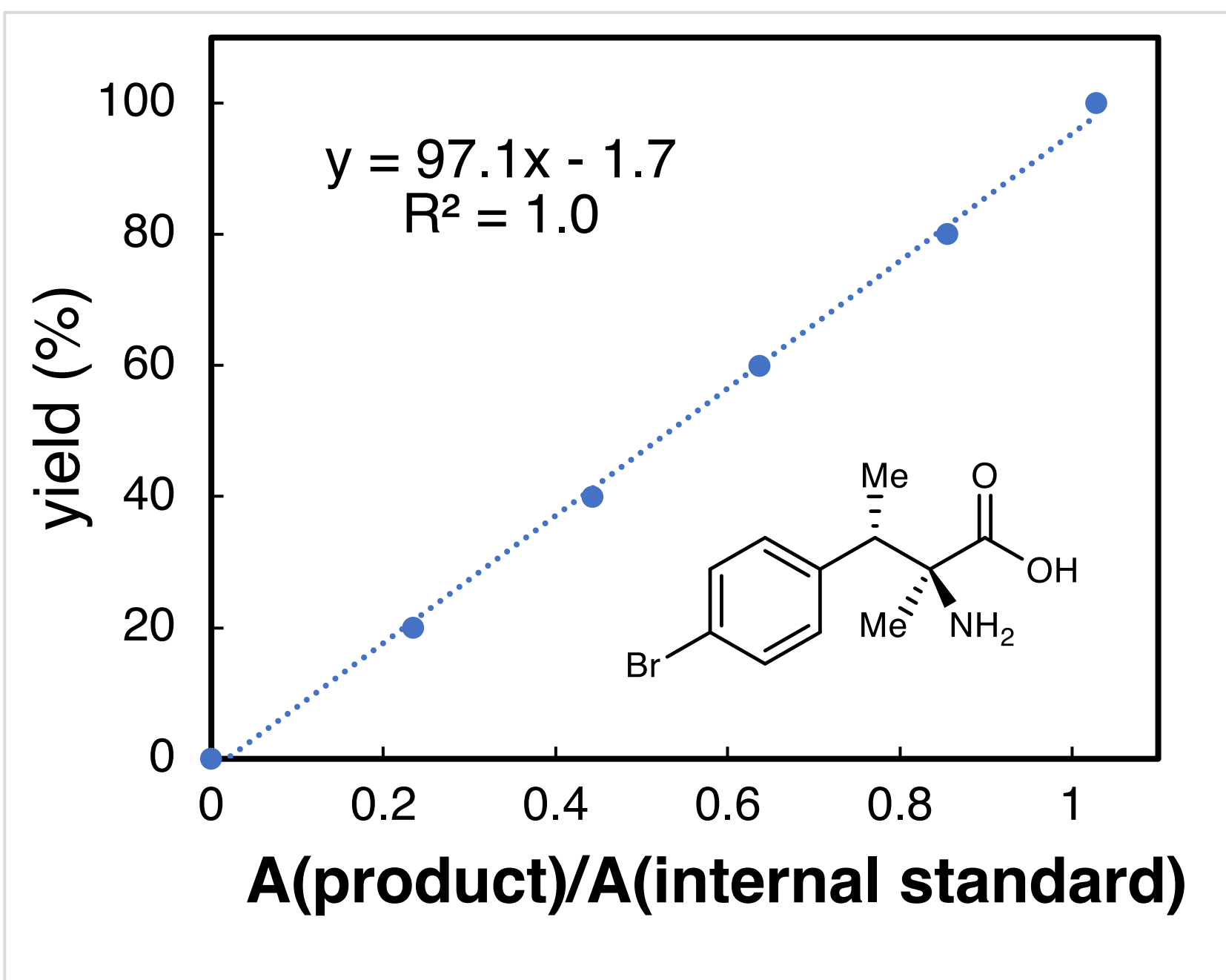

y = 97.1x - 1.7
R² = 1.0
yield (%)
A(product)/A(internal standard)
100
80
60
40
20
0
0
0.2
0.4
0.6
0.8
1
Me
O
OH
Me
NH2
Br

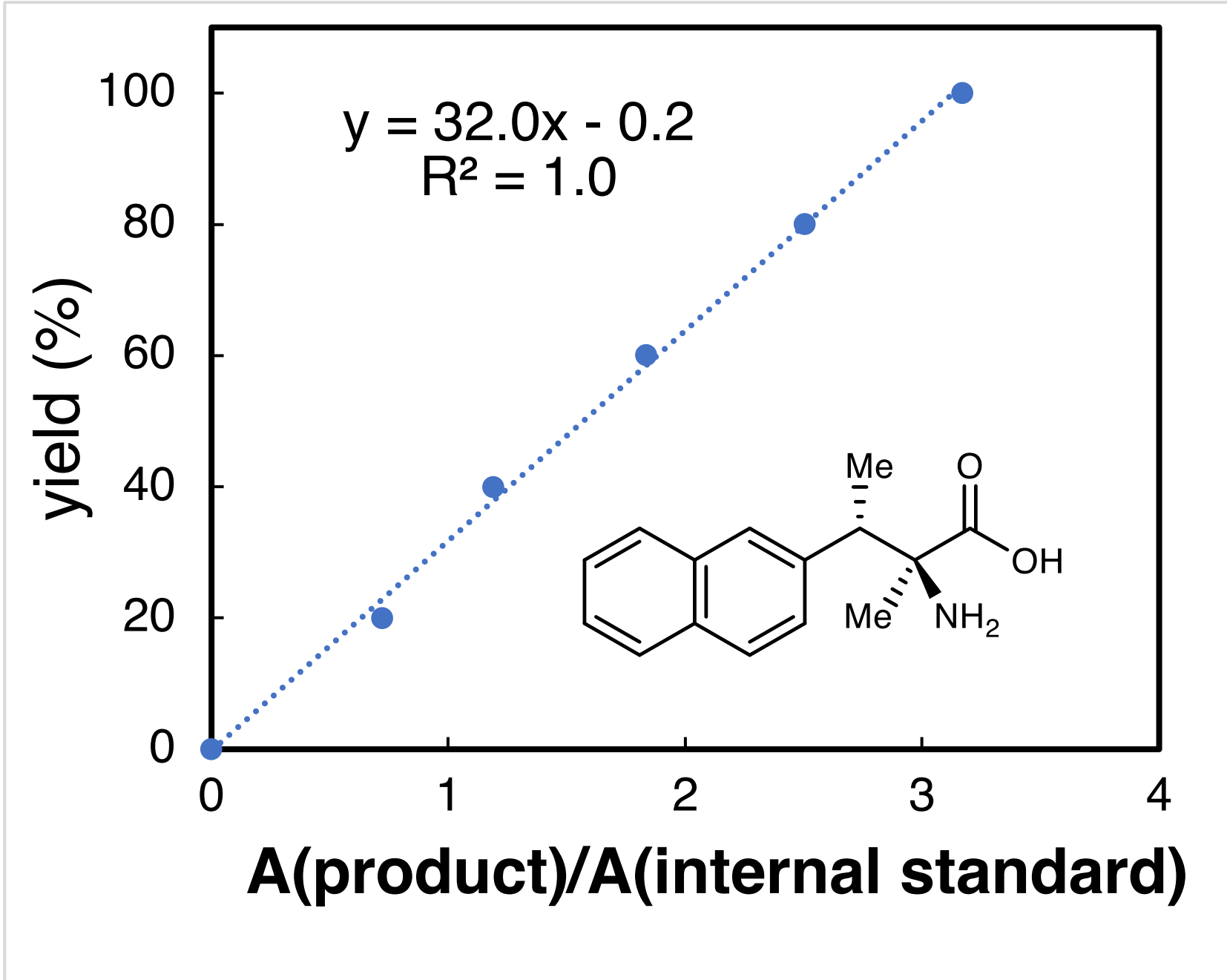
y = 32.0x - 0.2
R² = 1.0
yield (%)
A(product)/A(internal standard)
Me
O
OH
Me
NH2

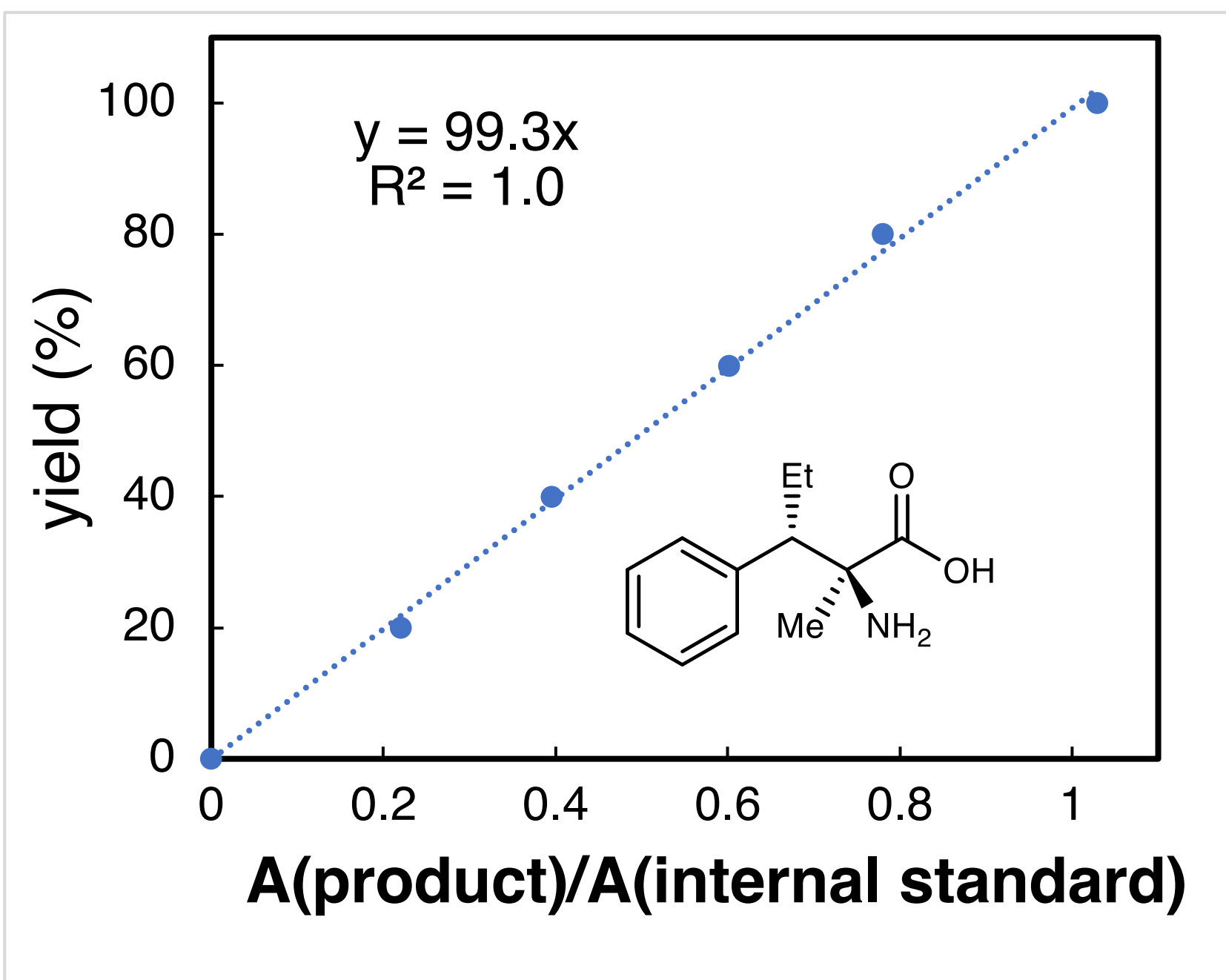
y = 99.3x
R² = 1.0
yield (%)
A(product)/A(internal standard)
Et
O
OH
Me
NH2

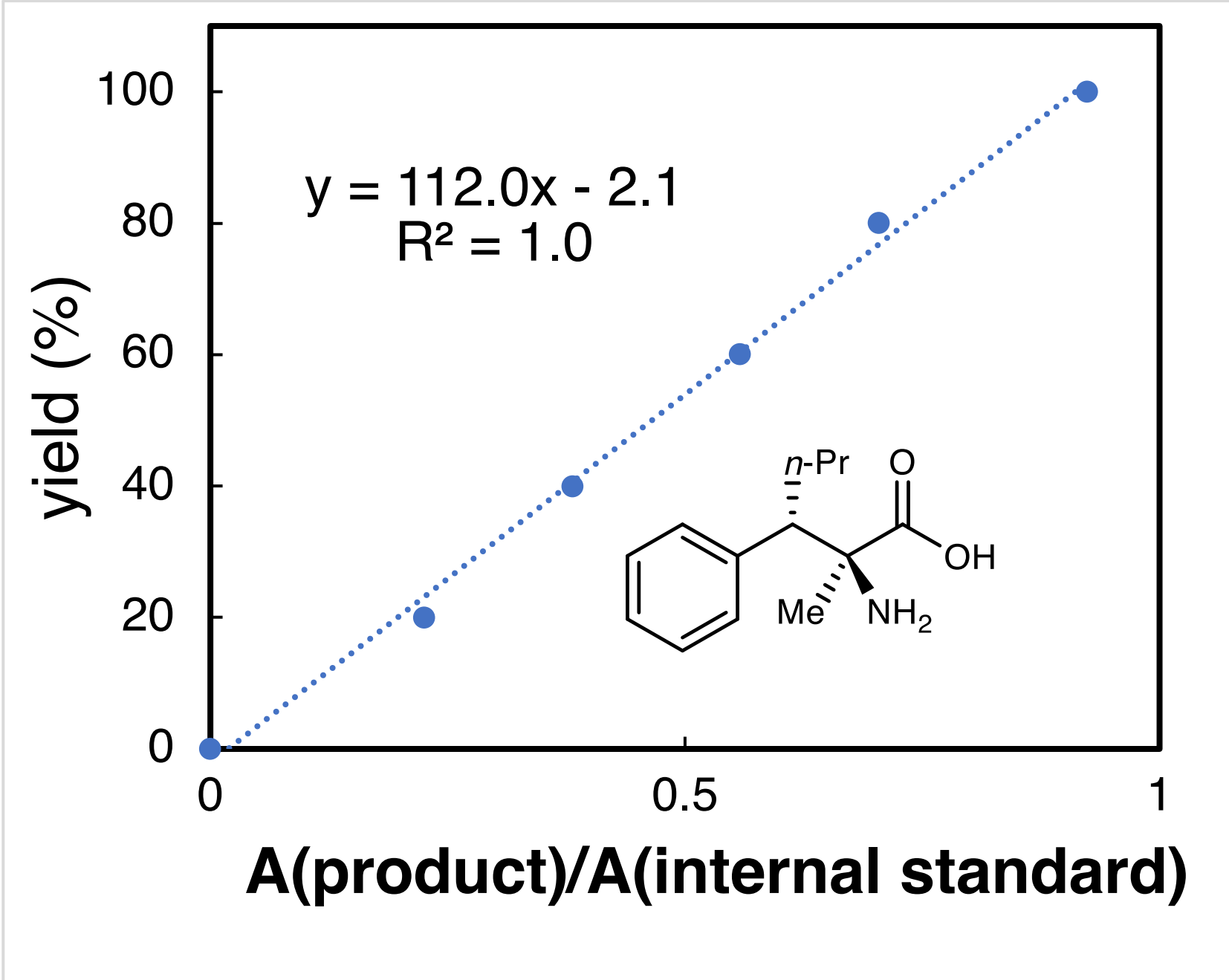
y = 112.0x - 2.1
R² = 1.0
n-Pr
O
OH
Me
NH2
yield (%)
A(product)/A(internal standard)
100
80
60
40
20
0
0
0.5
1

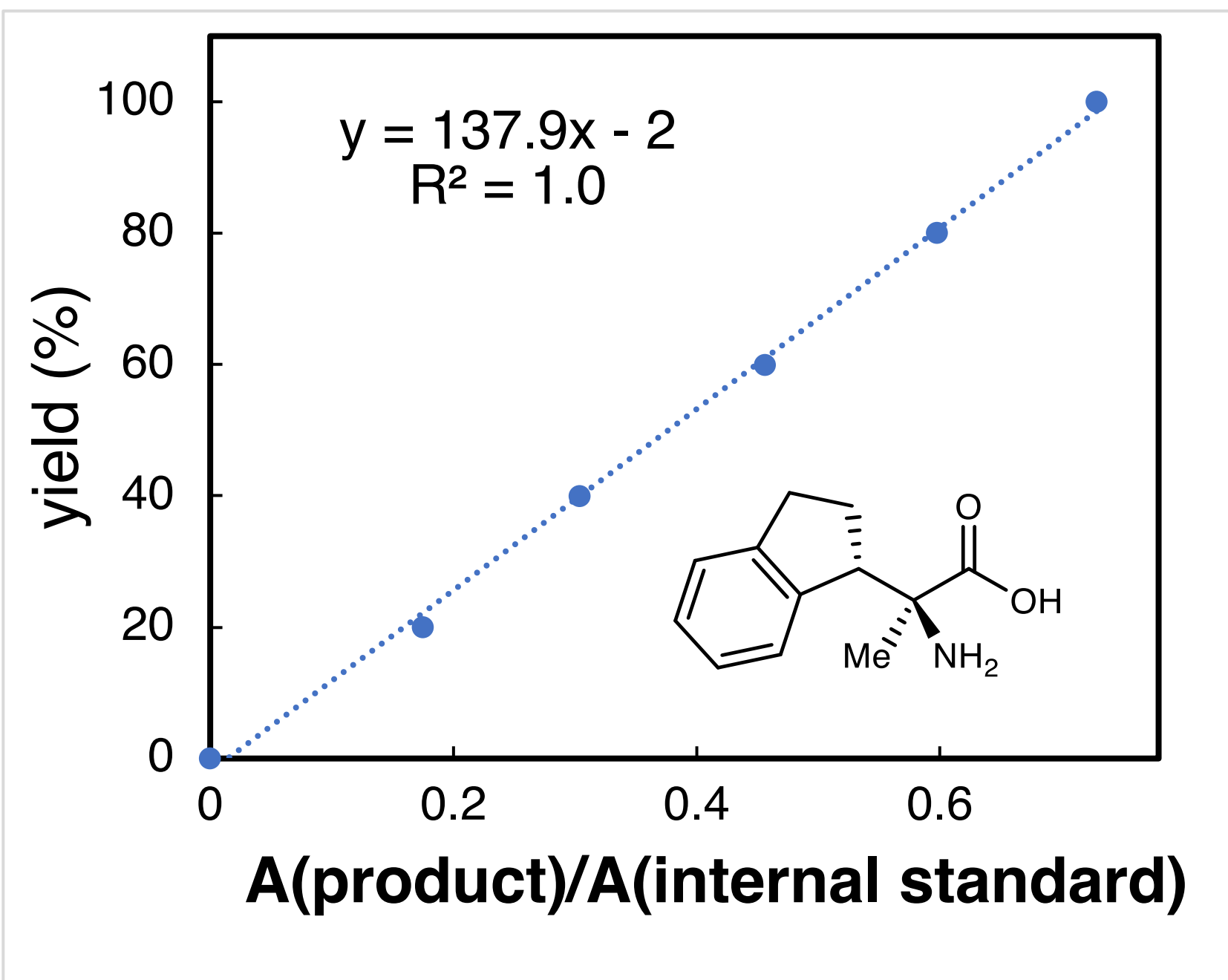
y = 137.9x - 2
R² = 1.0
O
OH
Me
NH2
yield (%)
A(product)/A(internal standard)
100
80
60
40
20
0
0
0.2
0.4
0.6

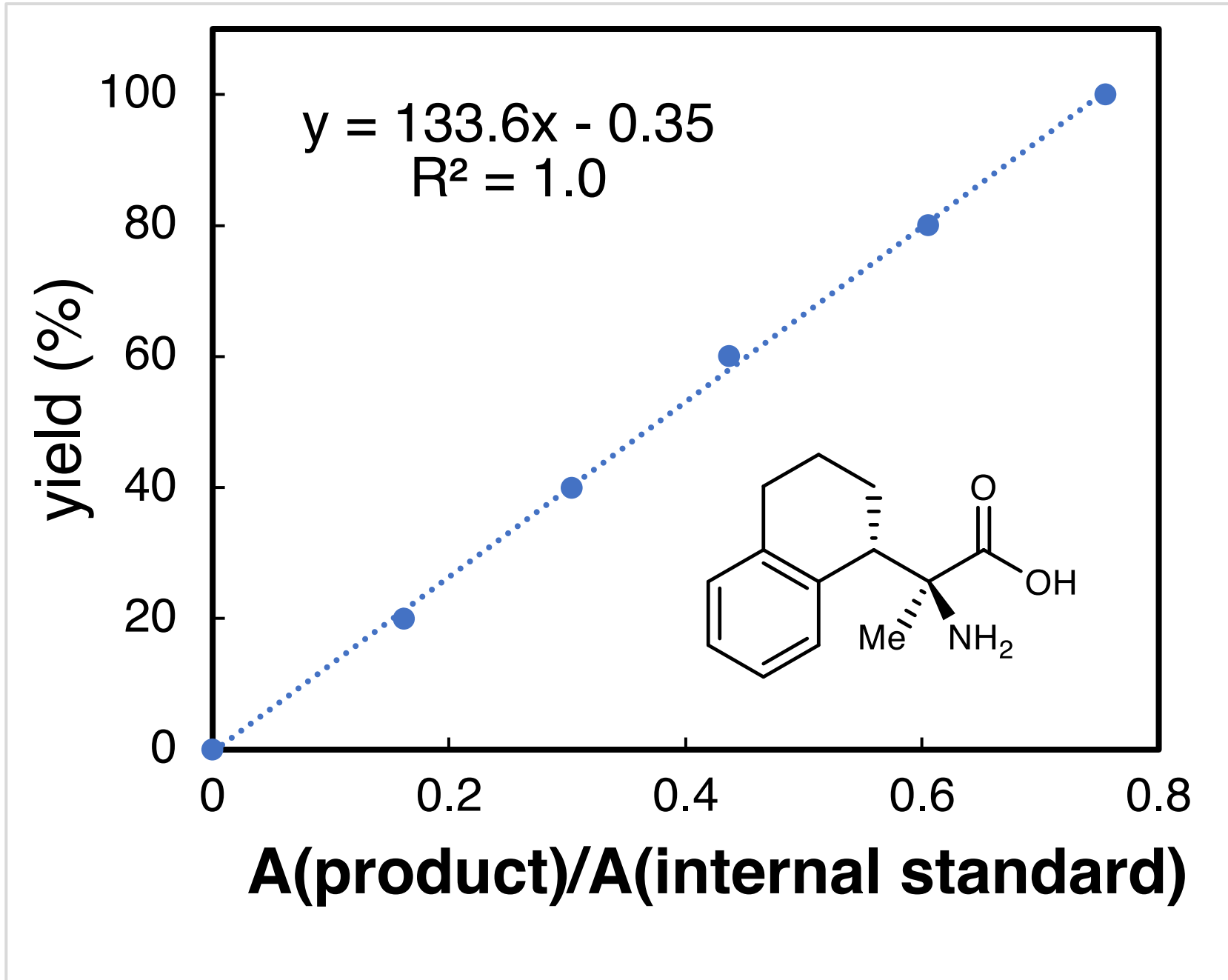
y = 133.6x - 0.35
R² = 1.0
yield (%)
A(product)/A(internal standard)
O
OH
Me
NH2

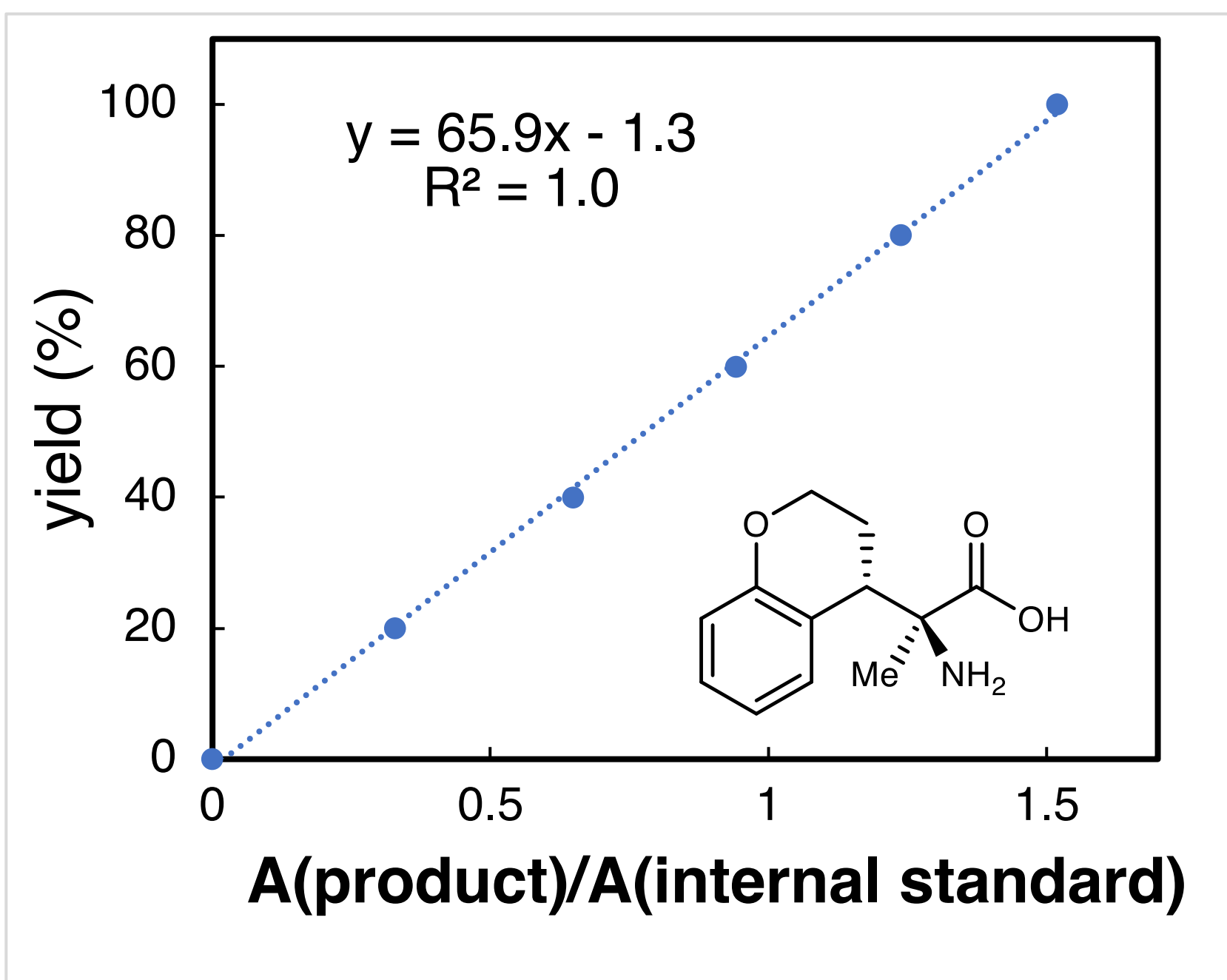
y = 65.9x - 1.3
R² = 1.0
yield (%)
A(product)/A(internal standard)
O
O
OH
Me
NH2

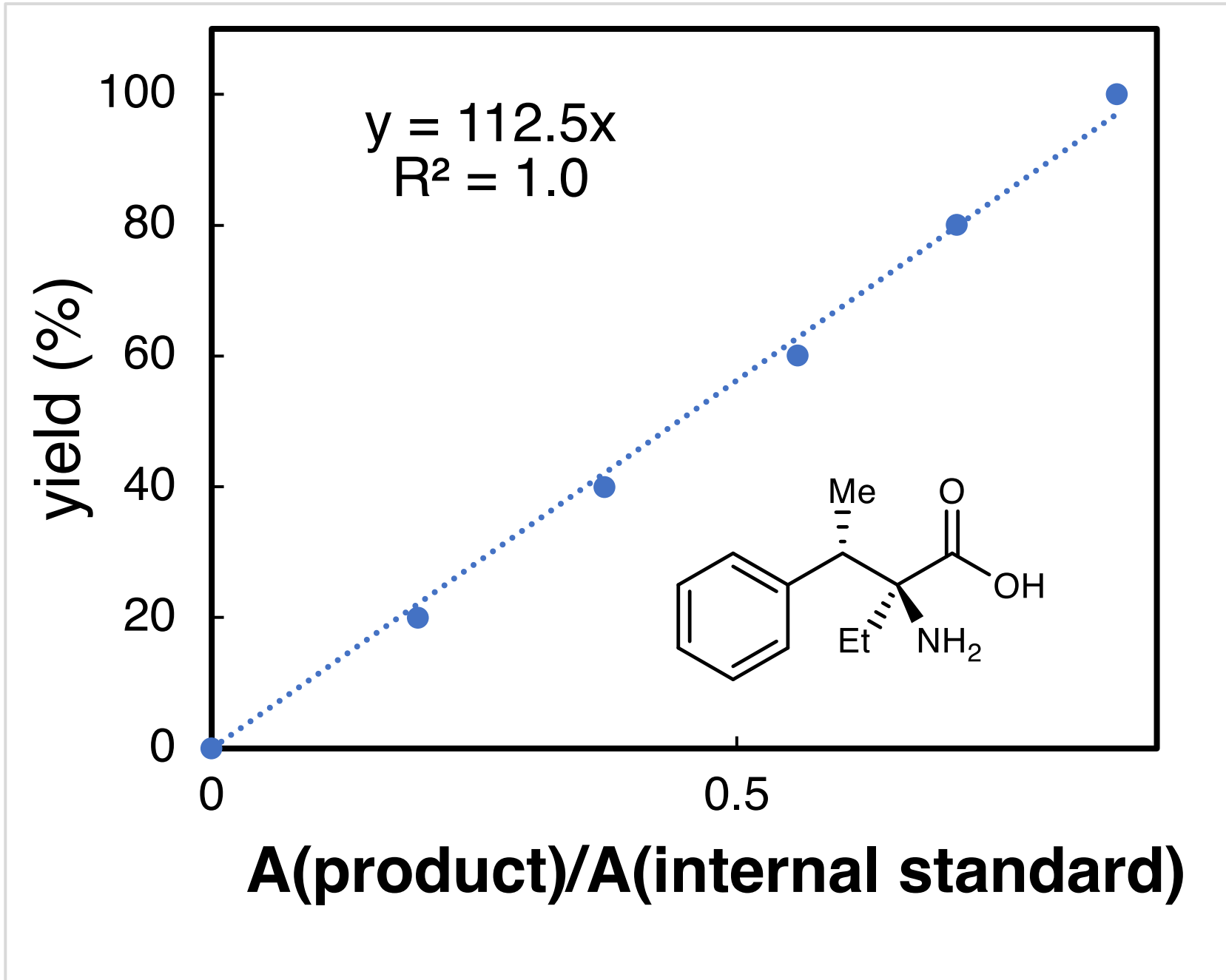

y = 112.5x
R² = 1.0
yield (%)
100
80
60
40
20
0
0
0.5
A(product)/A(internal standard)
Me
O
OH
Et
NH2

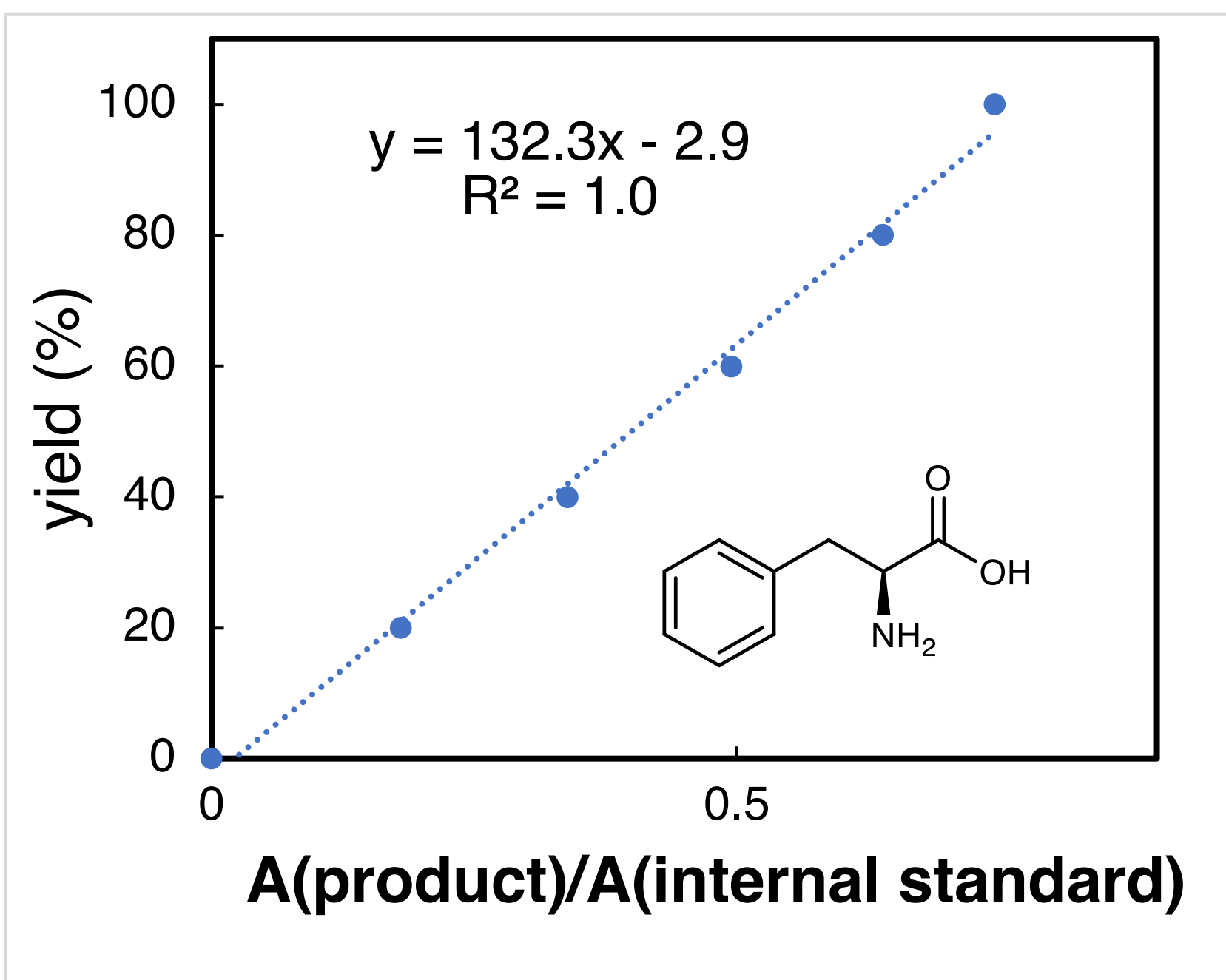

y = 132.3x - 2.9
R² = 1.0
yield (%)
100
80
60
40
20
0
0
0.5
A(product)/A(internal standard)
O
OH
NH2

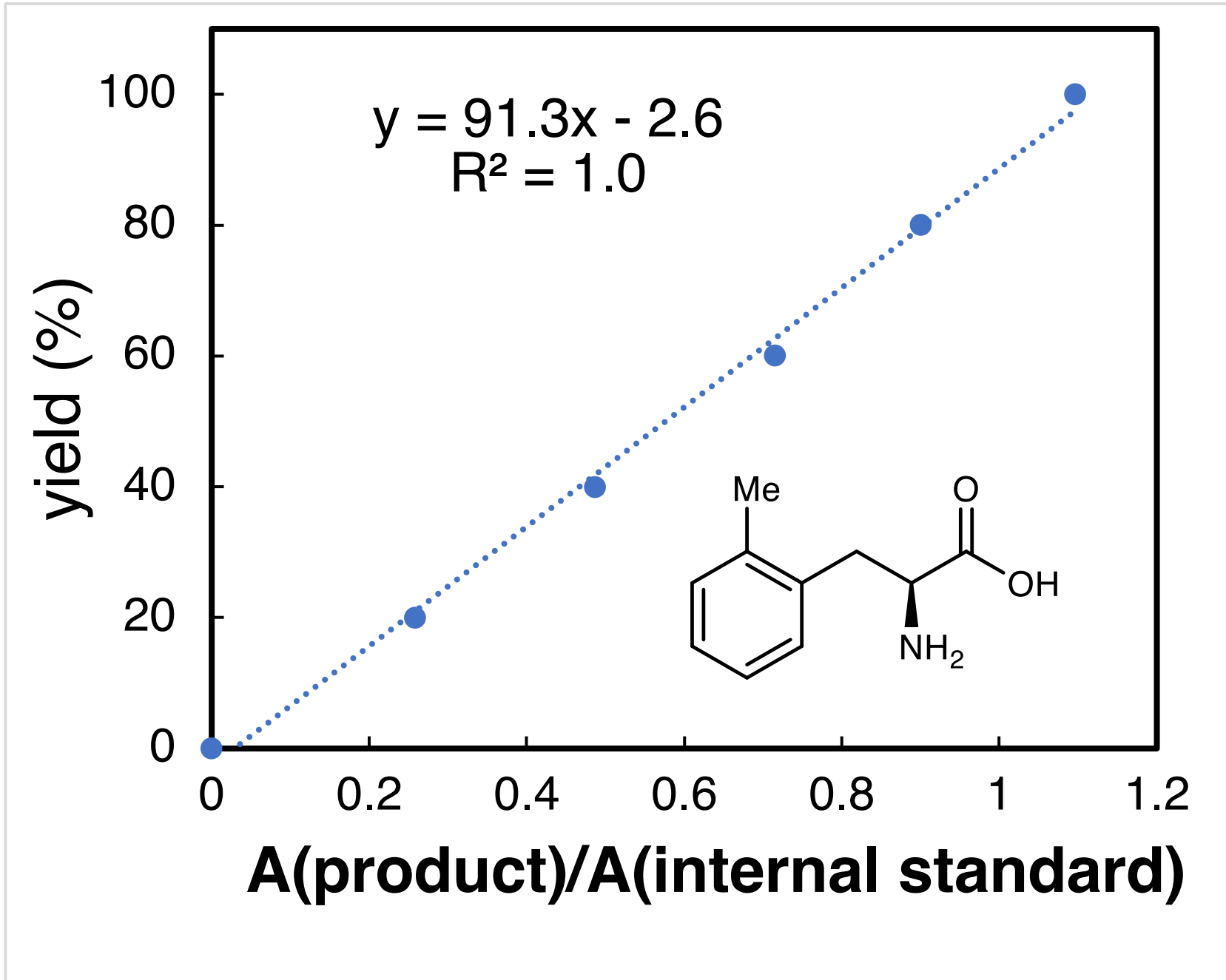
y = 91.3x - 2.6
R² = 1.0
yield (%)
100
80
60
40
20
0
0
0.2
0.4
0.6
0.8
1
1.2
A(product)/A(internal standard)
Me
O
OH
NH2

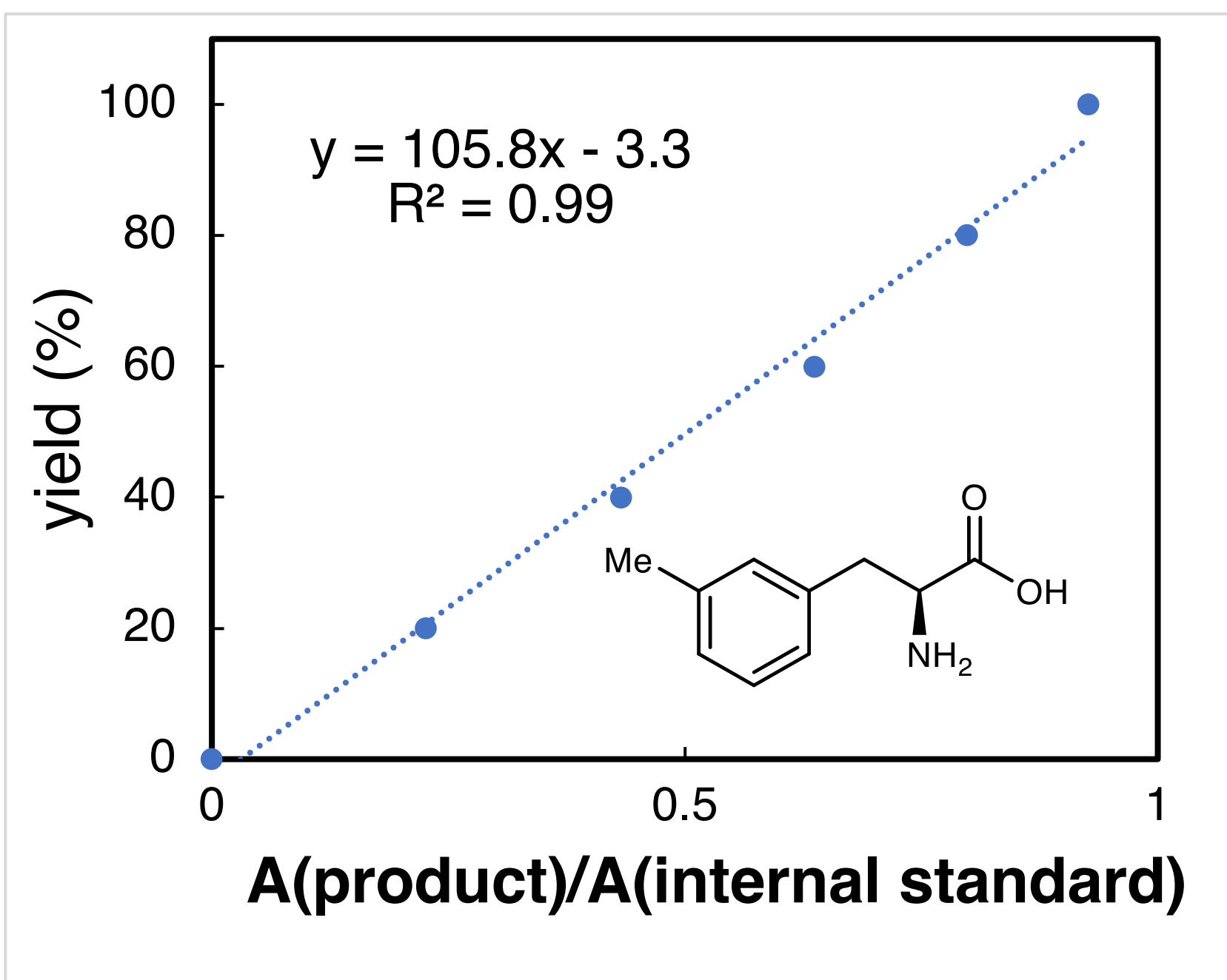
y = 105.8x - 3.3
R² = 0.99
yield (%)
100
80
60
40
20
0
0
0.5
1
A(product)/A(internal standard)
Me
O
OH
NH2

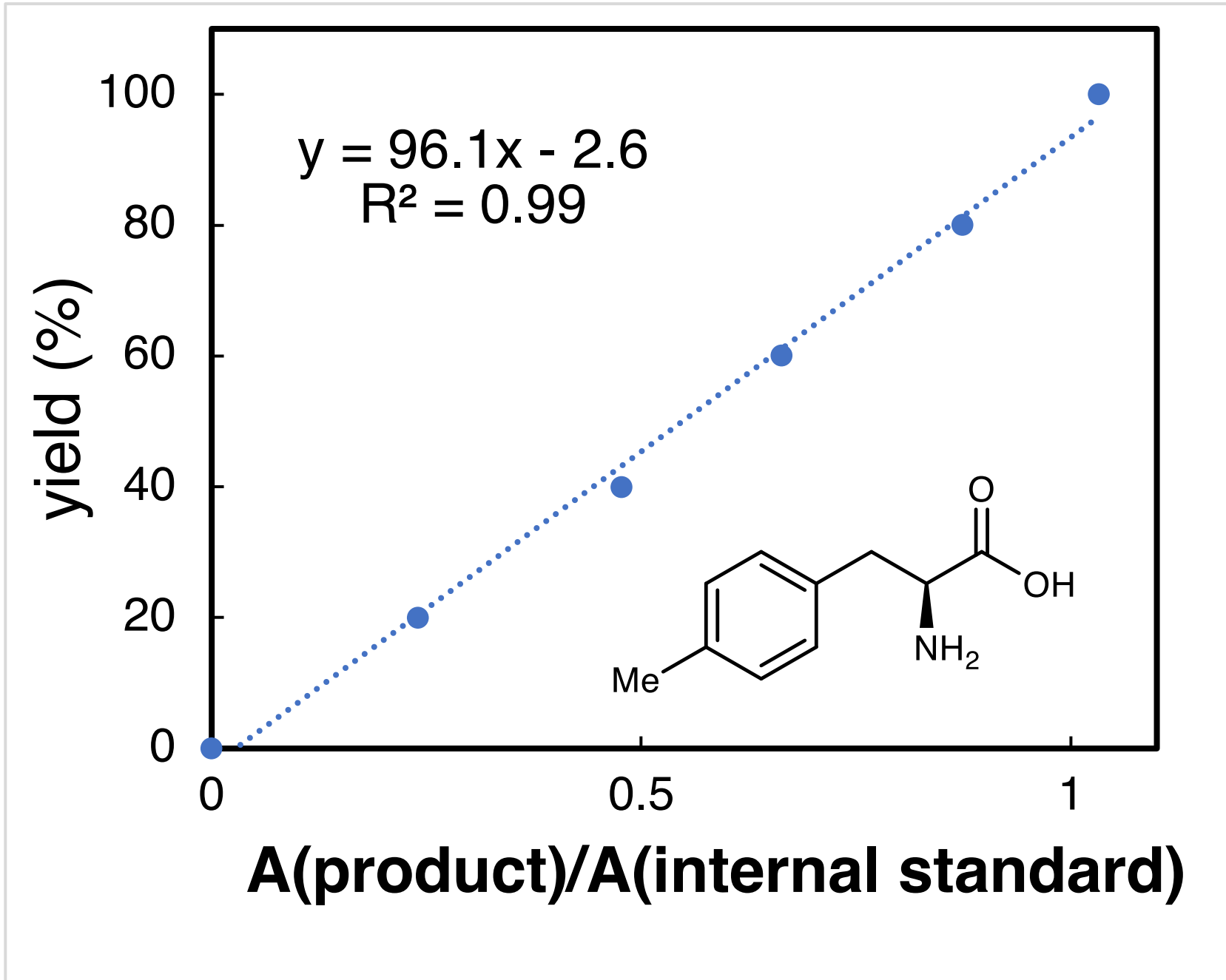

y = 96.1x - 2.6
R² = 0.99
yield (%)
A(product)/A(internal standard)
Me
NH2
OH
O

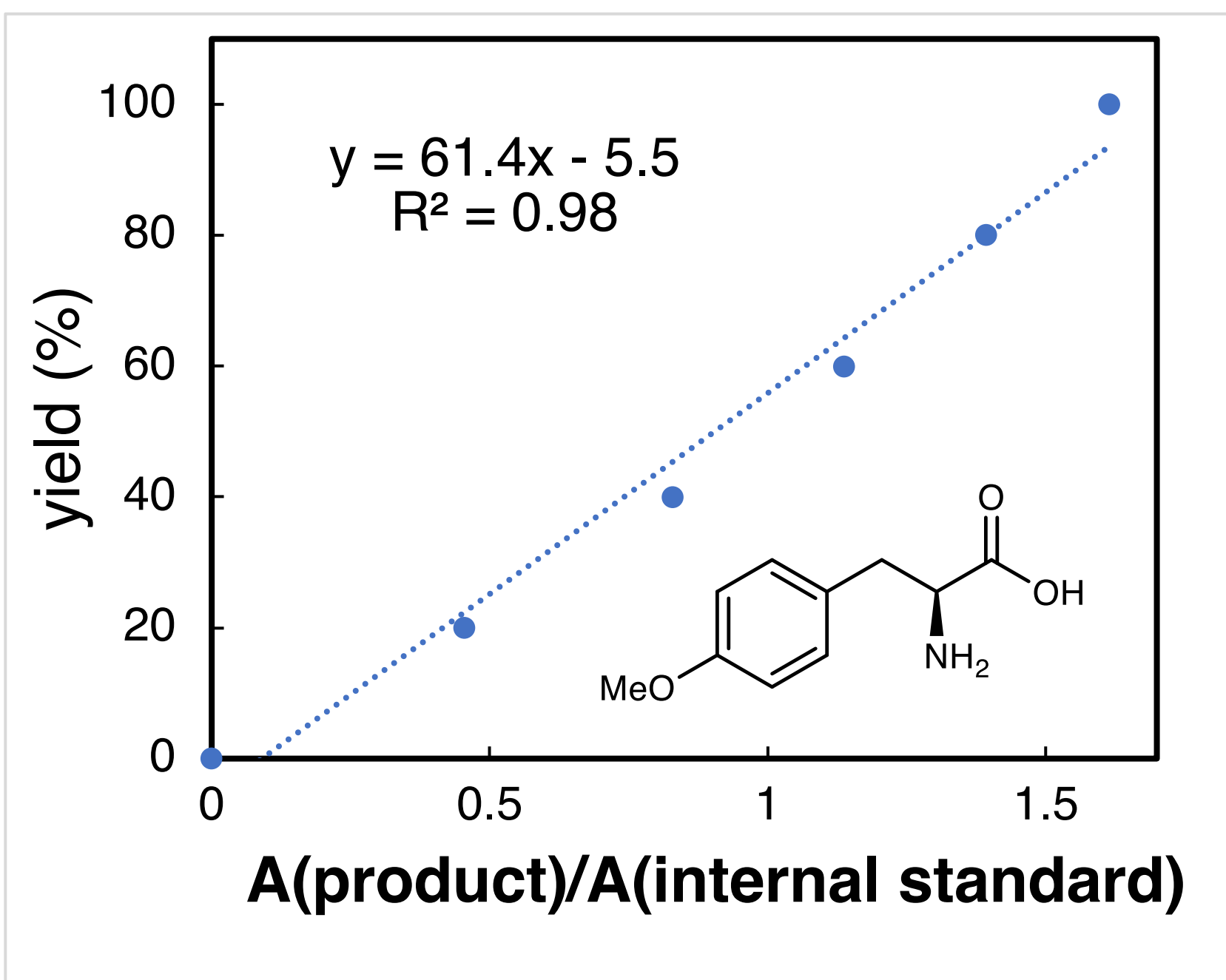

y = 61.4x - 5.5
R² = 0.98
yield (%)
A(product)/A(internal standard)
MeO
NH2
OH
O

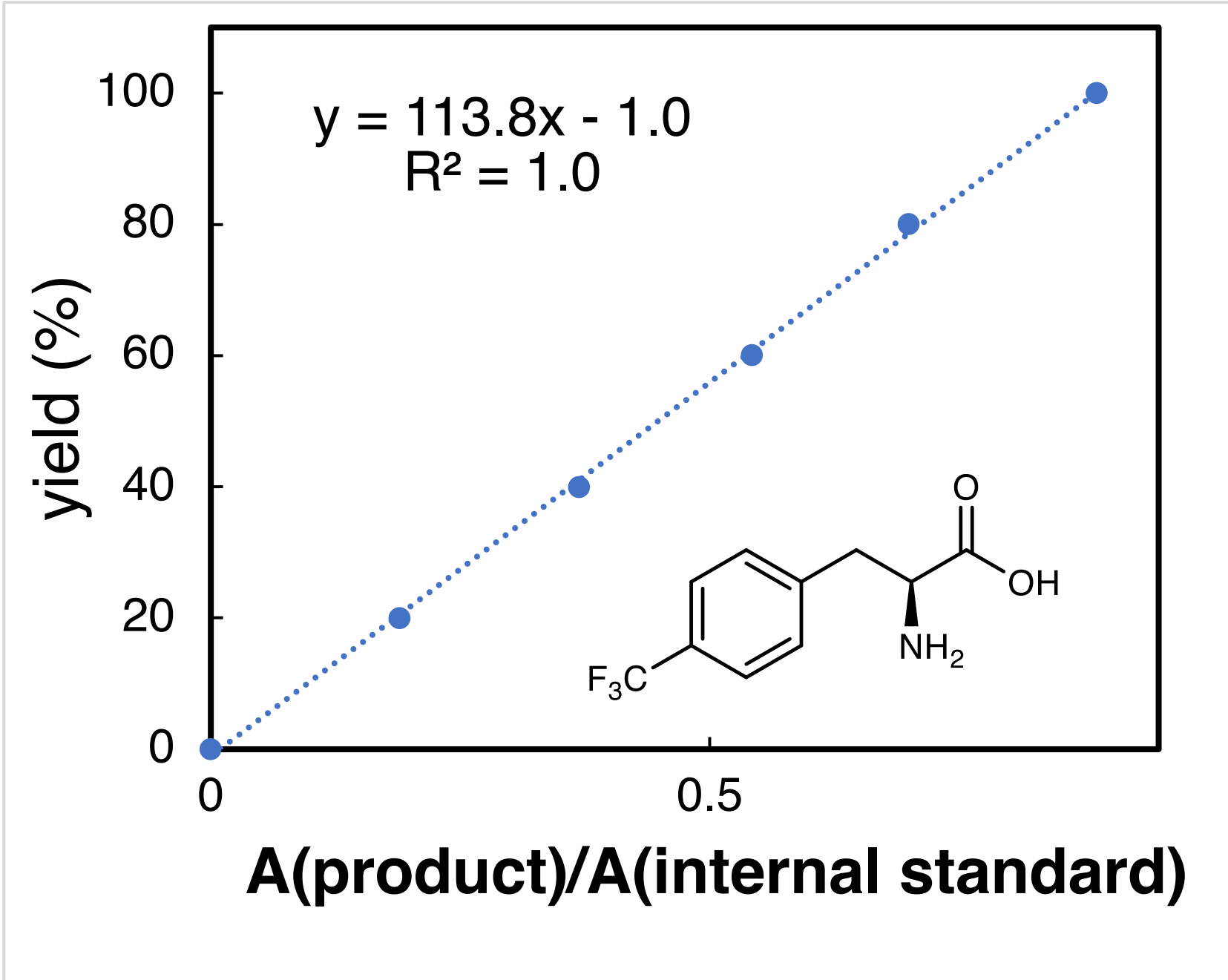

y = 113.8x - 1.0
R² = 1.0
yield (%)
100
80
60
40
20
0
0
0.5
A(product)/A(internal standard)
O
OH
NH2
F3C

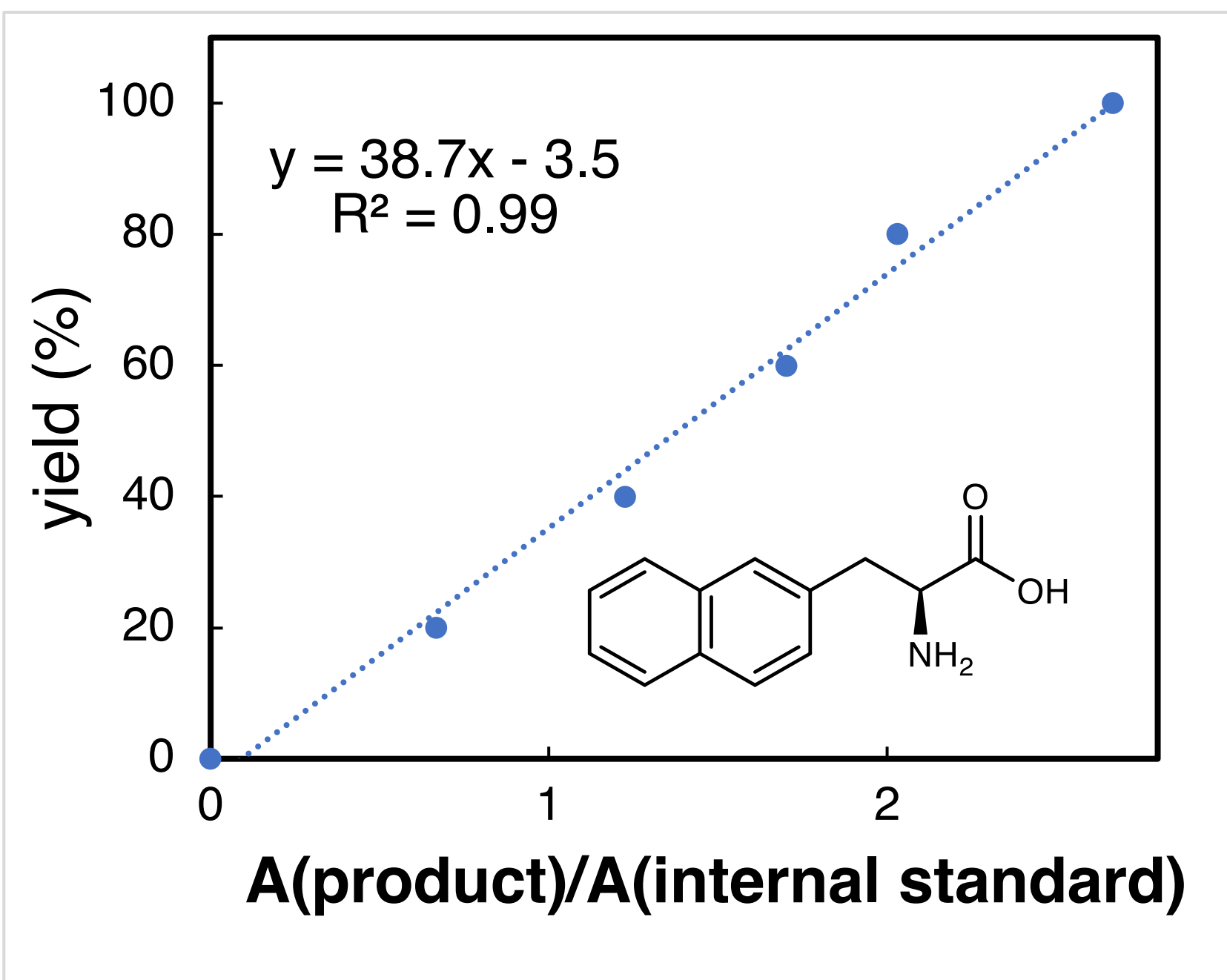

y = 38.7x - 3.5
R² = 0.99
yield (%)
100
80
60
40
20
0
0
1
2
A(product)/A(internal standard)
O
OH
NH2

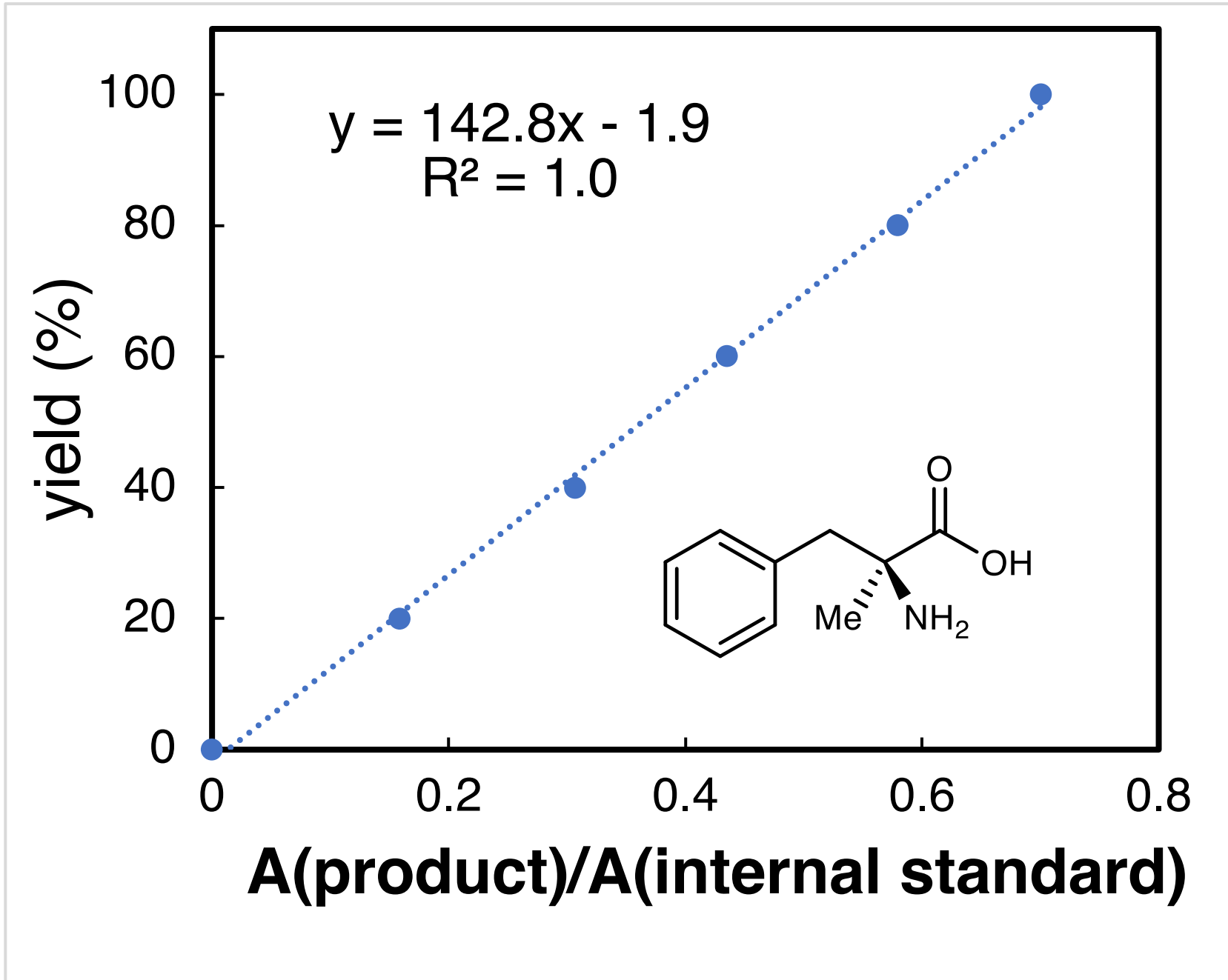
y = 142.8x - 1.9
R² = 1.0
100
80
60
40
20
0
yield (%)
0
0.2
0.4
0.6
0.8
A(product)/A(internal standard)
O
OH
Me
NH2

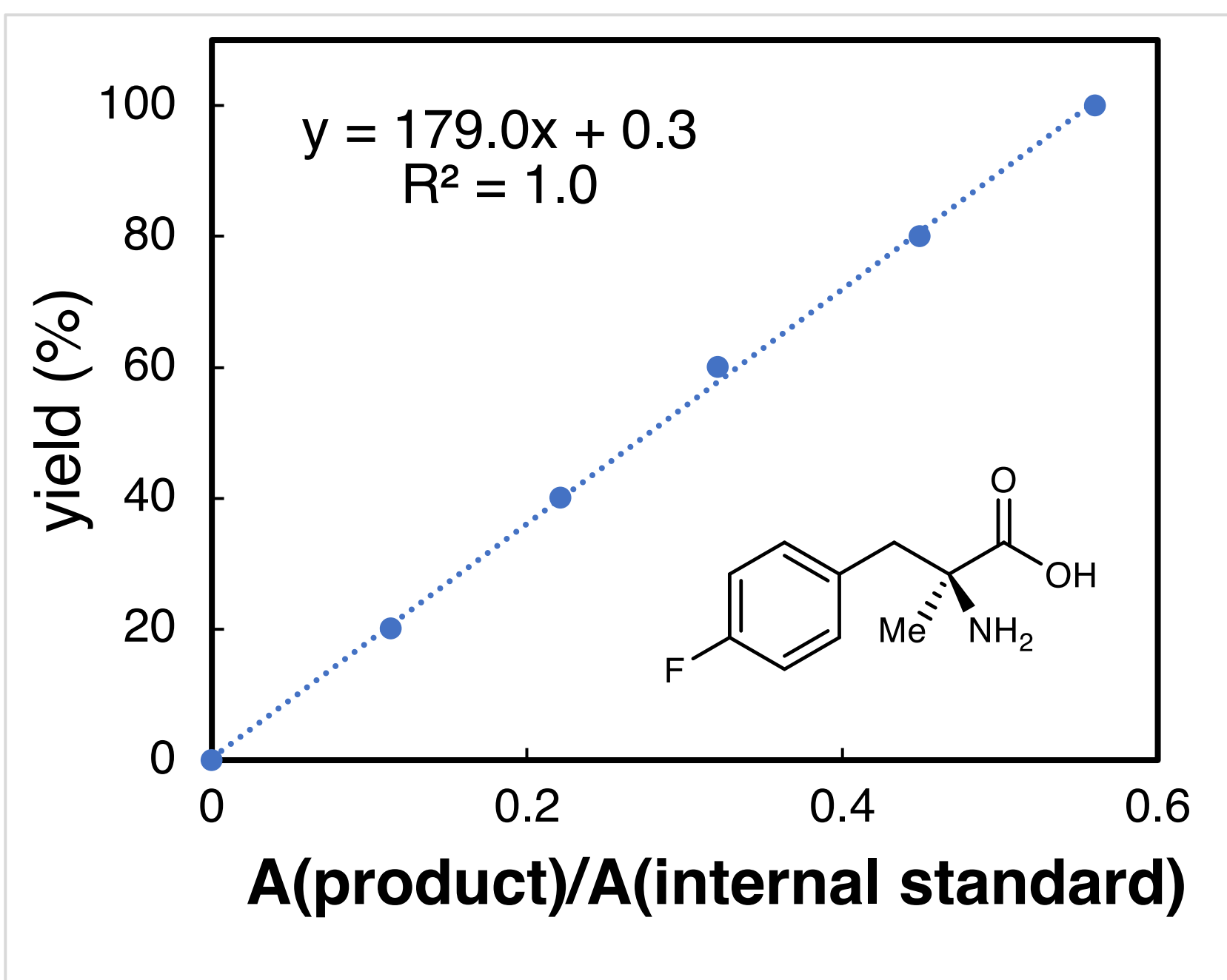
y = 179.0x + 0.3
R² = 1.0
100
80
60
40
20
0
yield (%)
0
0.2
0.4
0.6
A(product)/A(internal standard)
O
OH
F
Me
NH2

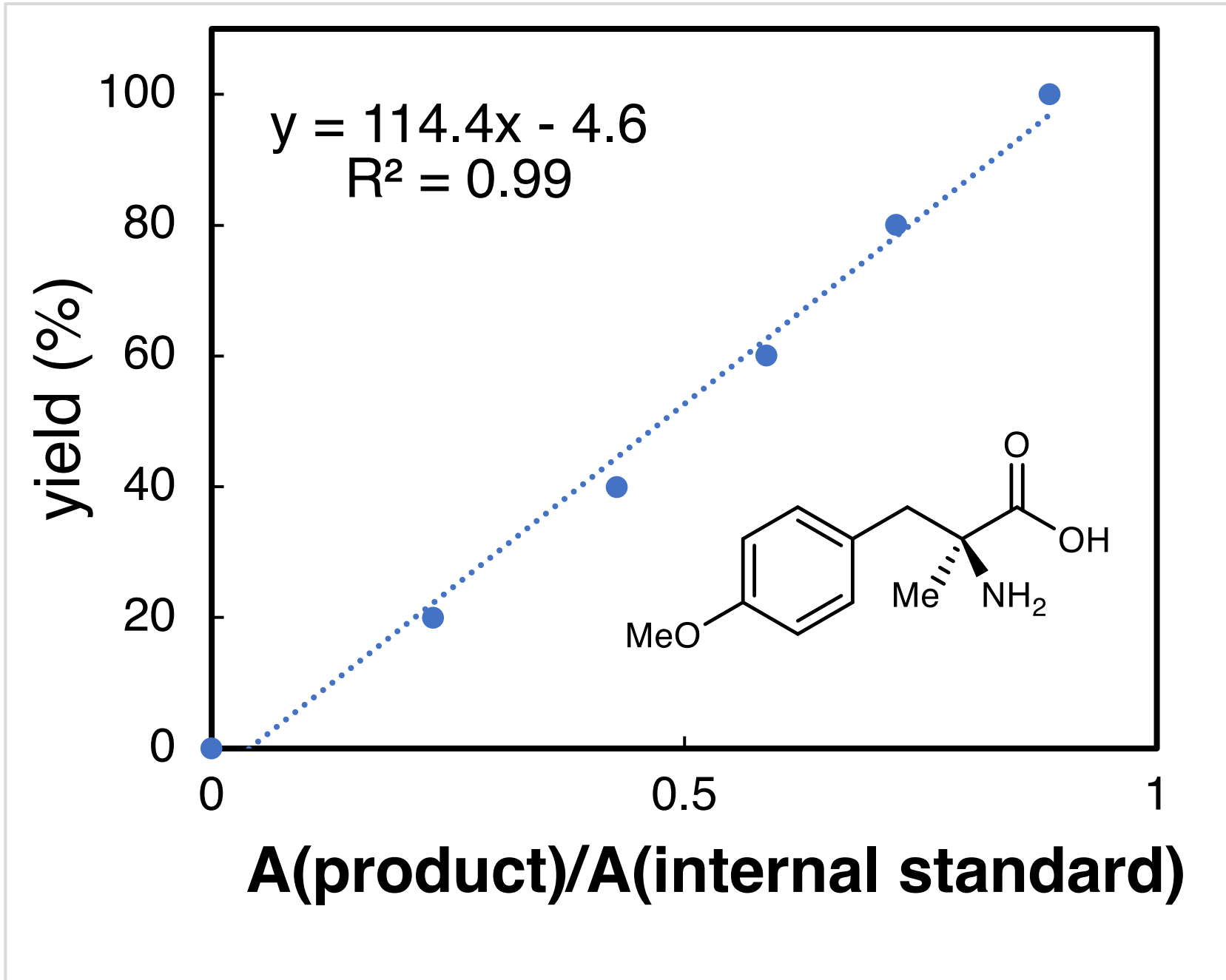

y = 114.4x - 4.6
R² = 0.99
yield (%)
100
80
60
40
20
0
0
0.5
1
A(product)/A(internal standard)
O
OH
MeO
Me
NH2

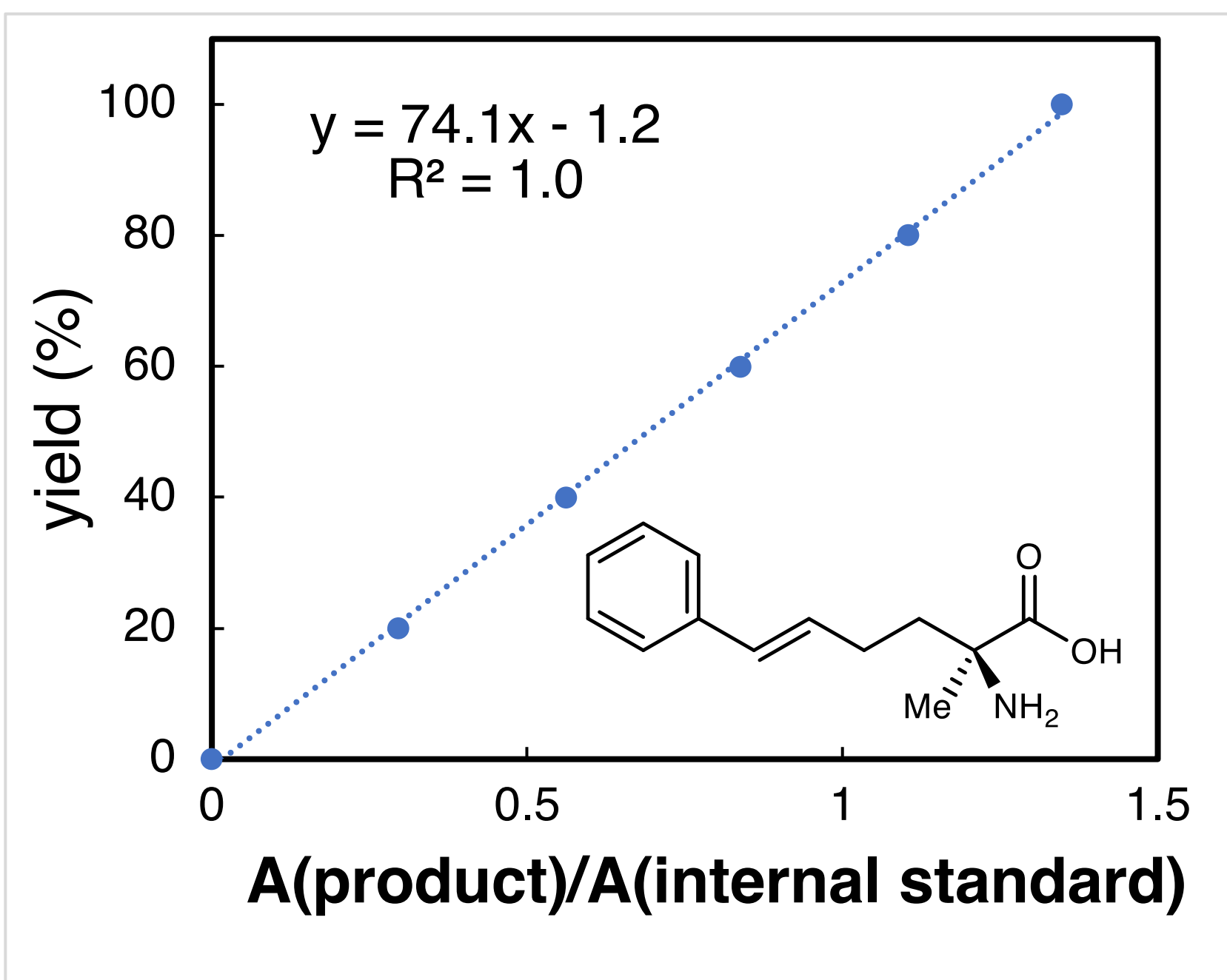

y = 74.1x - 1.2
R² = 1.0
yield (%)
100
80
60
40
20
0
0
0.5
1
1.5
A(product)/A(internal standard)
O
OH
Me
NH2

XII. HPLC traces for Marfey's analysis and Fmoc chloride derivatization

(2*S*,3*S*)-2-amino-2-methyl-3-phenylbutanoic acid (3a)

Fmoc derivatization of (*rac*)-**3a**

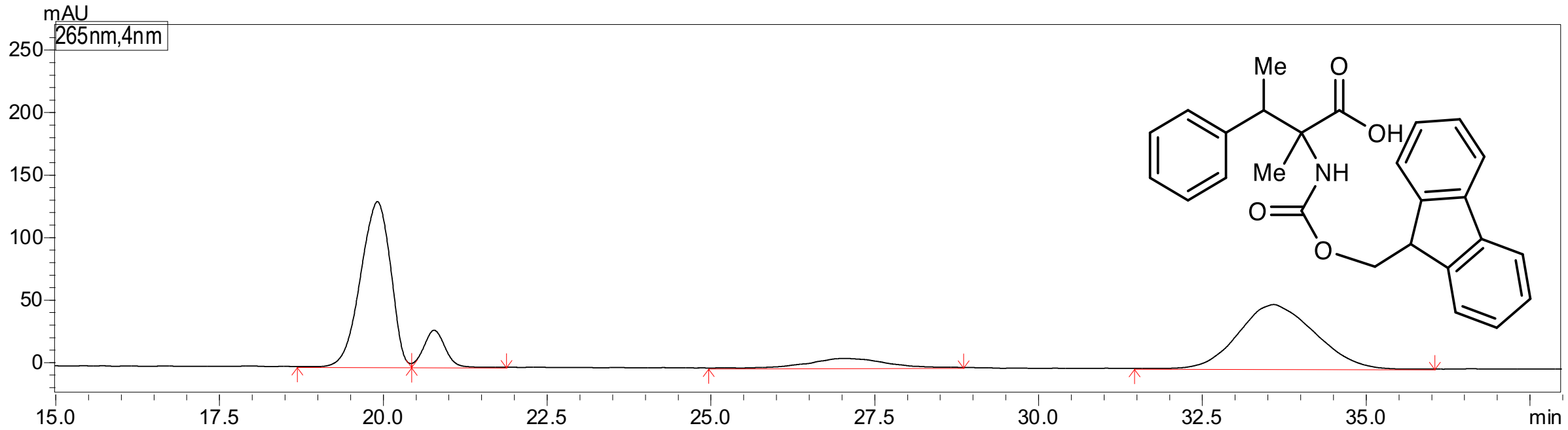

Peak#	Ret. Time	Height	Area	Area%
1	19.923	132133	4200703	43.968
2	20.788	29412	658097	6.888
3	27.052	7395	566721	5.932
4	33.603	51321	4128400	43.212
Total		220262	9553922	100.000

Fmoc derivatization of (2*S*,3*S*)-**3a**

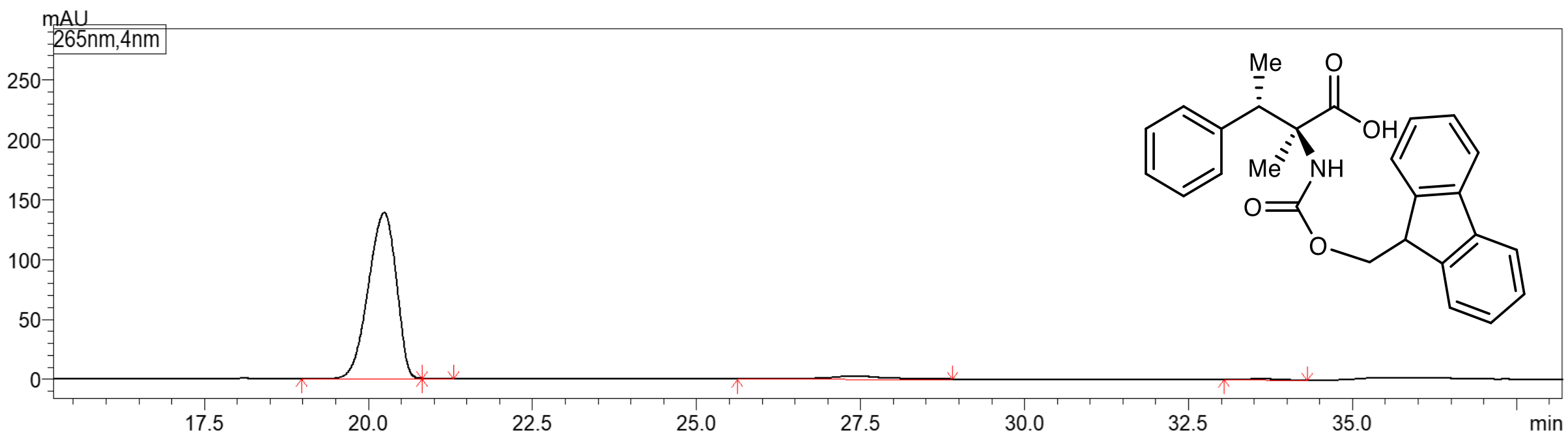

Peak#	Ret. Time	Height	Area	Area%
1	20.243	138861	4131546	95.868
2	20.826	461	7205	0.167
3	27.312	2228	151130	3.507
4	33.653	538	19718	0.458
Total		142087	4309599	100.000

(2*S*,3*S*)-2-amino-2-methyl-3-(o-tolyl)butanoic acid (3b)

Fmoc derivatization of (*rac*)-**3b**

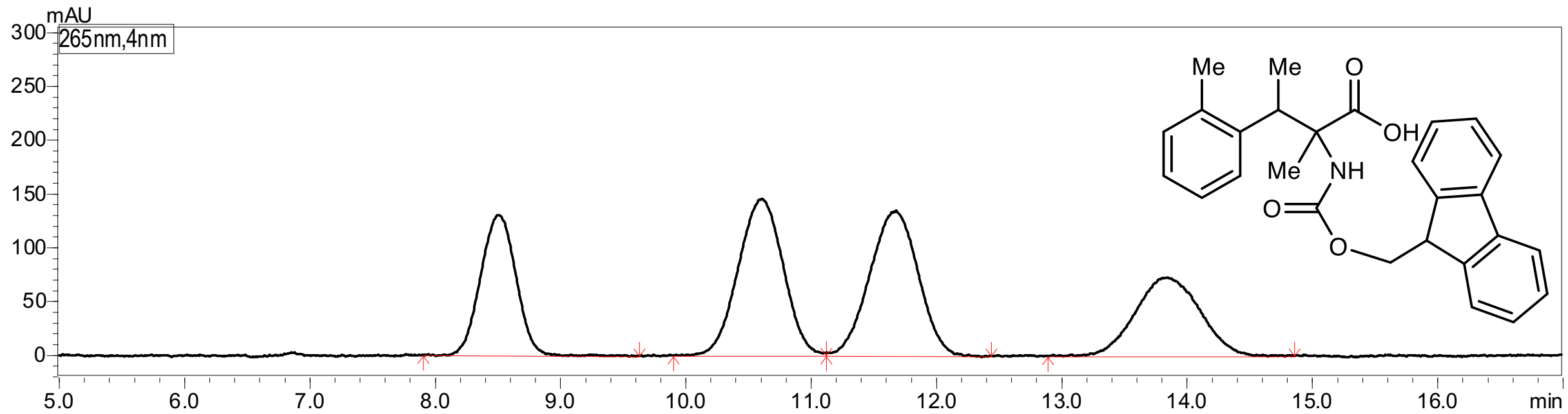

Peak Table | Compound | Group | Calibration Curve

Peak#	Ret. Time	Height	Area	Area%
1	8.506	130252	2539802	20.567
2	10.615	145245	3615821	29.281
3	11.686	134807	3609170	29.227
4	13.851	72507	2583846	20.924
Total		482810	12348638	100.000

Fmoc derivatization of (2*S*,3*S*)-**3b**

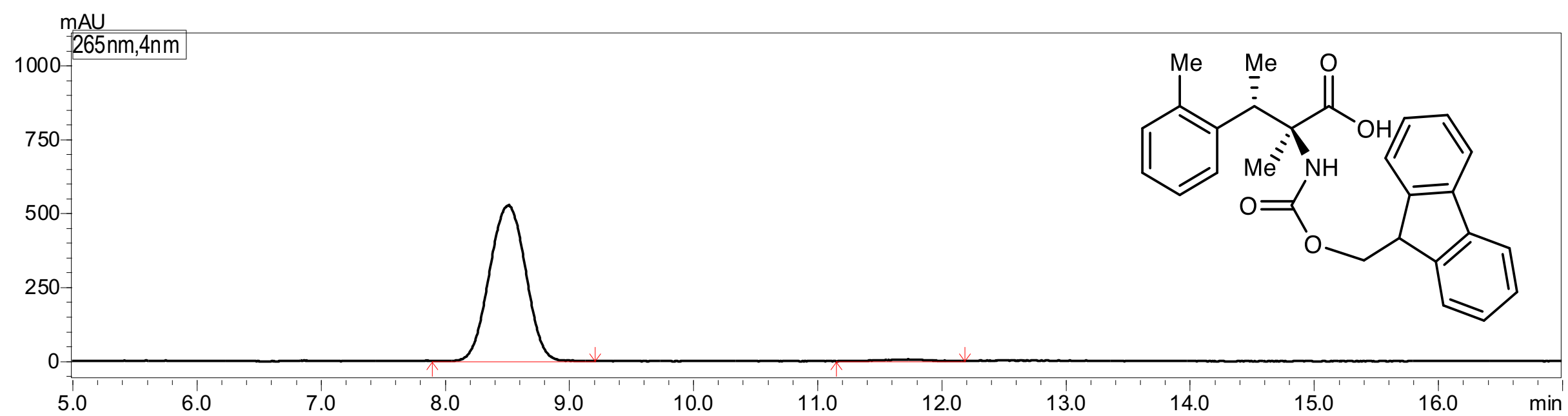

Peak Table | Compound | Group | Calibration Curve

Peak#	Ret. Time	Height	Area	Area%
1	8.517	527353	10631180	98.921
2	11.739	5305	115944	1.079
Total		532658	10747124	100.000

(2*S*,3*S*)-2-amino-2-methyl-3-(m-tolyl)butanoic acid (3c)

Fmoc derivatization of (*rac*)-**3c**

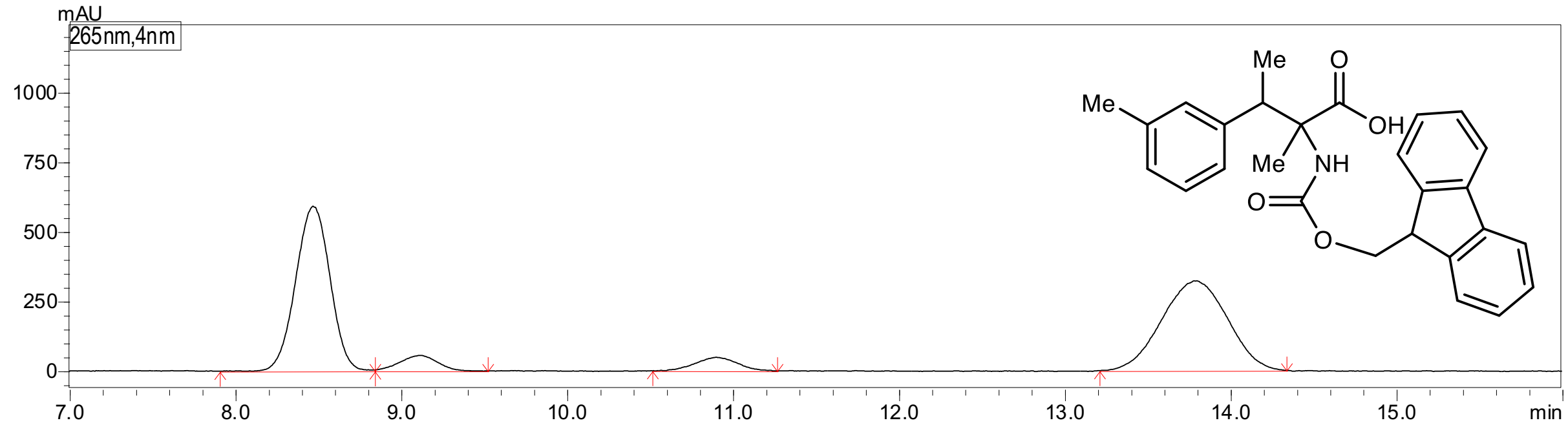

Peak Table | Compound | Group | Calibration Curve

Peak#	Ret. Time	Height	Area	Area%
1	8.470	591112	8520052	44.619
2	9.112	55589	887364	4.647
3	10.901	48477	852642	4.465
4	13.797	321562	8835125	46.269
Total		1016740	19095183	100.000

Fmoc derivatization of (2*S*,3*S*)-**3c**

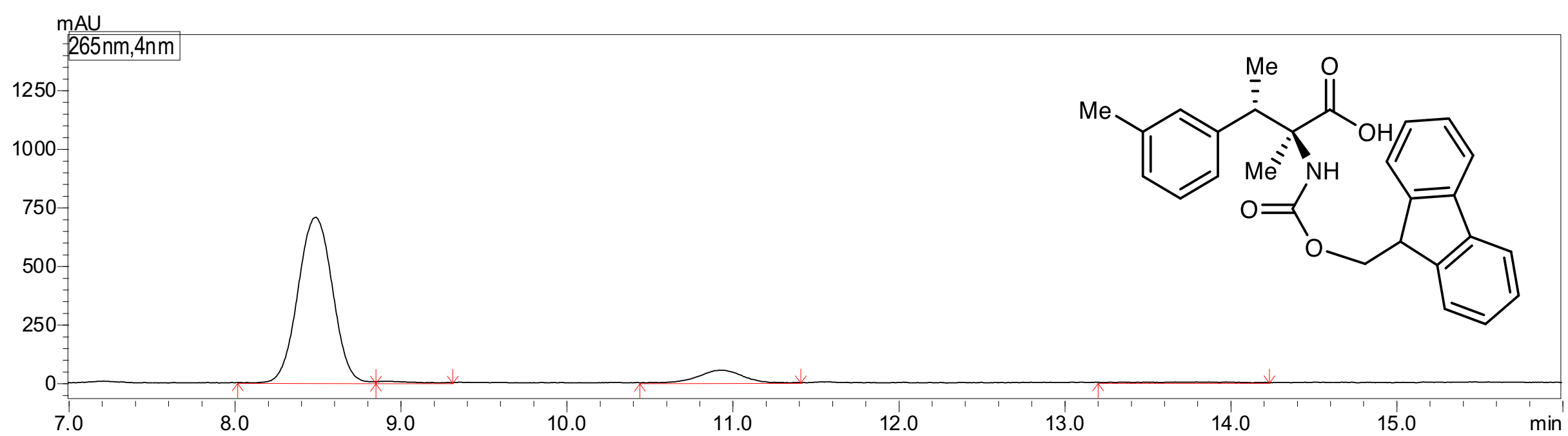

Peak Table | Compound | Group | Calibration Curve

Peak#	Ret. Time	Height	Area	Area%
1	8.491	705635	10150279	90.664
2	8.905	5871	66374	0.593
3	10.942	52820	925879	8.270
4	13.799	2220	52977	0.473
Total		766546	11195508	100.000

(2*S*,3*S*)-2-amino-2-methyl-3-(p-tolyl)butanoic acid (3d)

Fmoc derivatization of (*rac*)-**3d**

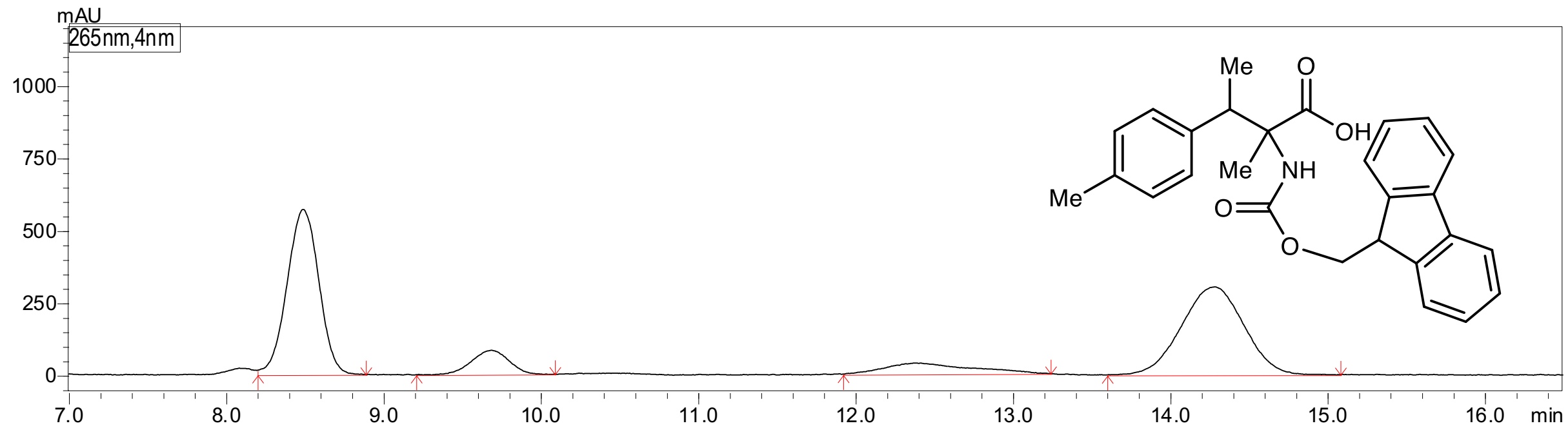

Peak#	Ret. Time	Height	Area	Area%
1	8.491	569711	8029660	41.828
2	9.686	83457	1367215	7.122
3	12.389	37684	1430946	7.454
4	14.287	303233	8368877	43.595
Total		994086	19196698	100.000

Fmoc derivatization of (2*S*,3*S*)-**3d**

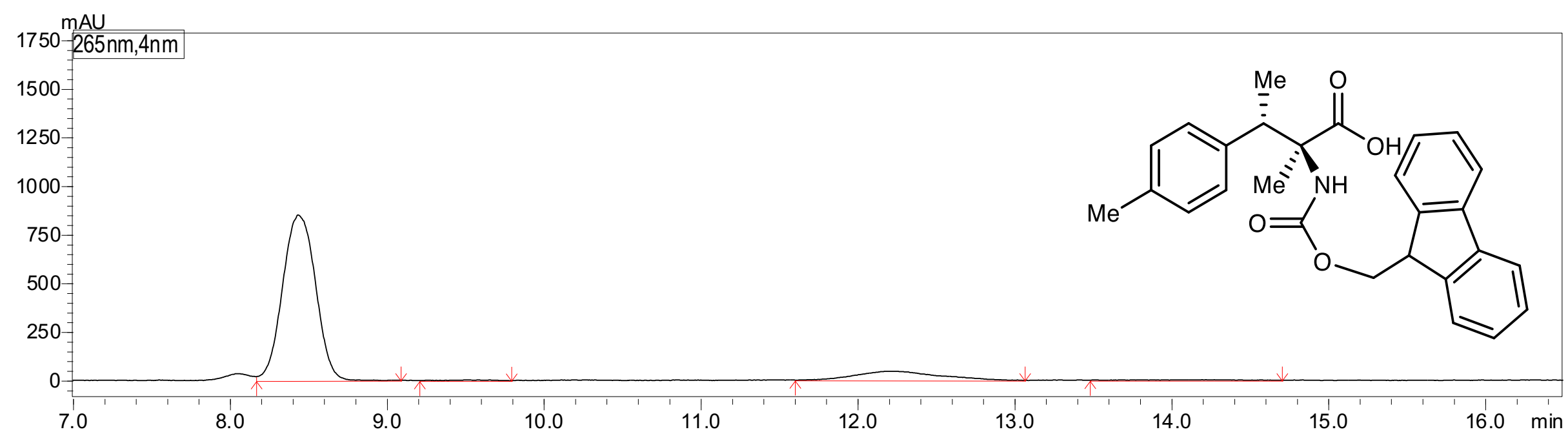

Peak#	Ret. Time	Height	Area	Area%
1	8.438	849575	12415356	87.512
2	9.504	3466	56430	0.398
3	12.191	45915	1646670	11.607
4	14.222	2485	68607	0.484
Total		901441	14187063	100.000

(2*S*,3*S*)-2-amino-3-(4-methoxyphenyl)-2-methylbutanoic acid (3e)

Fmoc derivatization of (*rac*)-**3e**

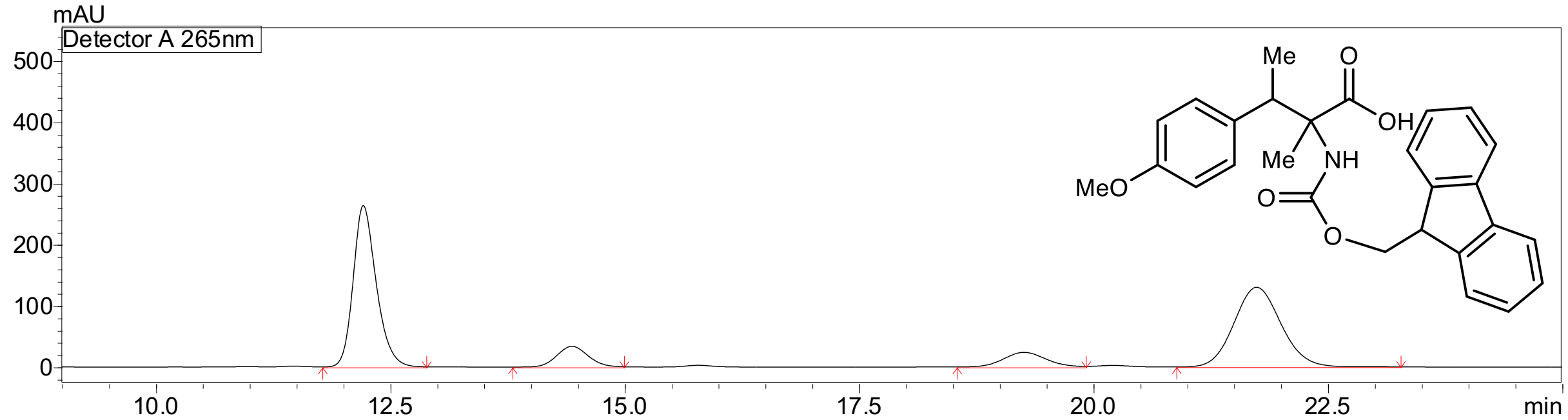

Peak#	Ret. Time	Height	Area	Area%
1	12.214	263657	4526315	42.679
2	14.439	33842	785697	7.408
3	19.261	23915	757640	7.144
4	21.741	130160	4535953	42.769
Total		451573	10605605	100.000

Fmoc derivatization of (2*S*,3*S*)-**3e**

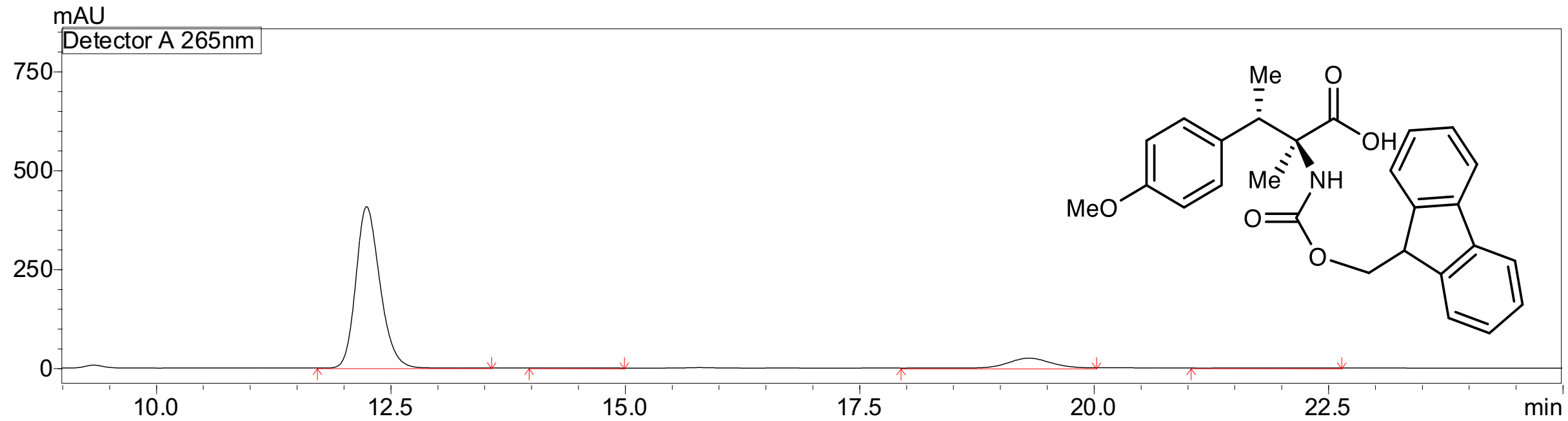

Peak#	Ret. Time	Height	Area	Area%
1	12.248	407737	7287946	90.150
2	14.502	131	3620	0.045
3	19.311	24992	788895	9.758
4	21.792	83	3763	0.047
Total		432943	8084224	100.000

(2*S*,3*S*)-2-amino-3-(4-fluorophenyl)-2-methylbutanoic acid (3f)

Fmoc derivatization of (*rac*)-**3f**

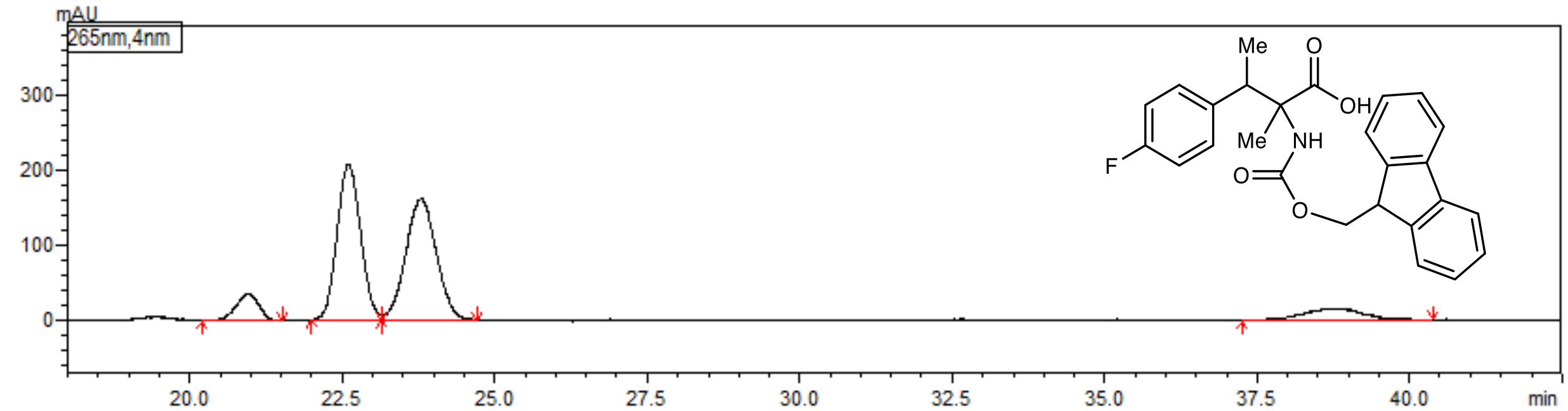

Peak Table | Compound | Group | Calibration Curve

Peak#	Ret. Time	Height	Area	Area%
1	20.979	35474	900948	6.861
2	22.596	207954	5550253	42.268
3	23.791	161687	5643220	42.976
4	38.749	15650	1036823	7.896
Total		420765	13131245	100.000

Fmoc derivatization of (2*S*,3*S*)-**3f**

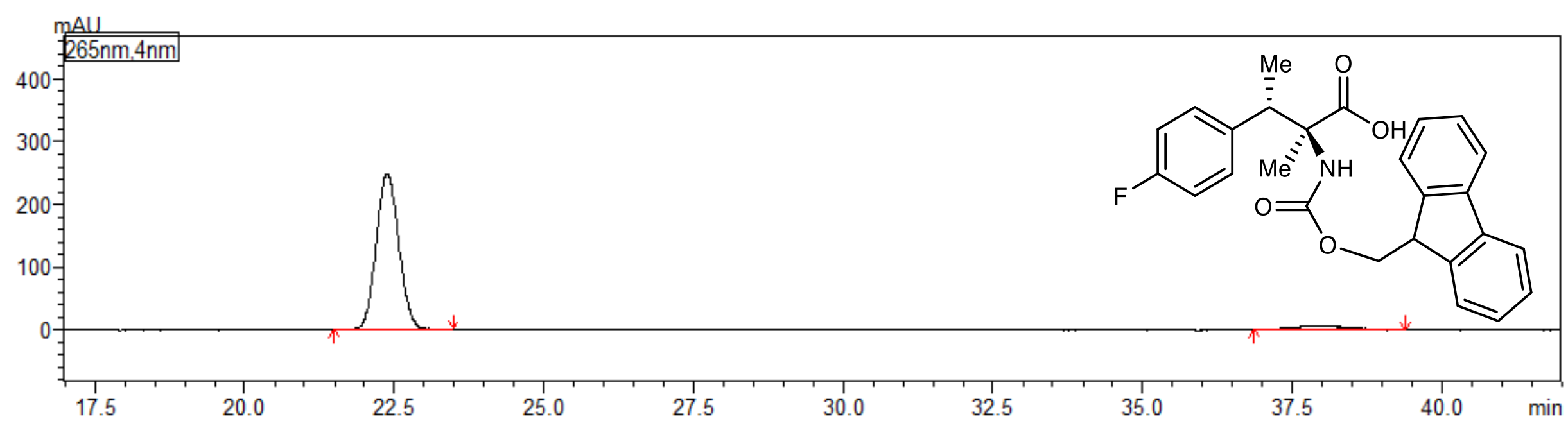

Peak Table | Compound | Group | Calibration Curve

Peak#	Ret. Time	Height	Area	Area%
1	22.381	247728	6483827	94.159
2	37.976	7179	402195	5.841
Total		254908	6886022	100.000

(2*S*,3*S*)-2-amino-3-(4-chlorophenyl)-2-methylbutanoic acid (3g)

Fmoc derivatization of (*rac*)-**3g**

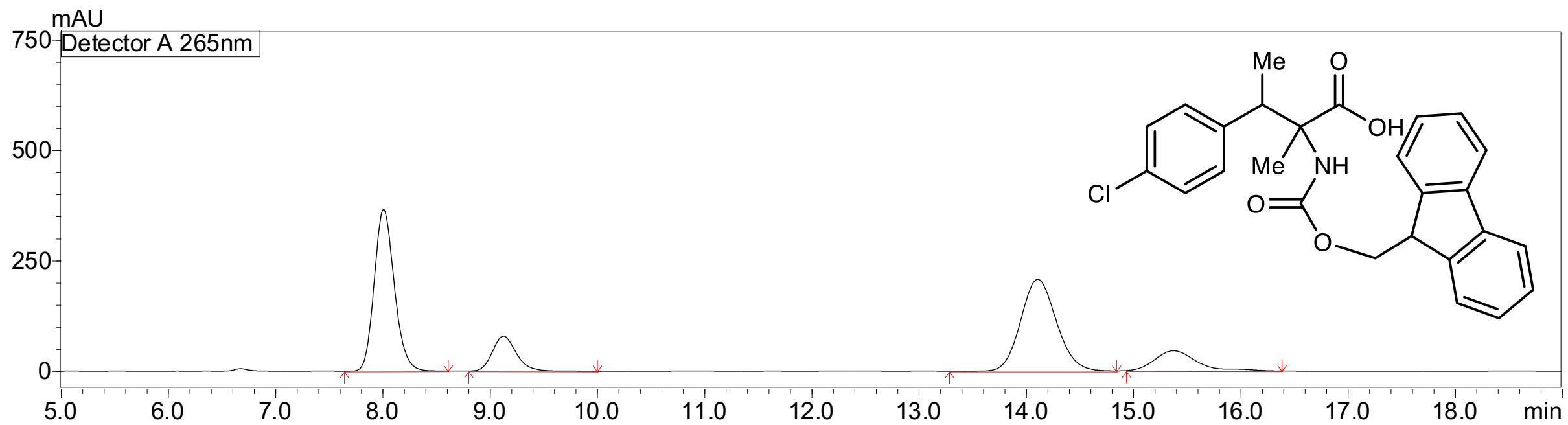

Peak Table | Compound | Group | Calibration Curve

Peak#	Ret. Time	Height	Area	Area%
1	8.012	365712	4767849	39.680
2	9.130	78868	1215343	10.115
3	14.112	207820	4811163	40.040
4	15.378	45229	1221445	10.165
Total		697629	12015800	100.000

Fmoc derivatization of (2*S*,3*S*)-**3g**

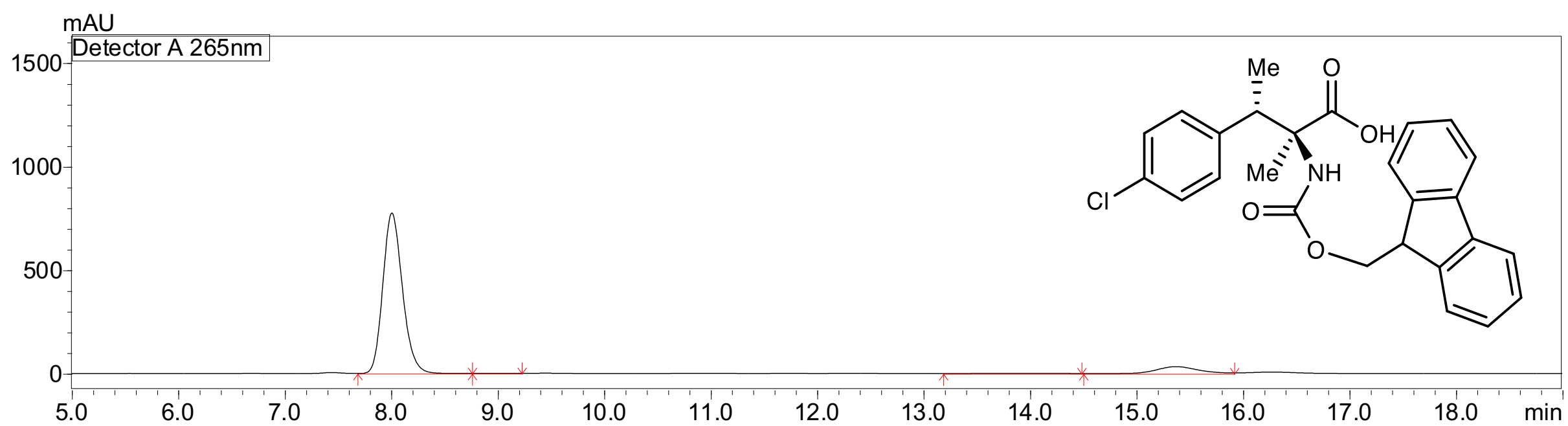

Peak Table | Compound | Group | Calibration Curve

Peak#	Ret. Time	Height	Area	Area%
1	8.008	774885	9911231	91.700
2	9.090	276	4537	0.042
3	14.007	732	22999	0.213
4	15.372	32826	869529	8.045
Total		808718	10808296	100.000

(2*S*,3*S*)-2-amino-3-(4-bromophenyl)-2-methylbutanoic acid (3h)

Fmoc derivatization of (*rac*)-**3h**

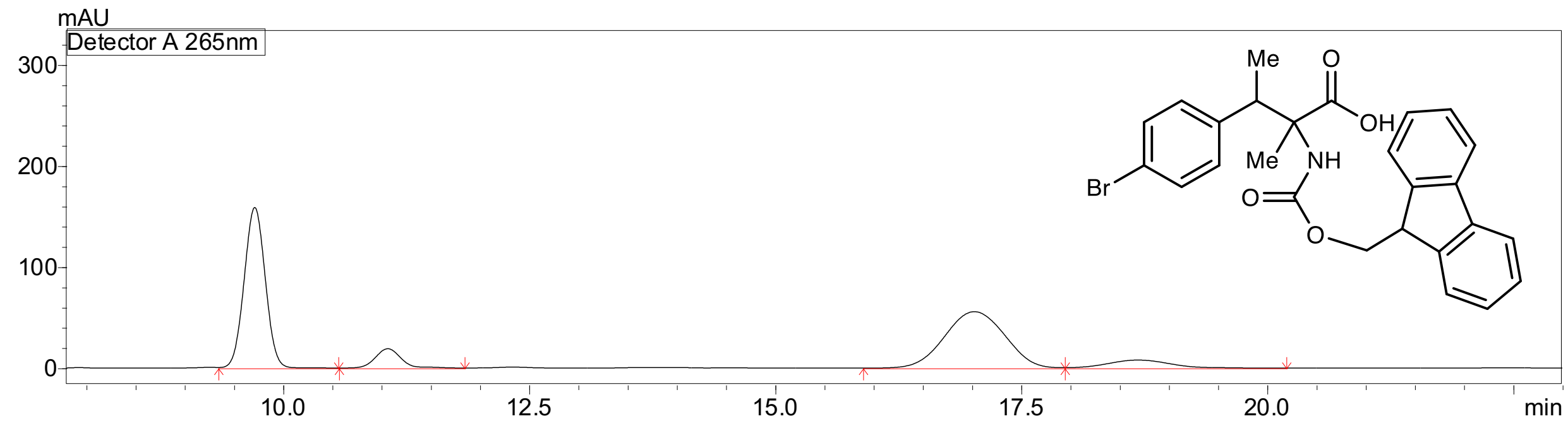

Peak#	Ret. Time	Height	Area	Area%
1	9.713	158775	2362466	43.525
2	11.065	19065	342644	6.313
3	17.025	55763	2368394	43.635
4	18.691	7970	354293	6.527
Total		241574	5427796	100.000

Fmoc derivatization of (2*S*,3*S*)-**3h**

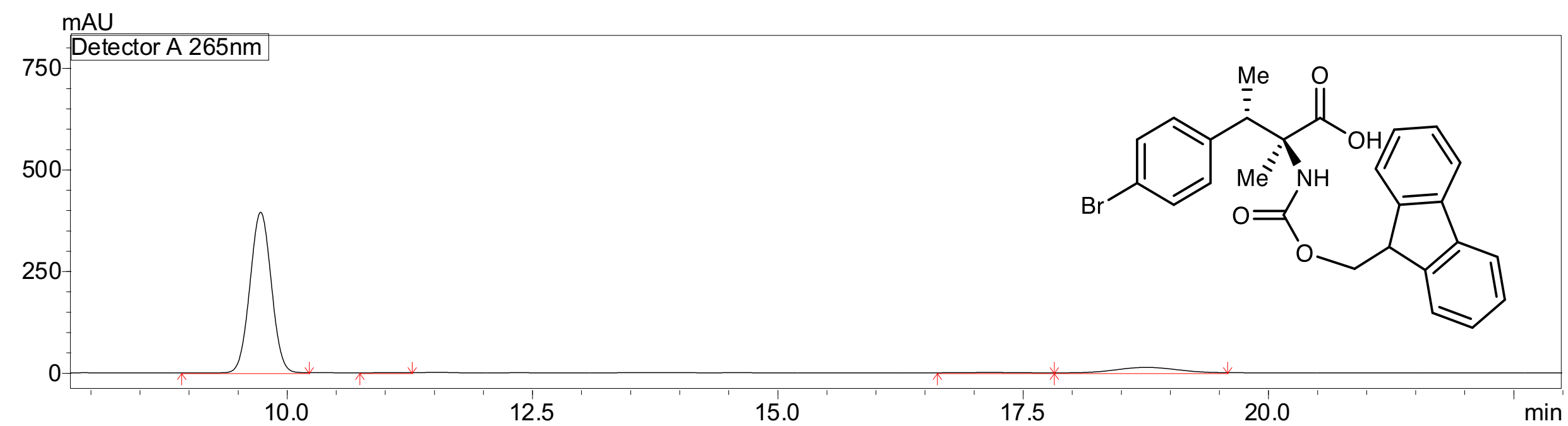

Peak#	Ret. Time	Height	Area	Area%
1	9.736	395267	5971442	90.585
2	11.095	501	8398	0.127
3	17.160	780	27963	0.424
4	18.760	13415	584282	8.863
Total		409963	6592085	100.000

(2*S*,3*S*)-2-amino-2-methyl-3-(naphthalen-2-yl)butanoic acid (3i)

Fmoc derivatization of (*rac*)-**3i**

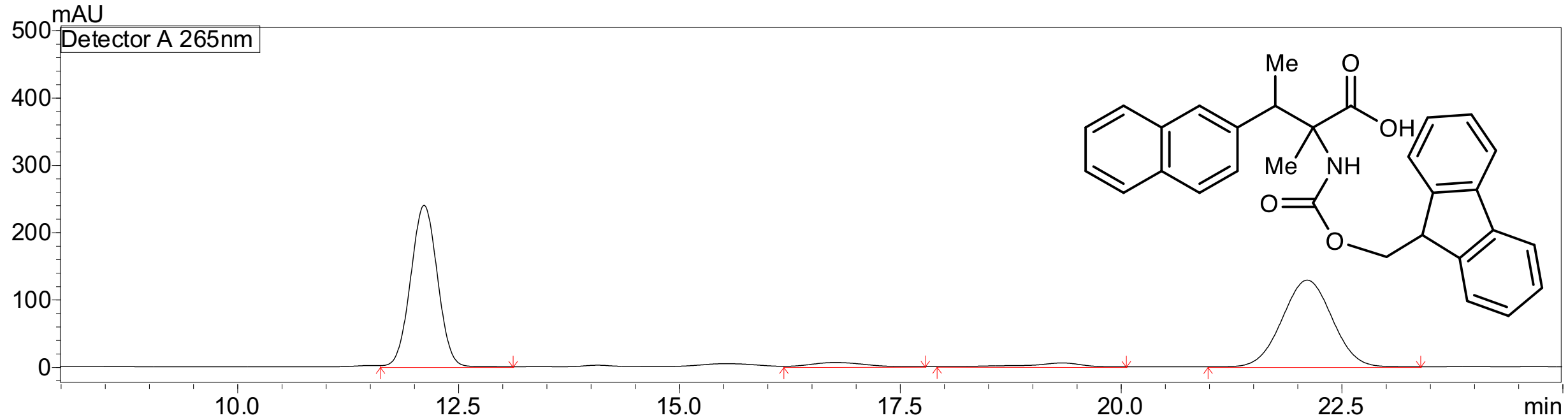

Peak Table	Compound	Group	Calibration Curve	
**Peak#**	**Ret. Time**	**Height**	**Area**	**Area%**
1	12.117	239751	5051489	47.700
2	16.774	6153	246757	2.330
3	19.339	5720	228054	2.153
4	22.114	128712	5063722	47.816
Total		380336	10590021	100.000

Fmoc derivatization of (2*S*,3*S*)-**3i**

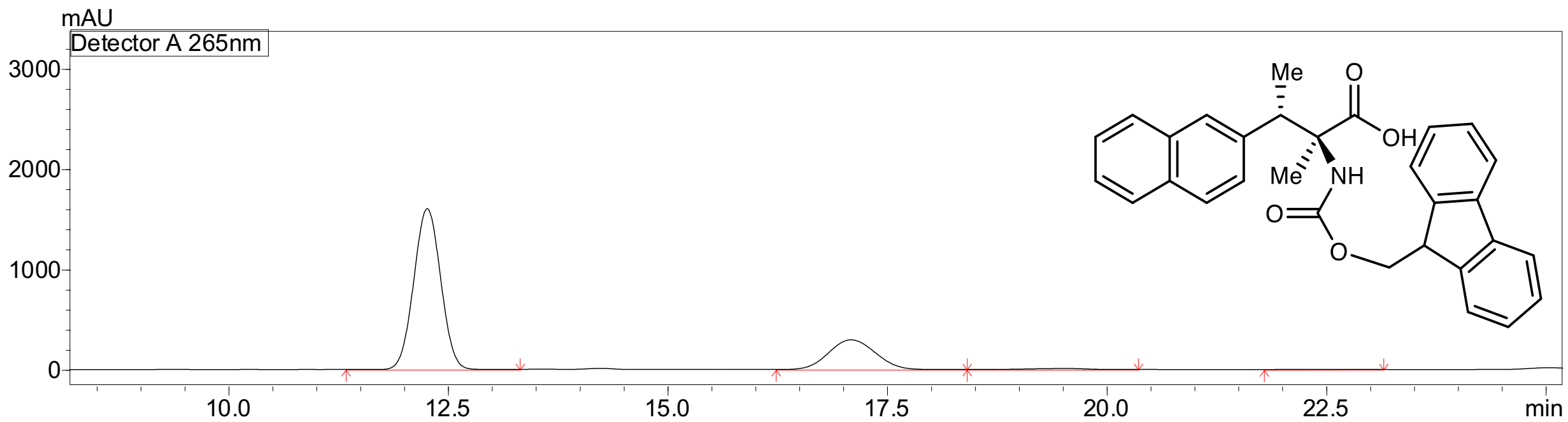

Peak Table	Compound	Group	Calibration Curve	
**Peak#**	**Ret. Time**	**Height**	**Area**	**Area%**
1	12.266	1603501	34117817	75.024
2	17.092	295954	10859927	23.881
3	19.529	10161	432329	0.951
4	22.499	1641	65912	0.145
Total		1911257	45475985	100.000

(2*S*,3*S*)-2-amino-2-methyl-3-phenylpentanoic acid (3j)

Fmoc derivatization of (*rac*)-**3j**

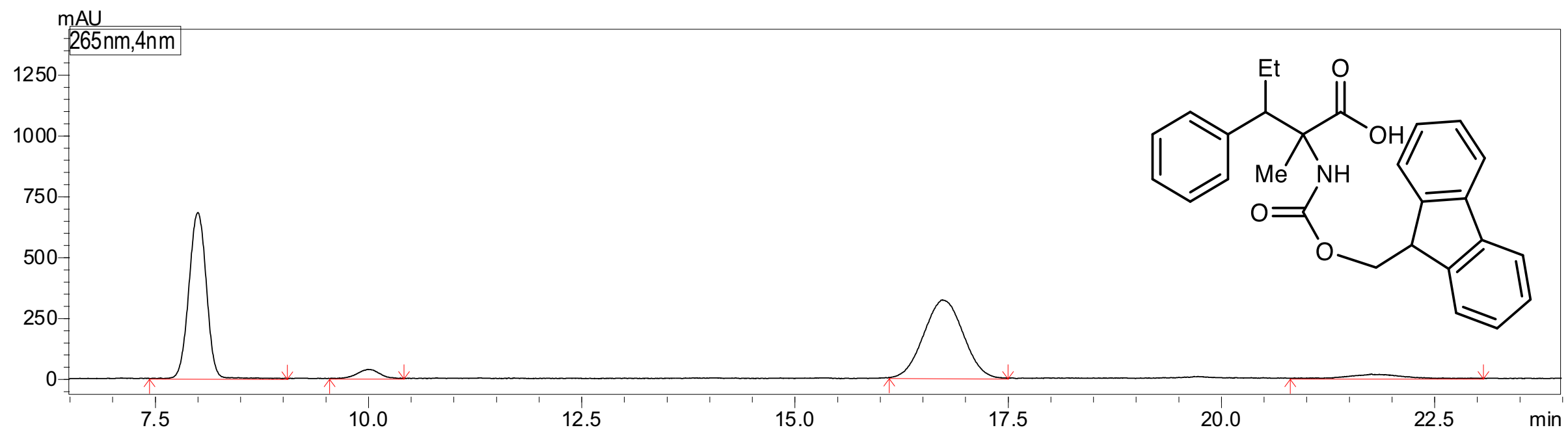

Peak Table	Compound	Group	Calibration Curve	
**Peak#**	**Ret. Time**	**Height**	**Area**	**Area%**
1	8.011	680850	9635350	45.560
2	10.007	36382	636874	3.011
3	16.742	319968	10243484	48.436
4	21.768	16984	632981	2.993
Total		1054184	21148690	100.000

Fmoc derivatization of (2*S*,3*S*)-**3j**

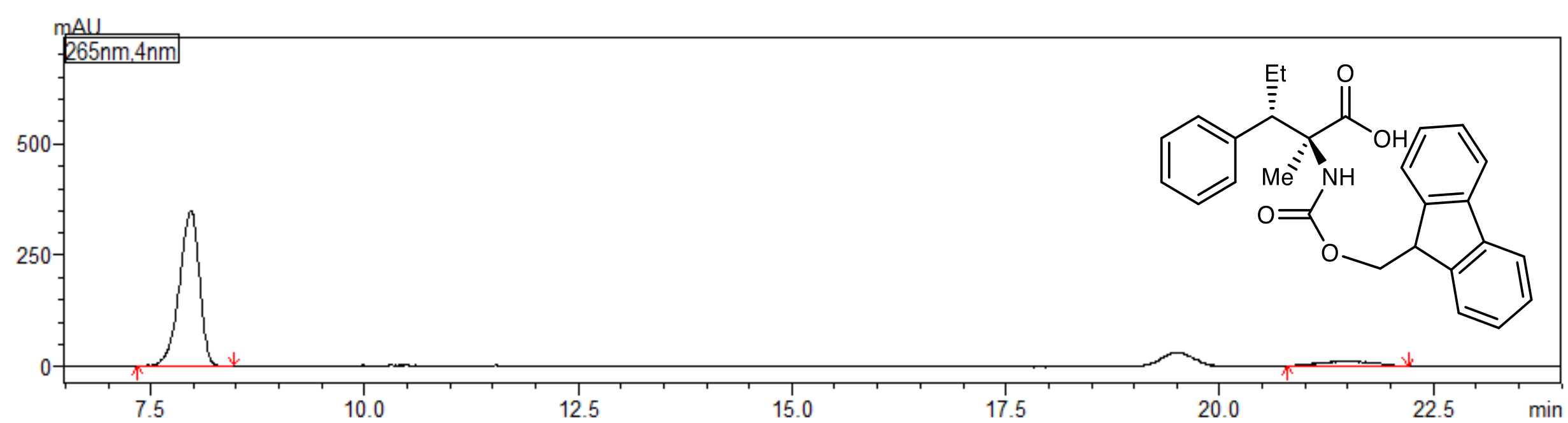

Peak Table	Compound	Group	Calibration Curve	
**Peak#**	**Ret. Time**	**Height**	**Area**	**Area%**
1	7.964	349836	5699024	90.828
2	21.497	12503	575517	9.172
Total		362338	6274541	100.000

(2*S*,3*S*)-2-amino-2-methyl-3-phenylhexanoic acid (3k)

Fmoc derivatization of (*rac*)-**3k**

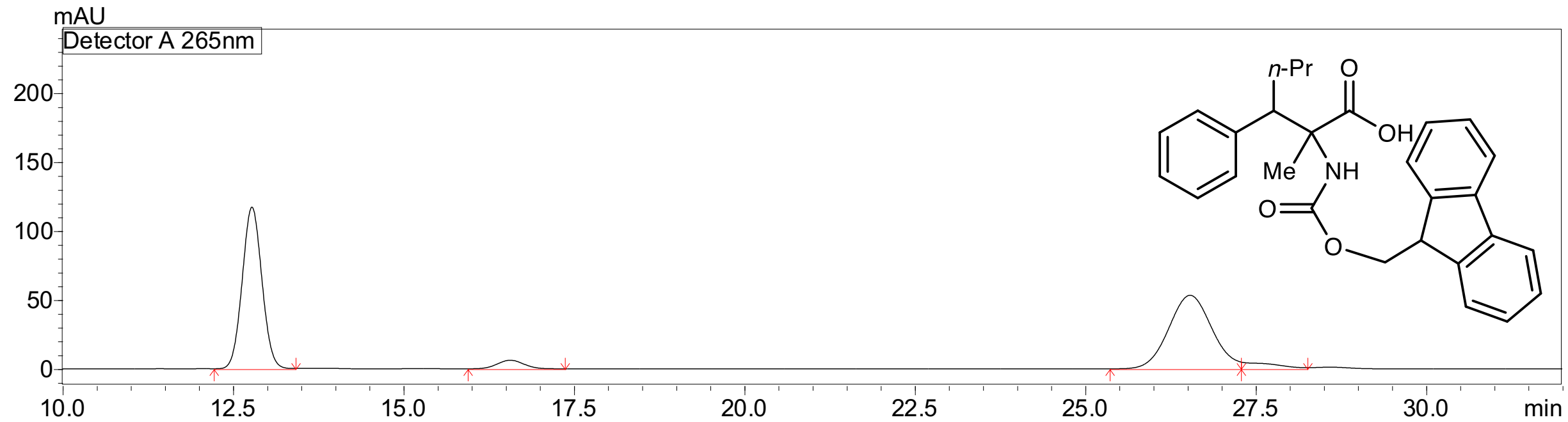

Peak Table | Compound | Group | Calibration Curve

Peak#	Ret. Time	Height	Area	Area%
1	12.780	117177	2357810	46.940
2	16.571	6184	166717	3.319
3	26.540	53337	2336351	46.513
4	27.296	4858	162167	3.228
Total		181556	5023045	100.000

Fmoc derivatization of (2*S*,3*S*)-**3k**

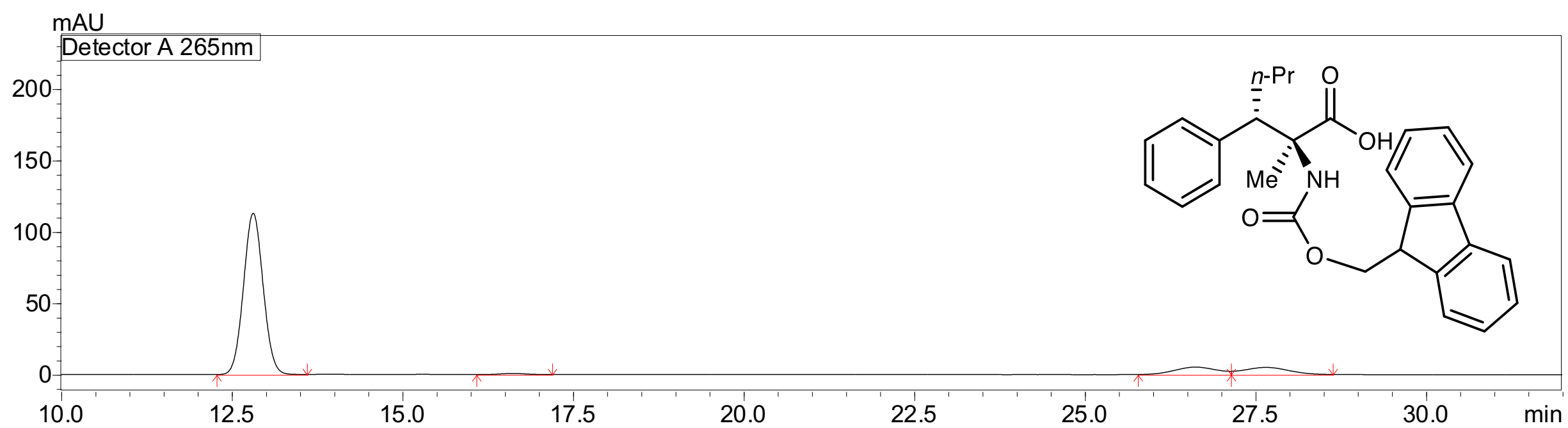

Peak Table | Compound | Group | Calibration Curve

Peak#	Ret. Time	Height	Area	Area%
1	12.816	112949	2258437	82.736
2	16.622	761	20244	0.742
3	26.623	5187	224109	8.210
4	27.654	4967	226909	8.313
Total		123863	2729698	100.000

(*S*)-2-amino-2-((*S*)-2,3-dihydro-1H-inden-1-yl)propanoic acid (3l)

Fmoc derivatization of (*rac*)-**3l**

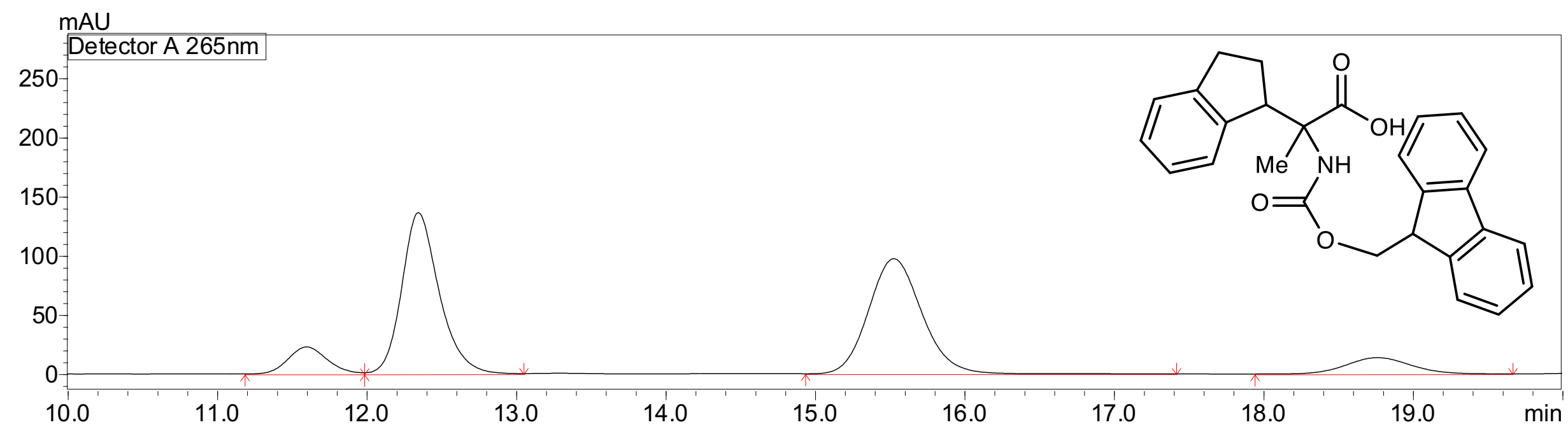

Peak#	Ret. Time	Height	Area	Area%
1	11.599	22855	416517	7.389
2	12.347	136350	2377740	42.183
3	15.528	97417	2425589	43.032
4	18.765	13776	416905	7.396
Total		270398	5636750	100.000

Fmoc derivatization of (2*S*,3*S*)-**3l**

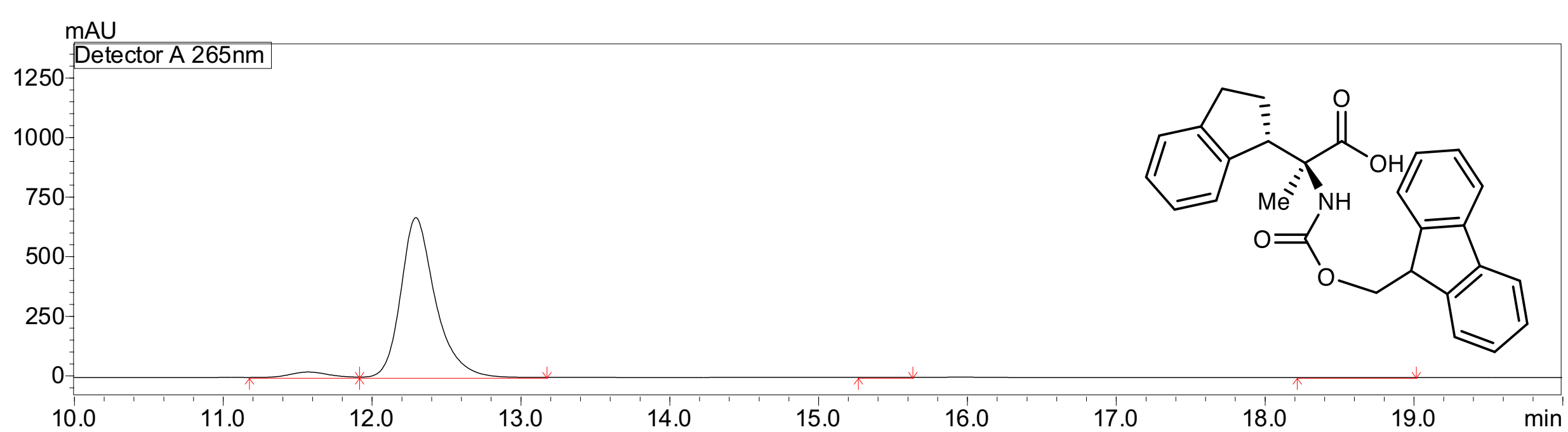

Peak#	Ret. Time	Height	Area	Area%
1	11.577	22892	406078	3.614
2	12.298	670912	10822357	96.319
3	15.509	280	5315	0.047
4	18.683	80	2189	0.019
Total		694164	11235939	100.000

(*S*)-2-amino-2-((*S*)-1,2,3,4-tetrahydronaphthalen-1-yl)propanoic acid (3m)

Fmoc derivatization of (*rac*)-**3m**

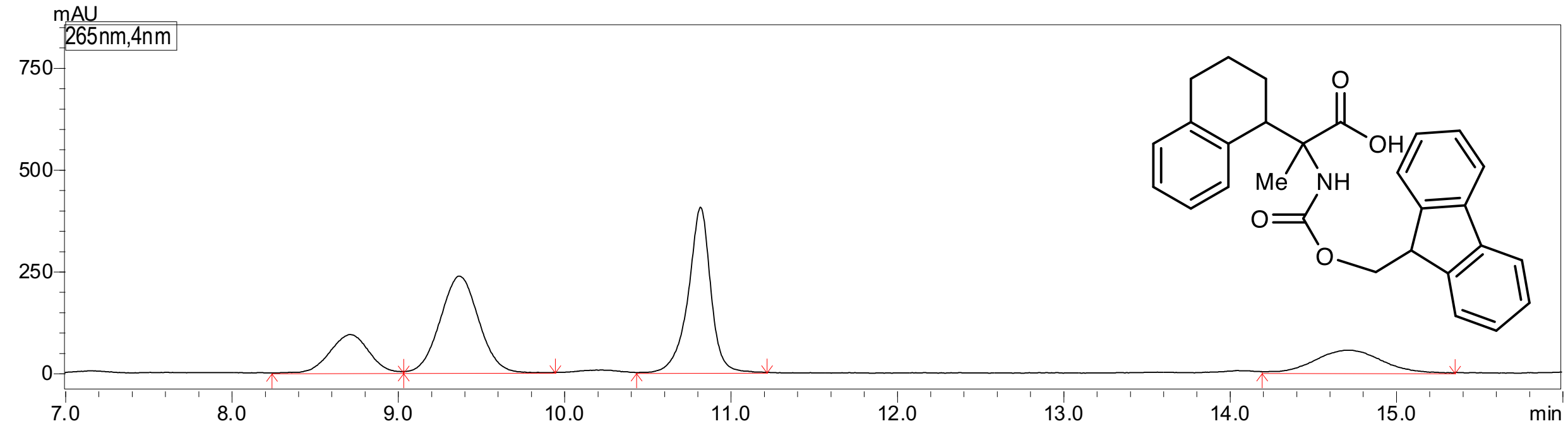

Peak#	Ret. Time	Height	Area	Area%
1	8.713	93763	1514618	14.273
2	9.370	236868	3789687	35.713
3	10.822	405615	3791808	35.733
4	14.732	55244	1515511	14.282
Total		791491	10611625	100.000

Fmoc derivatization of (2*S*,3*S*)-**3m**

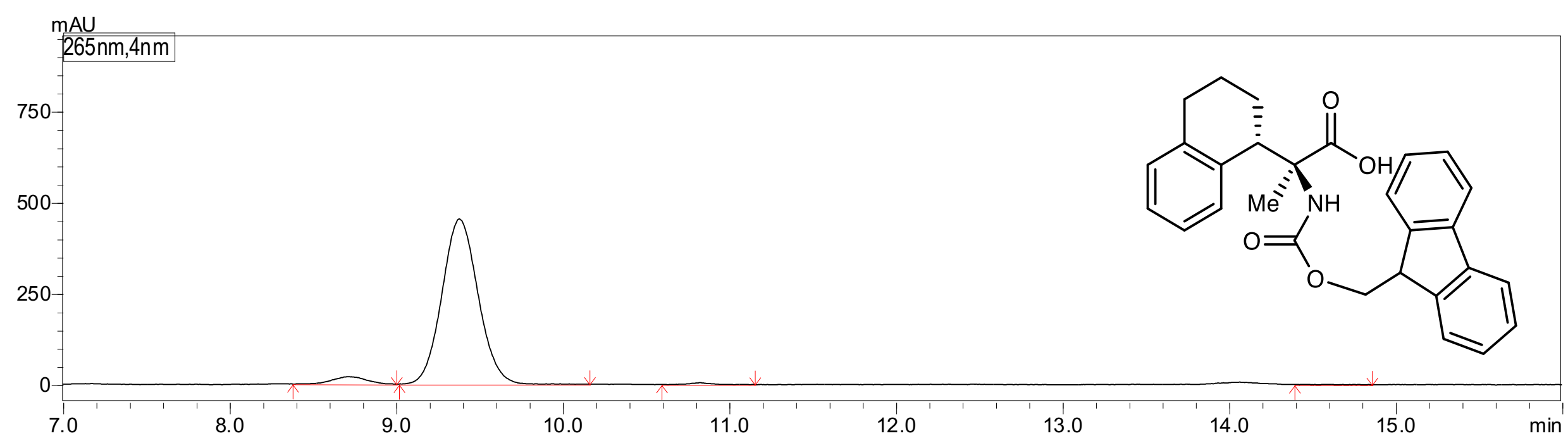

Peak#	Ret. Time	Height	Area	Area%
1	8.712	19962	275082	3.849
2	9.380	453174	6829339	95.569
3	10.824	5110	38309	0.536
4	14.436	602	3231	0.045
Total		478848	7145961	100.000

(*S*)-2-amino-2-((*S*)-chroman-4-yl)propanoic acid (3n)

Fmoc derivatization of (*rac*)-**3n**

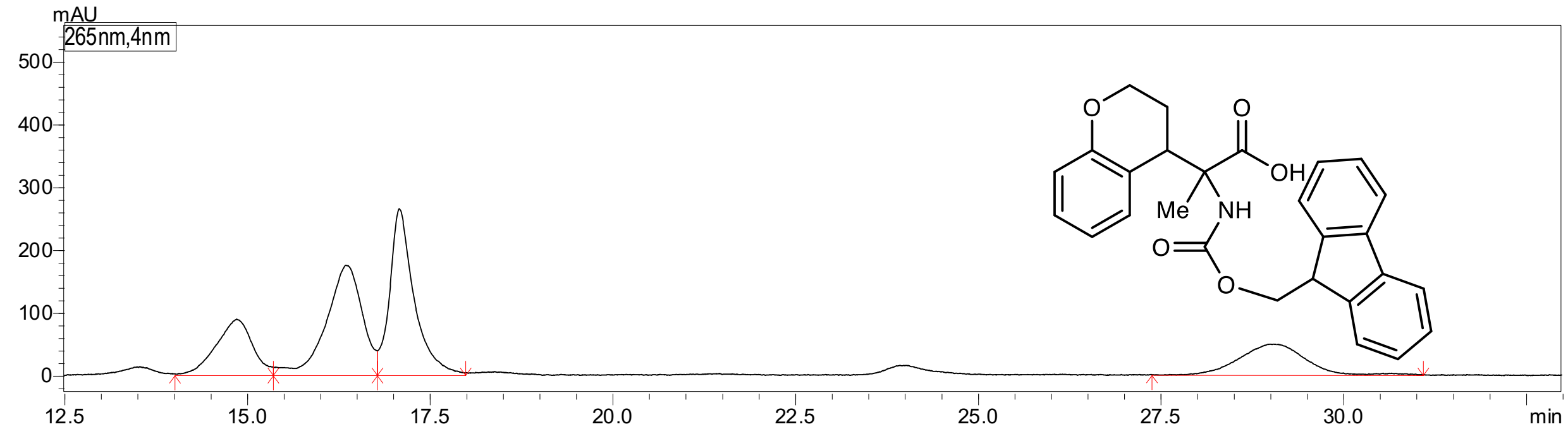

Peak#	Ret. Time	Height	Area	Area%
1	14.863	88443	3027718	16.797
2	16.358	174148	5940420	32.955
3	17.088	263897	6115248	33.925
4	29.054	48508	2942322	16.323
Total		574996	18025708	100.000

Fmoc derivatization of (2*S*,3*S*)-**3n**

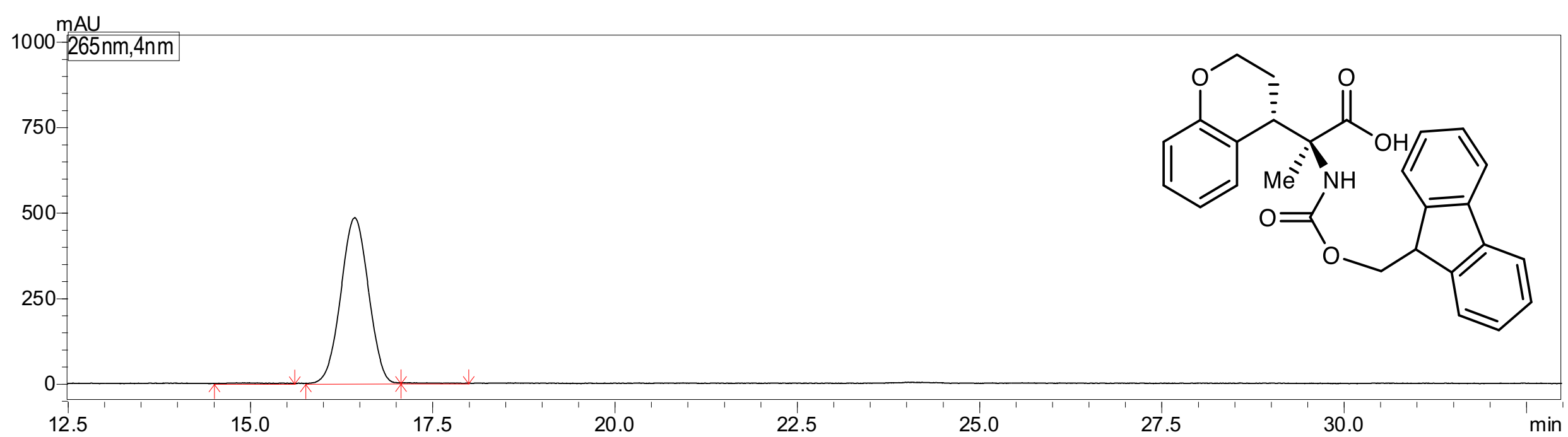

Peak#	Ret. Time	Height	Area	Area%
1	14.909	1848	49479	0.387
2	16.445	484354	12730526	99.462
3	17.084	1417	19347	0.151
Total		487619	12799352	100.000

(2*S*,3*S*)-2-amino-2-ethyl-3-phenylbutanoic acid (3o)

Fmoc derivatization of (*rac*)-**3o**

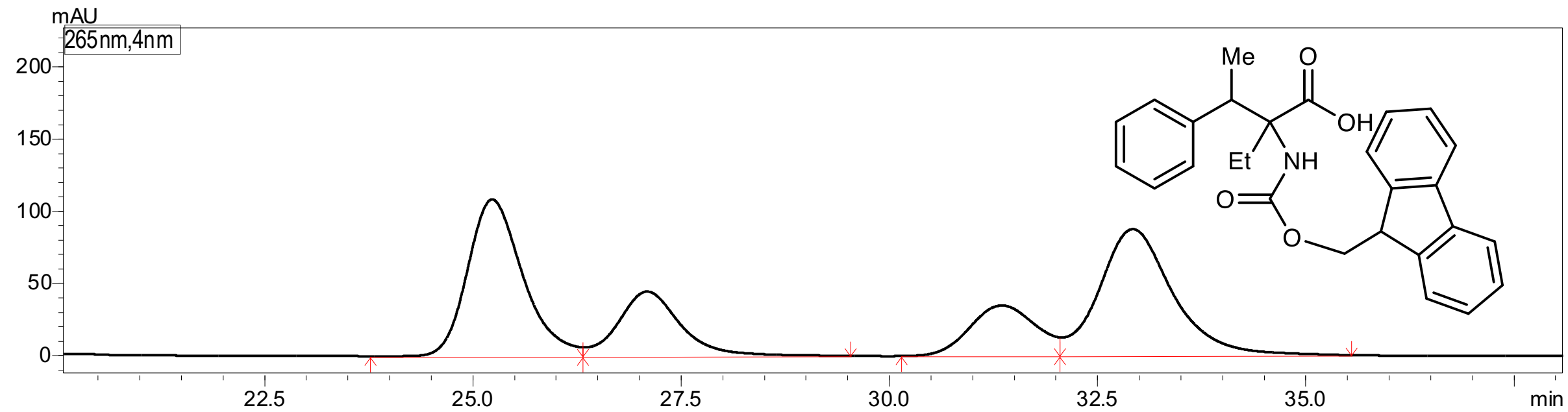

Peak#	Ret. Time	Height	Area	Area%
1	25.242	108635	5005108	34.178
2	27.104	44751	2330262	15.913
3	31.364	34892	1922461	13.128
4	32.935	87627	5386291	36.781
Total		275905	14644121	100.000

Fmoc derivatization of (2*S*,3*S*)-**3o**

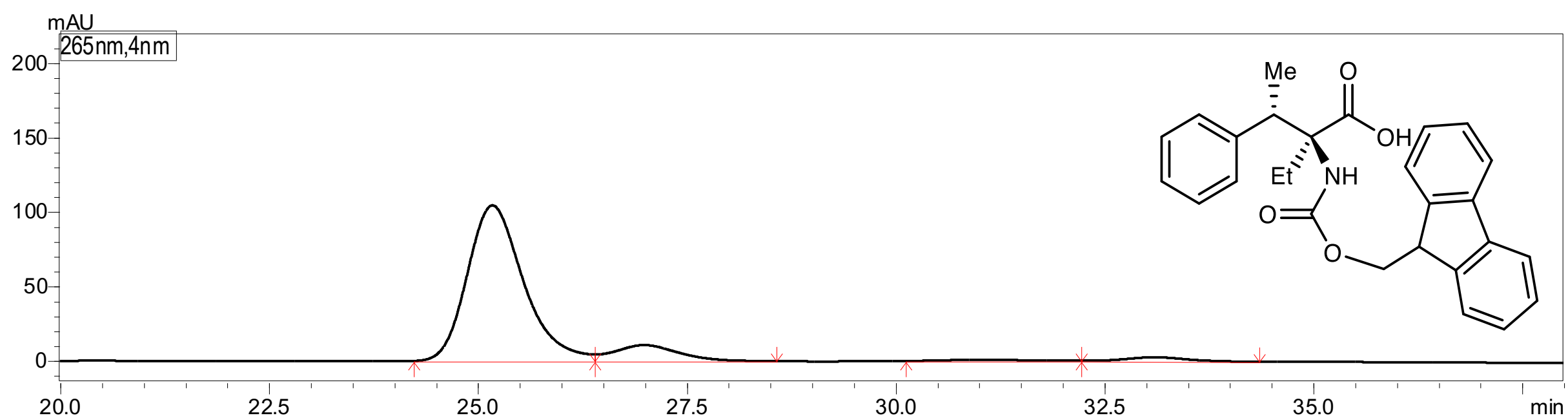

Peak#	Ret. Time	Height	Area	Area%
1	25.179	104482	4910400	85.965
2	26.996	10778	580373	10.160
3	31.081	853	66365	1.162
4	33.101	2920	154967	2.713
Total		119034	5712104	100.000

L-phenylalanine (5a)

Marfey's analysis of (*rac*)-**5a**

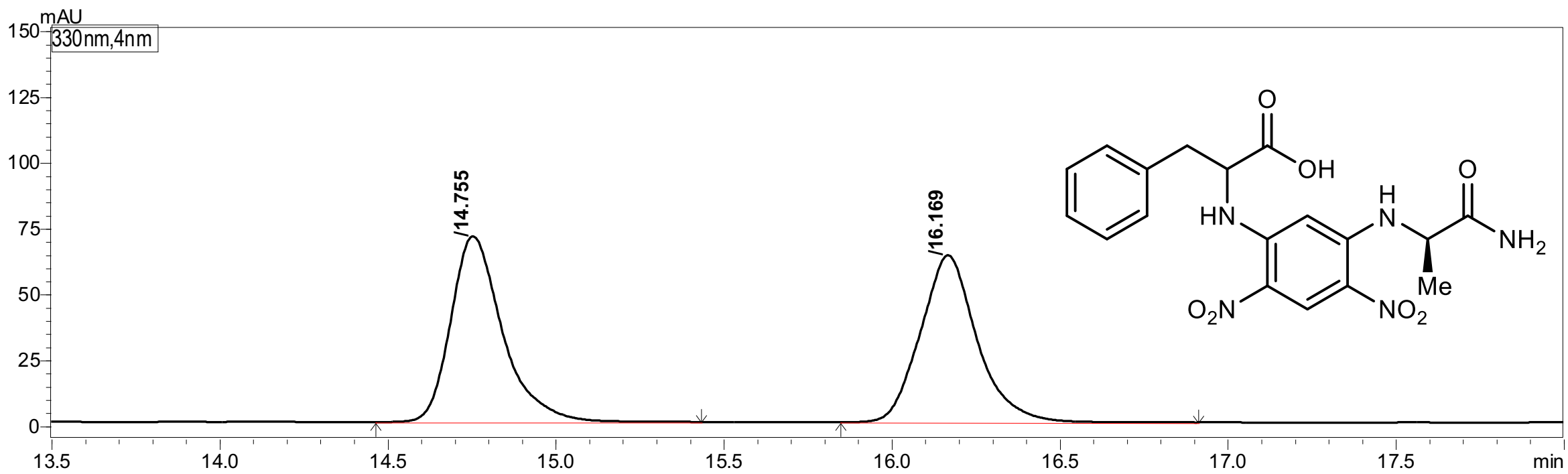

Peak#	Ret. Time	Area	Area%
1	14.757	12797	1.332
2	16.175	948270	98.668
Total		961066	100.000

Marfey's analysis of L-**5a**

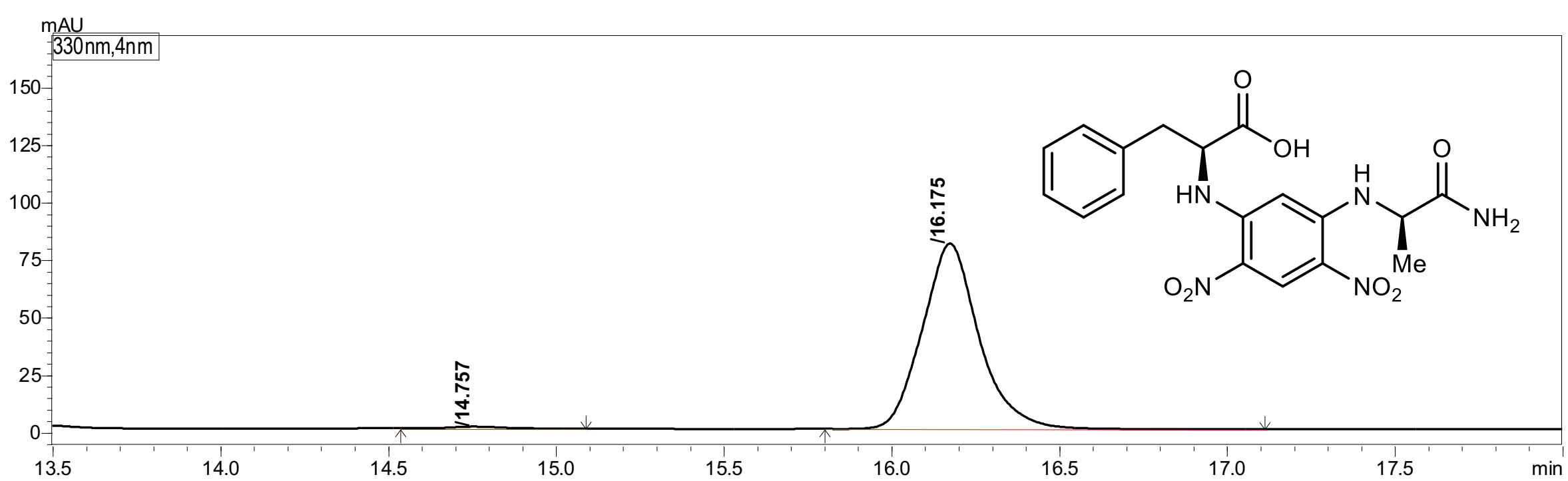

Peak#	Ret. Time	Area	Area%
1	14.755	755655	50.422
2	16.169	742997	49.578
Total		1498652	100.000

L-2-amino-3-(o-tolyl)propanoic acid (5b)

Marfey's analysis of (*rac*)-**5b**

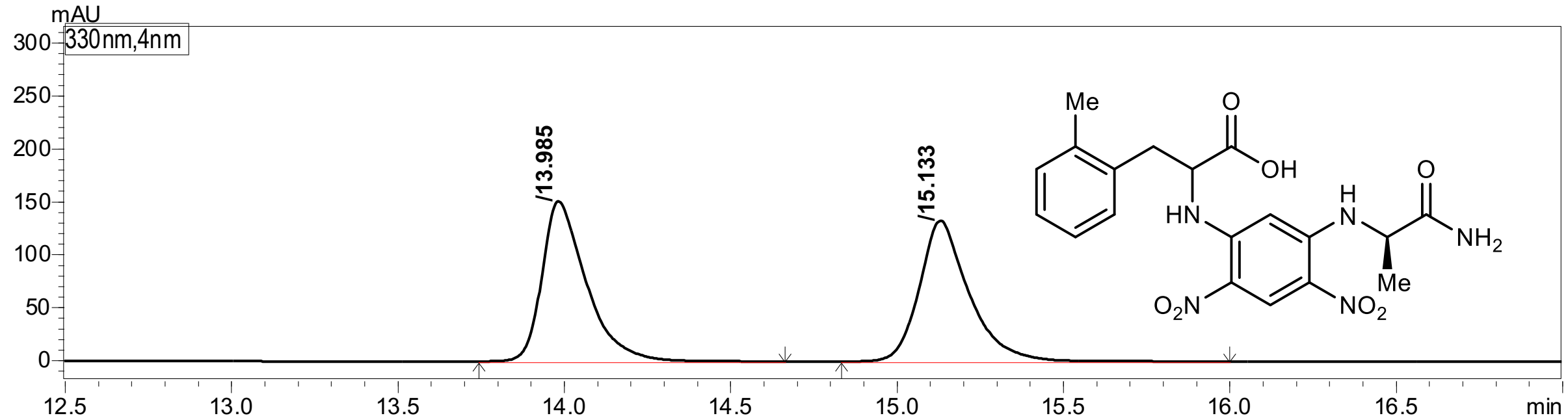

Peak#	Ret. Time	Area	Area%
1	13.985	1396923	50.328
2	15.133	1378703	49.672
Total		2775627	100.000

Marfey's analysis of L-**5b**

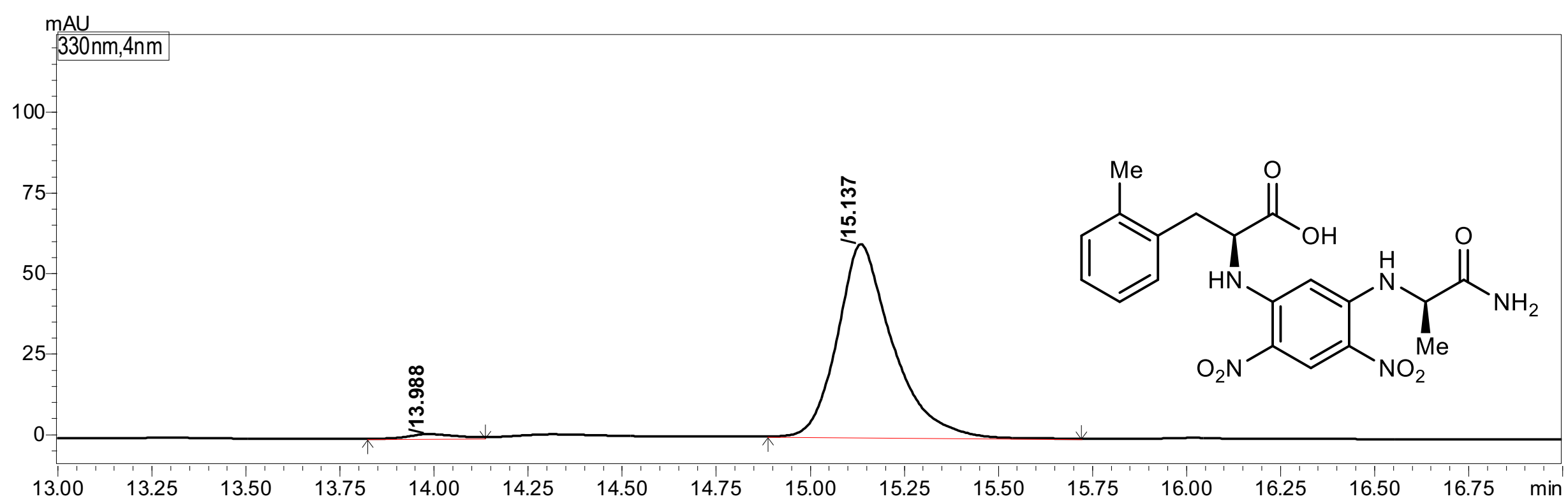

Peak#	Ret. Time	Height	Area	Area%
1	13.988	1318	10688	1.726
2	15.137	59741	608730	98.274
Total		61059	619418	100.000

L-2-amino-3-(m-tolyl)propanoic acid (5c)

Marfey's analysis of (*rac*)-**5c**

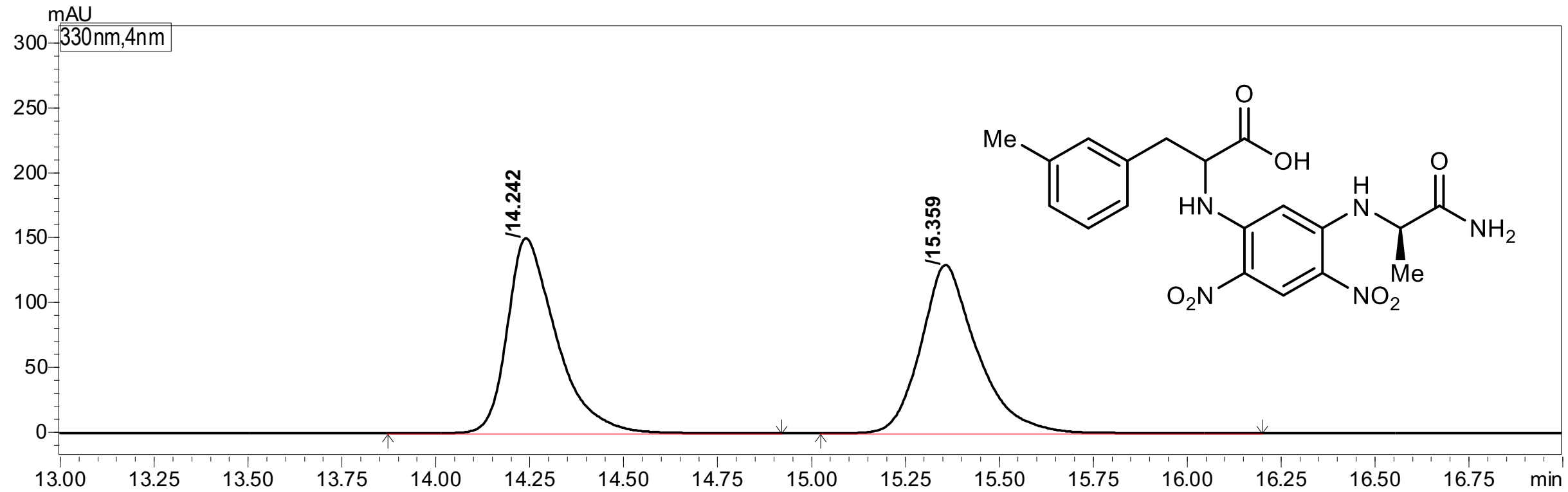

Peak#	Ret. Time	Height	Area	Area%
1	14.242	149664	1367411	50.201
2	15.359	129628	1356459	49.799
Total		279292	2723870	100.000

Marfey's analysis of L-**5c**

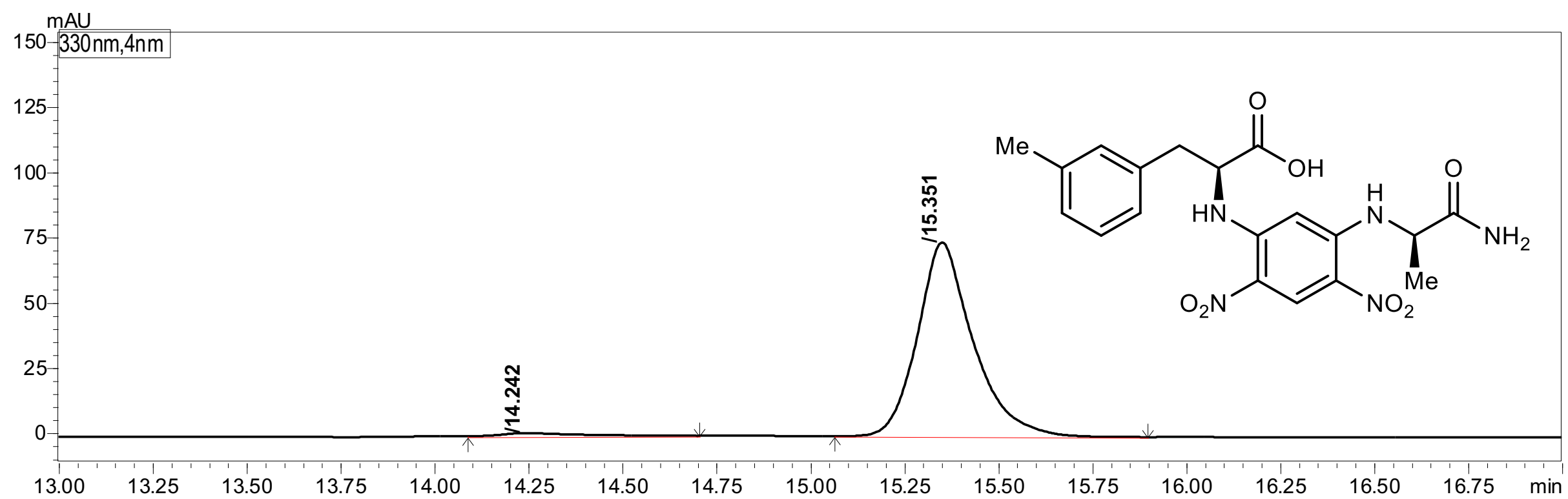

Peak#	Ret. Time	Height	Area	Area%
1	14.242	1322	19856	2.518
2	15.351	74338	768603	97.482
Total		75660	788460	100.000

L-2-amino-3-(p-tolyl)propanoic acid (5d)

Marfey's analysis of (*rac*)-**5d**

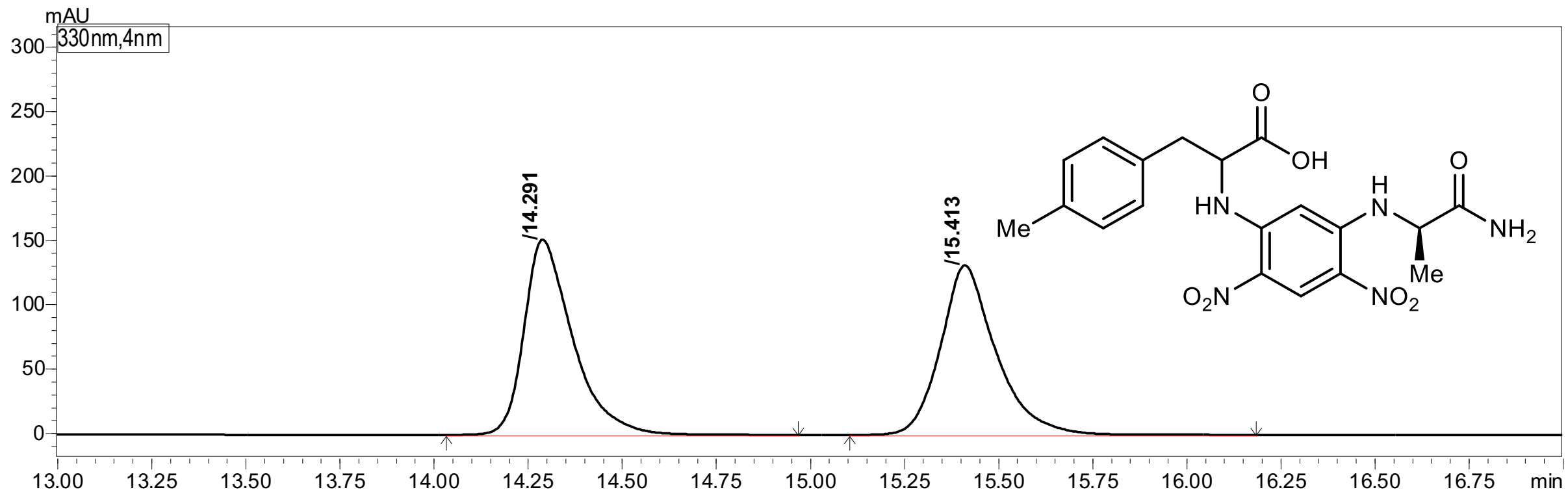

Peak#	Ret. Time	Height	Area	Area%
1	14.291	151087	1365345	50.234
2	15.413	131613	1352631	49.766
Total		282700	2717976	100.000

Marfey's analysis of L-**5d**

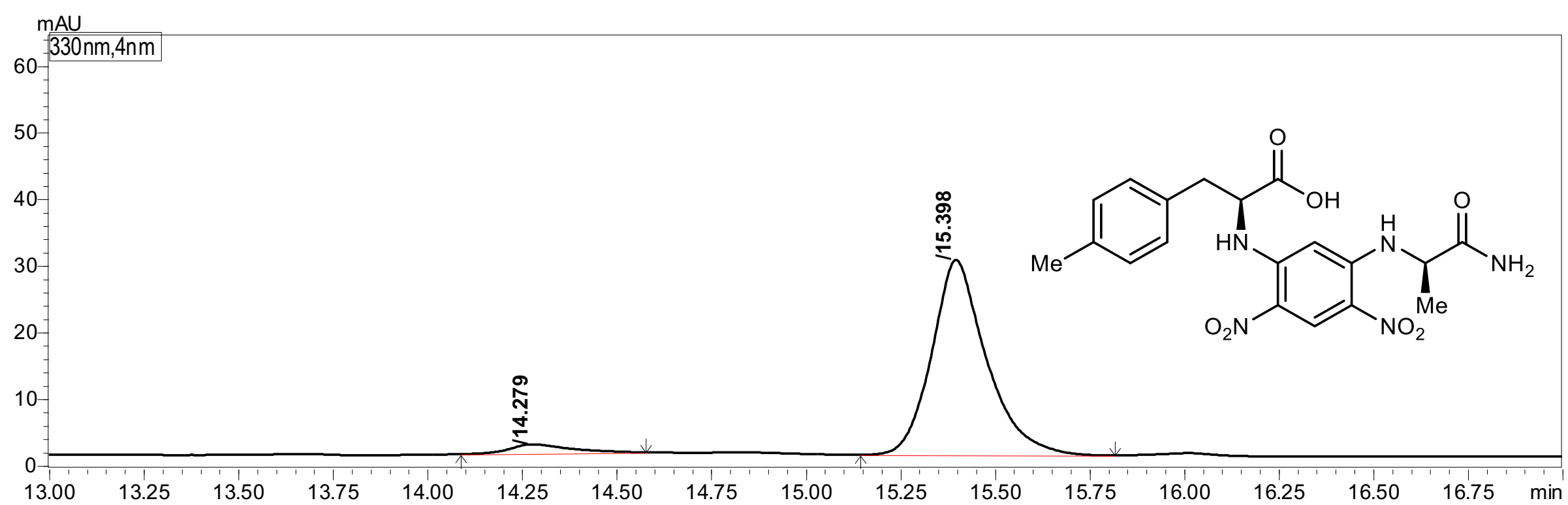

Peak#	Ret. Time	Height	Area	Area%
1	14.279	1367	15089	4.875
2	15.398	29241	294448	95.125
Total		30608	309537	100.000

L-2-amino-3-(4-methoxyphenyl)propanoic acid (5e)

Marfey's analysis of (*rac*)-**5e**

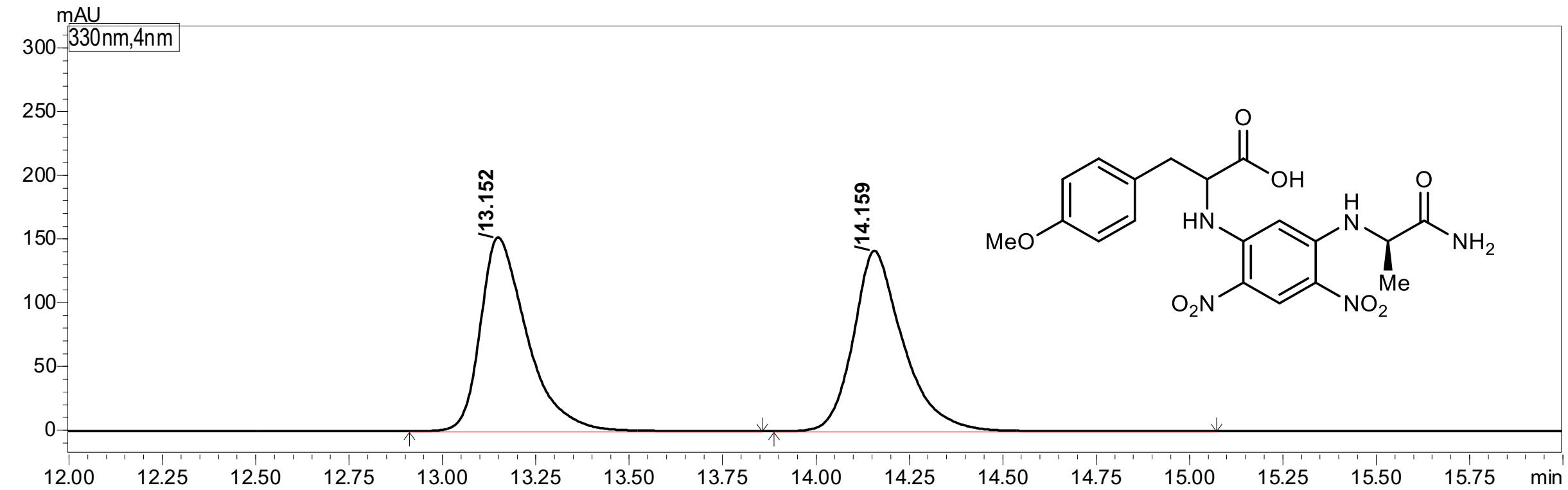

Peak#	Ret. Time	Height	Area	Area%
1	13.152	151698	1317957	50.057
2	14.159	141585	1314967	49.943
Total		293283	2632923	100.000

Marfey's analysis of L-**5e**

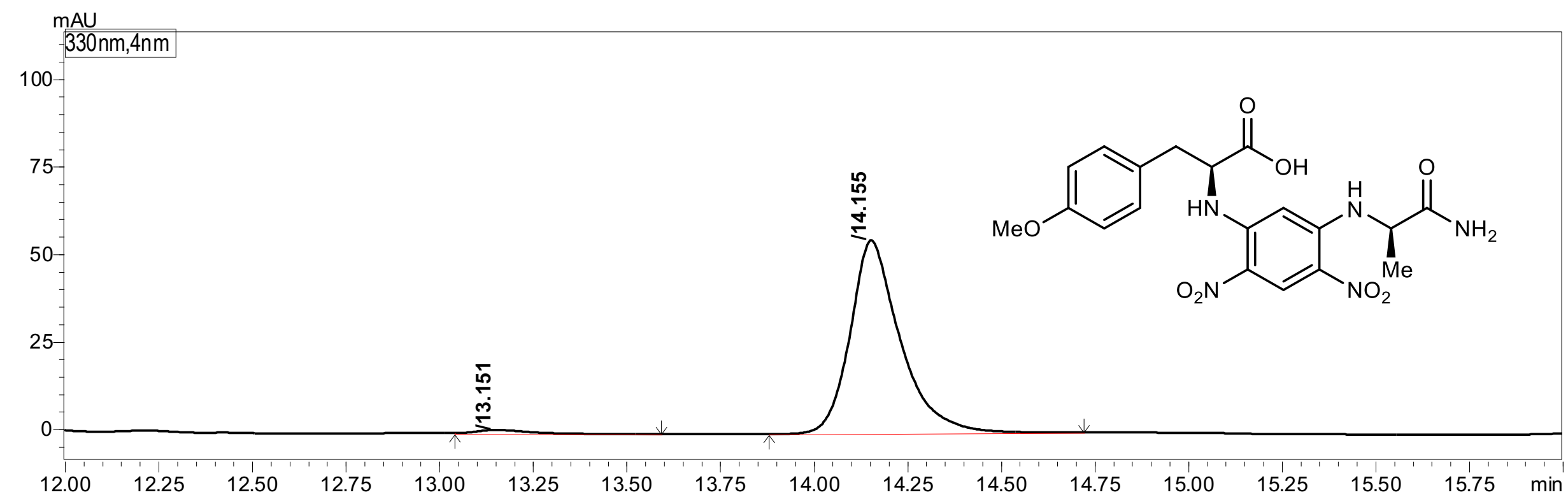

Peak#	Ret. Time	Height	Area	Area%
1	13.151	1134	11228	2.137
2	14.155	55037	514258	97.863
Total		56171	525486	100.000

L-2-amino-3-(4-(trifluoromethyl)phenyl)propanoic acid (5f)

Marfey's analysis of (*rac*)-**5f**

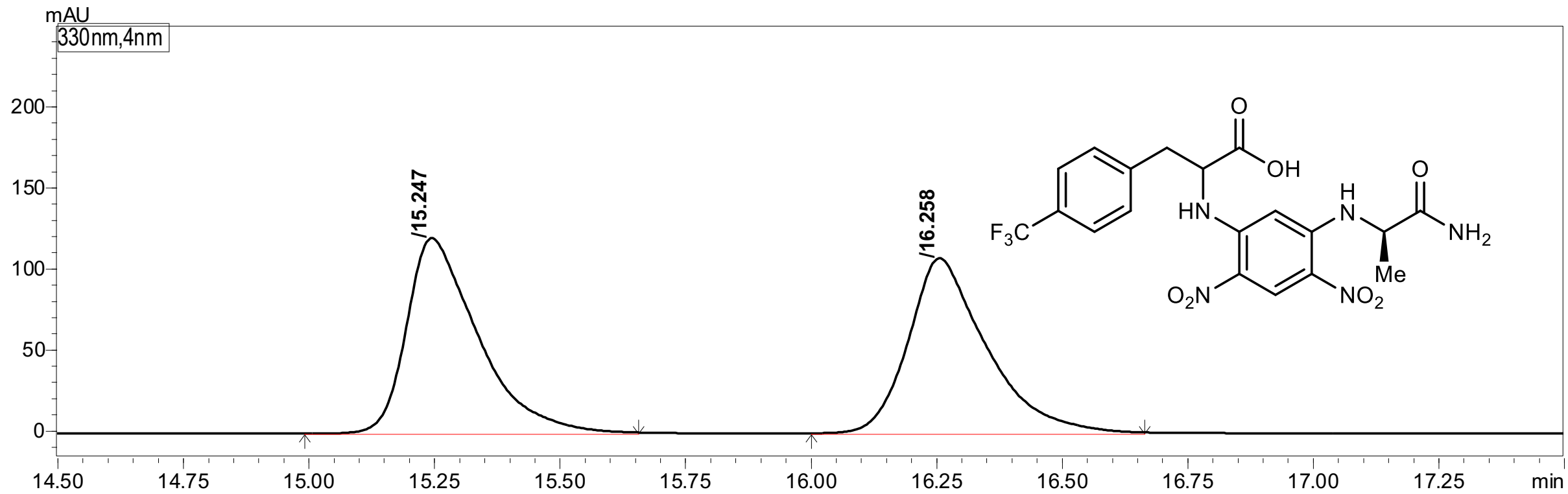

Peak#	Ret. Time	Height	Area	Area%
1	15.247	120424	1195370	50.415
2	16.258	107750	1175674	49.585
Total		228174	2371044	100.000

Marfey's analysis of L-**5f**

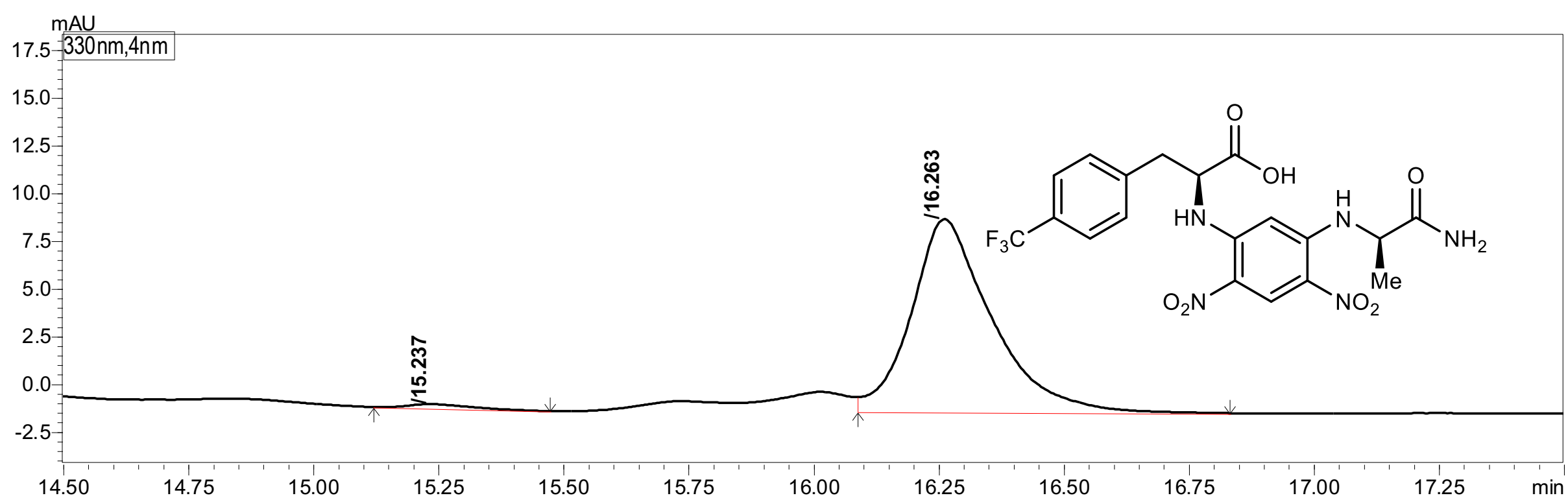

Peak#	Ret. Time	Height	Area	Area%
1	15.237	220	1675	1.461
2	16.263	10110	112983	98.539
Total		10330	114657	100.000

L-2-amino-3-(naphthalen-2-yl)propanoic acid (5g)

Marfey's analysis of (*rac*)-**5g**

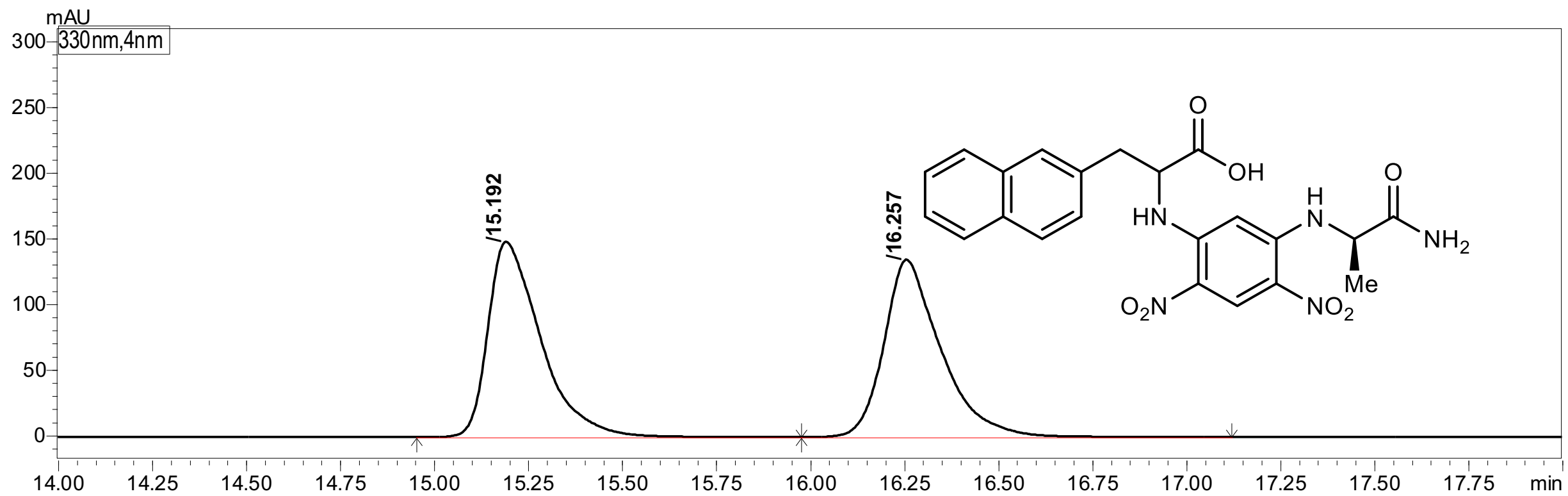

Peak#	Ret. Time	Height	Area	Area%
1	15.192	148309	1451771	50.468
2	16.257	134748	1424838	49.532
Total		283057	2876609	100.000

Marfey's analysis of **L-5g**

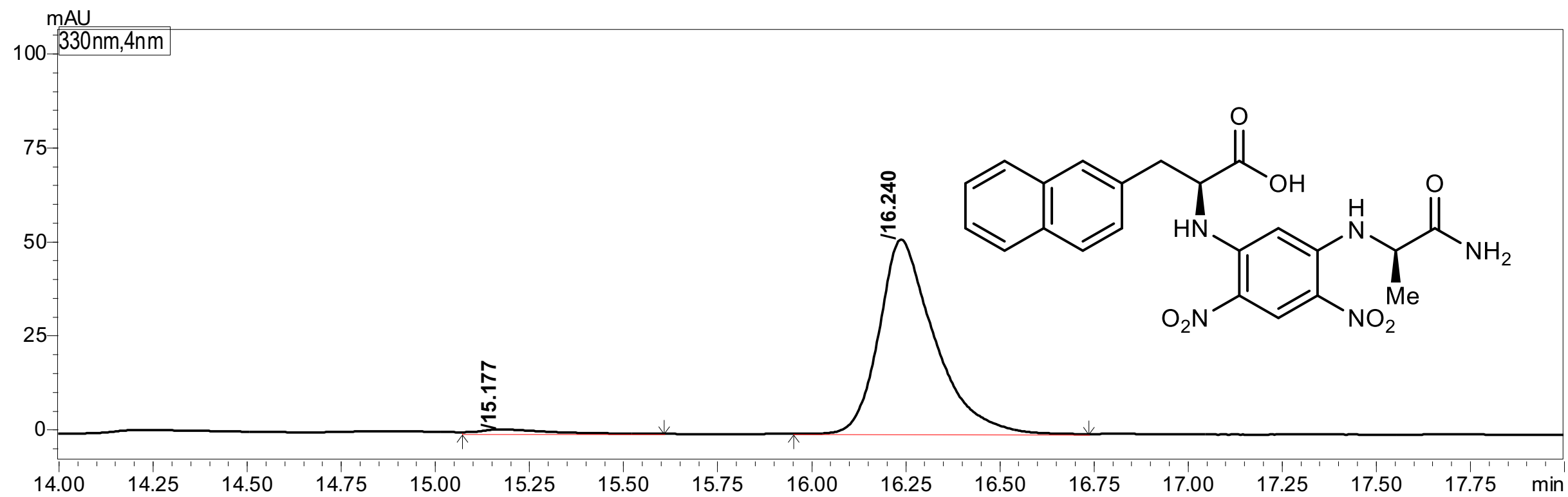

Peak#	Ret. Time	Height	Area	Area%
1	15.177	1182	14925	2.741
2	16.240	51697	529477	97.259
Total		52878	544402	100.000

(*S*)-2-amino-2-methyl-3-phenylpropanoic acid (6a)

Fmoc derivatization of (*rac*)-**6a**

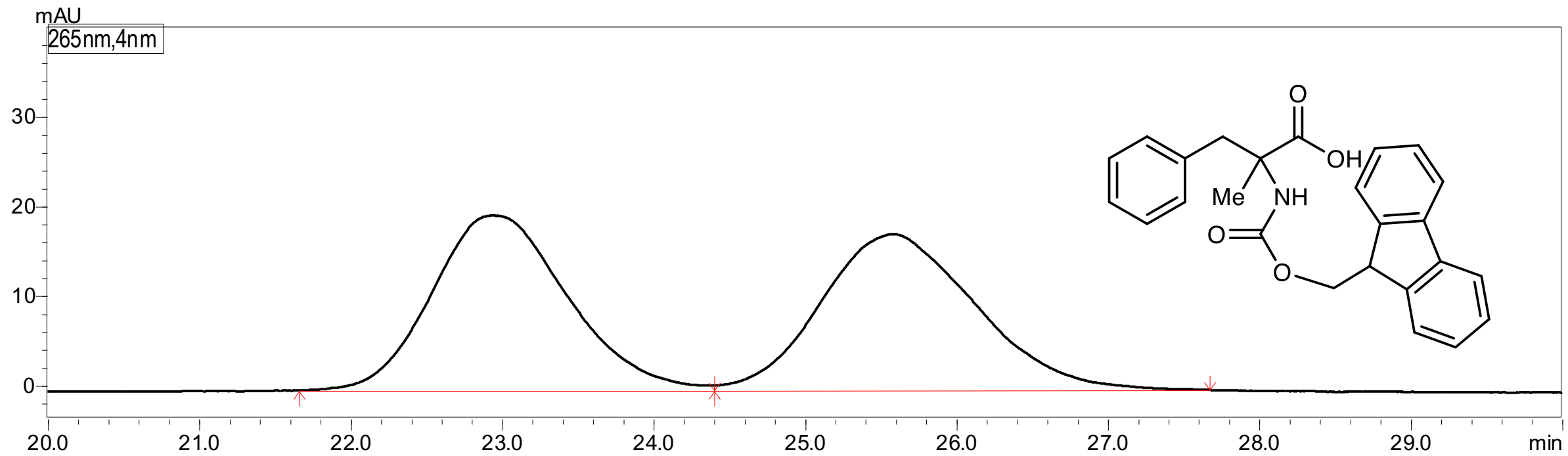

Peak Table | Compound | Group | Calibration Curve

Peak#	Ret. Time	Height	Area	Area%
1	22.937	19527	1214229	49.599
2	25.592	17415	1233860	50.401
Total		36943	2448089	100.000

Fmoc derivatization of (*S*)-**6a**

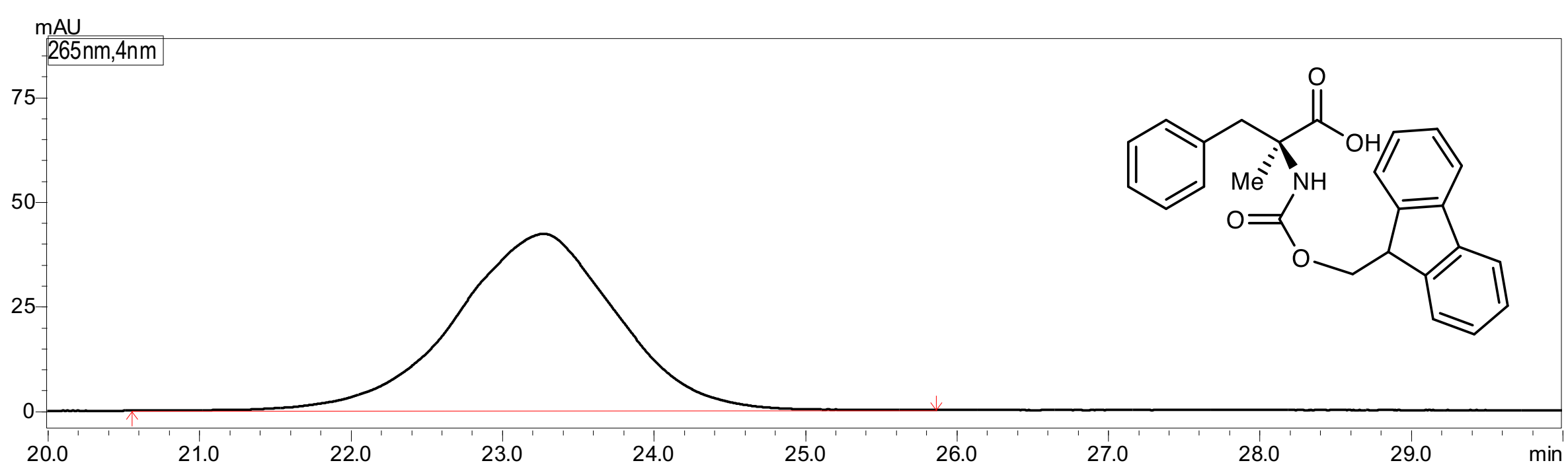

Peak Table | Compound | Group | Calibration Curve

Peak#	Ret. Time	Height	Area	Area%
1	23.277	42162	3114698	100.000
Total		42162	3114698	100.000

(*S*)-2-amino-3-(4-fluorophenyl)-2-methylpropanoic acid (6b)

Fmoc-Cl protection of (*rac*)-**6b**

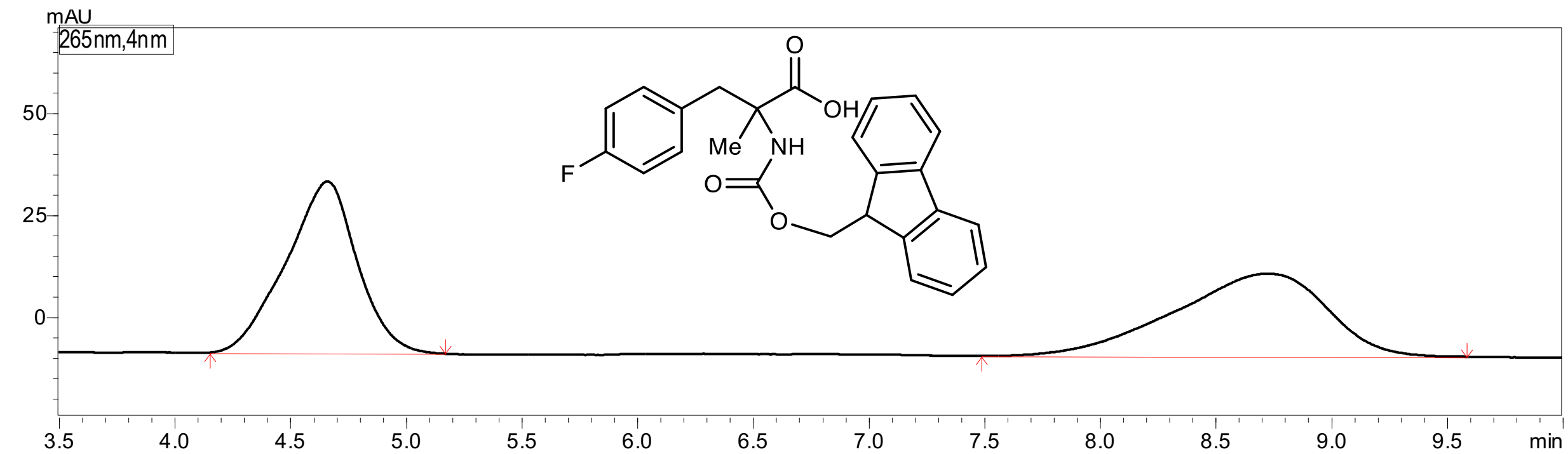

Peak Table | Compound | Group | Calibration Curve

Peak#	Ret. Time	Height	Area	Area%
1	4.663	42133	902845	49.354
2	8.733	20316	926478	50.646
Total		62450	1829322	100.000

Fmoc-Cl protection of (*S*)-**6b**

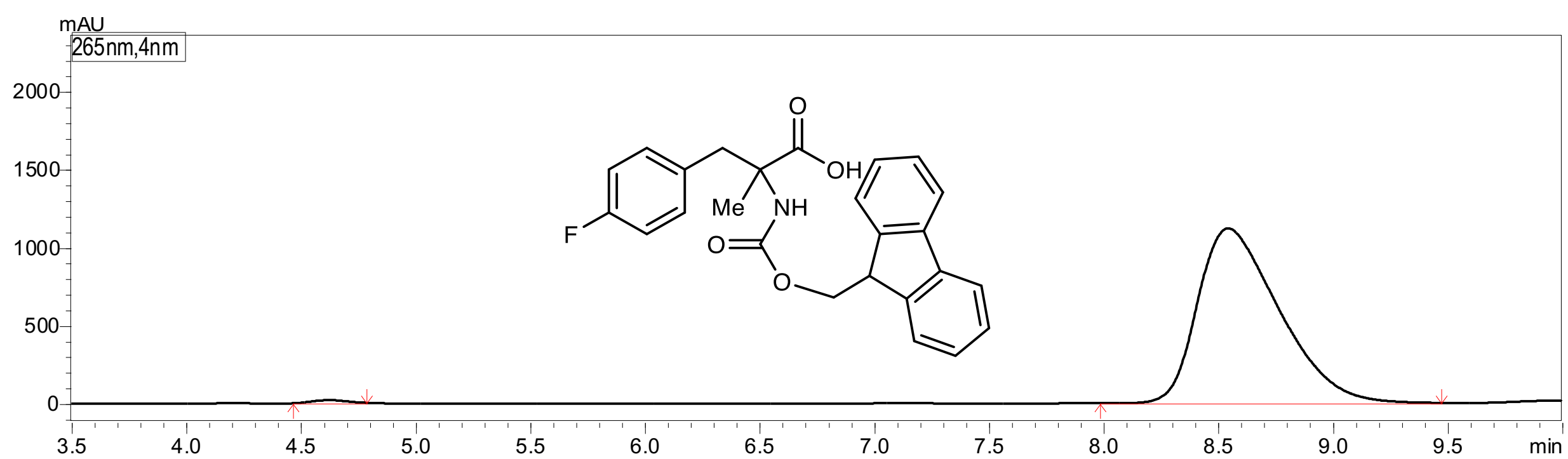

Peak Table | Compound | Group | Calibration Curve

Peak#	Ret. Time	Height	Area	Area%
1	4.625	20708	225034	0.810
2	8.545	1118621	27567862	99.190
Total		1139329	27792896	100.000

(*S*)-2-Amino-2-methyl-3-(4-(trifluoromethyl)phenyl)propanoic acid (6c)

Fmoc derivatization of (*rac*)-**6c**

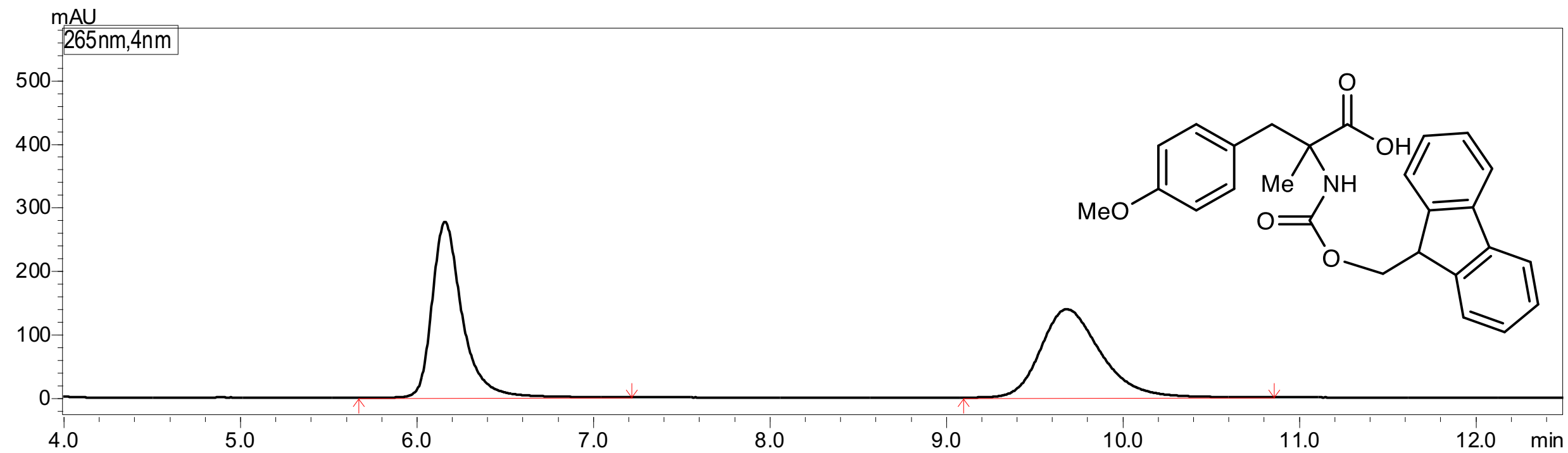

Peak#	Ret. Time	Height	Area	Area%
1	6.164	276217	3260767	49.910
2	9.688	138949	3272508	50.090
Total		415166	6533275	100.000

Fmoc derivatization of (*S*)-**6c**

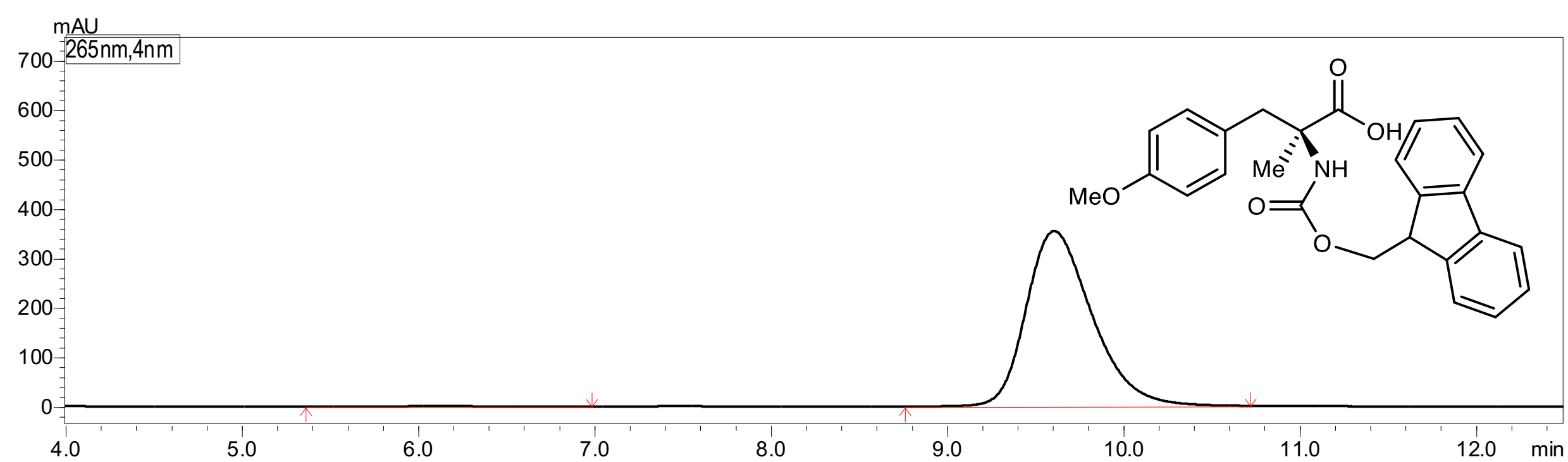

Peak#	Ret. Time	Height	Area	Area%
1	6.122	1548	29794	0.322
2	9.610	354039	9212289	99.678
Total		355587	9242083	100.000

(*S*, *E*)-2-amino-2-methyl-6-phenylhex-5-enoic acid (7a)

Fmoc derivatization of (*rac*)-**7a**

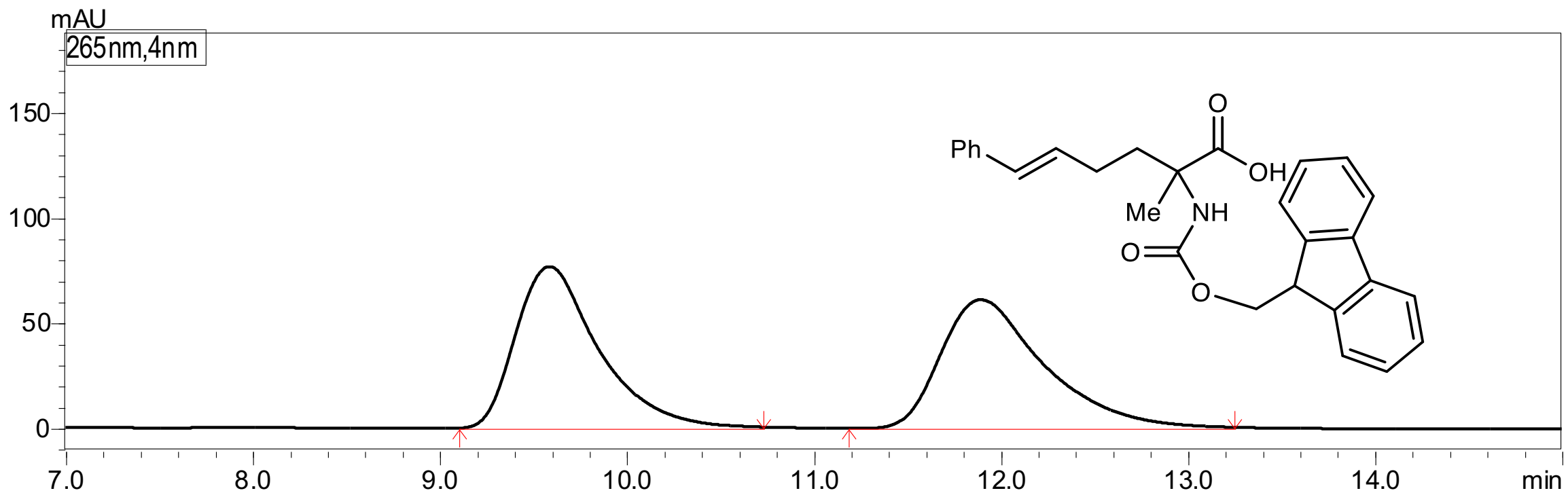

Peak Table | Compound | Group | Calibration Curve

Peak#	Ret. Time	Height	Area	Area%
1	9.588	76869	2381926	50.268
2	11.895	61091	2356512	49.732
Total		137960	4738438	100.000

Fmoc derivatization of (*S*,*E*)-**7a**

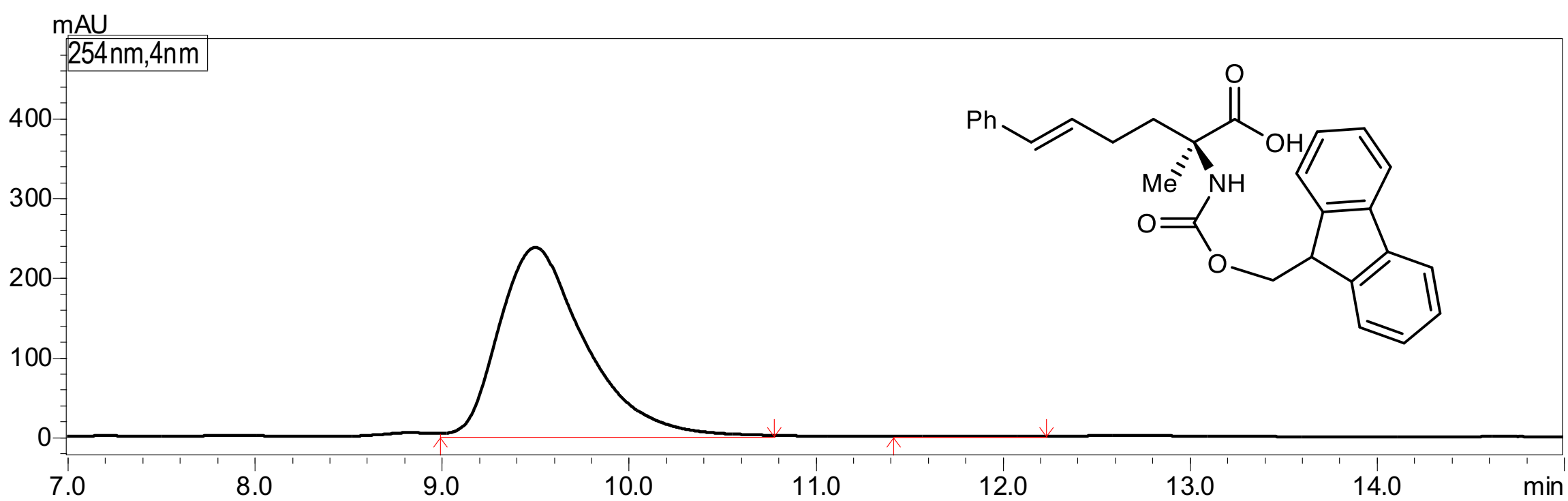

Peak Table | Compound | Group | Calibration Curve

Peak#	Ret. Time	Height	Area	Area%
1	9.506	236949	7644571	99.902
2	11.734	320	7473	0.098
Total		237269	7652044	100.000

XIII. Computational Studies

Density functional theory (DFT) and local coupled-cluster (DLPNO-CCSD(T)) calculations of the formation of the ring opened homoallyl radical 8a

To investigate the pathway of the formation of the ring-opened homoallyl radical **8a**, we performed DFT and DLPNO-CCSD(T) calculations at the DLPNO-CCSD(T)/def2-TZVPP/SMD(water)//M06-2X/def2-TZVP level of theory using the Gaussian 16 software package[21] and ORCA 6.0.1 software package.[22] Geometries were optimized in the gas phase using the (U)M06-2X functional[23] with the def2-TZVP basis set. Vibrational frequency calculations were performed at the same level of theory as geometry optimization to confirm that the obtained transition state connects its respective minima. Single-point energies were calculated with the DLPNO-CCSD(T) method[24,25,26] with the "tightPNO" keyword and the def2-TZVPP basis set. Solvation effects were considered in the single point energy calculations using the SMD model[27] in water. Our calculations show that the homoallyl radical **8a** is generated by the ring opening of the cyclopropyl substituted benzyl radical **8b** via transition state **TS1** ($\Delta G^{\ddagger}$ = 13.5 kcal/mol). Radical **8a** is 5.1 kcal/mol less stable than **8b**.

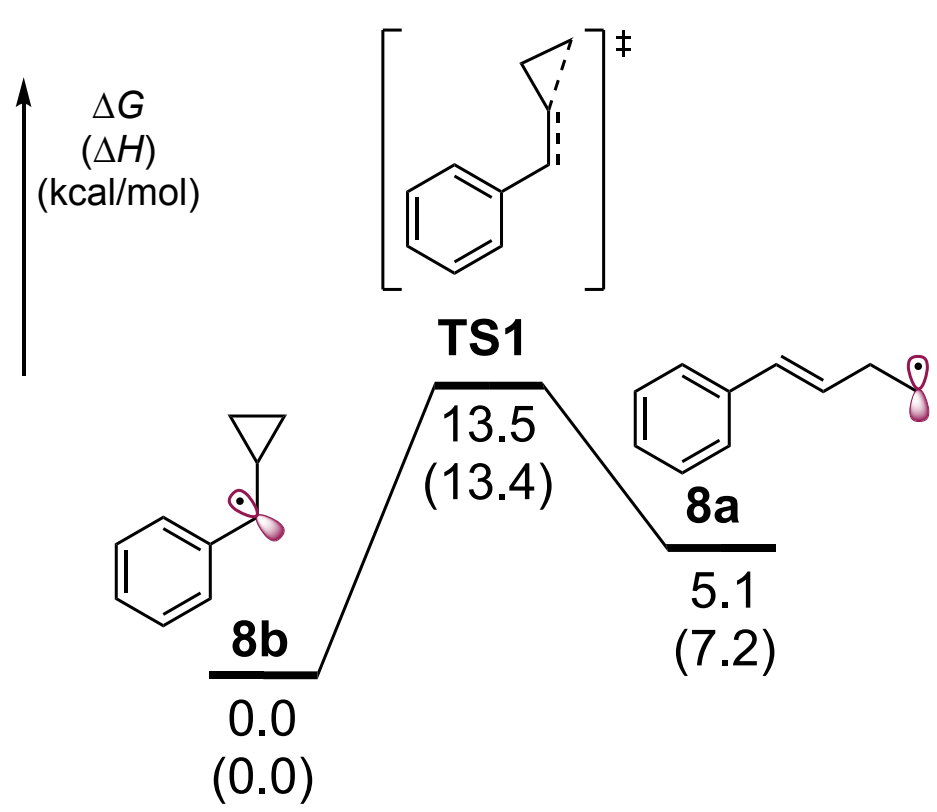

Figure S7. Reaction energy profile of the formation of the ring opened homoallyl radical **8a**.

Classical molecular dynamics (MD) simulations

Classical molecular dynamics (MD) simulations were performed to investigate the interactions between active site residues and the secondary benzyl radical in near-attack conformations (NACs) in the stereoselectivity-determining radical addition step. MD simulations were also performed to study the PLP cofactor-bound enzyme without the radical intermediate. Because the PLP cofactor

in *Tm*TA resides near the interface of two polypeptide chains and residues from both polypeptide chains define the active-site environment, our MD simulations were performed with a dimeric *Tm*TA^{QAM}. The starting dimeric structure of wt *Tm*TA was obtained from the Protein Data Bank (chain A and D, PDB ID: 1LW5)[28]. The Mutagenesis tool in PyMOL[29] was used to generate the *Tm*TA^{QAM} variant from wt *Tm*TA by applying mutations F85M, W86Q, and E88A. The structure of the *Tm*TA^{QAM} variant was compared with that generated by Alphafold3, and the structure generated by PyMOL Mutagenesis was used as the initial geometry in the MD simulations. Classical MD simulations were carried out using the pmemd[30] module of the GPU-accelerated Amber 22 software.[31] The Amber ff19SB[32] force field was used in all classical MD simulations. The second-generation general Amber force field (gaff2)[33] was used for the secondary benzyl radical and the PLP cofactor. Using the Merz-Singh-Kollman scheme,[34,35] RESP charge fitting[36] on electrostatic potential generated at the B3LYP-D3(BJ)/6-31G(d) level of theory was performed to generate partial charges for the radical and the PLP cofactor. Protonation states of enzyme residues were determined using the H++ server.[37] The enzyme was then put into a solvated cuboid box with periodic boundary condition using the TIP3P water model.[38] The minimum distance between the enzyme surface and the edge of the water box was set to 10 Å. Water molecules were treated with the SHAKE algorithm.[39] The system was neutralized by adding Na^+ counterions. Long-range electrostatic was calculated using the particle-mesh-Ewald method.[40] Lennard-Jones and electrostatic interaction cut-offs were set to 12 Å. The classical MD simulations were performed in several steps. We first performed a 30,000-step energy minimization. Next, the system was gradually heated from 0 K to 300 K in 200 ps, which was followed by an equilibration using the isothermal–isobaric ensemble (NPT) in the next 500 ps. Finally, 500 ns production MD simulations were performed using the same conditions as the equilibration with a time step of 2 fs without any restraint. Three independent replicas were simulated for each system. After the MD simulations, clustering analysis was carried out using the CPPTRAJ module[41] to identify the most populated structure from the classical MD simulations of all three replicas. The RMSD values of the Cα, C, and N atoms of the protein backbone were used as the distance metric in the clustering analysis. A representative snapshot of the most visited structure of the holo state of *Tm*TA^{QAM} was then used for docking with the secondary benzyl radical. MD simulations of the near-attack conformations (NACs)[42] of the quinonoid reacting with the (*Re*)- and (*Si*)-faces of the radical were

performed to study the preferred substrate binding pose and the possible interaction modes between activate site residues and the radical substrate. A total of 3 independent replicas of 500 ns MD simulations of NACs were performed following the same four-step computational approach described above (energy minimization, heating, equilibration, and production runs). In the NAC simulations, the initial distance between the $C_{benzylic}$ atom of the radical and the Cα atom of the quinonoid is 2.8 Å, and it was restrained in a range of 2.8–3.0 Å with a harmonic potential of 100 $kcal \cdot mol^{-1} \cdot Å^{-2}$. The NAC simulations were performed with two different initial geometries exposing either the (*Re*)- or (*Si*)-face of the benzylic radical to the quinonoid. Notwithstanding the restraints added, the (*Re*)- and (*Si*)-faces of the benzylic radical could both be exposed to the quinonoid regardless in the same 500 ns trajectory, indicating relatively flexible binding to the benzyl radical prior to the C–C bond formation. To simplify analysis of the interactions with the benzyl radical in pathways leading to the two diastereomeric products, we separated the MD frames based on whether the (*Re*)- or (*Si*)-face of the radical is exposed to the quinonoid. This is achieved by using the sign of dihedral $\theta(C_{\alpha}^{quinonoid}\text{-}C_{methyl}^{radical}\text{-}C_{phenyl}^{radical}\text{-}C_{benzylic}^{radical})$[43] to categorize each MD frame. Positive values indicate that the (*Re*)-face of the benzyl radical is oriented towards the quinonoid, while negative values indicate that the (*Si*)-face for the benzyl radical is exposed to the quinonoid.

Classical MD simulation of *Tm*TA^{QAM}

Three independent replicas of 500 ns MD simulations were performed to analyze the active site of *Tm*TA^{QAM} with the PLP cofactor in the absence of the benzyl radical intermediate. The carboxylate group of the quinonoid establishes persistent polar interactions with hydrogen bond donor groups on the side chains of S6, R171, and R316 (**Figure S8**). The pyridoxal pyridinolate oxygen atom of the quinonoid maintains a strong intramolecular hydrogen bonding interaction with the quinonoid NH (**Figure S9**). In addition, the side chains of N139 and R171 are also placed relatively close to the pyridoxal phenolate oxygen, providing additional stabilization via hydrogen bonding interactions. From the (*Si*)-face of the quinonoid, the pyridine ring of the quinonoid forms a π-π stacking interaction with the imidazole ring of the H83 side chain (**Figure S10**). The pyridine ring is further stabilized via hydrogen bonding interactions with the side chain carboxylic acid group of D168. Multiple hydrogen bond donor residues interact with the PLP 5’-phosphate group

(**Figure S11**). These include the side chain OH groups in T59, the backbone NH groups of G58 and T59, the side chain NH_3^+ group in K199, and the side chain guanidinium group of R231’. In this PLP binding pose, the (*Re*)-face of the prochiral Cα of the quinonoid is blocked by R171, K199, and R231’ residues. This is further validated by distance analysis (**Figure S12**) showing that the median distances between quinonoid Cα and C_{Gdm}^{R171}, N^{K199}, and $C_{Gdm}^{R231'}$ are 5.4, 4.6, and 5.3 Å, respectively.

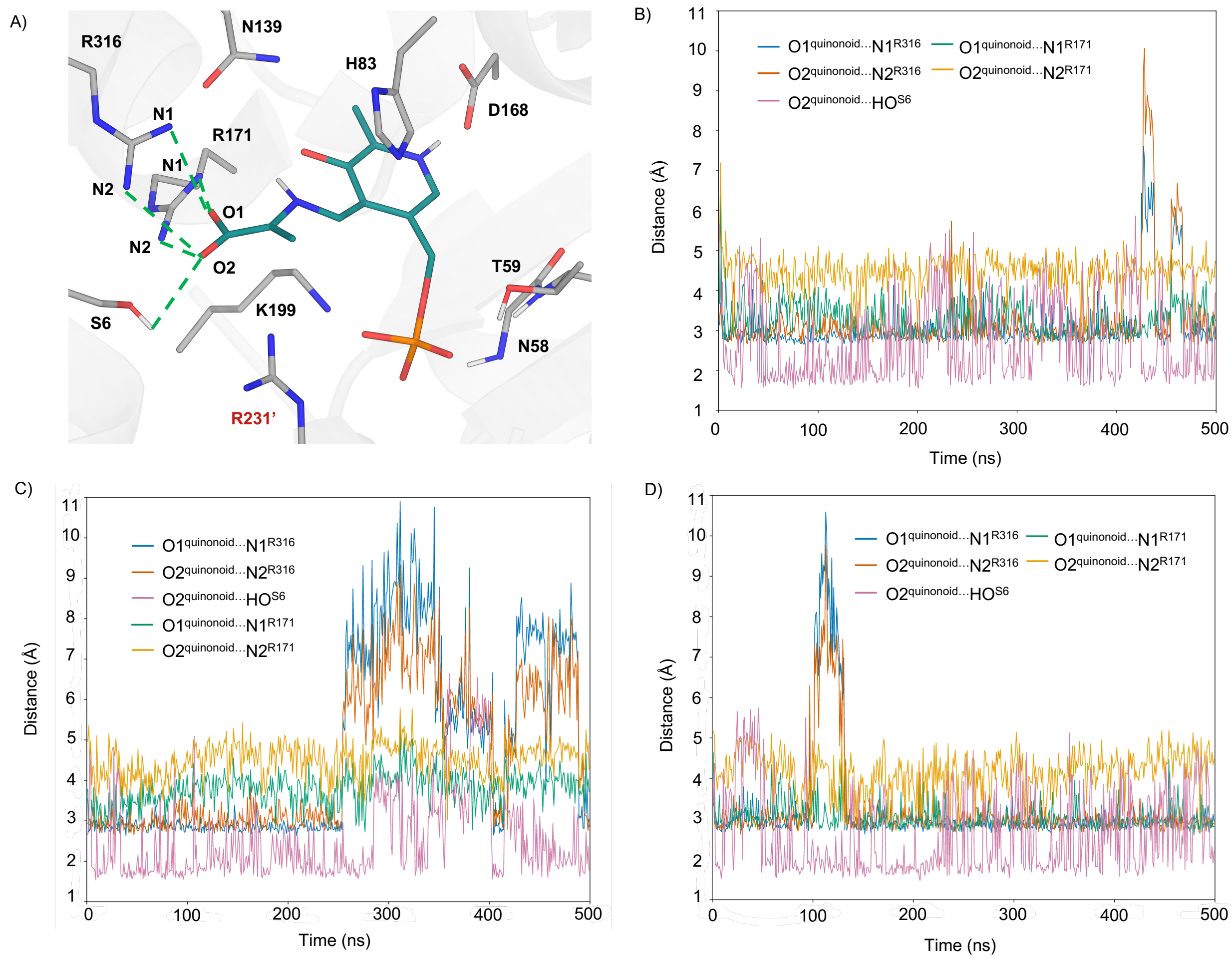

Figure S8. (A) Interactions of the amino acid carboxylate group of the quinonoid intermediate with active site residues of *Tm*TA^{QAM}. (B–D) Distances from the O atoms of the carboxylate group of the quinonoid to the N atoms in the side chain of R171 and R316, and the H atom of the OH group in the side chain of S6. Distances in replicas 1–3 are shown in panels B–D, respectively.

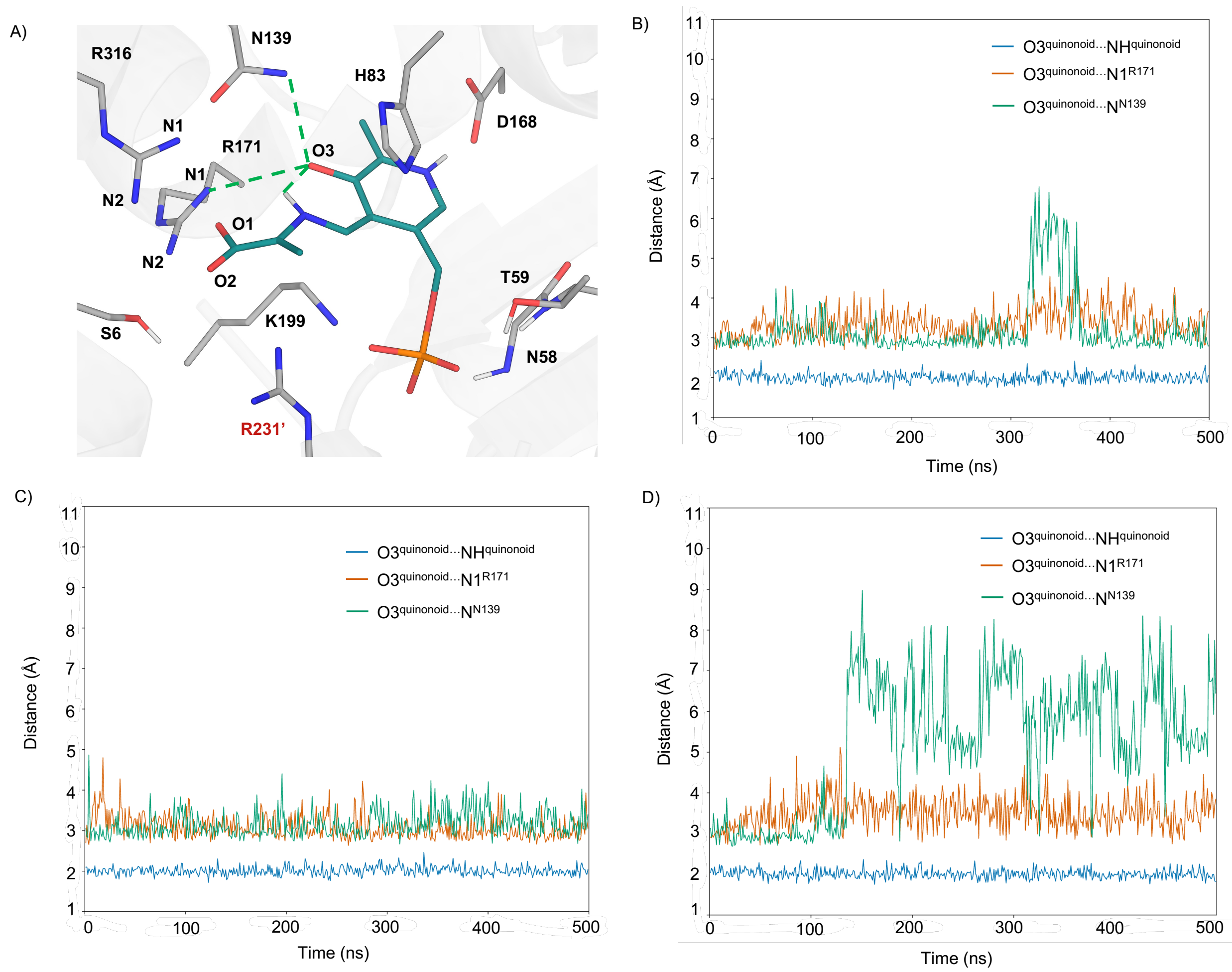

Figure S9. (A) Interactions of the pyridoxal pyridinolate oxygen atom of the quinonoid with active site residues of *Tm*TA^{QAM}. (B–D) Distances from the pyridinolate oxygen of quinonoid to the N atoms in the side chain of N139 and R171 and the quinonoid NH group. Distances in replicas 1–3 are shown in panels B–D, respectively.

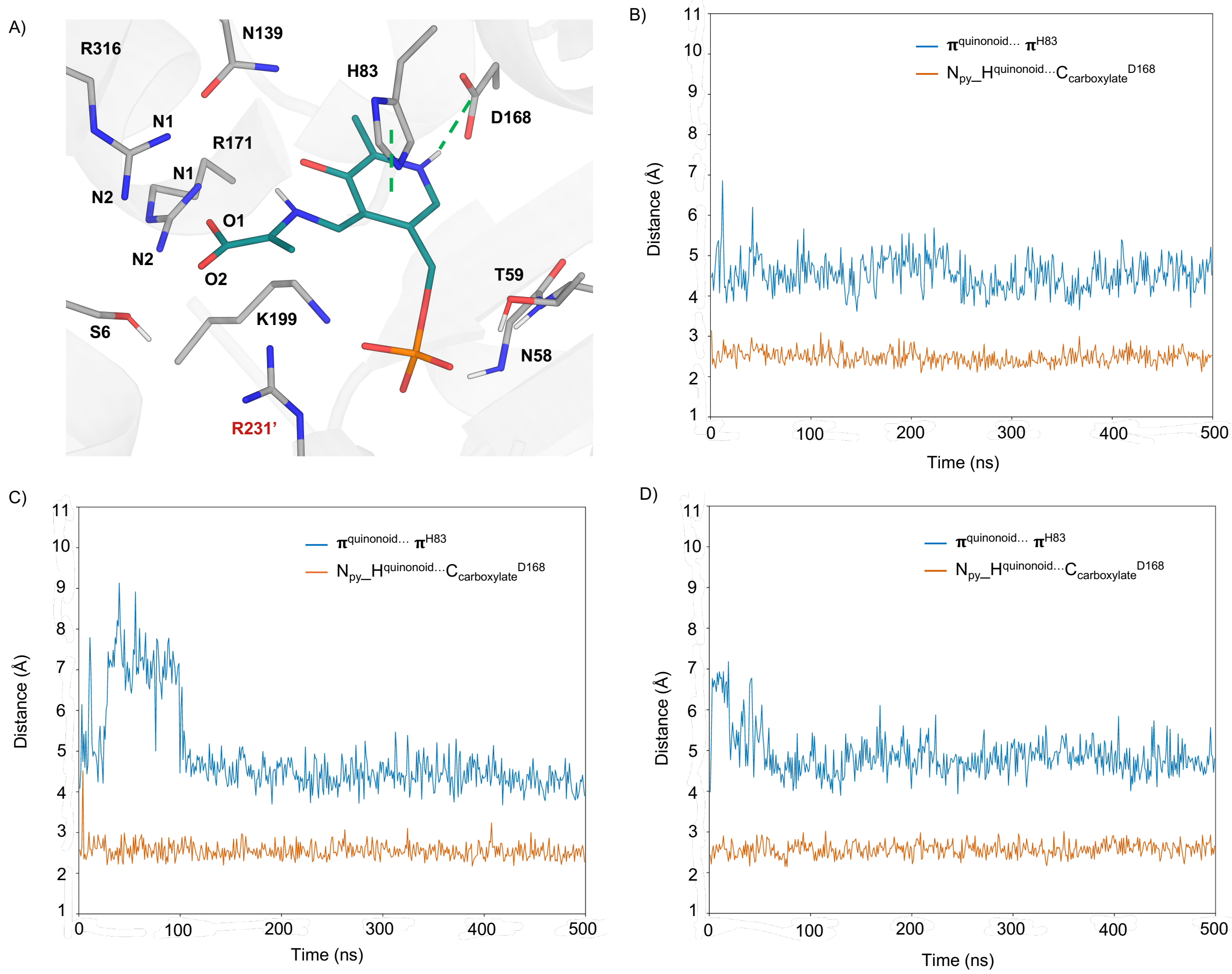

Figure S10. (A) Interactions of the PLP pyridine moiety with active site residues of *Tm*TA^{QAM}. (B–D) Distances from the H atom of the pyridine NH group of quinonoid to the C atom of the carboxylate group in the side chain of D168, and from the centroids of the pyridine moiety of the quinonoid and the imidazole group in the side chain of H83 residue. Distances in replicas 1–3 are shown in panels B–D, respectively.

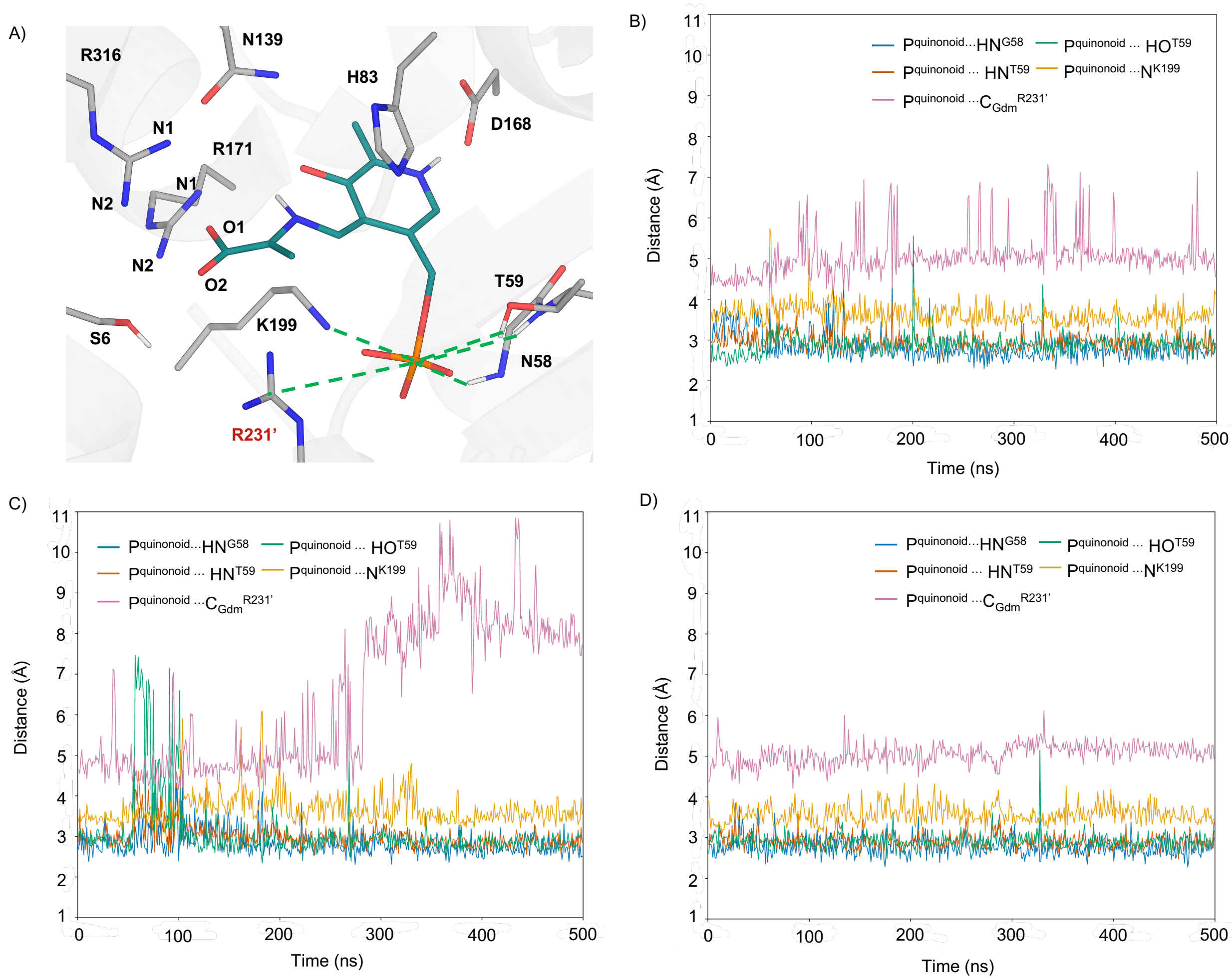

Figure S11. (A) Interactions of the PLP 5'-phosphate of the quinonoid with active site residues of *Tm*TA^{QAM}. (B–D) Distances from the P atom of the 5'-phosphate group to the H atoms of the NH group in the main chains of G58 and T59 residues, the H atom of the OH group in the side chain of T59, the N atom in the side chain of K199, and the guanidinium carbon of R231'. Distances in replicas 1–3 are shown in panels B–D, respectively.

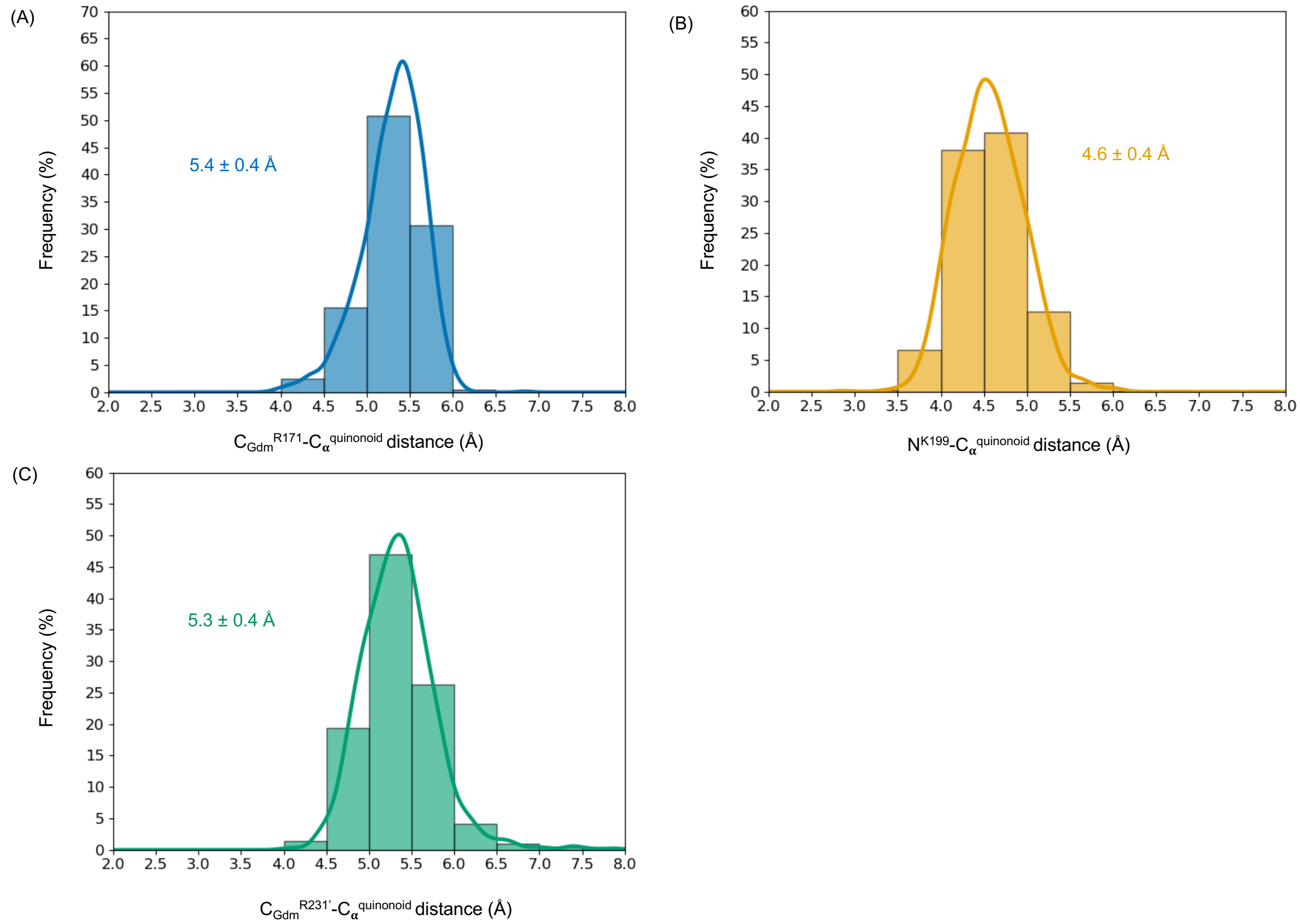

Figure S12. Distances from the quinonoid Cα to (A) the guanidinium carbon of R171 (B) the N atom in the side chain of K199 (C) and the guanidinium carbon of R231'.

MD simulations of near-attack conformations (NACs) of radical addition with *Tm*TA^{QAM}

Three independent replicas of 500 ns MD simulation of the NACs of the α-methyl secondary benzyl radical addition to the quinonoid were performed. The NACs of quinonoid attacking the (*Re*)- and (*Si*)-faces of the α-methyl secondary benzyl radical were both sampled to study the preferred radical binding poses and possible interaction modes between active site residues and the radical in pathways leading to the major and minor diastereomeric products, respectively. In the NAC MD frames of (*Re*)-face radical addition, the Arg-π interaction is weaker because of the eclipsed repulsion between the methyl group of the benzyl radical and the methyl group on the Cα of the quinonoid.

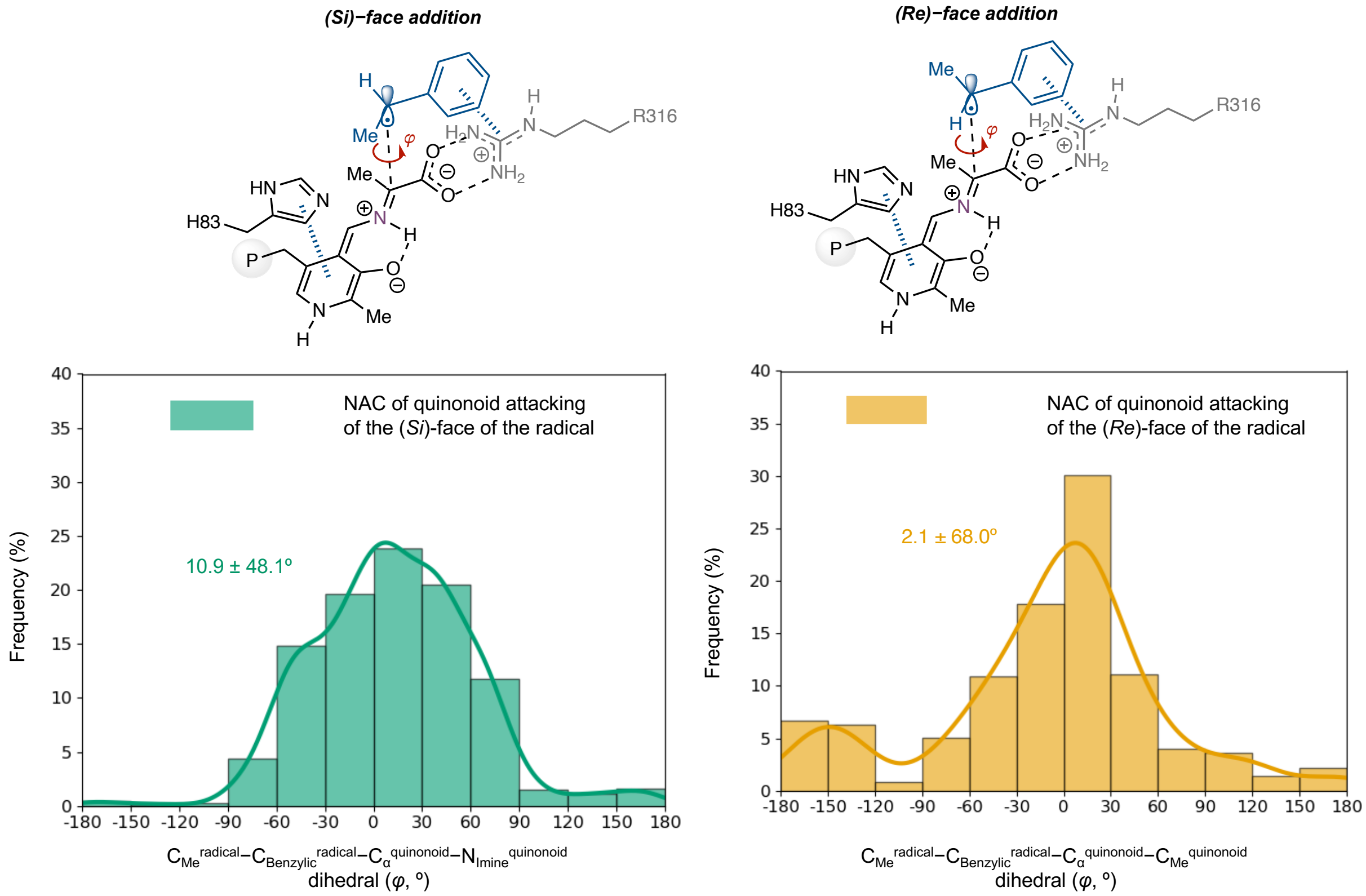

Figure S13. Dihedral analysis to investigate the steric repulsion between the α-methyl secondary benzyl radical and the quinonoid in the MD simulations of the near-attack conformations (NACs) of the quinonoid attacking the (*Re*)- and (*Si*)-face of the prochiral secondary radical. φ, dihedral of $C_{Me}^{radical}$–$C_{Benzylic}^{radical}$–$C_{\alpha}^{quinonoid}$–$N_{Imine}^{quinonoid}$ in (*Si*)-face radical addition and that of $C_{Me}^{radical}$–$C_{Benzylic}^{radical}$–$C_{\alpha}^{quinonoid}$–$C_{Me}^{quinonoid}$ in (*Re*)-face radical addition.

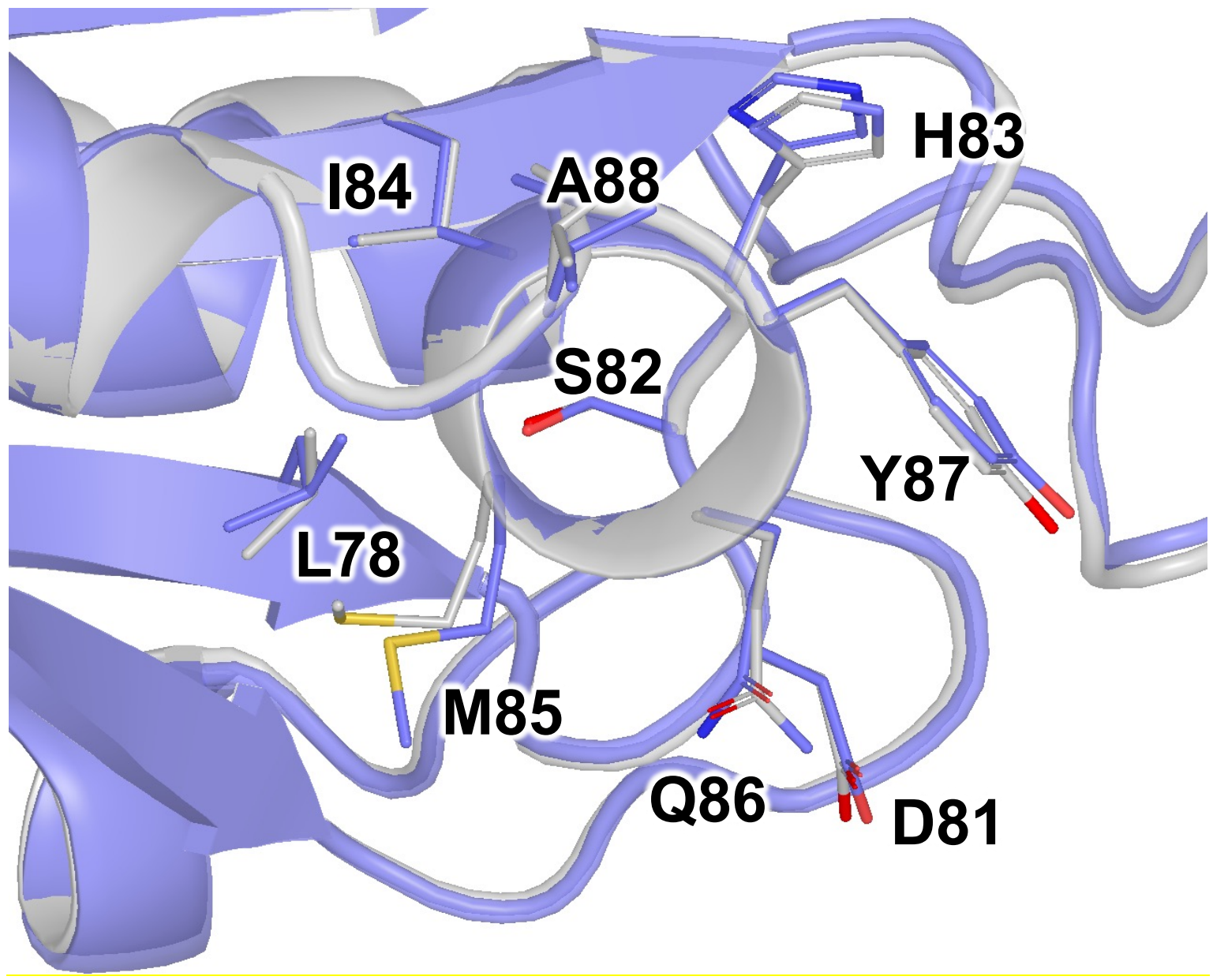

Figure S14. Overlay of the structures of *Tm*TA^{QAM} generated by AlphaFold3 (blue) and the Mutagenesis tool in PyMOL (grey) by applying mutations F85M, W86Q, and E88A from the chain A of the crystal structure of wt *Tm*TA (PDB ID: 1LW5). The RMSD between the two predicted structures is 0.286 Å, indicating high levels of similarity. The structure generated by PyMOL Mutagenesis was used as the initial geometry in the MD simulations.

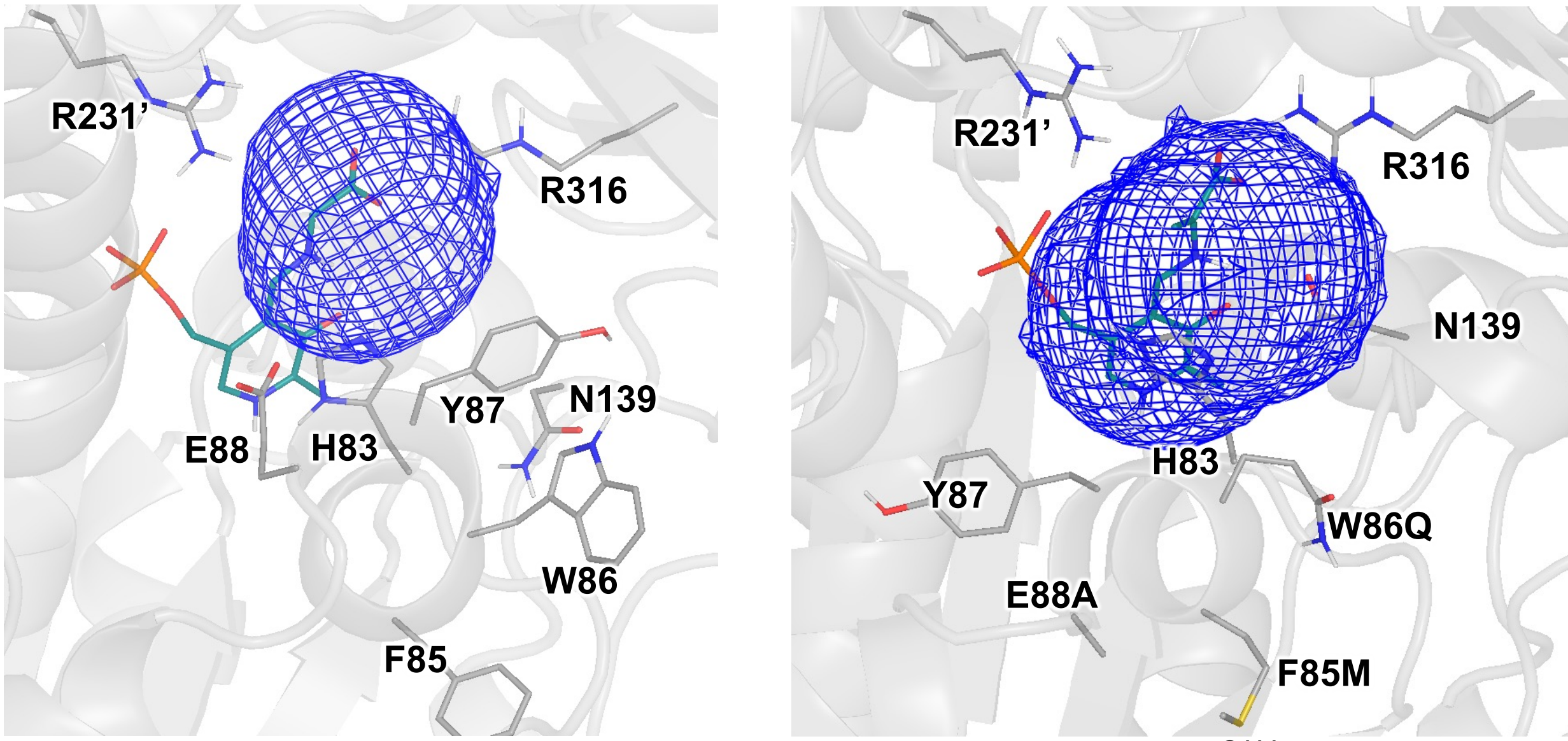

wt *Tm*TA
Average bottleneck radius: 2.81 Å

*Tm*TA^{QAM}
Average bottleneck radius: 3.78 Å

Figure S15. Substrate entrance channel for wt *Tm*TA and *Tm*TA^{QAM} variants obtained from Caver 3.0.[44] The most populated structures from three replicas of 500 ns MD simulations were used in the Caver analyses. These analyses indicate that the *Tm*TA^{QAM} variant has a wider entrance channel.

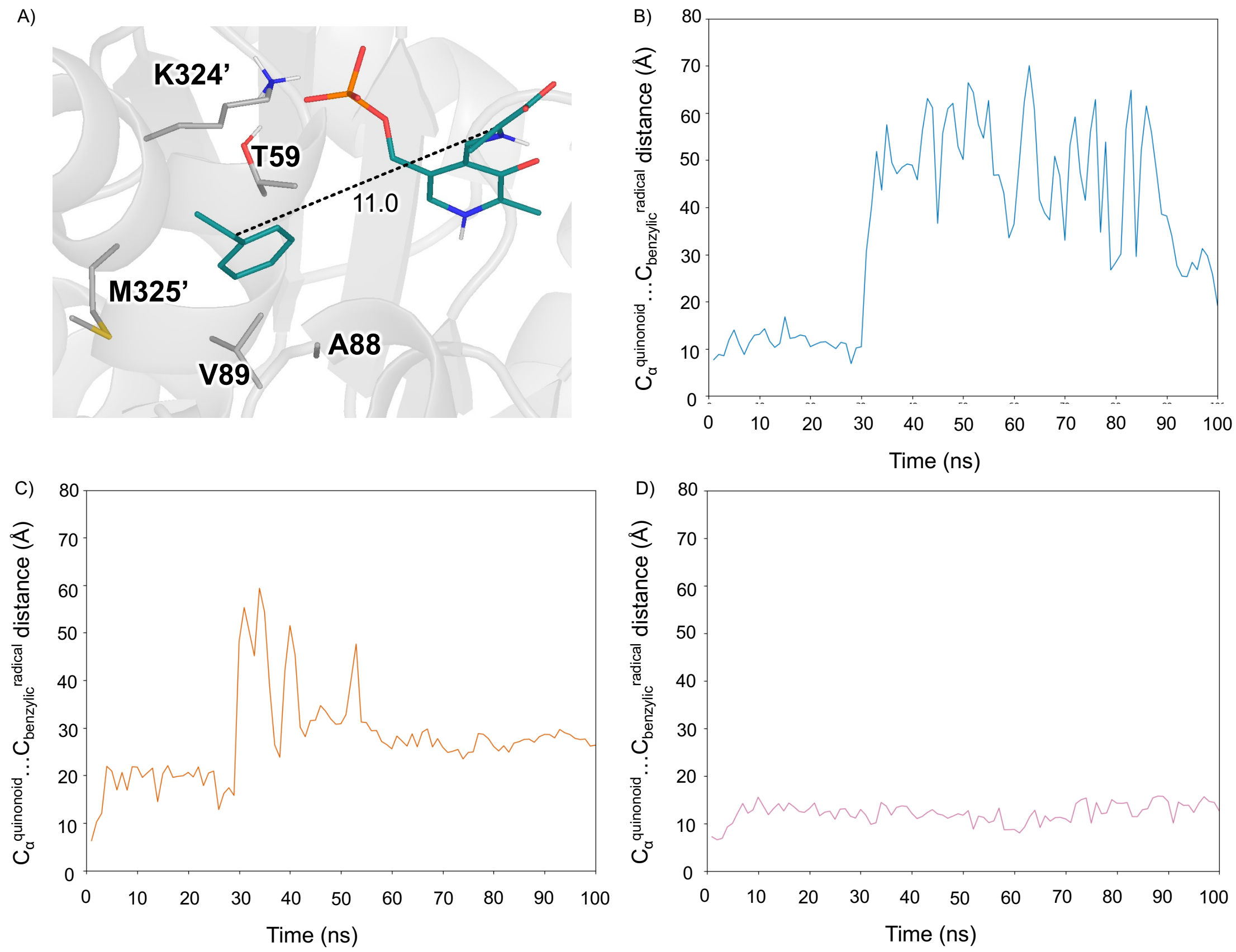

Figure S16. Classical MD simulations of the benzylic radical–quinonoid complex without restraints. In the initial geometry of the MD simulations, the benzylic radical was placed at the (*Si*)-face of the quinonoid, and the distance between the $C_{benzylic}$ atom of the radical and the Cα atom of the quinonoid was 3.0 Å. The radical quickly moved away from the quinonoid during the 500 ps equilibration. (A) A snapshot of replica 1 of the unrestrained MD simulations at 30 ns. The distance between the $C_{benzylic}$ atom of the radical and the Cα atom of the quinonoid is shown in Å. (B–D) Distances between the Cα atom of the quinonoid and the $C_{benzylic}$ atom of the radical during the 100 ns simulations. Distances in replicas 1–3 are shown in panels B–D, respectively. In replicas 1 and 2, the radical completely exited the enzyme after 30 ns.

Cartesian coordinates of all DFT-optimized structures

8b

M06-2X SCF energy: -387.579099 a.u.

M06-2X enthalpy: -387.390380 a.u.

M06-2X gibbs free energy: -387.435411 a.u.

DLPNO-CCSD(T) SCF energy in solution: -386.886234 a.u.

DLPNO-CCSD(T) enthalpy in solution: -386.6975153 a.u.

DLPNO-CCSD(T) gibbs free energy in solution: -386.7425463 a.u.

Cartesian coordinates

ATOM	X	Y	Z
C	-3.17118200	0.29898000	-0.00000700
C	-2.71381200	-1.01769400	-0.00000500
C	-1.36258900	-1.28694700	0.00000200
C	-0.40140500	-0.24639200	0.00000900
C	-0.89063900	1.08156800	0.00000700
C	-2.24662800	1.33977000	-0.00000100
H	-4.23202000	0.50993700	-0.00001300
H	-3.42307000	-1.83566600	-0.00000900
H	-1.01495400	-2.31328700	0.00000400
H	-0.19699400	1.91115200	0.00001000
H	-2.59267400	2.36574000	-0.00000200
C	0.97373900	-0.56965100	0.00001500
H	1.23672500	-1.62289200	0.00001500
C	2.08725200	0.37060100	0.00000100

C	3.35556700	0.00356900	0.74380200
C	3.35555500	0.00356300	-0.74381800
H	1.84108000	1.42235200	-0.00000100
H	3.35890500	-0.94947400	1.25373800
H	3.87449300	0.79871300	1.25938300
H	3.87447200	0.79870300	-1.25941400
H	3.35888300	-0.94948400	-1.25374700

8a

M06-2X SCF energy: -387.5643950 a.u.
M06-2X enthalpy: -387.3773680 a.u.
M06-2X gibbs free energy: -386.4257280 a.u.
DLPNO-CCSD(T) SCF energy in solution: -386.8731429 a.u.
DLPNO-CCSD(T) enthalpy in solution: -386.6861159 a.u.
DLPNO-CCSD(T) gibbs free energy in solution: -386.7344759 a.u.

Cartesian coordinates

ATOM	X	Y	Z
C	3.26942500	0.29824700	-0.20091800
C	2.80770700	-1.00912700	-0.21037200
C	1.45644300	-1.26934800	-0.03548600
C	0.53916100	-0.23383500	0.14579200
C	1.02101500	1.07771500	0.16439200
C	2.36882600	1.34001200	-0.00983200
H	4.32288000	0.50653900	-0.33422900

H	3.49978600	-1.82909200	-0.35276600
H	1.09952800	-2.29261200	-0.04393500
H	0.33769000	1.90041700	0.33044300
H	2.72248200	2.36293300	0.01008500
C	-0.88432600	-0.56446600	0.31057900
H	-1.09659200	-1.61869900	0.47231300
C	-1.90896800	0.28191500	0.26252100
C	-3.34549100	-0.12143100	0.42120700
H	-1.73719500	1.33698100	0.06909700
H	-3.38812300	-1.19642900	0.64643800
C	-4.16181100	0.19608700	-0.78418500
H	-3.77510200	0.38418500	1.29218800
H	-3.73838300	0.07051500	-1.77023300
H	-5.21885300	0.40064600	-0.70159400

TS1

M06-2X SCF energy: -387.1234080 a.u.

M06-2X enthalpy: -386.9371810 a.u.

M06-2X gibbs free energy: -386.9820090 a.u.

DLPNO-CCSD(T) SCF energy in solution: -386.8623614 a.u.

DLPNO-CCSD(T) enthalpy in solution: -386.6761344 a.u.

DLPNO-CCSD(T) gibbs free energy in solution: -386.7209624 a.u.

Imaginary frequency: -280.2364 cm^{-1}

Cartesian coordinates

ATOM X Y Z

C	-3.18264200	0.36036200	0.08360000
C	-2.76851000	-0.97033400	0.14505900
C	-1.41612800	-1.28715700	0.07146200
C	-0.43669100	-0.28517600	-0.06951600
C	-0.87303400	1.05408000	-0.11880800
C	-2.22474900	1.36870500	-0.04657000
H	-4.24239600	0.61175800	0.14227500
H	-3.50572200	-1.76791200	0.25158200
H	-1.09949500	-2.33156500	0.11773800
H	-0.14148400	1.85954000	-0.19856900
H	-2.53657400	2.41390800	-0.08444000
C	0.96571000	-0.66485200	-0.15710300
H	1.19255200	-1.71594400	0.05129100
C	2.01081800	0.16885600	-0.46874800
C	3.47068500	0.41416200	0.88969300
C	3.44627700	-0.23998300	-0.43691700
H	1.80960800	1.19479900	-0.78496900
H	3.21538000	-0.15156700	1.78373100
H	3.69638300	1.47484000	0.99011100
H	4.07324300	0.22710900	-1.20443400
H	3.58809900	-1.32694800	-0.41723500

XIV. ReferencesUncategorized References

1. Wang, T.-C.; Mai, B. K.; Zhang, Z.; Bo, Z.; Li, J.; Liu, P.; Yang, Y. Stereoselective amino acid synthesis by photobiocatalytic oxidative coupling. *Nature* **2024,** *629*, 98-104.

2. Wang, T.-C.; Zhang, Z.; Rao, G.; Li, J.; Shirah, J.; Britt, R. D.; Zhu, Q.; Yang, Y. Threonine Aldolase-Catalyzed Enantioselective α-Alkylation of Amino Acids through Unconventional Photoinduced Radical Initiation. *J. Am. Chem. Soc.* **2024,** *146*, 22476-22484.

3. Kille, S.; Acevedo-Rocha, C. G.; Parra, L. P.; Zhang, Z.-G.; Opperman, D. J.; Reetz, M. T.; Acevedo, J. P. Reducing Codon Redundancy and Screening Effort of Combinatorial Protein Libraries Created by Saturation Mutagenesis. *ACS Synth. Biol.* **2013,** *2*, 83-92.

4. Gibson, D. G.; Young, L.; Chuang, R.-Y.; Venter, J. C.; Hutchison, C. A.; Smith, H. O. Enzymatic assembly of DNA molecules up to several hundred kilobases. *Nat. Methods* **2009,** *6*, 343-345.

5. Kazmierski, W. M.; Urbanczyk-Lipkowska, Z.; Hruby, V. J. New Amino Acids for the Topographical Control of Peptide Conformation: Synthesis of All the Isomers of .alpha.,.beta.-Dimethylphenylalanine and .alpha.,.beta.-Dimethyl-1,2,3,4-tetrahydroisoquinoline-3-carboxylic Acid of High Optical Purity. *J. Org. Chem.* **1994,** *59*, 1789-1795.

6. Davis, F. A.; Liang, C.-H.; Liu, H. Asymmetric Synthesis of β-Substituted α-Amino Acids Using 2H-Azirine-2-carboxylate Esters. Synthesis of 3,3-Disubstituted Aziridine-2-carboxylate Esters. *J. Org. Chem.* **1997,** *62*, 3796-3797.

7. Soloshonok, V. A.; Tang, X.; Hruby, V. J.; Meervelt, L. V. Asymmetric Synthesis of α,β-Dialkyl-α-phenylalanines via Direct Alkylation of a Chiral Alanine Derivative with Racemic α-Alkylbenzyl Bromides. A Case of High Enantiomer Differentiation at Room Temperature. *Org. Lett.* **2001,** *3*, 341-343.

8. Williams, R. M.; Im, M. N. Asymmetric synthesis of monosubstituted and .alpha.,.alpha.-disubstituted .alpha.-amino acids via diastereoselective glycine enolate alkylations. *J. Am. Chem. Soc.* **1991,** *113*, 9276-9286.

9. Stoll, S.; Schweiger, A. EasySpin, a comprehensive software package for spectral simulation and analysis in EPR. *Journal of Magnetic Resonance* **2006,** *178*, 42-55.

10. Youn, J. H.; Go, S. Y.; Chung, H.; Lee, H.; Chung, T. D.; Cheong, P. H.-Y.; Lee, H. G. Dual Function of N-Iodosuccinimide for C(sp3)–B Bond Activation. *Org. Lett.* **2024,** *26*, 198-203.

11. Go, S. Y.; Chung, H.; Shin, S. J.; An, S.; Youn, J. H.; Im, T. Y.; Kim, J. Y.; Chung, T. D.; Lee, H. G. A Unified Synthetic Strategy to Introduce Heteroatoms via Electrochemical Functionalization of Alkyl Organoboron Reagents. *J. Am. Chem. Soc.* **2022,** *144*, 9149-9160.

12. Roh, B.; Farah, A. O.; Kim, B.; Feoktistova, T.; Moeller, F.; Kim, K. D.; Cheong, P. H.-Y.; Lee, H. G. Stereospecific Acylative Suzuki–Miyaura Cross-Coupling: General Access to Optically Active α-Aryl Carbonyl Compounds. *J. Am. Chem. Soc.* **2023,** *145*, 7075-7083.

13. Li, H.; Wang, L.; Zhang, Y.; Wang, J. Transition-Metal-Free Synthesis of Pinacol Alkylboronates from Tosylhydrazones. *Angew. Chem. Int. Ed.* **2012,** *51*, 2943-2946.

14. Hu, J.; Sun, H.; Cai, W.; Pu, X.; Zhang, Y.; Shi, Z. Nickel-Catalyzed Borylation of Aryl- and Benzyltrimethylammonium Salts via C–N Bond Cleavage. *J. Org. Chem.* **2016,** *81*, 14-24.

15. Lima, F.; Kabeshov, M. A.; Tran, D. N.; Battilocchio, C.; Sedelmeier, J.; Sedelmeier, G.; Schenkel, B.; Ley, S. V. Visible Light Activation of Boronic Esters Enables Efficient Photoredox $C(sp^2)$–$C(sp^3)$ Cross-Couplings in Flow. *Angew. Chem. Int. Ed.* **2016,** *55*, 14085-14089.

16. Cheng, L.; Li, D.; Mai, B. K.; Bo, Z.; Cheng, L.; Liu, P.; Yang, Y. Stereoselective amino acid synthesis by synergistic photoredox-pyridoxal radical biocatalysis. *Science* **2023,** *381*, 444-451.

17. Ueki, H.; Ellis, T. K.; Martin, C. H.; Boettiger, T. U.; Bolene, S. B.; Soloshonok, V. A. Improved Synthesis of Proline-Derived Ni(II) Complexes of Glycine: Versatile Chiral Equivalents of Nucleophilic Glycine for General Asymmetric Synthesis of α-Amino Acids. *J. Org. Chem.* **2003,** *68*, 7104-7107.

18. Martínez-Mingo, M.; García-Viada, A.; Prendes, D. S.; Alonso, I.; Rodríguez, N.; Arrayás, R. G.; Carretero, J. C. Palladium-Catalyzed PIDA-Mediated δ-C(sp3)−H Acetoxylation of Amino Acid Derivatives: Overriding Competitive Intramolecular Amination. *Angew. Chem. Int. Ed.* **2022,** *61*, e202209865.

19. Trost, B. M.; Dogra, K. Synthesis of Novel Quaternary Amino Acids Using Molybdenum-Catalyzed Asymmetric Allylic Alkylation. *J. Am. Chem. Soc.* **2002,** *124*, 7256-7257.

20. Chen, W.; Hartwig, J. F. Control of Diastereoselectivity for Iridium-Catalyzed Allylation of a Prochiral Nucleophile with a Phosphate Counterion. *J. Am. Chem. Soc.* **2013,** *135*, 2068-2071.

21. Frisch, M. J.; Trucks, G. W.; Schlegel, H. B.; Scuseria, G. E.; Robb, M. A.; Cheeseman, J. R.; Scalmani, G.; Barone, V.; Petersson, G. A.; Nakatsuji, H.; Li, X.; Caricato, M.; Marenich, A. V.; Bloino, J.; Janesko, B. G.; Gomperts, R.; Mennucci, B.; Hratchian, H. P.; Ortiz, J. V.; Izmaylov,

A. F.; Sonnenberg, J. L.; Williams-Young, D.; Ding, F.; Lipparini, F.; Egidi, F.; Goings, J.; Peng, B.; Petrone, A.; Henderson, T.; Ranasinghe, D.; Zakrzewski, V. G.; Gao, J.; Rega, N.; Zheng, G.; Liang, W.; Hada, M.; Ehara, M.; Toyota, K.; Fukuda, R.; Hasegawa, J.; Ishida, M.; Nakajima, T.; Honda, Y.; Kitao, O.; Nakai, H.; Vreven, T.; Throssell, K.; Montgomery, J. A. Jr.; Peralta, J. E.; Ogliaro, F.; Bearpark, M. J.; Heyd, J. J.; Brothers, E. N.; Kudin, K. N.; Staroverov, V. N.; Keith, T. A.; Kobayashi, R.; Normand, J.; Raghavachari, K.; Rendell, A. P.; Burant, J. C.; Iyengar, S. S.; Tomasi, J.; Cossi, M.; Millam, J. M.; Klene, M.; Adamo, C.; Cammi, R.; Ochterski, J. W.; Martin, R. L.; Morokuma, K.; Farkas, O.; Foresman, J. B.; Fox, D. J. *Gaussian 16, Revision C.01*; Gaussian, Inc.: Wallingford CT, 2019.

22. Neese, F. Software update: the ORCA program system, version 5.0 *WIREs Comput. Mol. Sci.*, **2022**, *12*, e1606.

23. Zhao, Y.; Truhlar, D. G. The M06 suite of density functionals for main group thermochemistry, thermochemical kinetics, noncovalent interactions, excited states, and transition elements: Two new functionals and systematic testing of four M06 class functionals and 12 other functionals. *Theor. Chem. Acc.* **2008**, *120*, 215–241.

24. Purvis, G. D., III; Bartlett, R. J. A Full Coupled-Cluster Singles and Doubles Model: The Inclusion of Disconnected Triples. *J. Chem. Phys.* **1982**, *76*, 1910−1918.

25. Riplinger, C.; Neese, F. An efficient and near linear scaling pair natural orbital based local coupled cluster method. *J. Chem. Phys***.** **2013**, *138*, 034106.

26. Riplinger, C.; Sandhoefer, B.; Hansen, A.; Neese, F. Natural triple excitations in local coupled cluster calculations with pair natural orbitals. *J. Chem. Phys.* **2013**, *139*, 134101.

27. Marenich, A. V.; Cramer, C. J.; Truhlar, D. G. Universal solvation model based on solute electron density and on a continuum model of the solvent defined by the bulk dielectric constant and atomic surface tensions. *J. Phys. Chem. B.* **2009**, *113*, 6378-6396.

28. Kielkopf, C. L.; Burley, S. K. X-ray Structures of Threonine Aldolase Complexes: Structural Basis of Substrate Recognition. *Biochemistry* **2002**, *41*, 11711–11720.

29. The PyMOL Molecular Graphics System, Version 2.5.0 Schrödinger, LLC.

30. Salomon-Ferrer, R.; Götz, A. W.; Poole, D.; Grand, S. L.; Walker, R. C. Routine microsecond molecular dynamics simulations with AMBER on GPUs. 2. Explicit solvent particle mesh Ewald. *J. Chem. Theory Comput.* **2013**, *9*, 3878-3888.

31. Case, D. A.; Aktulga, H. M.; Belfon, K. A. A.; Ben-Shalom, I. Y.; Berryman, J. T.; Brozell, S. R.; Cerutti, D. S.; Cheatham, T. E., III; Cisneros, G. A.; Cruzeiro, V. W. D.; Darden, T. A.; Duke, R. E.; Giambasu, G.; Gilson, M. K.; Gohlke, H.; Goetz, A. W.; Harris, R. C.; Izadi, S.; Izmailov, S. A.; Kasavajhala, K.; Kaymak, M. C.; King, E.; Kovalenko, A.; Kurtzman, T.; LeGrand, S.; Li, P.; Liu, J.; Luchko, T.; Luo, R.; Machado, M.; Man, V.; Manathunga, M.; Merz, K. M., Jr.; Miao, Y.; Mikhailovskii, O.; Monard, G.; Nguyen, H.; O'Hearn, K. A.; Onufriev, A. V.; Pan, F.; Pantano, S.; Qi, R.; Rahnamoun, A.; Roe, D. R.; Roitberg, A.; Sagui, C.; Schott-Verdugo, S.; Shajan, A.; Shen, J.; Simmerling, C. L.; Skrynnikov, N. R.; Smith, J.; Swails, J.; Walker, R. C.; Wang, J.; Wei, H.; Wolf, R. M.; Wu, X.; Xiong, Y.; Xue, Y.; York, D. M.; Zhao, S.; Kollman, P. A. *Amber 2022*, University of California, San Francisco, 2022.

32. Tian, C.; Kasavajhala, K.; Belfon, K. A. A.; Raguette, L.; Huang, H.; Migues, A. N.; Bickel, J.; Wang, Z.; Pincay, J.; Wu, Q.; Simmerling, C. ff19SB: Amino-Acid-Specific Protein Backbone Parameters Trained against Quantum Mechanics Energy Surfaces in Solution. *J. Chem. Theory Comput.* **2020**, *16*, 528-552.

33. Wang, J.; Wolf, R. M.; Caldwell, J. W.; Kollman, P. A.; Case, D. A. Development and testing of a general amber force field. *J. Comput. Chem.* **2004**, *25*, 1157-1174.

34. Singh, U. C.; Kollman, P. A. An approach to computing electrostatic charges for molecules. *J. Comput. Chem.* **1984**, *5*, 129-145.

35. Besler, B. H.; Merz Jr, K. M.; Kollman, P. A. Atomic charges derived from semiempirical methods. *J. Comput. Chem.* **1990**, *11*, 431-439.

36. Bayly, C. I.; Cieplak, P.; Cornell, W.; Kollman, P. A. A well-behaved electrostatic potential based method using charge restraints for deriving atomic charges: the RESP model. *J. Phys. Chem.* **1993**, *97*, 10269-10280.

37. Anandakrishnan, R.; Aguilar, B.; Onufriev, A. V. H++ 3.0: Automating pK prediction and the preparation of biomolecular structures for atomistic molecular modeling and simulations. *Nucleic Acids Res*. **2012**, *40*, W537-W541.

38. Jorgensen, W. L.; Chandrasekhar, J.; Madura, J. D.; Impey, R. W.; Klein, M. L. Comparison of simple potential functions for simulating liquid water. *J. Chem. Phys.* **1983**, *79*, 926-935.

39. Ryckaert, J.; Ciccotti, G.; Berendsen, H. J. C. Numerical integration of the cartesian equations of motion of a system with constraints: Molecular dynamics of n-alkanes. *J. Comput. Phys.* **1977**, *23*, 327-341.

40. Darden, T.; York, D.; Pedersen, L. Particle mesh Ewald: An N·log(N) method for Ewald sums in large systems. *J. Chem. Phys.* **1993**, *98*, 10089-10092.

41. Roe, D. R.; Cheatham, T. E. PTRAJ and CPPTRAJ: Software for processing and analysis of molecular dynamics trajectory data. *J. Chem. Theory Comput.* **2013**, *9*, 3084-3095.

42. Soler, J.; Gergel, S.; Klaus, C.; Hammer, S. C.; Garcia-Borràs, M. Enzymatic control over reactive intermediates enables direct oxidation of alkenes to carbonyls by a P450 iron-oxo species. *J. Am. Chem. Soc.* **2022**, *144*, 15954-15968.

43. Rui, J.; Mu, X.; Soler, J.; Paris, J. C.; Guo, Y.; Garcia-Borràs, M.; Huang, X. Merging photoredox with metalloenzymatic catalysis for enantioselective decarboxylative $C(sp^3)–N_3$ and $C(sp^3)$–SCN bond formation. *Nat. Catal.* **2024**, *7*, 1394-1403.

44. Chovancova, E.; Pavelka, A.; Benes, P.; Strnad, O.; Brezovsky, J.; Kozlikova, B.; Gora, A.; Sustr, V.; Klvana, M.; Medek, P.; Biedermannova, L.; Sochor, J.; Damborsky, J. CAVER 3.0: A Tool for the Analysis of Transport Pathways in Dynamic Protein Structures. *PLoS Comput. Biol.* **2012**, *8*, e1002708.

XV. ^{1}H, ^{19}F and ^{13}C NMR spectra of compounds

XIV. ^{1}H, ^{19}F and ^{13}C NMR spectra of compounds

^{1}H NMR (400 MHz, DMSO-d_6) of Potassium trifluoro (1-phenylbutyl)- borate ((***rac***)-**1k**)

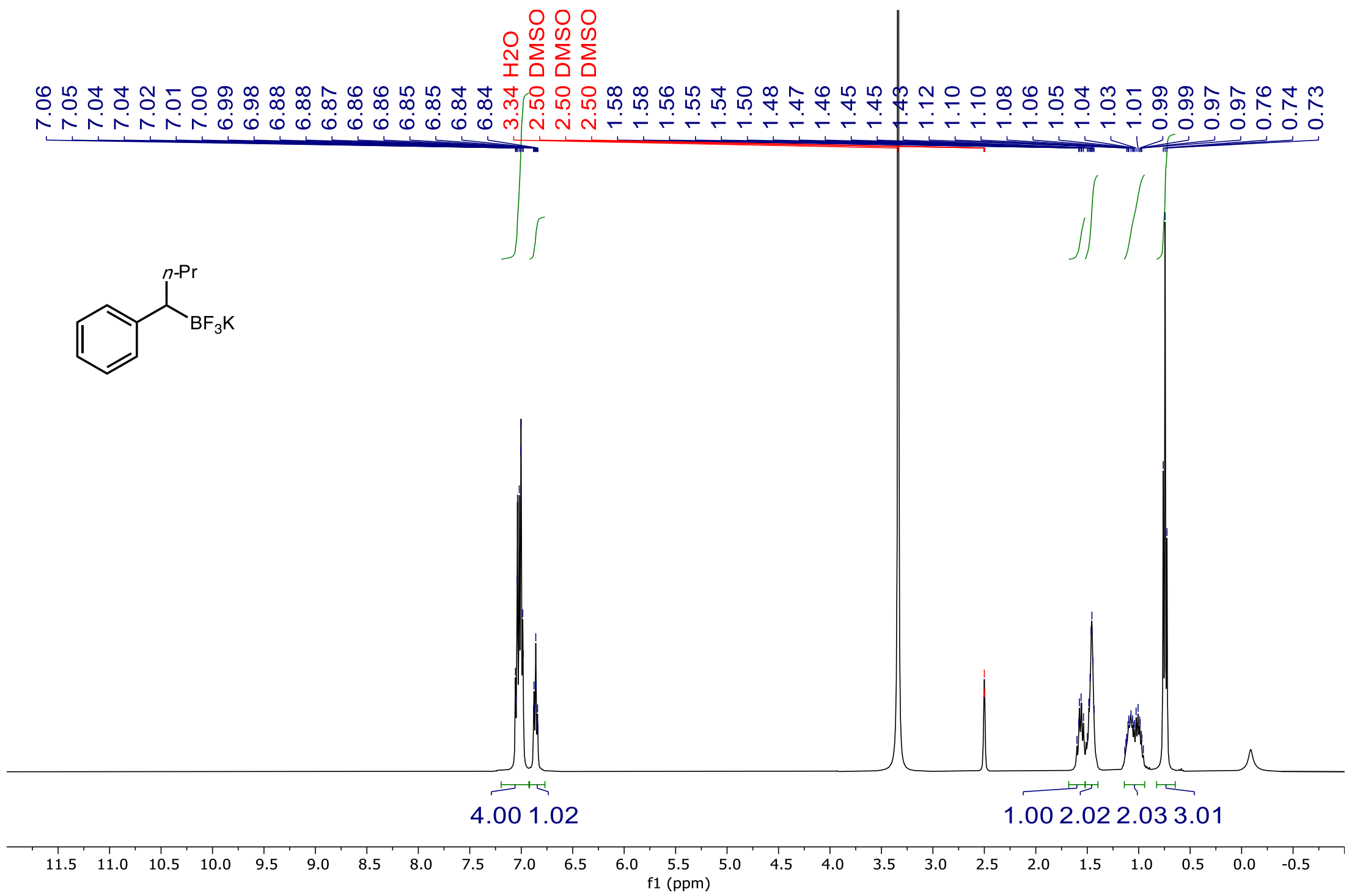

^{19}F NMR (376 MHz, DMSO-d_6) of Potassium trifluoro (1-phenylbutyl)- borate ((***rac***)-**1k**)

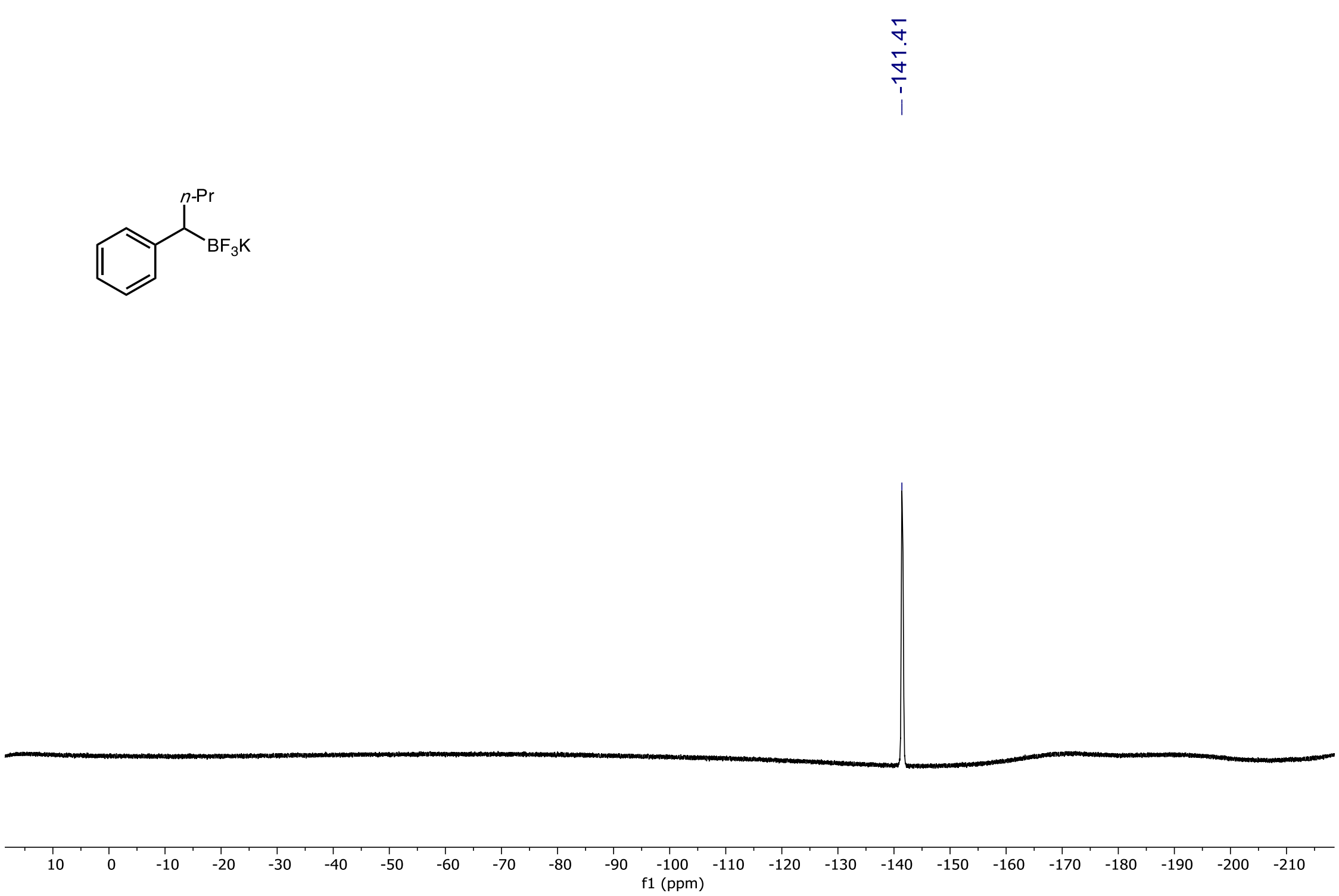

^{11}B NMR (128 MHz, DMSO-d_6) of Potassium trifluoro (1-phenylbutyl)- borate ((***rac***)**-1k**)

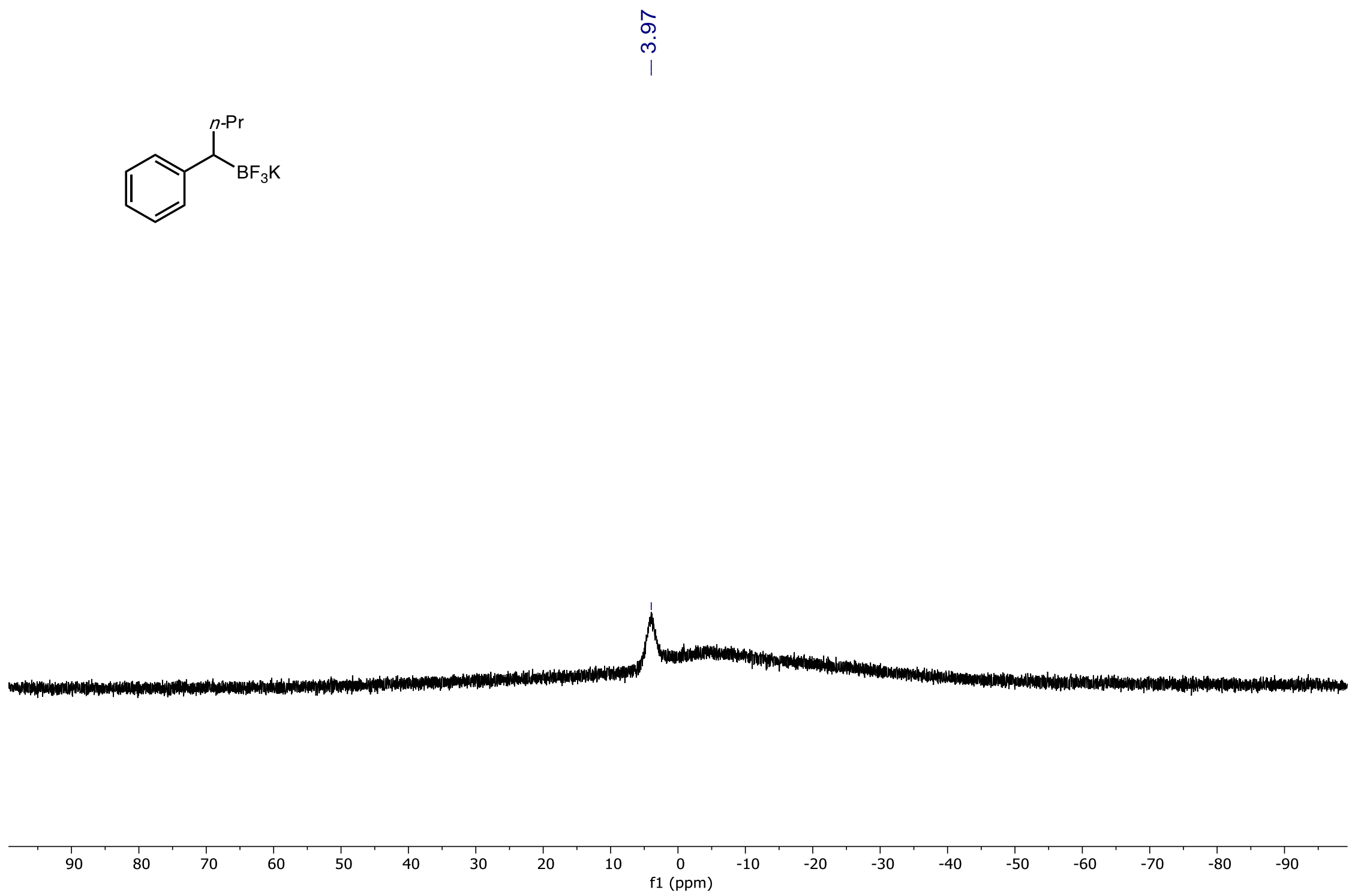

^{13}C NMR (101 MHz, DMSO-d_6) of Potassium trifluoro (1-phenylbutyl)- borate ((***rac***)**-1k**)

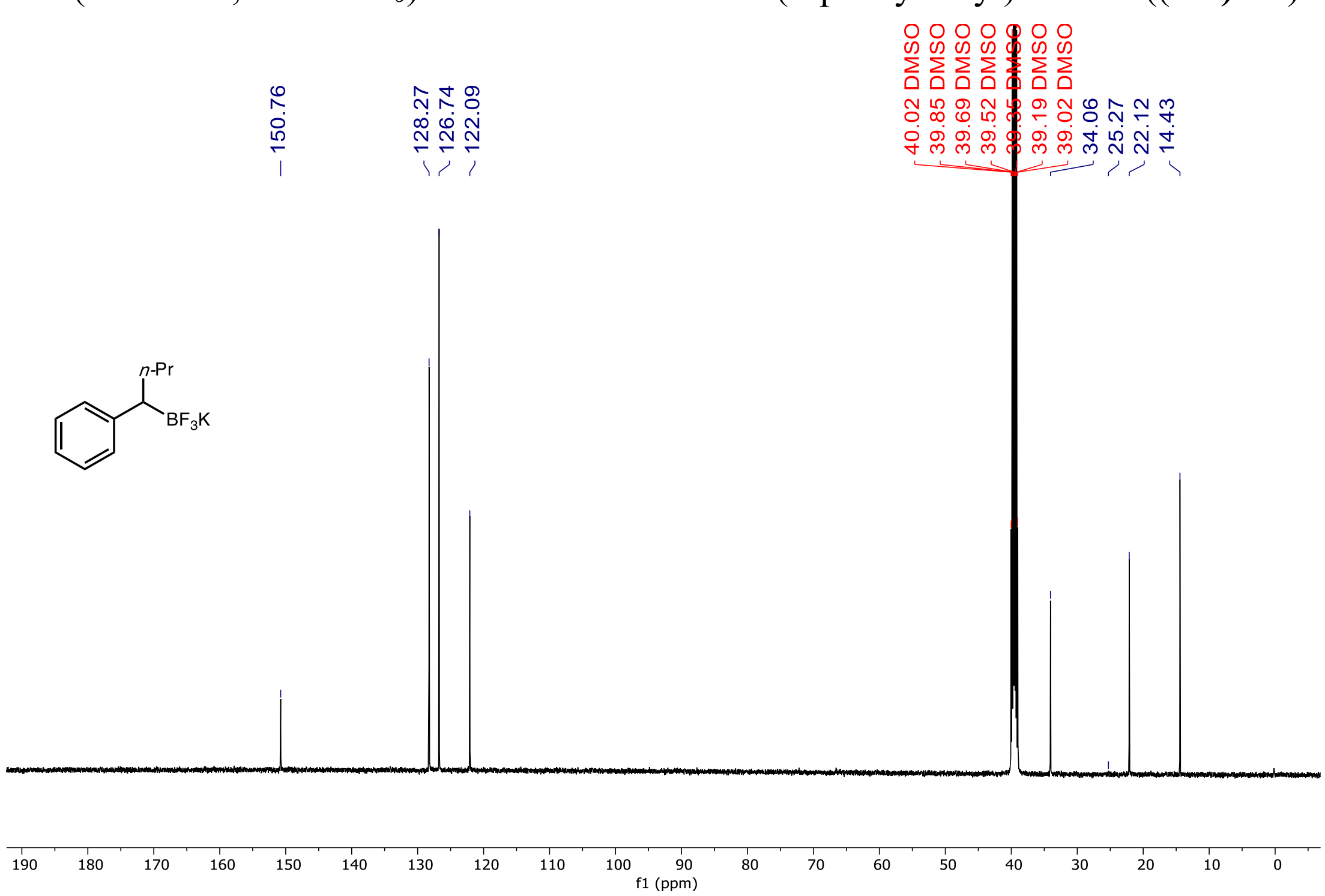

^{1}H NMR (400 MHz, DMSO-d_6) of Chroman-4-yltrifluoroboronate, potassium salt ((***rac***)-**1n**)

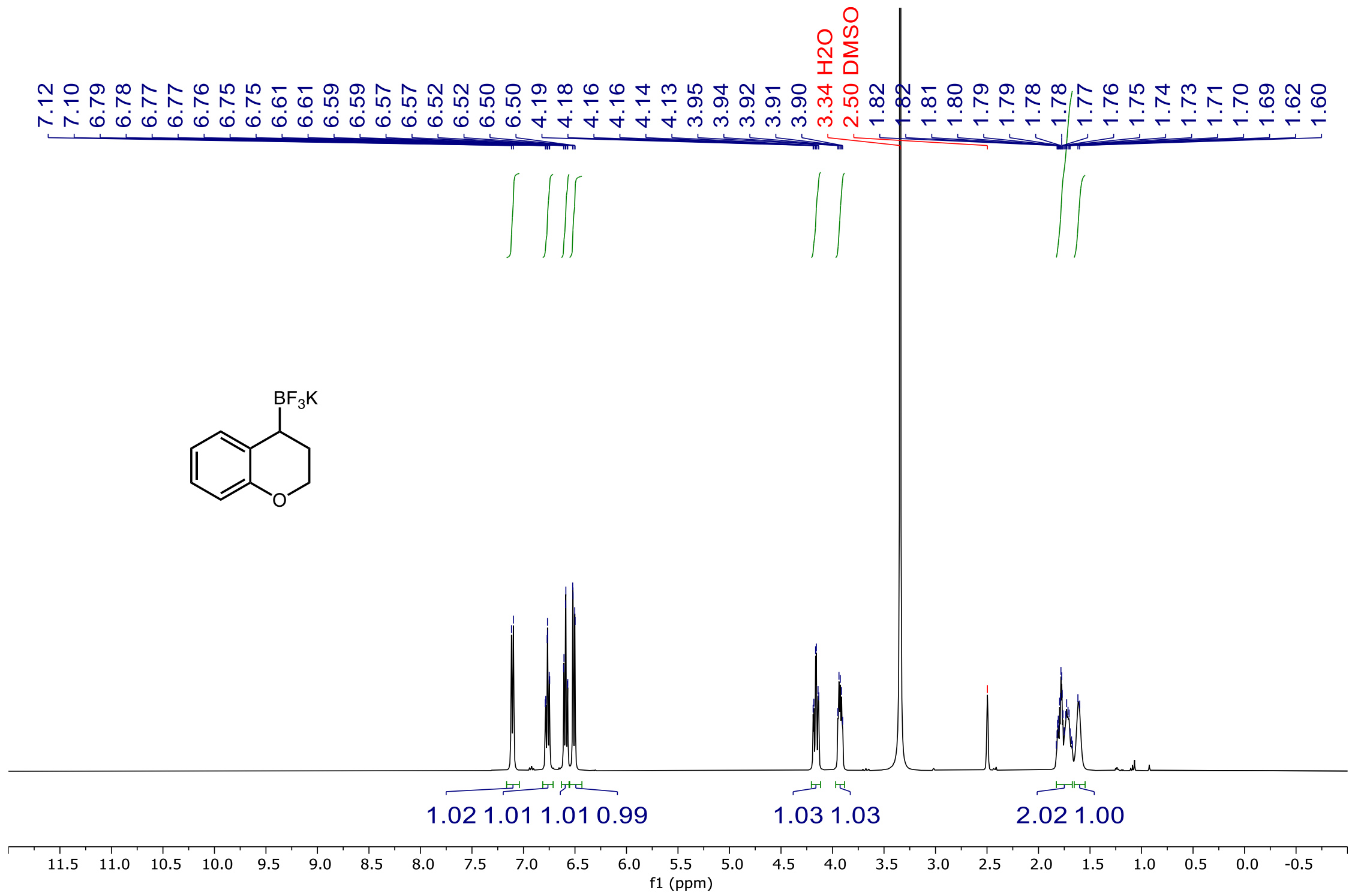

^{19}F NMR (376 MHz, DMSO-d_6) of Chroman-4-yltrifluoroboronate, potassium salt ((***rac***)-**1n**)

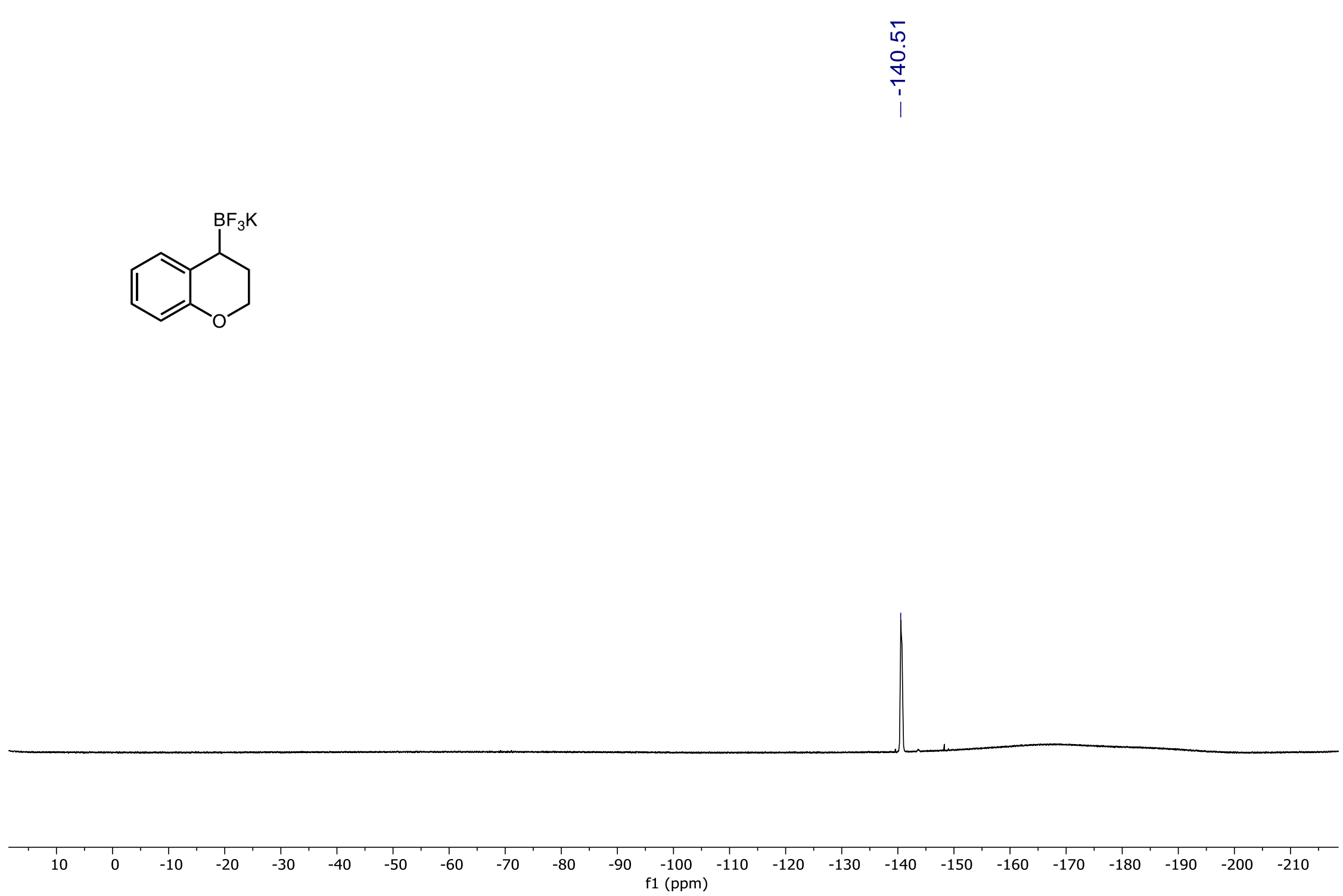

^{11}B NMR (128 MHz, DMSO-d_6) of Chroman-4-yltrifluoroboronate, potassium salt ((***rac***)-**1n**)

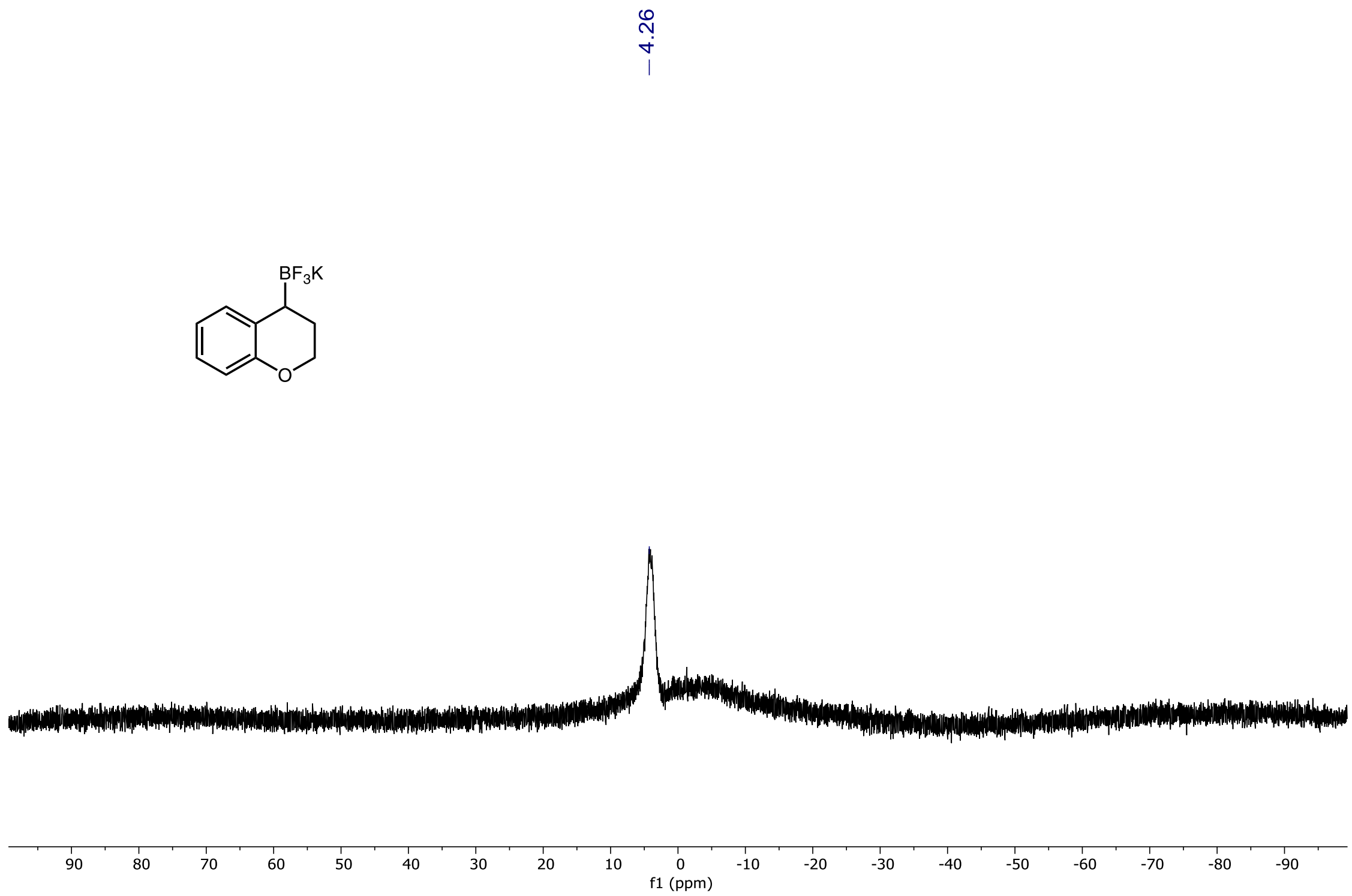

^{13}C NMR (101 MHz, DMSO-d_6) of Chroman-4-yltrifluoroboronate, potassium salt ((***rac***)-**1n**)

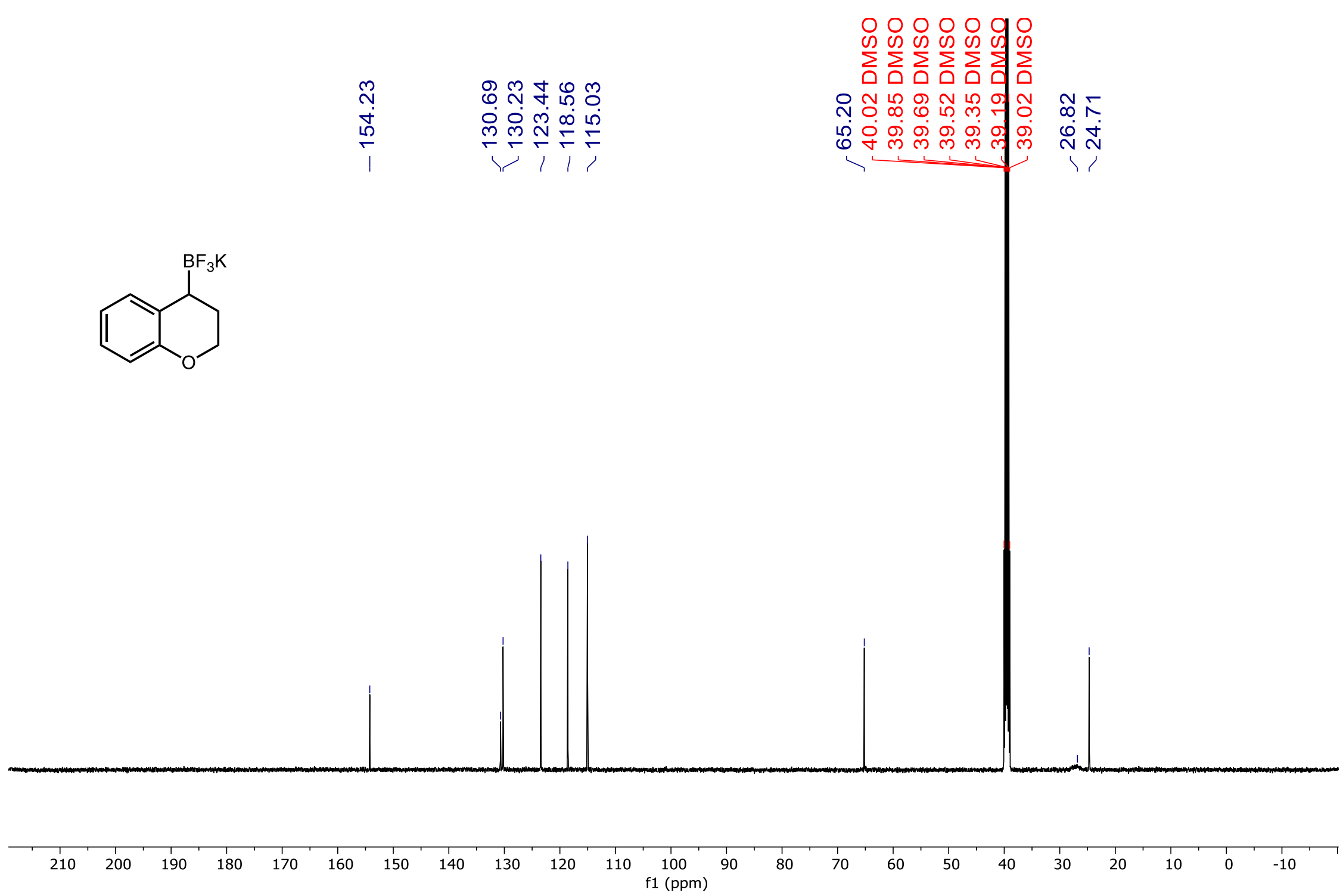

^{1}H NMR (400 MHz, D_2O) of (*2S*, *3S*)-2-amino-2-methyl-3-phenylbutanoic acid (**3a**)

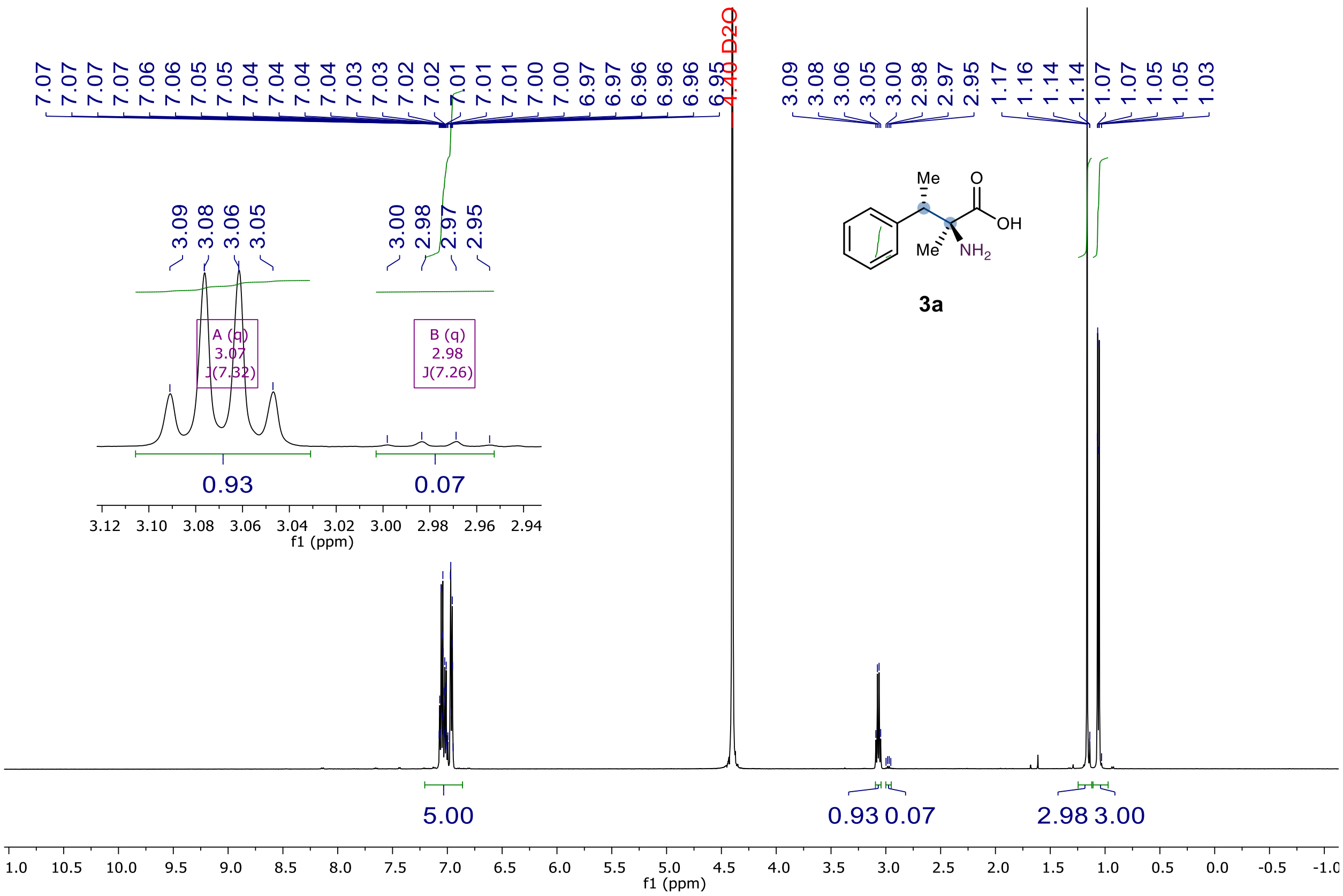

^{13}C NMR (101 MHz, D_2O) of (*2S*, *3S*)-2-amino-2-methyl-3-phenylbutanoic acid (**3a**)

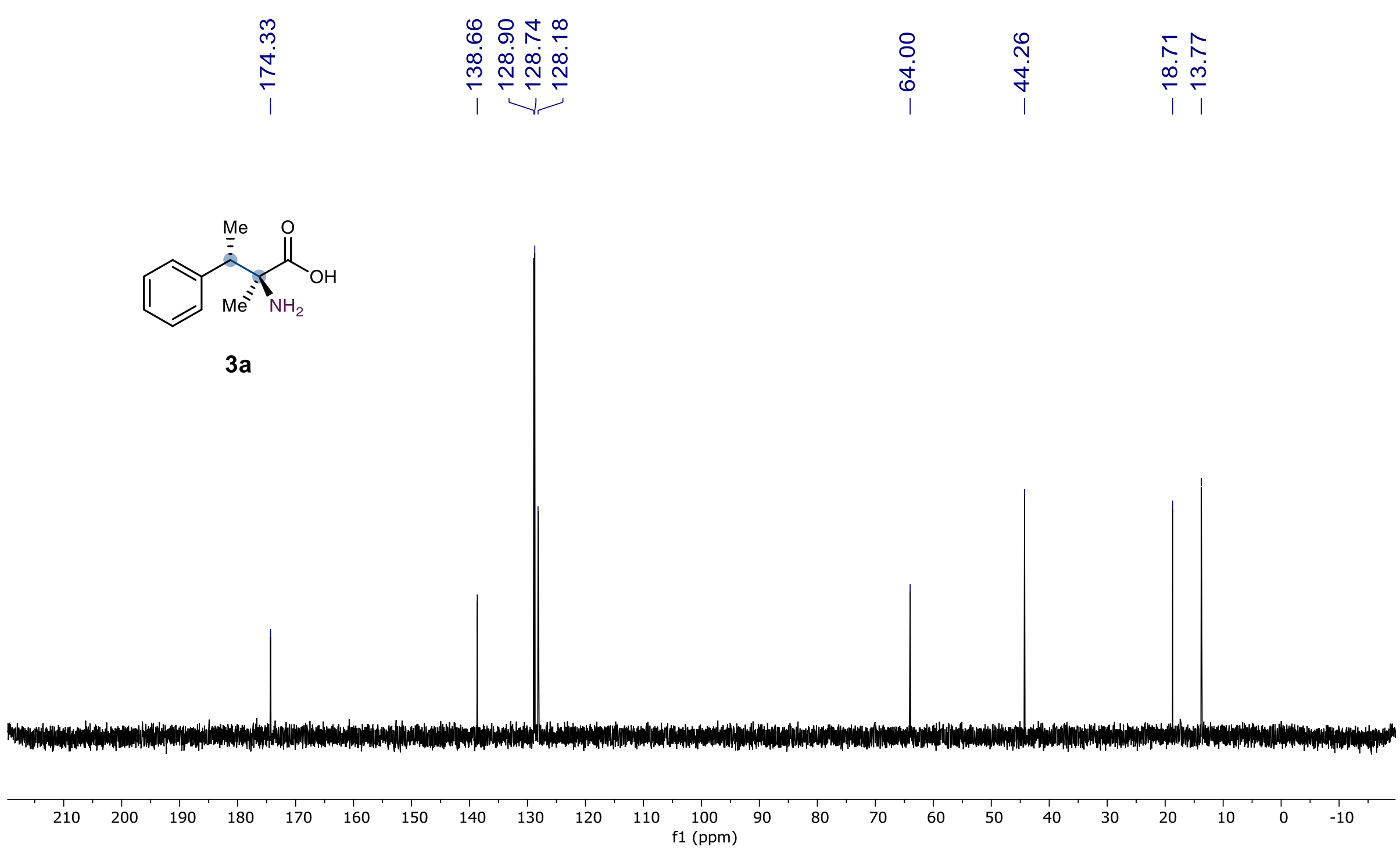

^{1}H NMR (400 MHz, D_2O) of (*2S*, *3S*)-2-amino-2-methyl-3-(o-tolyl)butanoic acid (**3b**)

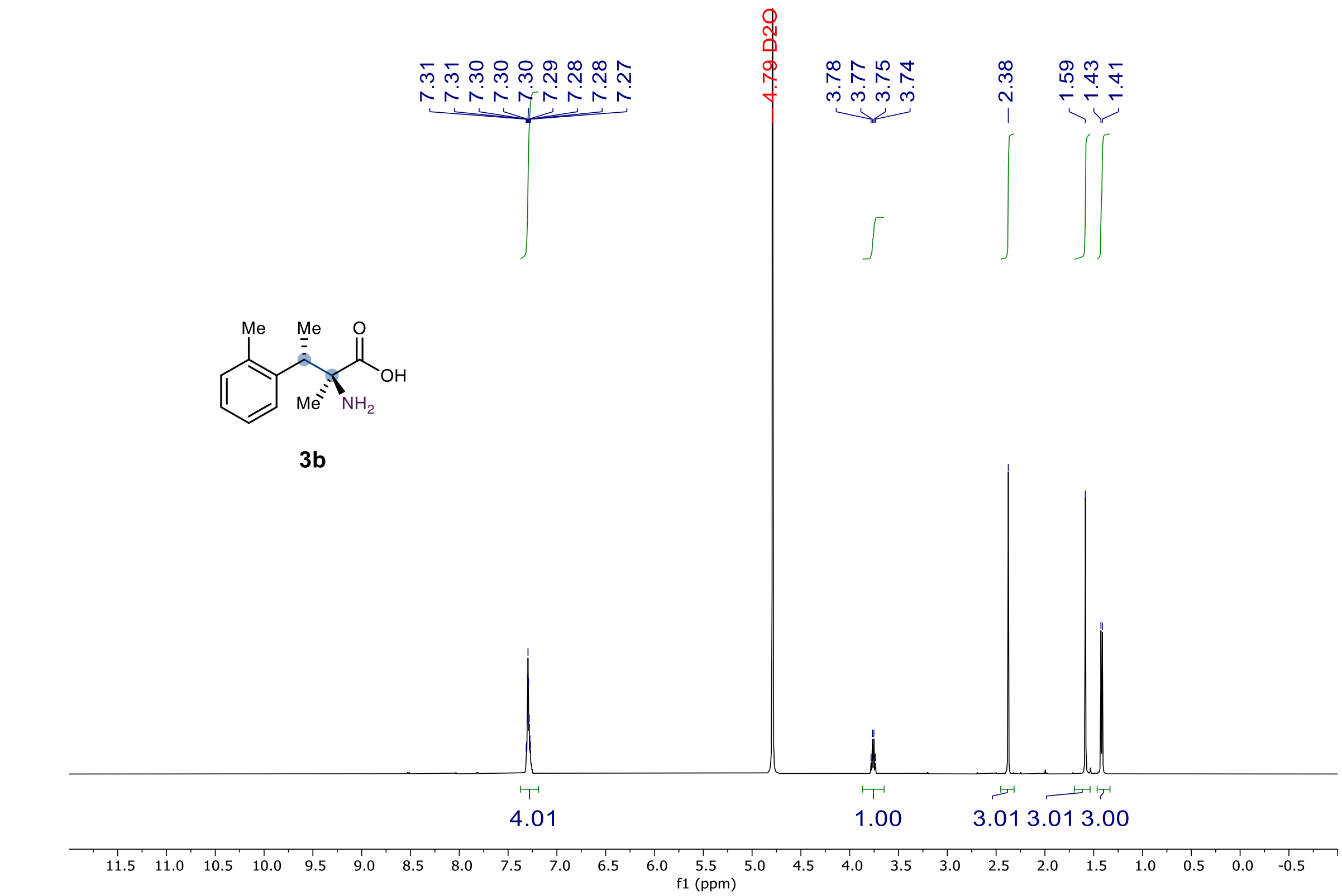

^{13}C NMR (101 MHz, D_2O) of (*2S*, *3S*)-2-amino-2-methyl-3-(o-tolyl)butanoic acid (**3b**)

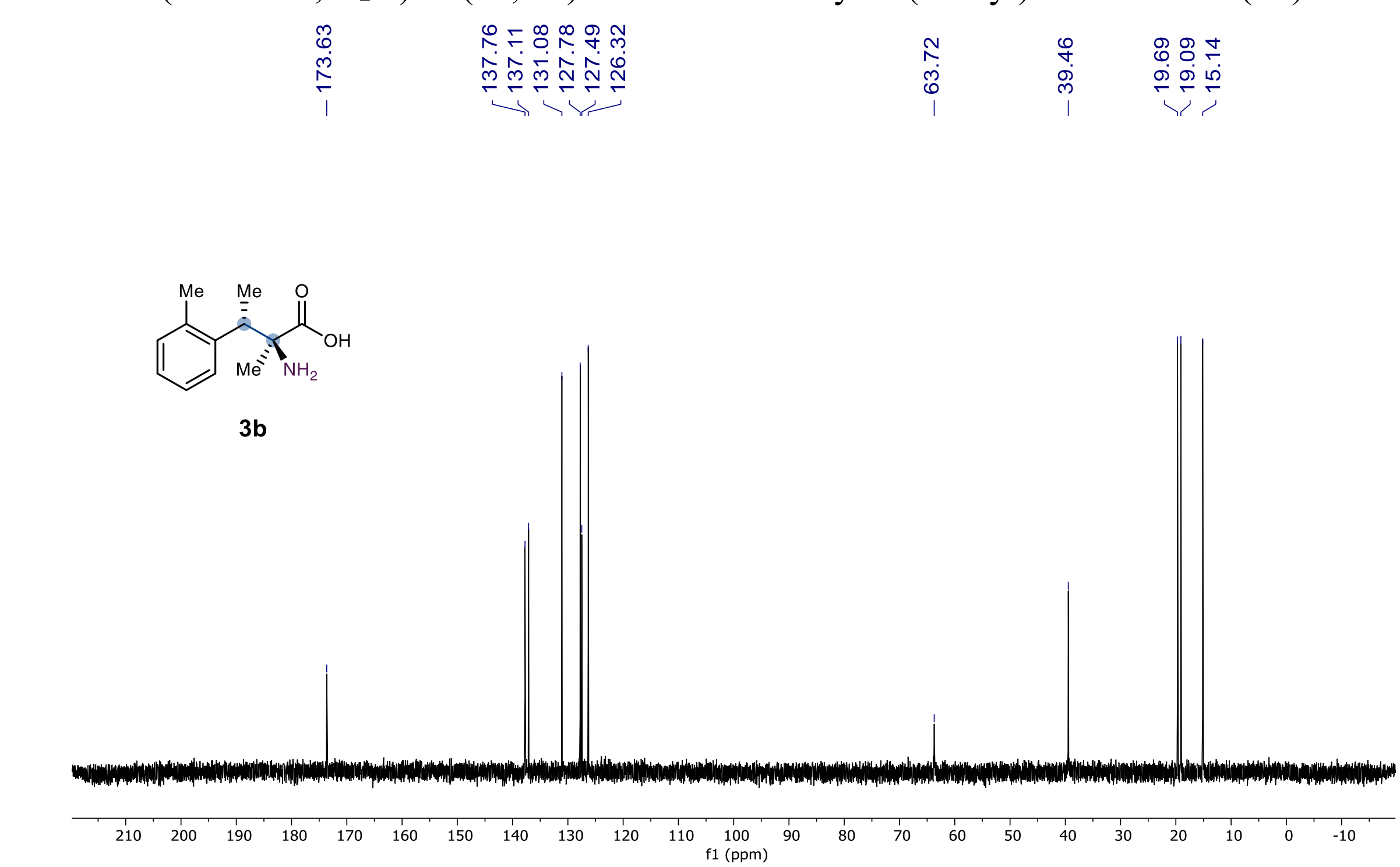

^{1}H NMR (400 MHz, D_2O) of (*2S*, *3S*)-2-amino-2-methyl-3-(m-tolyl)butanoic acid (**3c**)

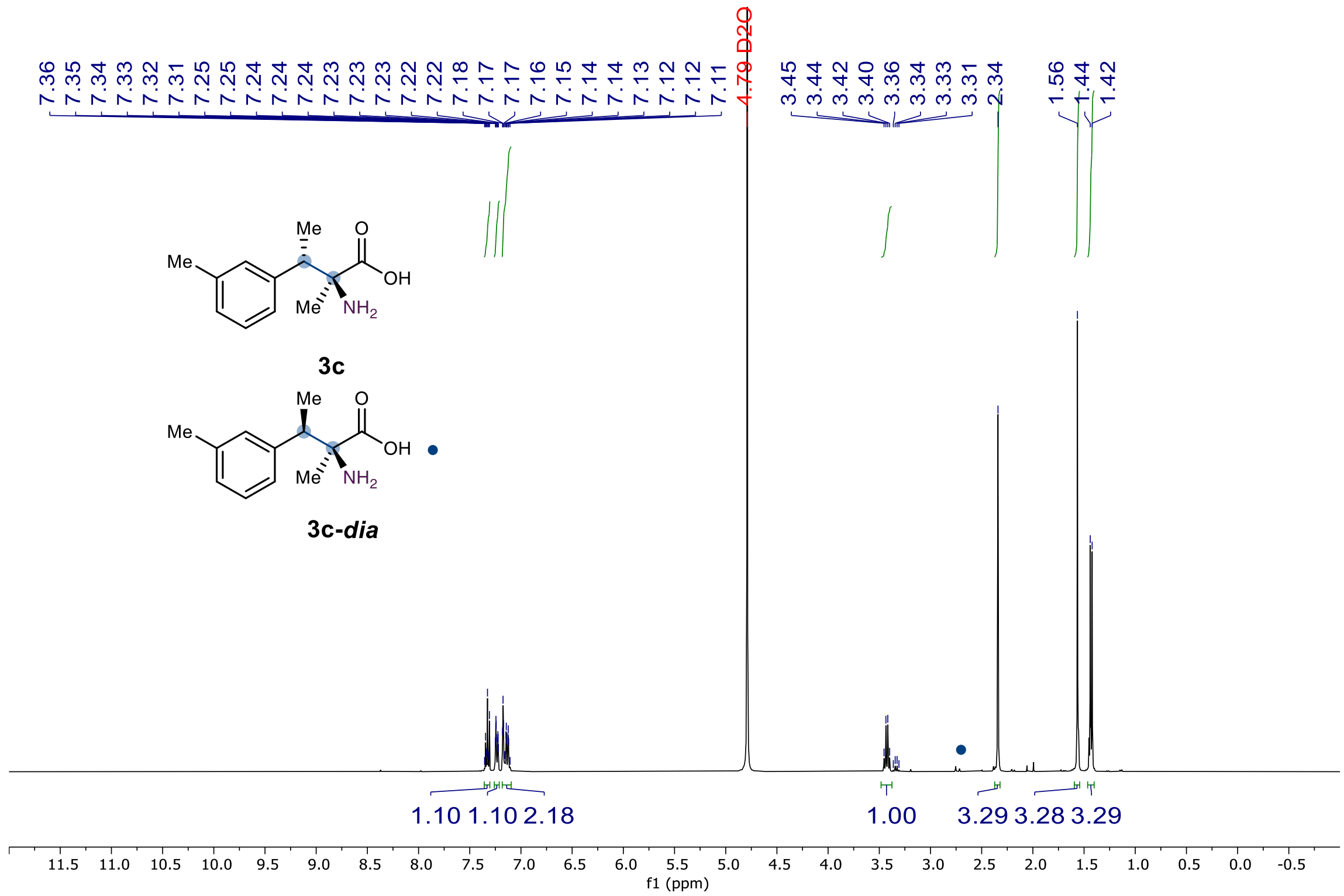

^{13}C NMR (101 MHz, D_2O) of (*2S*, *3S*)-2-amino-2-methyl-3-(m-tolyl)butanoic acid (**3c**)

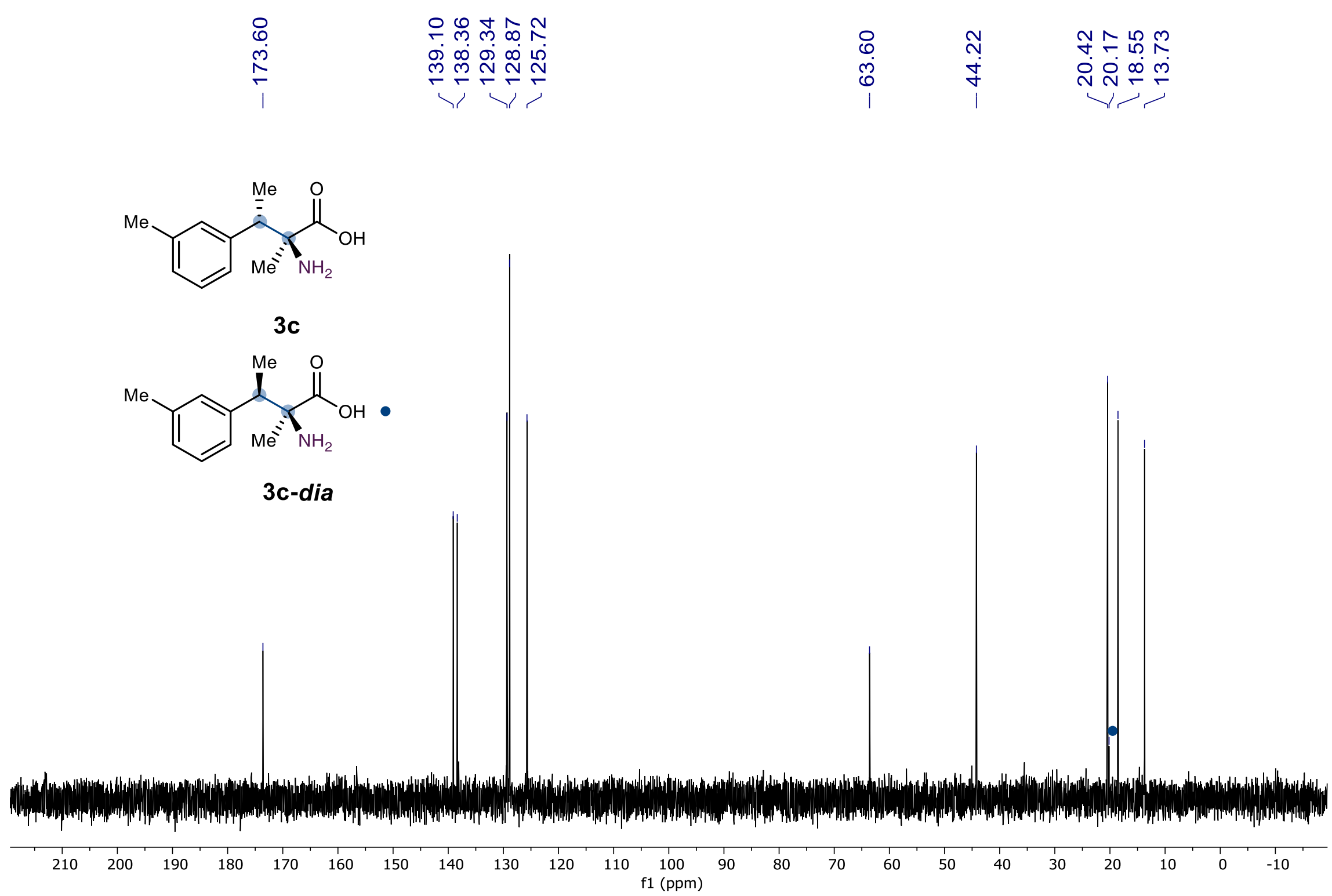

^{1}H NMR (400 MHz, D_2O) of (*2S*, *3S*)-2-amino-2-methyl-3-(p-tolyl)butanoic acid (**3d**)

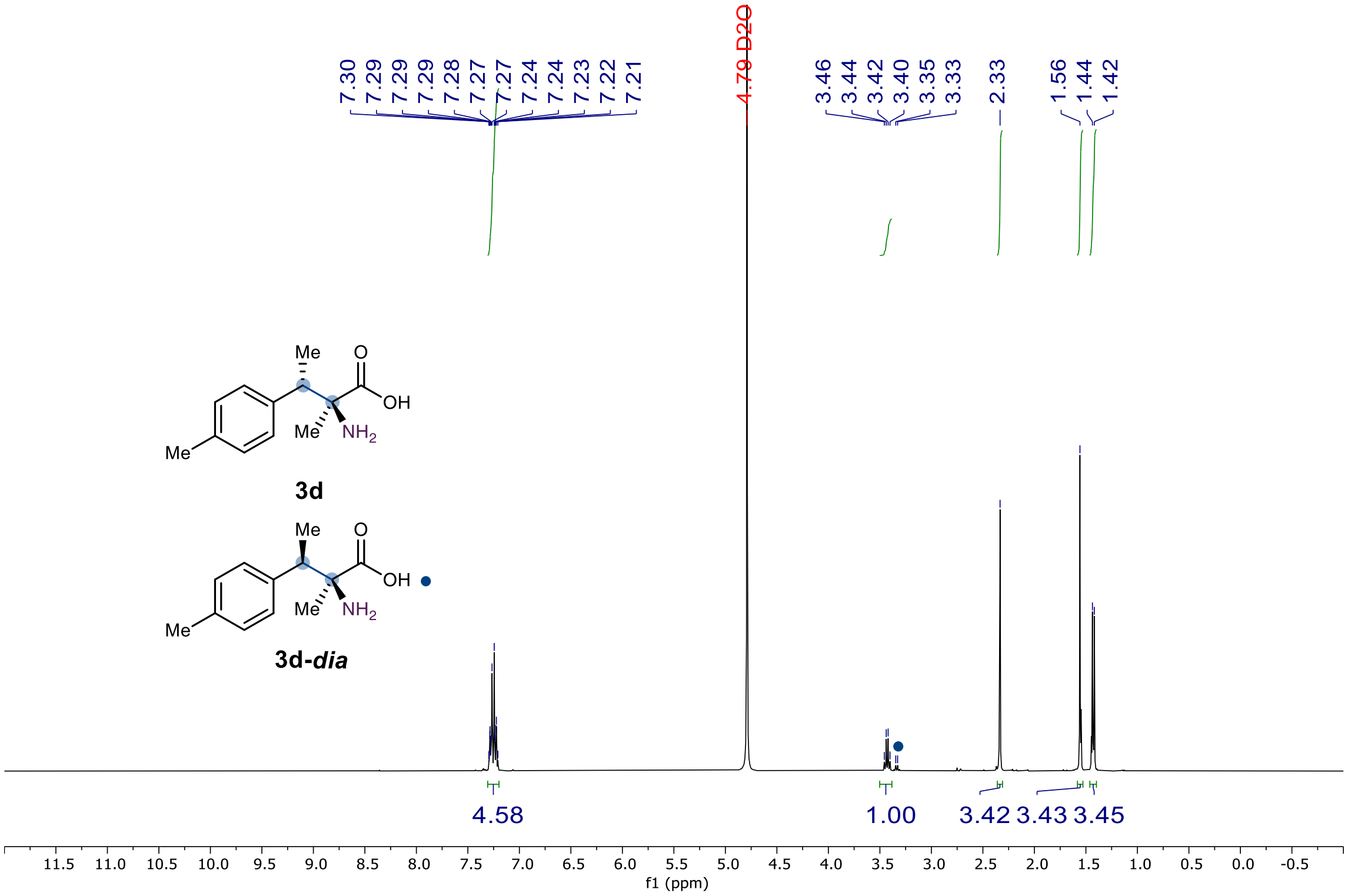

^{13}C NMR (101 MHz, D_2O) of (*2S*, *3S*)-2-amino-2-methyl-3-(p-tolyl)butanoic acid (**3d**)

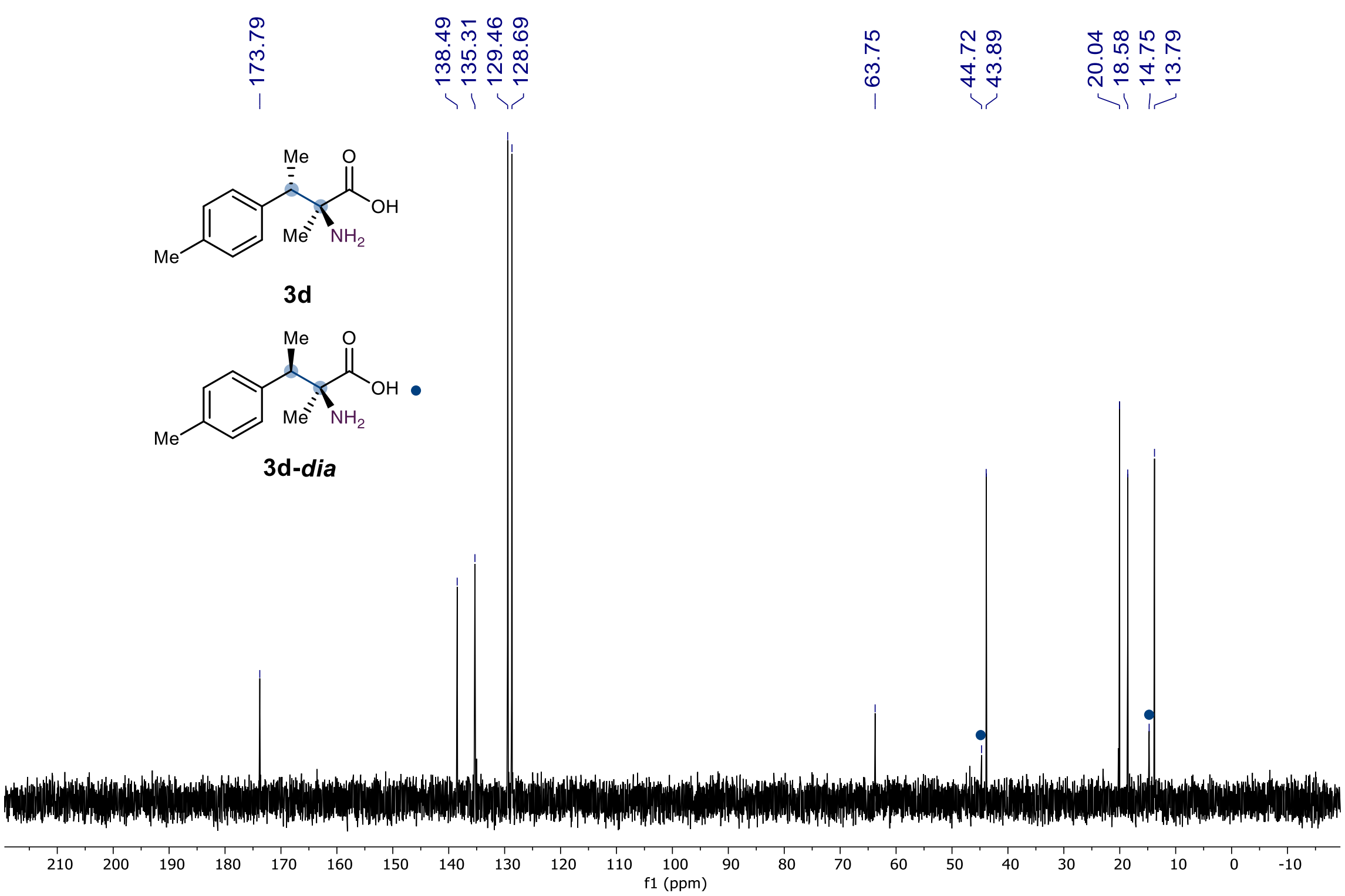

^{1}H NMR (400 MHz, D_2O) of (*2S*, *3S*)-2-amino-3-(4-methoxyphenyl)-2-methylbutanoic acid (**3e**)

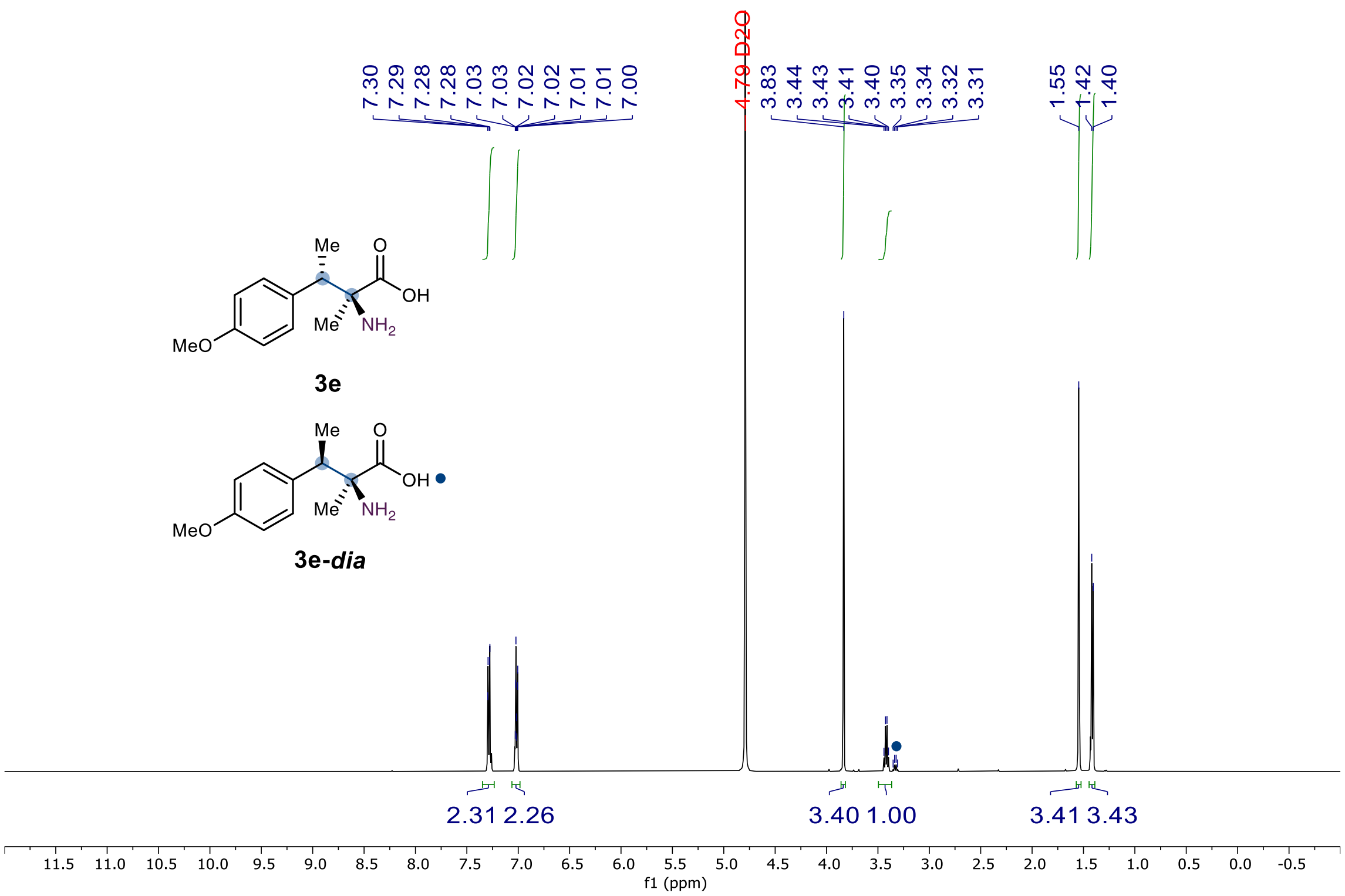

^{13}C NMR (101 MHz, D_2O) of (*2S*, *3S*)-2-amino-3-(4-methoxyphenyl)-2-methylbutanoic acid (**3e**)

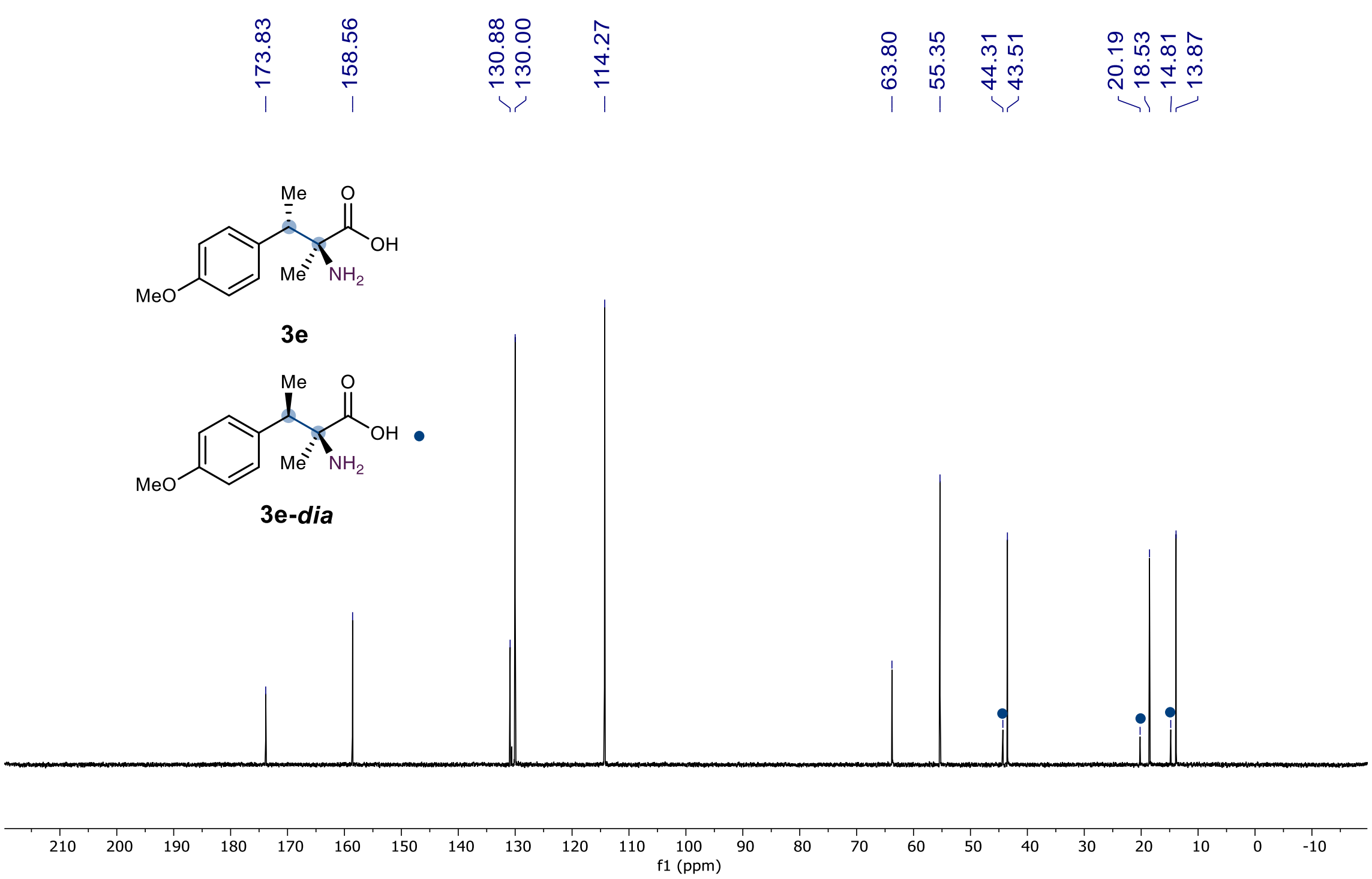

^{1}H NMR (400 MHz, D_2O) of (*2S*, *3S*)-2-amino-3-(4-fluorophenyl)-2-methylbutanoic acid (**3f**)

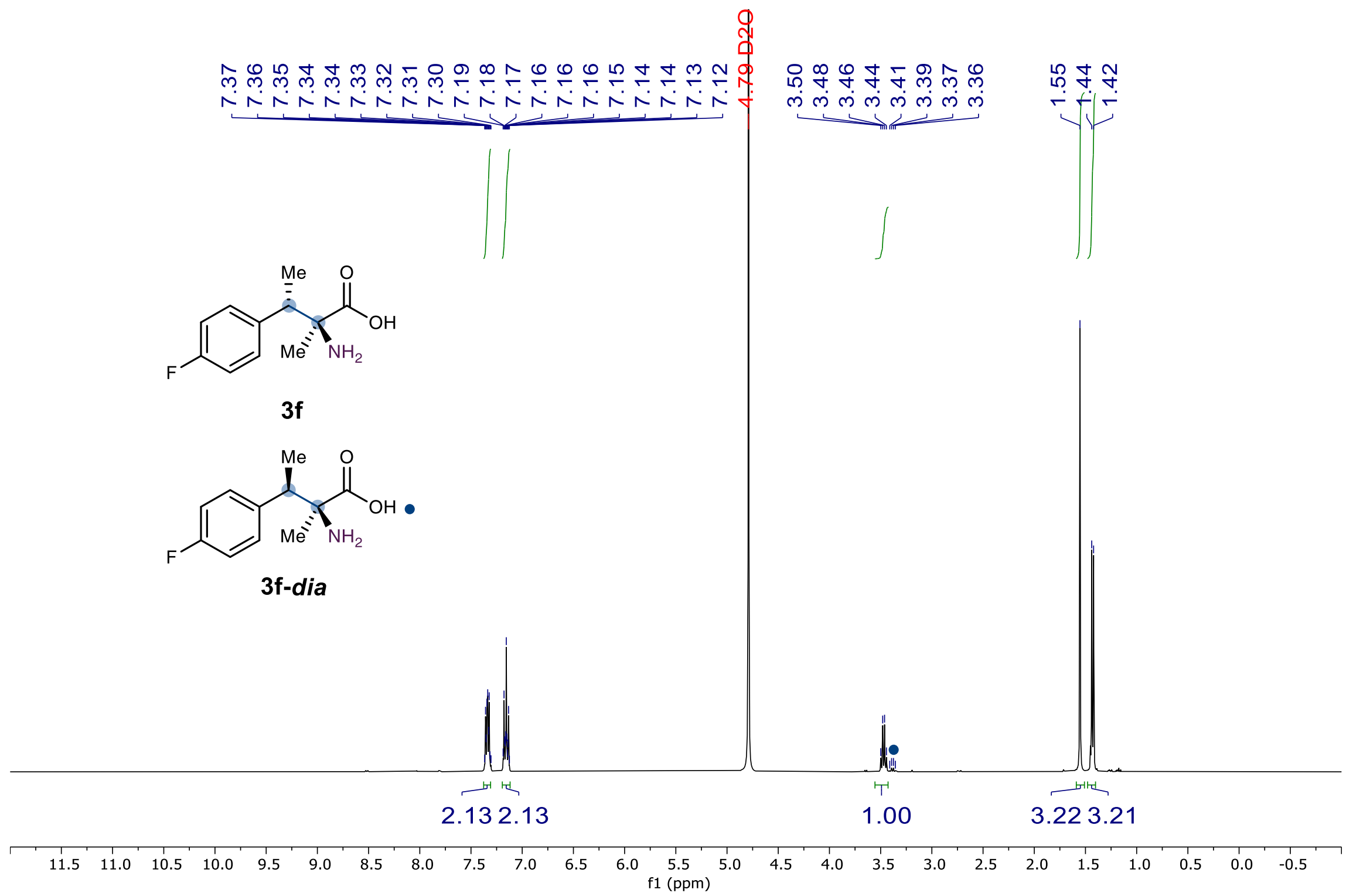

^{13}C NMR (101 MHz, D_2O) of (*2S*, *3S*)-2-amino-3-(4-fluorophenyl)-2-methylbutanoic acid (**3f**)

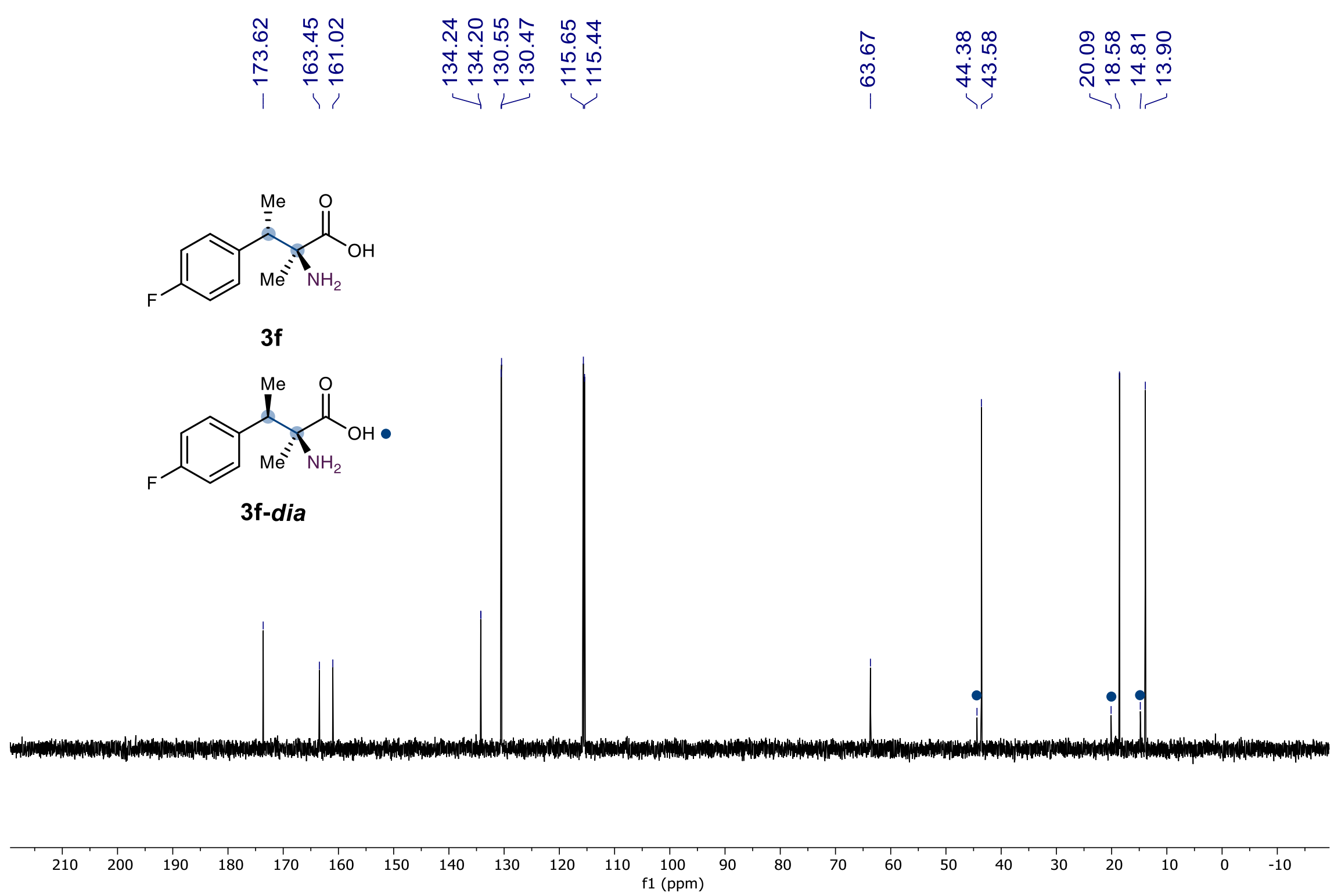

^{19}F NMR (376 MHz, D_2O) of (*2S*, *3S*)-2-amino-3-(4-fluorophenyl)-2-methylbutanoic acid (**3f**)

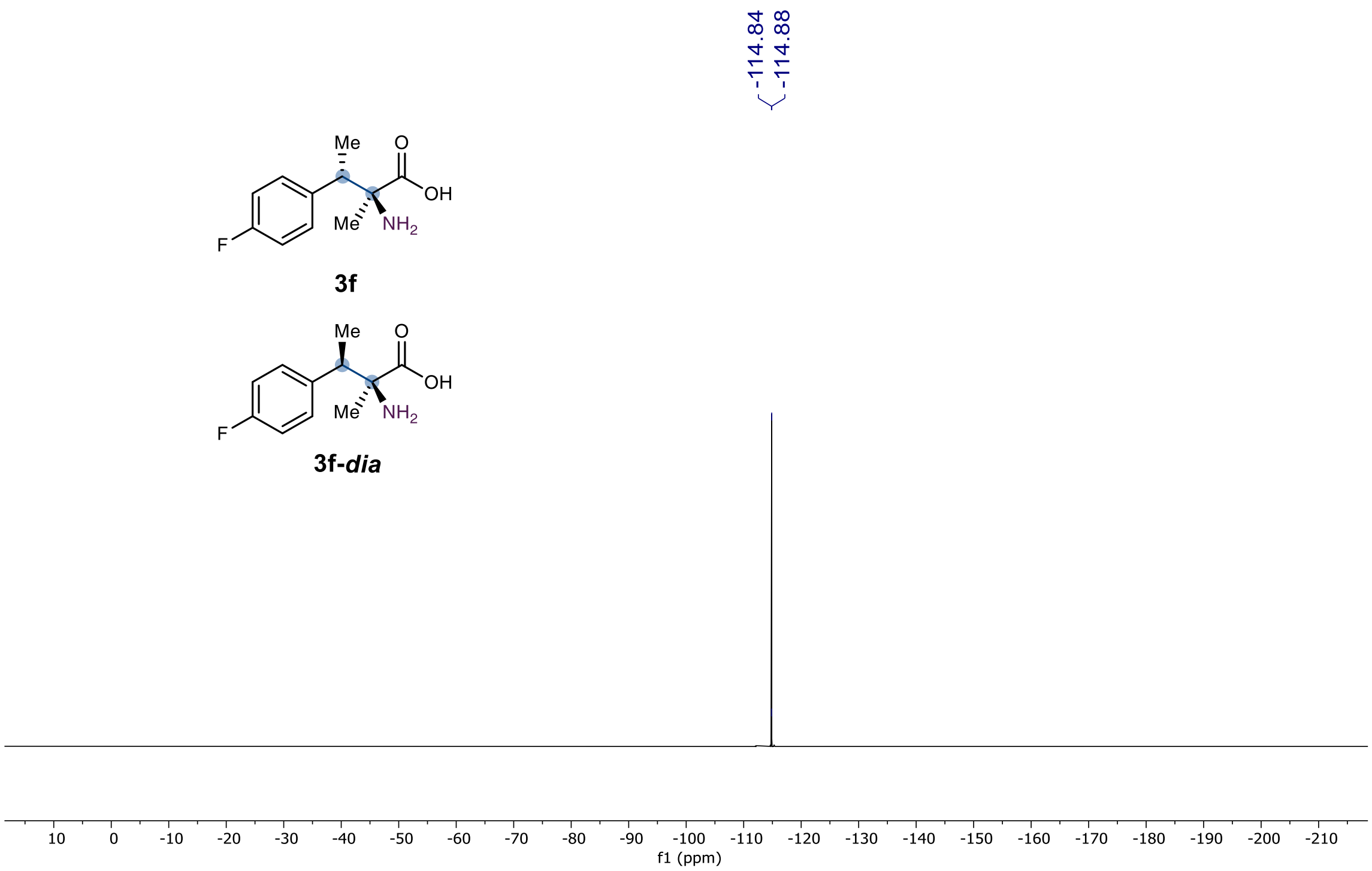

^{1}H NMR (400 MHz, D_2O) of (*2S*, *3S*)-2-amino-3-(4-chlorophenyl)-2-methylbutanoic acid (**3g**)

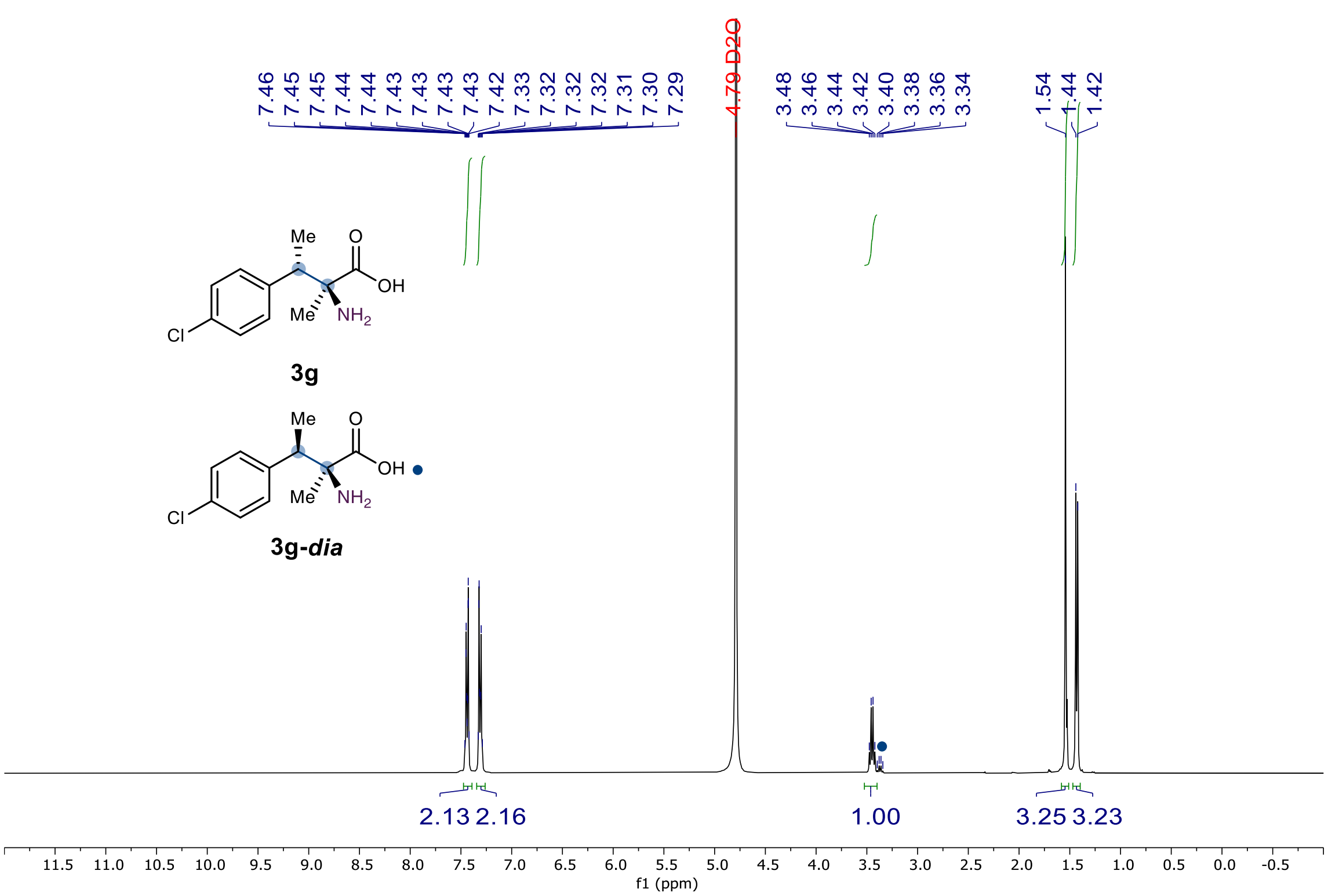

^{13}C NMR (101 MHz, D_2O) of (*2S*, *3S*)-2-amino-3-(4-chlorophenyl)-2-methylbutanoic acid (**3g**)

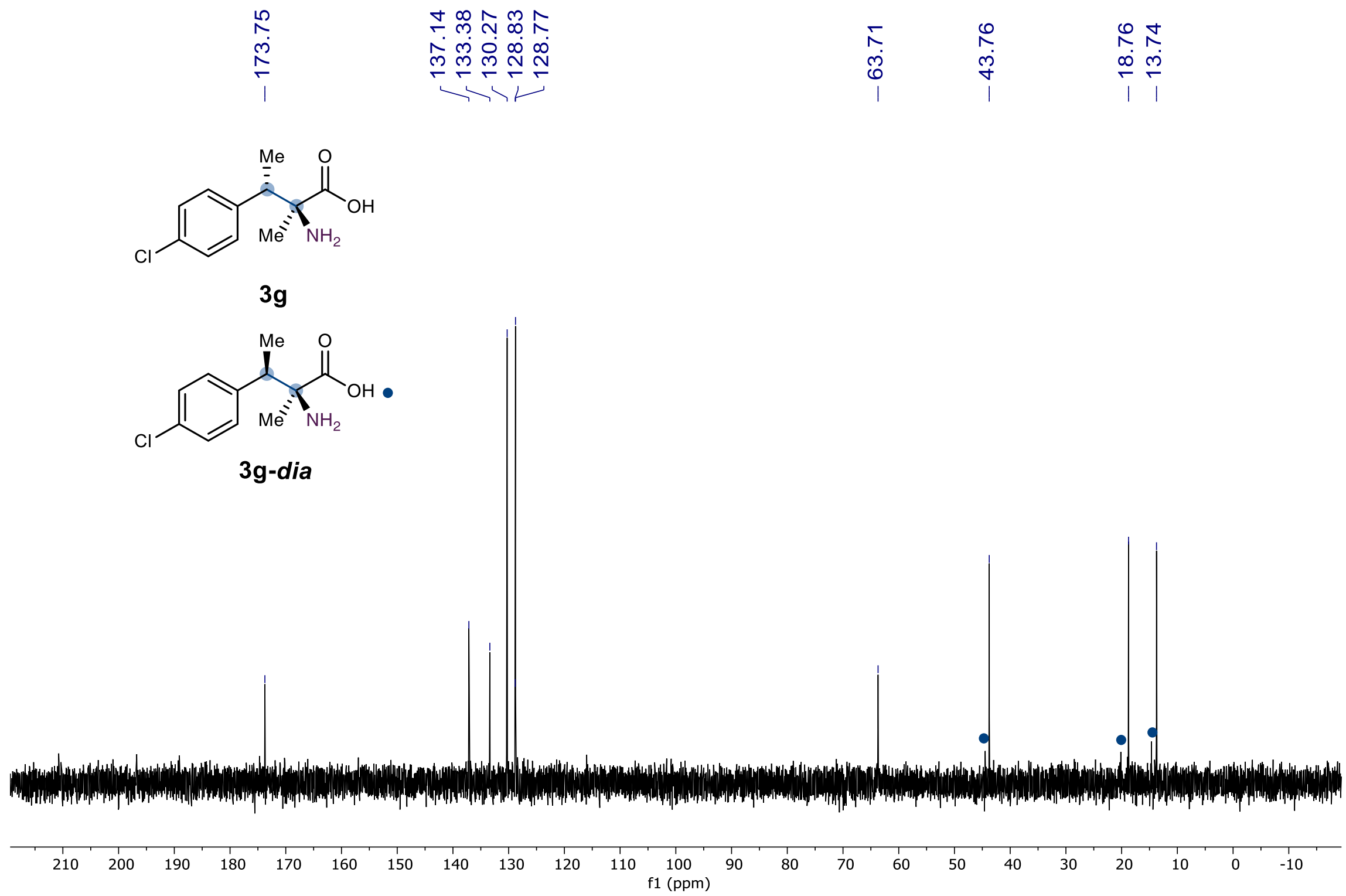

^{1}H NMR (400 MHz, D_2O) of (*2S*, *3S*)-2-amino-3-(4-bromophenyl)-2-methylbutanoic acid (**3h**)

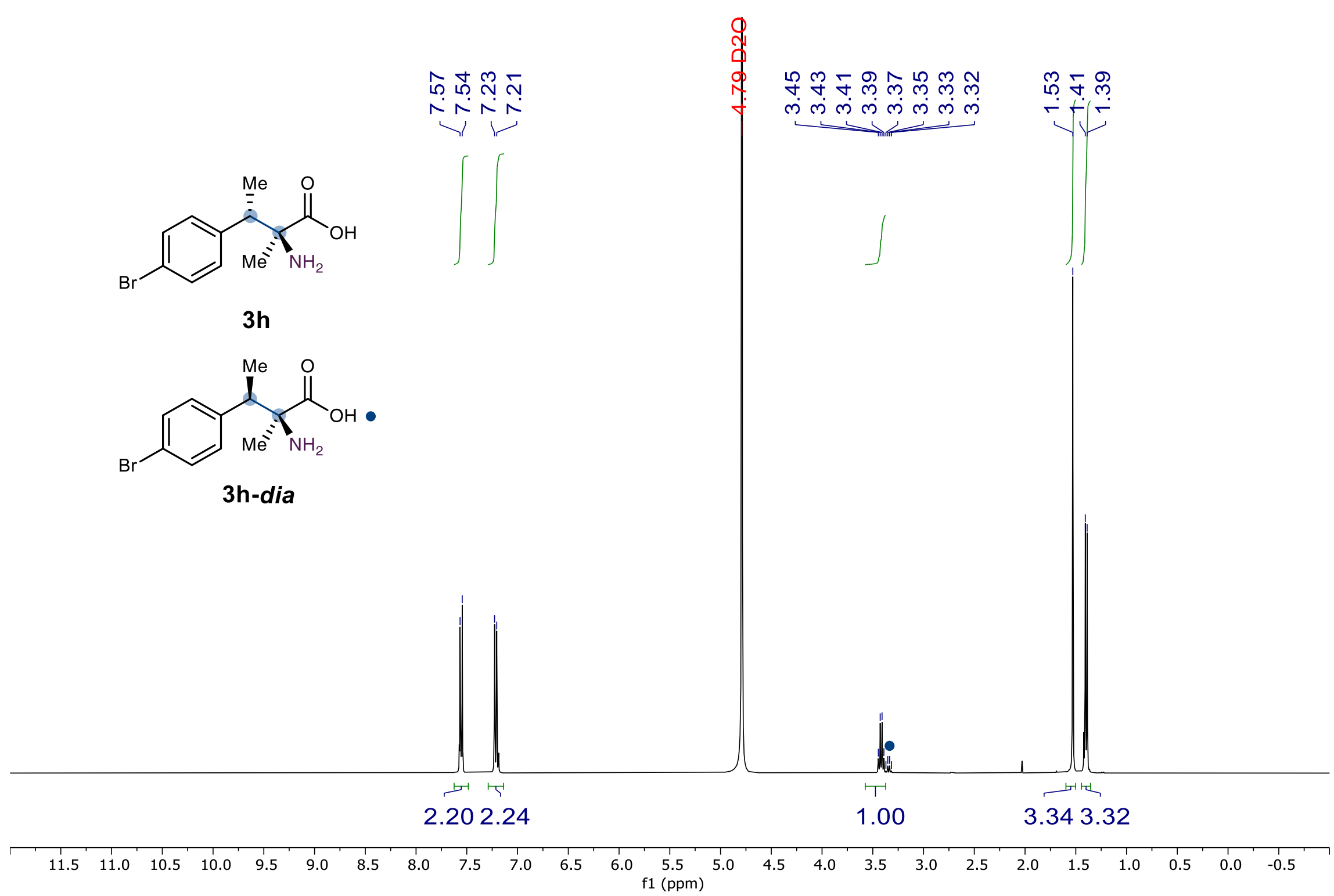

^{13}C NMR (101 MHz, D_2O) of (*2S*, *3S*)-2-amino-3-(4-bromophenyl)-2-methylbutanoic acid (**3h**)

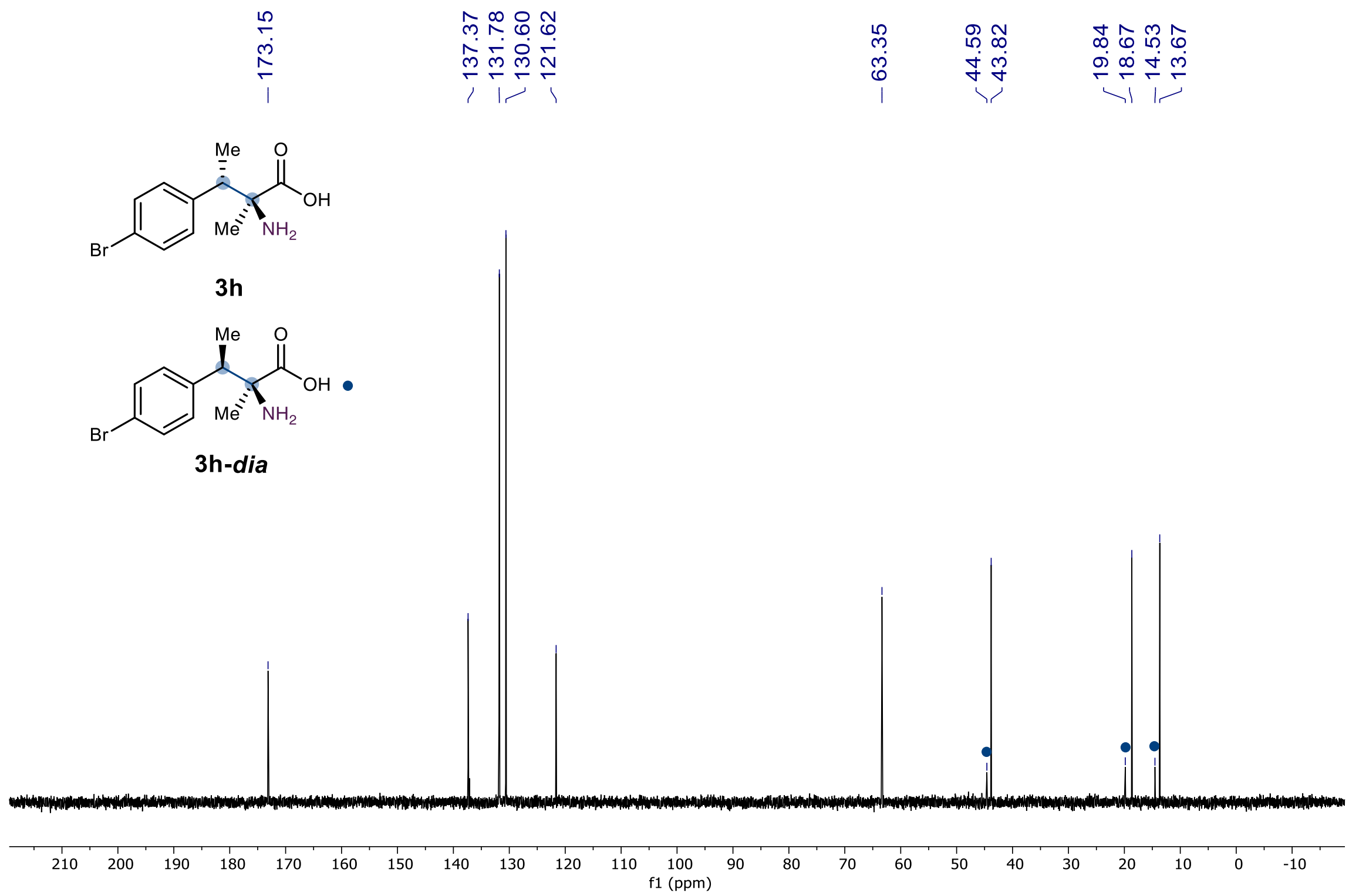

^{1}H NMR (400 MHz, D_2O) of (*2S*, *3S*)-2-amino-2-methyl-3-(naphthalen-2-yl)butanoic acid (**3i**)

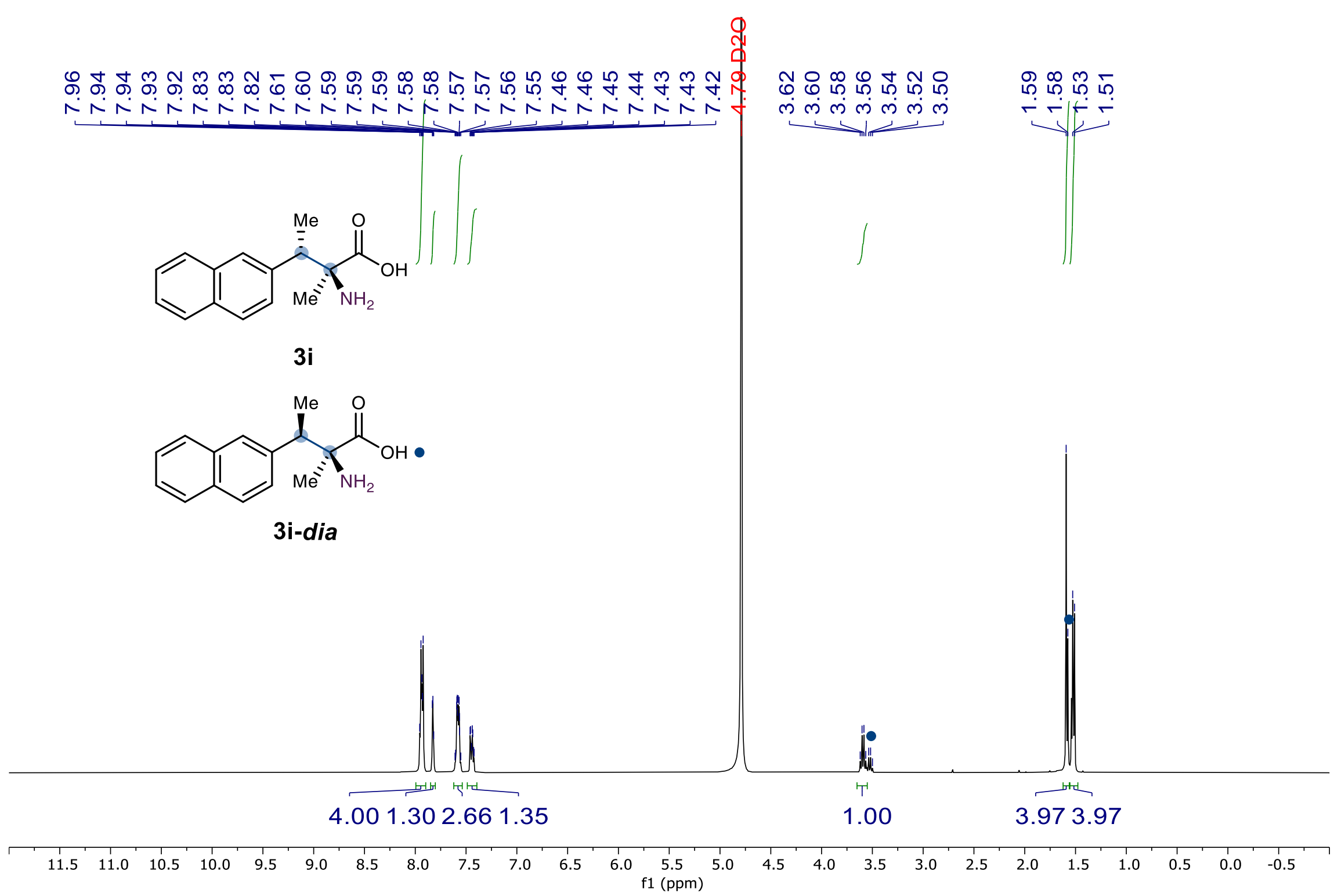

^{13}C NMR (101 MHz, D_2O) of (*2S*, *3S*)-2-amino-2-methyl-3-(naphthalen-2-yl)butanoic acid (**3i**)

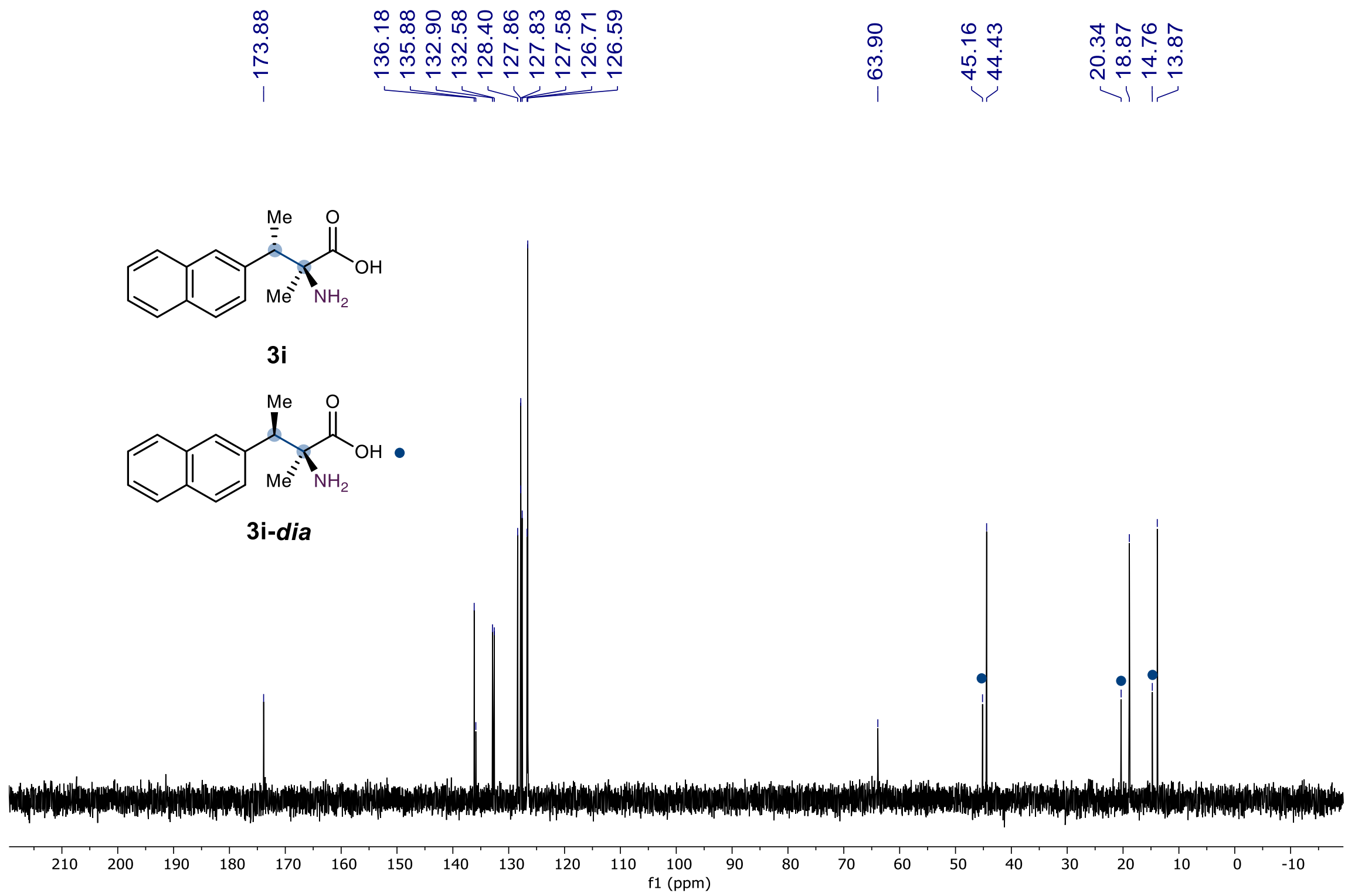

^{1}H NMR (400 MHz, D_2O) of (*2S*, *3S*)-2-amino-2-methyl-3-phenylpentanoic acid (**3j**)

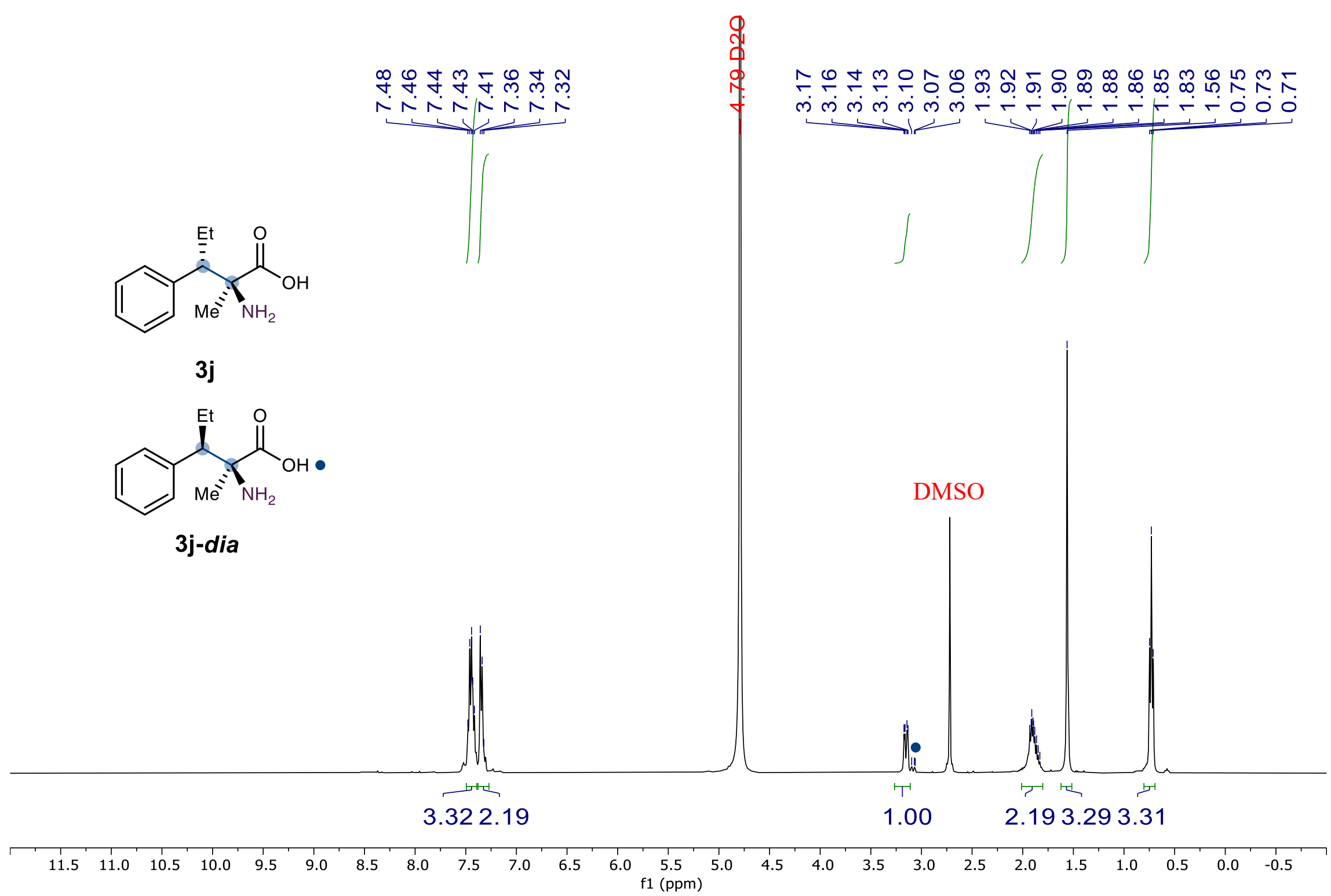

^{13}C NMR (101 MHz, D_2O) of (*2S*, *3S*)-2-amino-2-methyl-3-phenylpentanoic acid (**3j**)

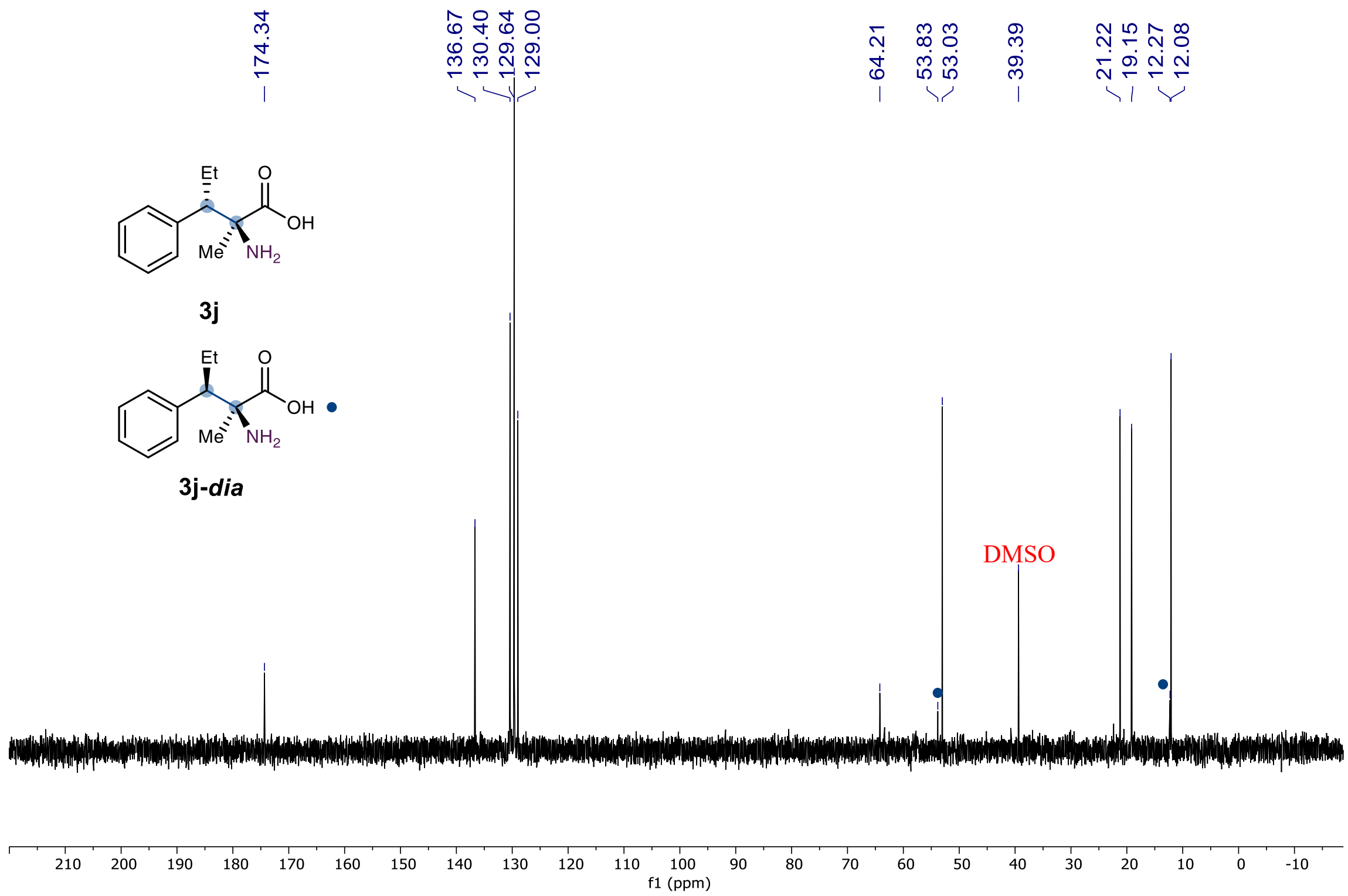

^{1}H NMR (400 MHz, D_2O) of (*2S*, *3S*)-2-amino-2-methyl-3-phenylhexanoic acid (**3k**)

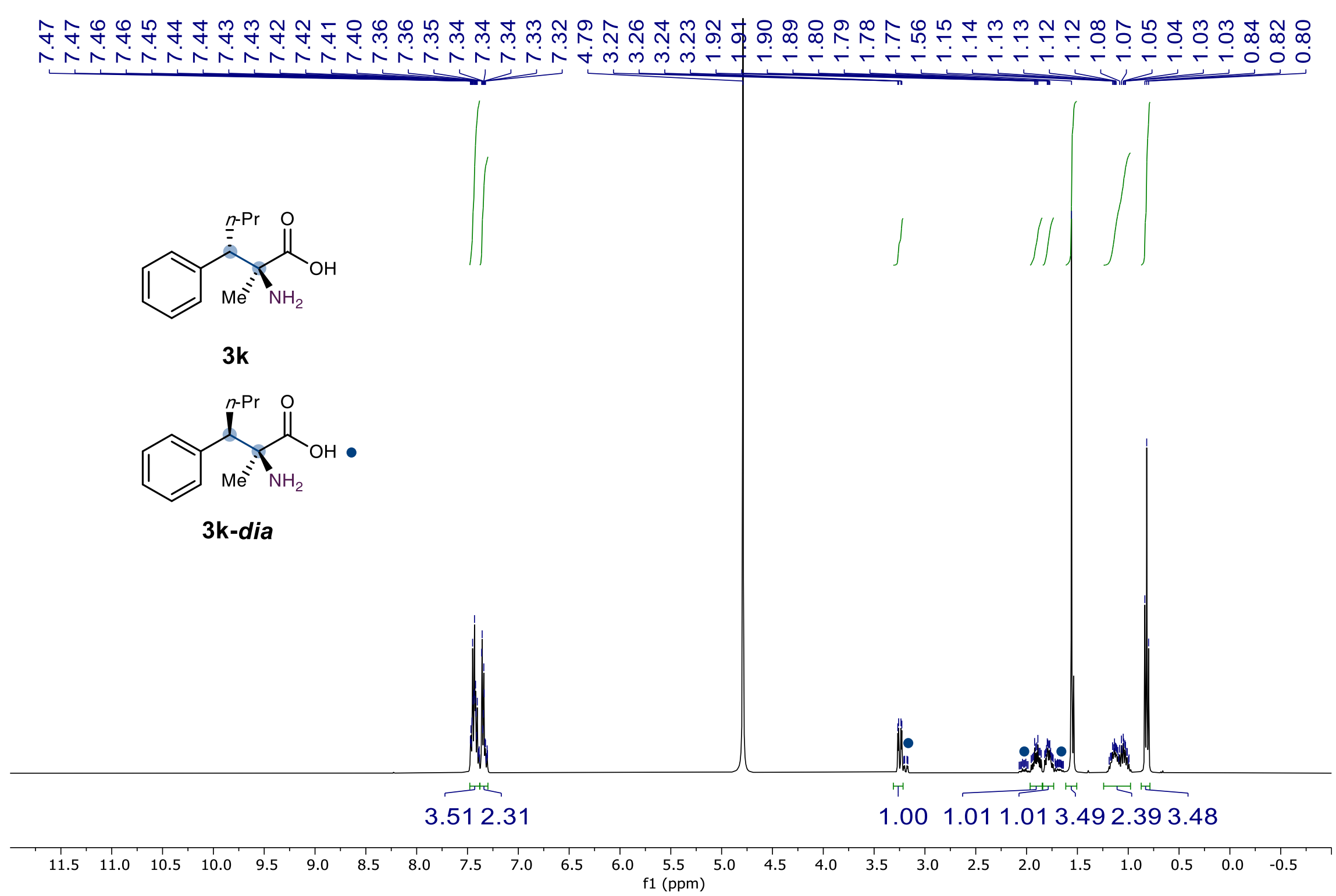

^{13}C NMR (101 MHz, D_2O) of (*2S*, *3S*)-2-amino-2-methyl-3-phenylhexanoic acid (**3k**)

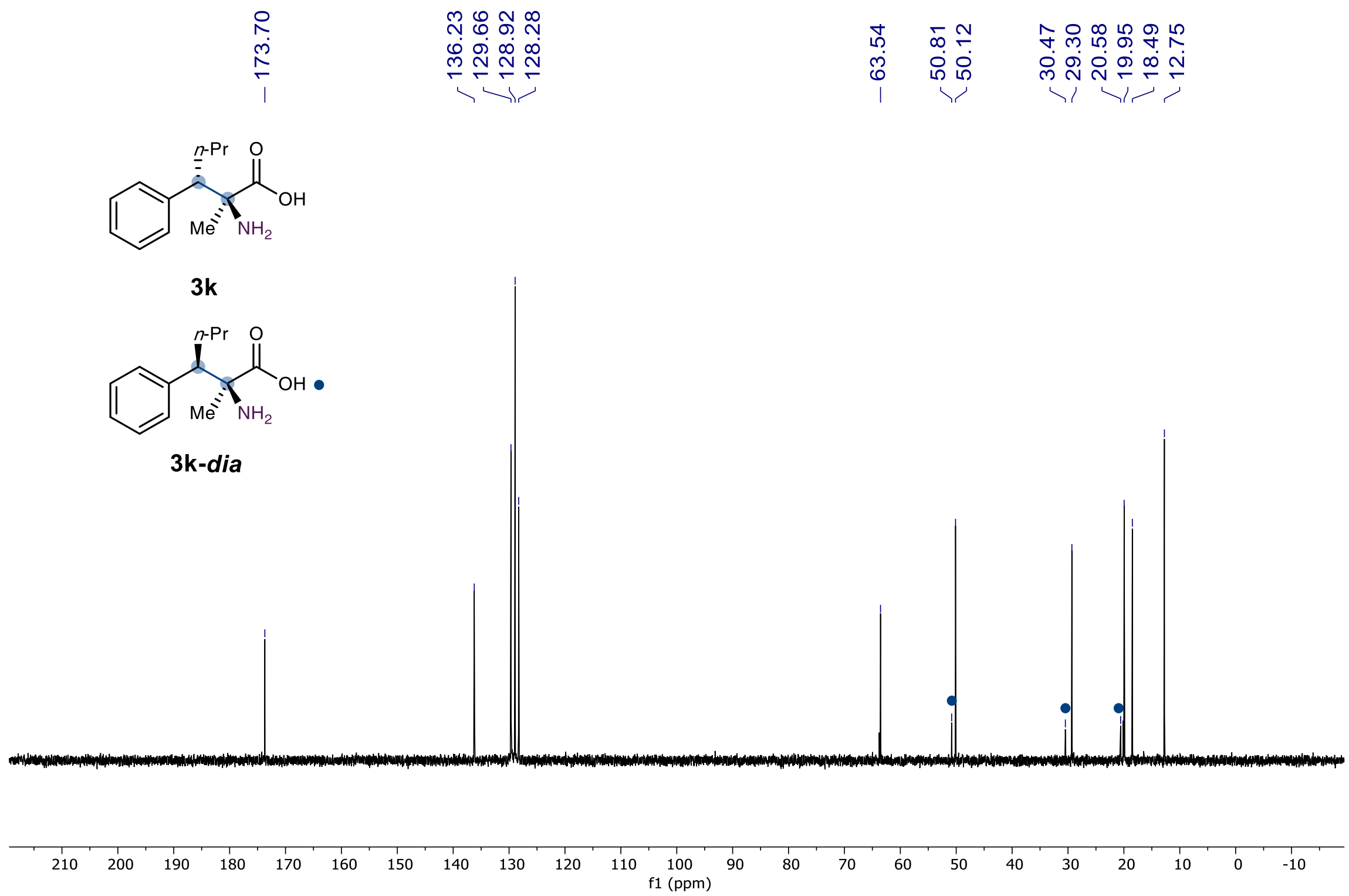

^{1}H NMR (400 MHz, D_2O) of (*S*)-2-amino-2-((*S*)-2,3-dihydro-1H-inden-1-yl)propanoic acid (**3l**)

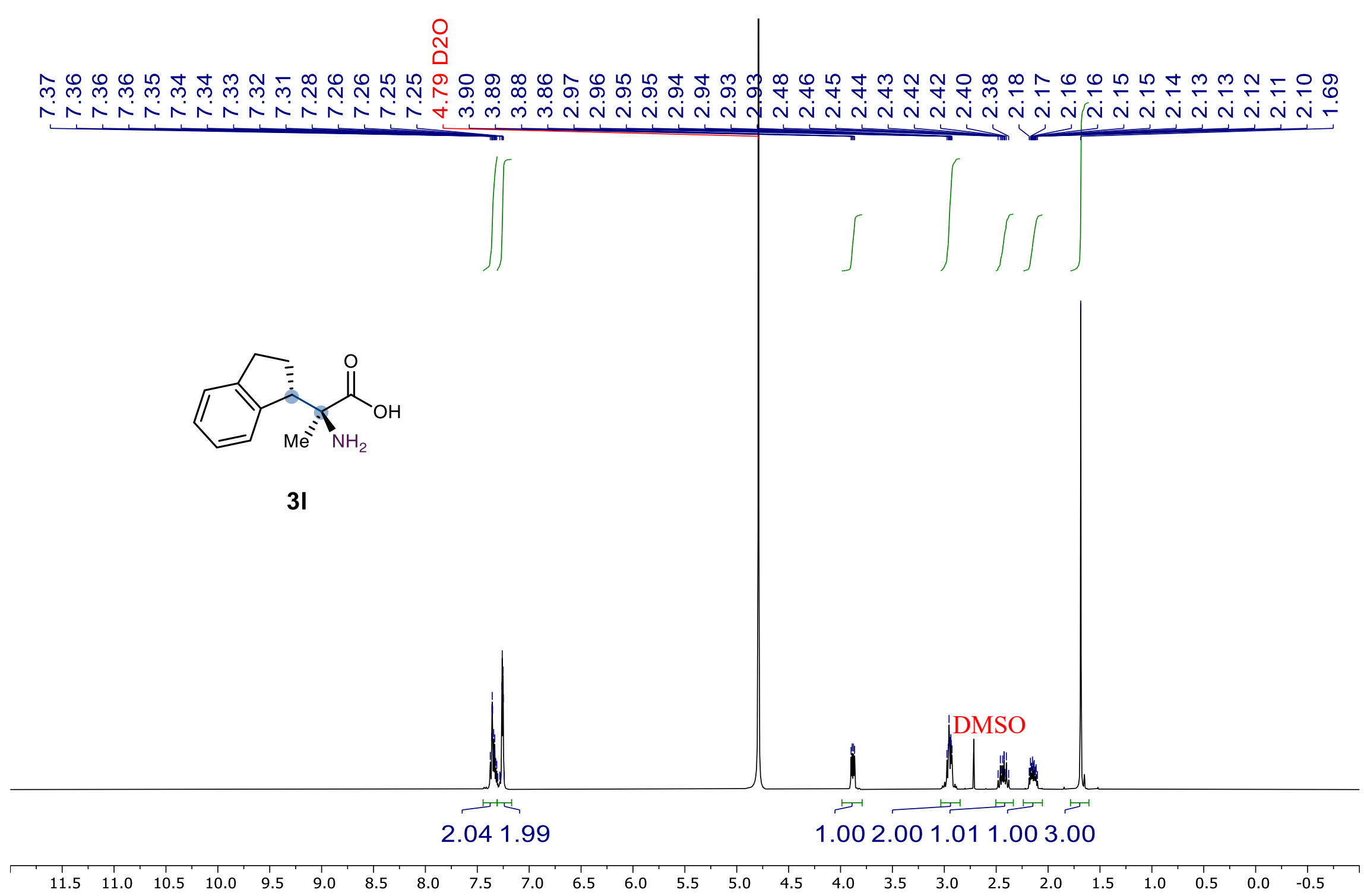

^{13}C NMR (101 MHz, D_2O) of (*S*)-2-amino-2-((*S*)-2,3-dihydro-1H-inden-1-yl)propanoic acid (**3l**)

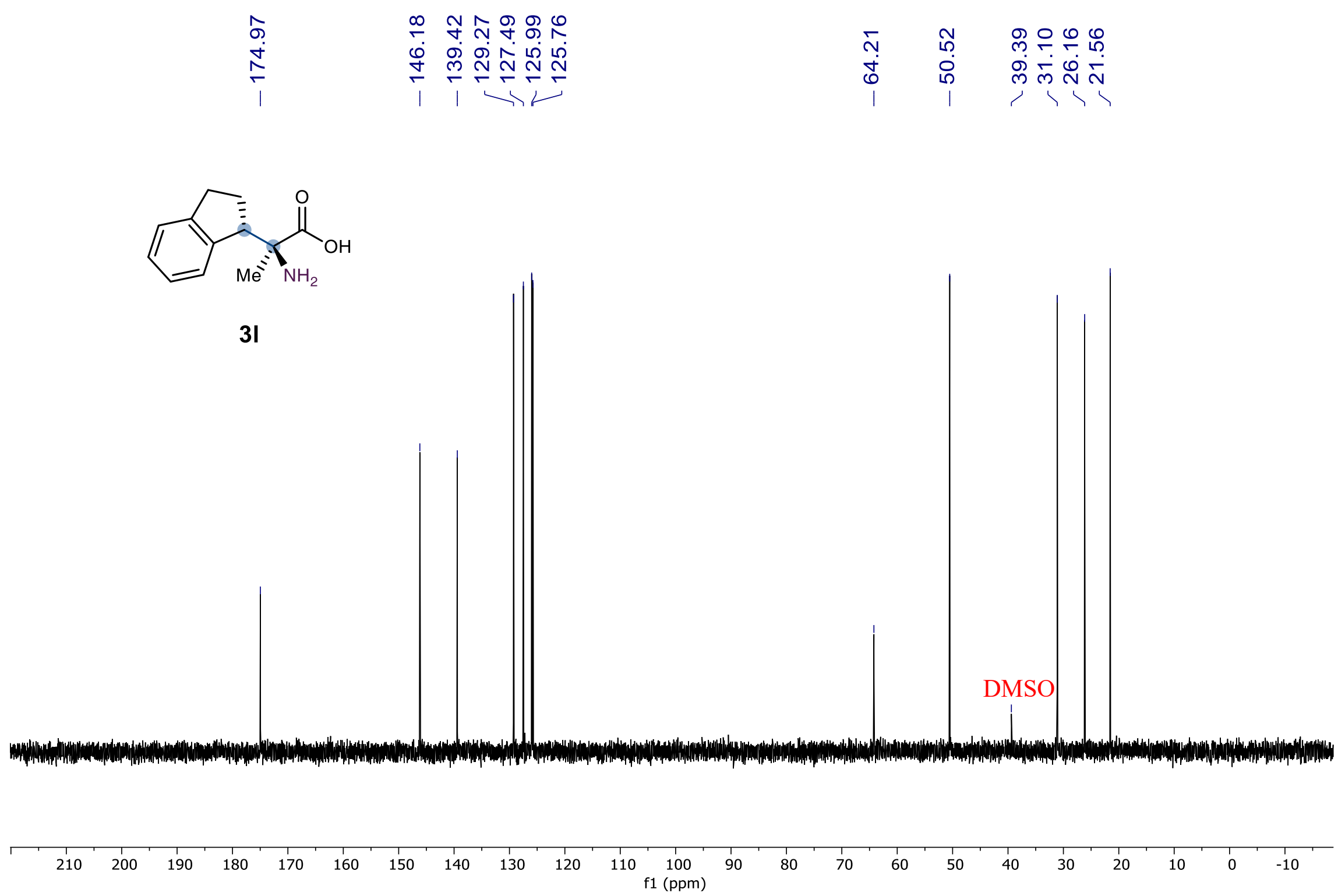

^{1}H NMR (400 MHz, D_2O) of (S)-2-amino-2-((S)-1,2,3,4-tetrahydronaphthalen-1-yl)propanoic acid (**3m**)

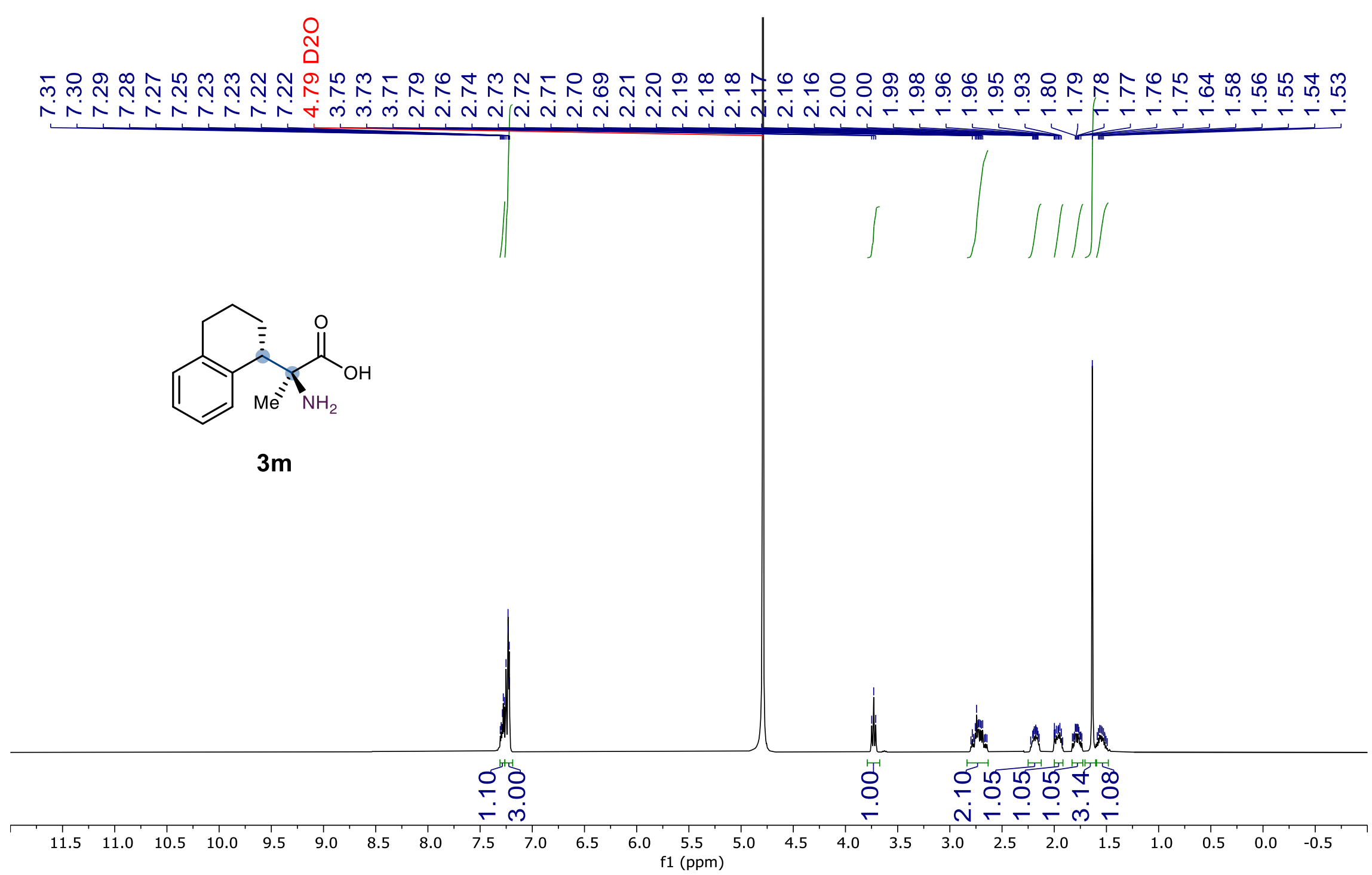

^{13}C NMR (101 MHz, D_2O) of (*S*)-2-amino-2-((*S*)-1,2,3,4-tetrahydronaphthalen-1-yl)propanoic acid (**3m**)

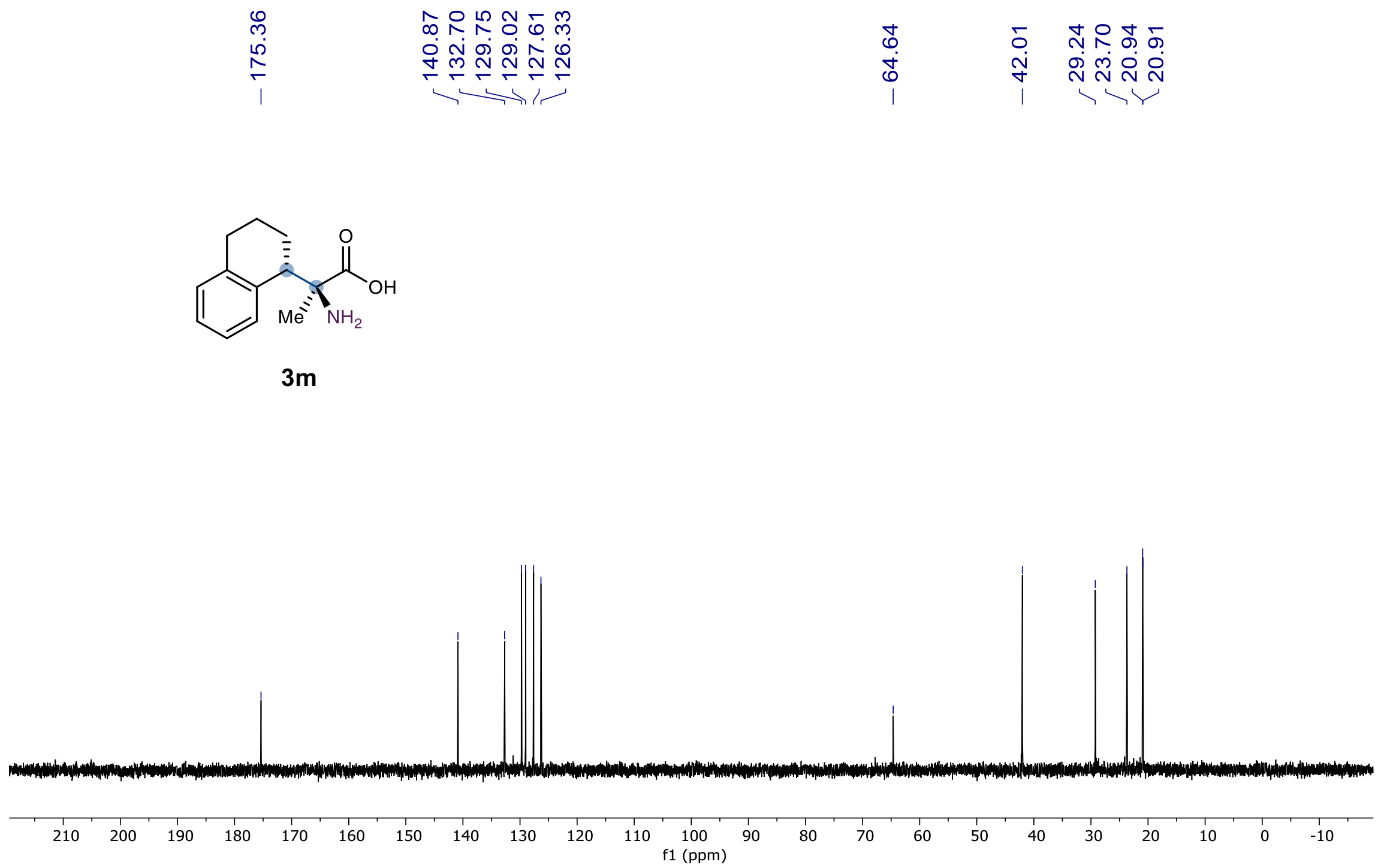

^{1}H NMR (400 MHz, D_2O) of (*S*)-2-amino-2-((*S*)-chroman-4-yl)propanoic acid (**3n**)

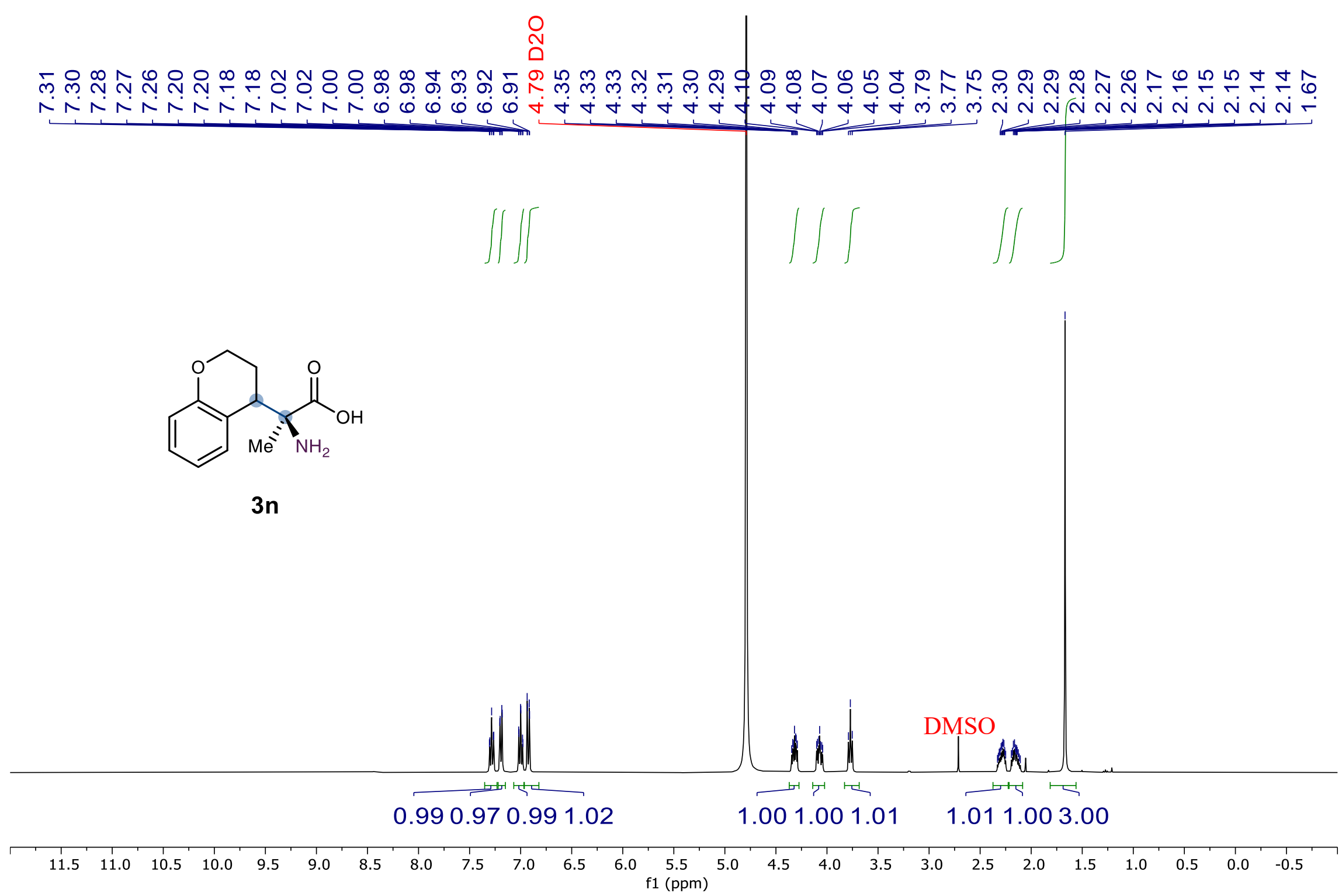

^{13}C NMR (101 MHz, D_2O) of (*S*)-2-amino-2-((*S*)-chroman-4-yl)propanoic acid (**3n**)

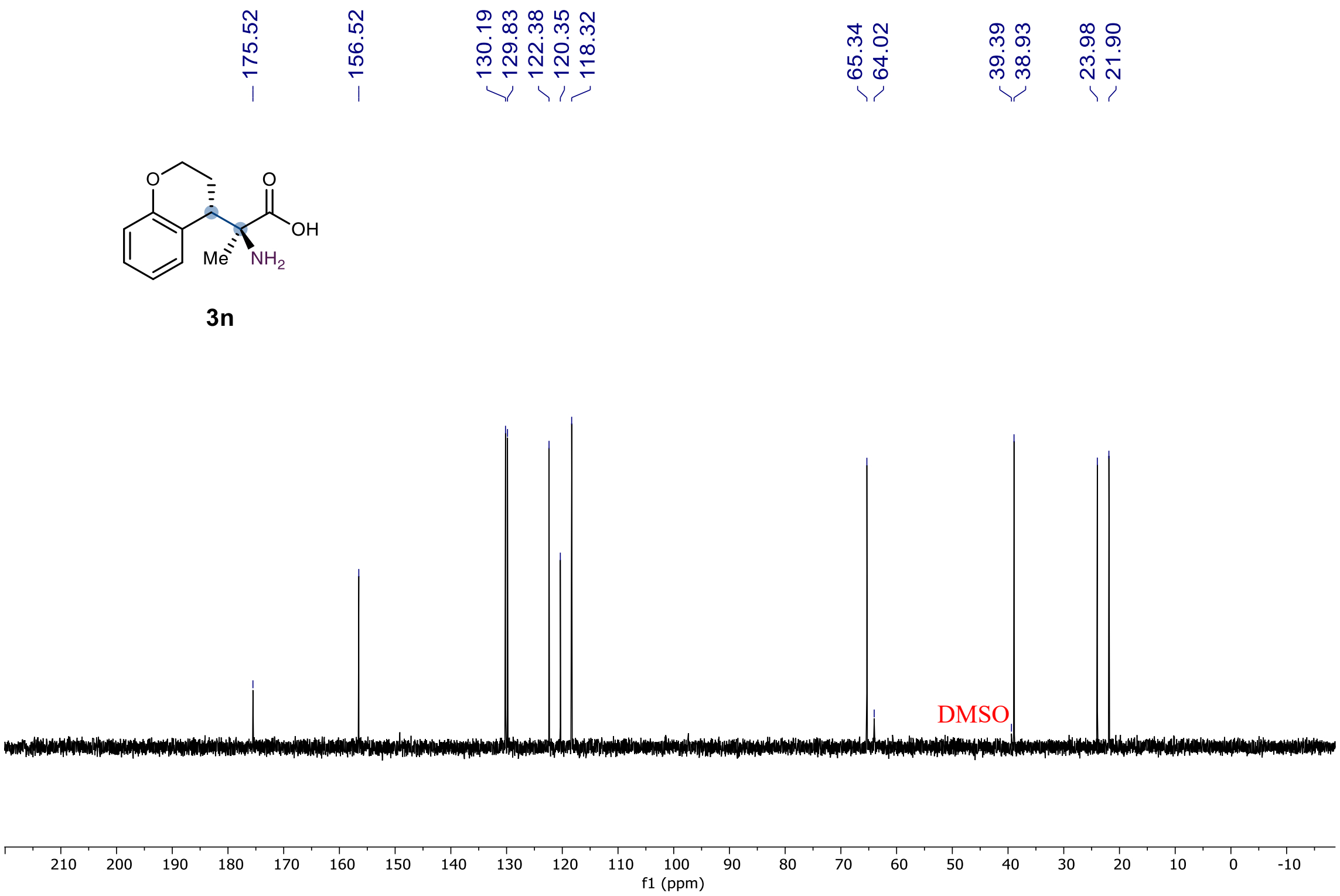

^{1}H NMR (400 MHz, D_2O) of (*2S*, *3S*)-2-amino-2-ethyl-3-phenylbutanoic acid (**3o**)

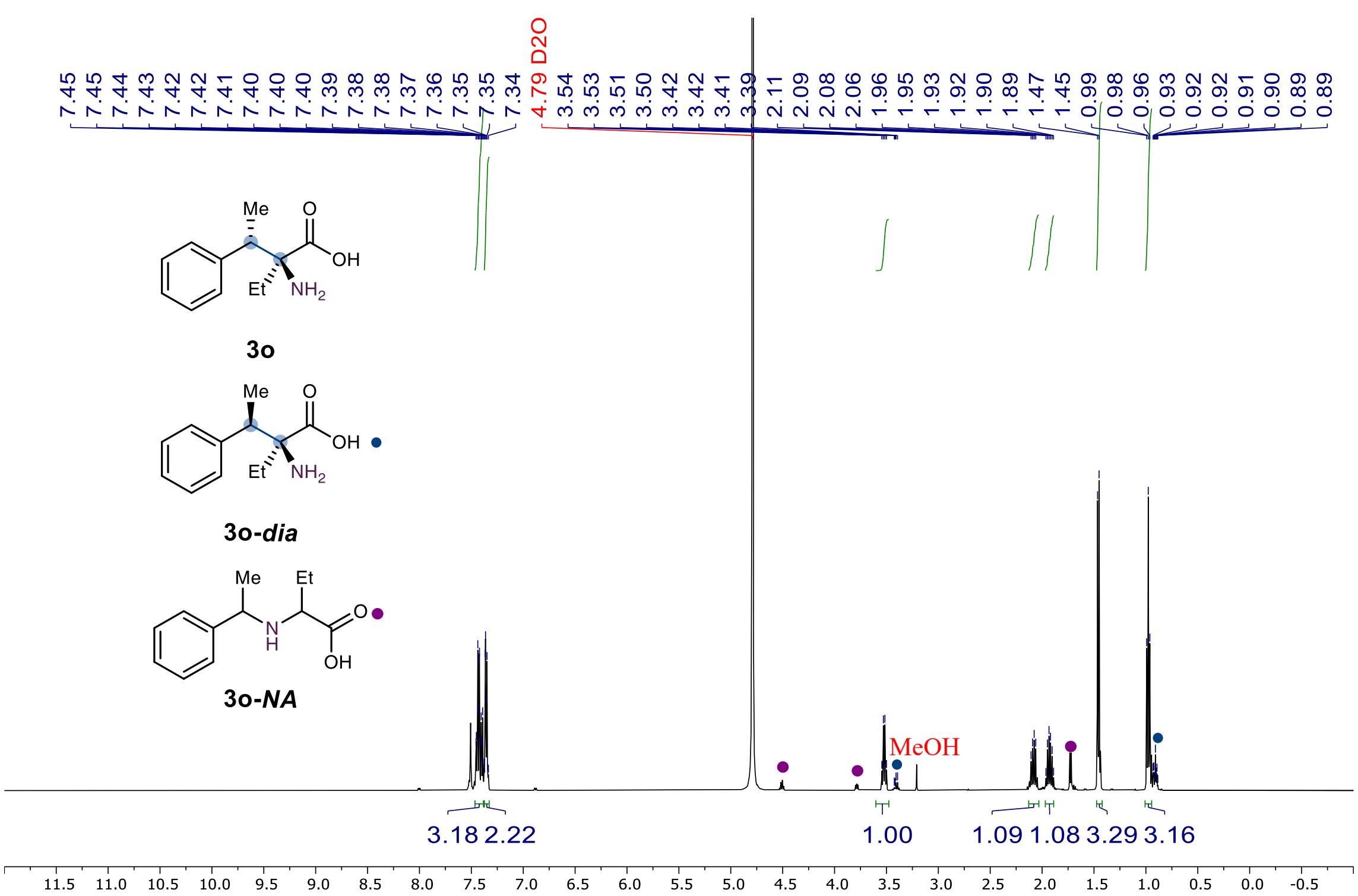

^{13}C NMR (101 MHz, D_2O) of (*2S*, *3S*)-2-amino-2-ethyl-3-phenylbutanoic acid (**3o**)

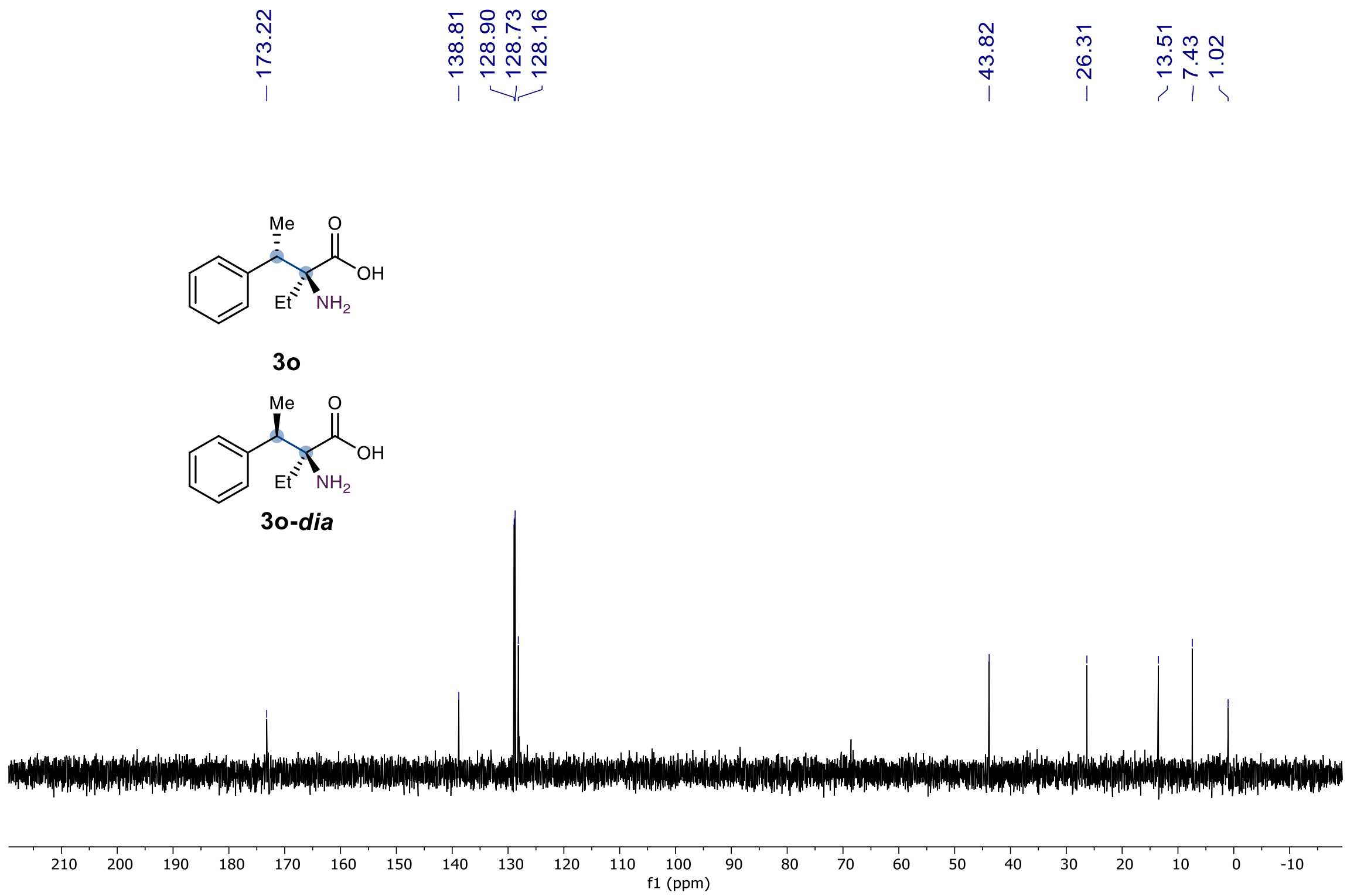

^{1}H NMR (400 MHz, D_2O) of (*E*)-2-amino-2-methyl-6-phenylhex-5-enoic acid (**7a**)

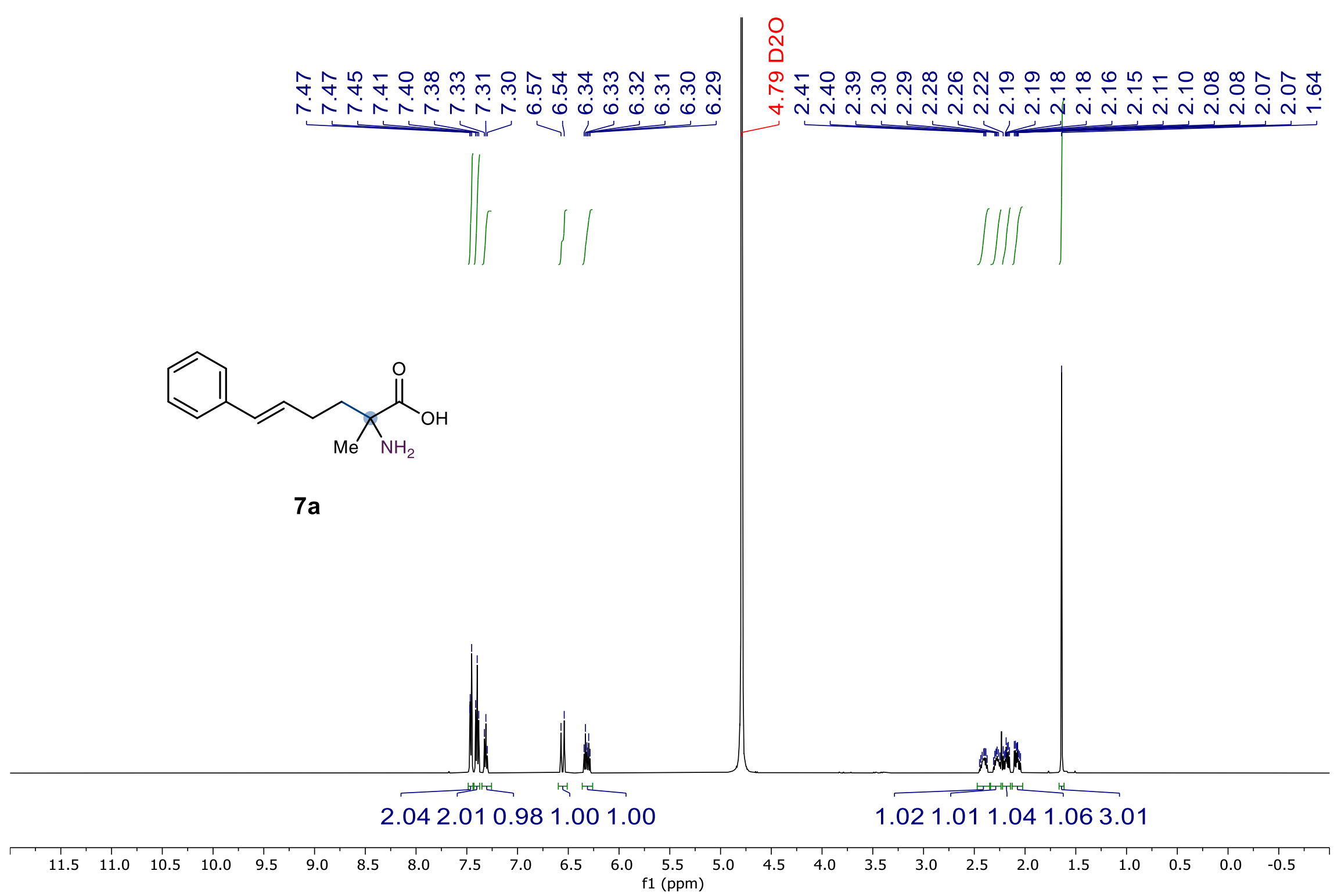

^{13}C NMR (101 MHz, D_2O) of (*E*)-2-amino-2-methyl-6-phenylhex-5-enoic acid (**7a**)

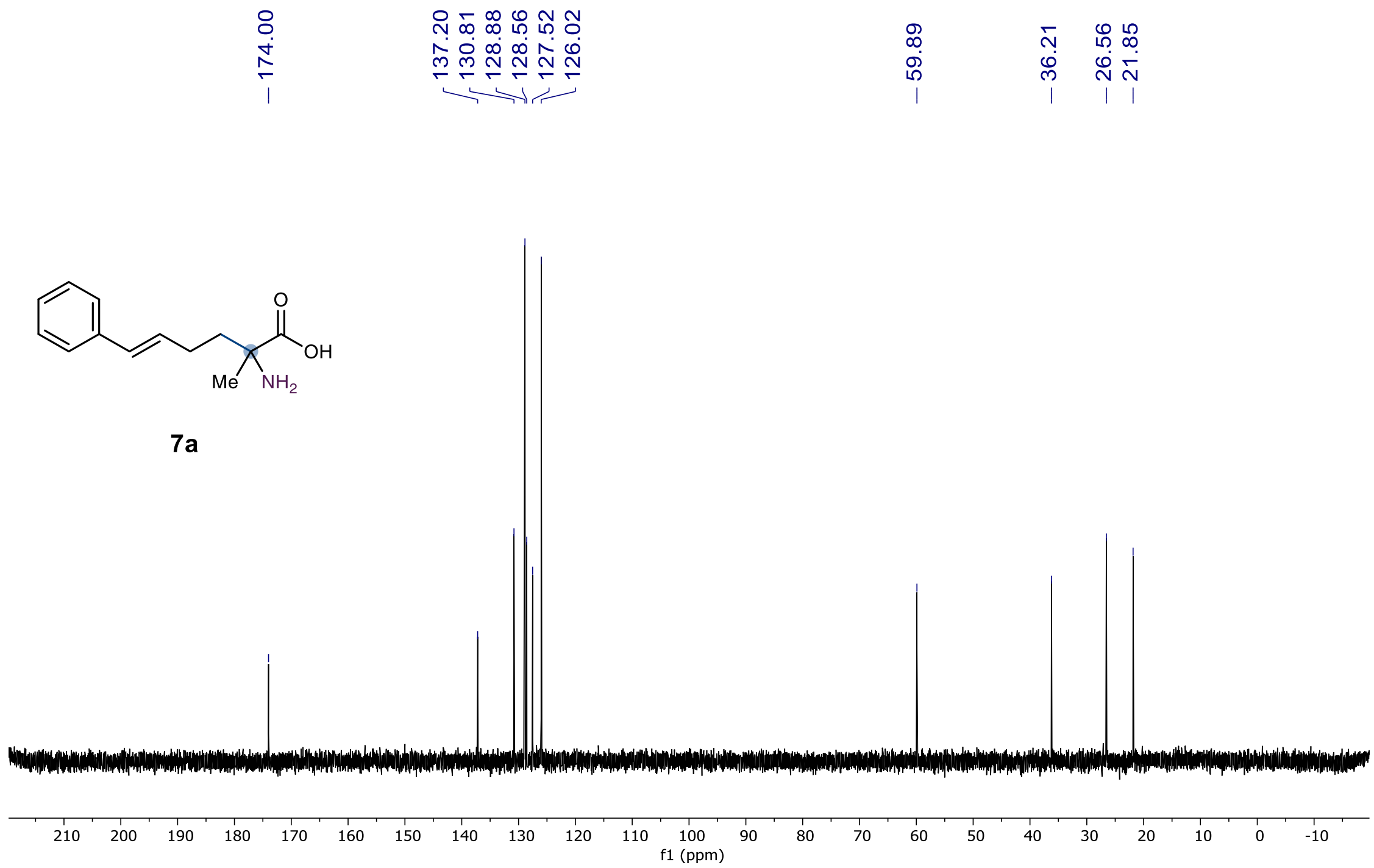

^{1}H NMR (400 MHz, $CDCl_3$) *anti*-Methyl-2-benzamido-2-methyl-3-phenylpent-4-enoate **(S9)**

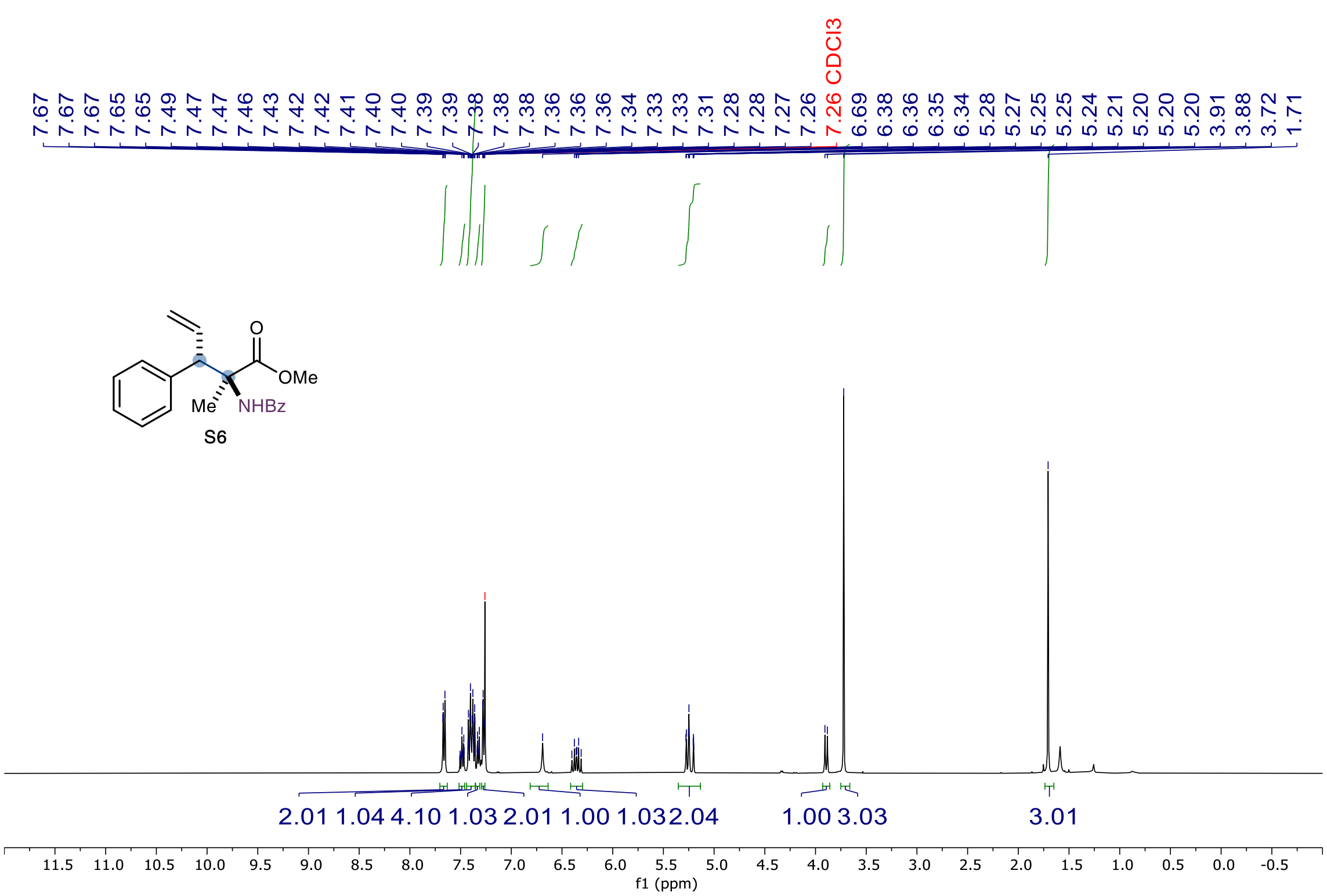

^{13}C NMR (101 MHz, $CDCl_3$) *anti*-Methyl-2-benzamido-2-methyl-3-phenylpent-4-enoate **(S9)**

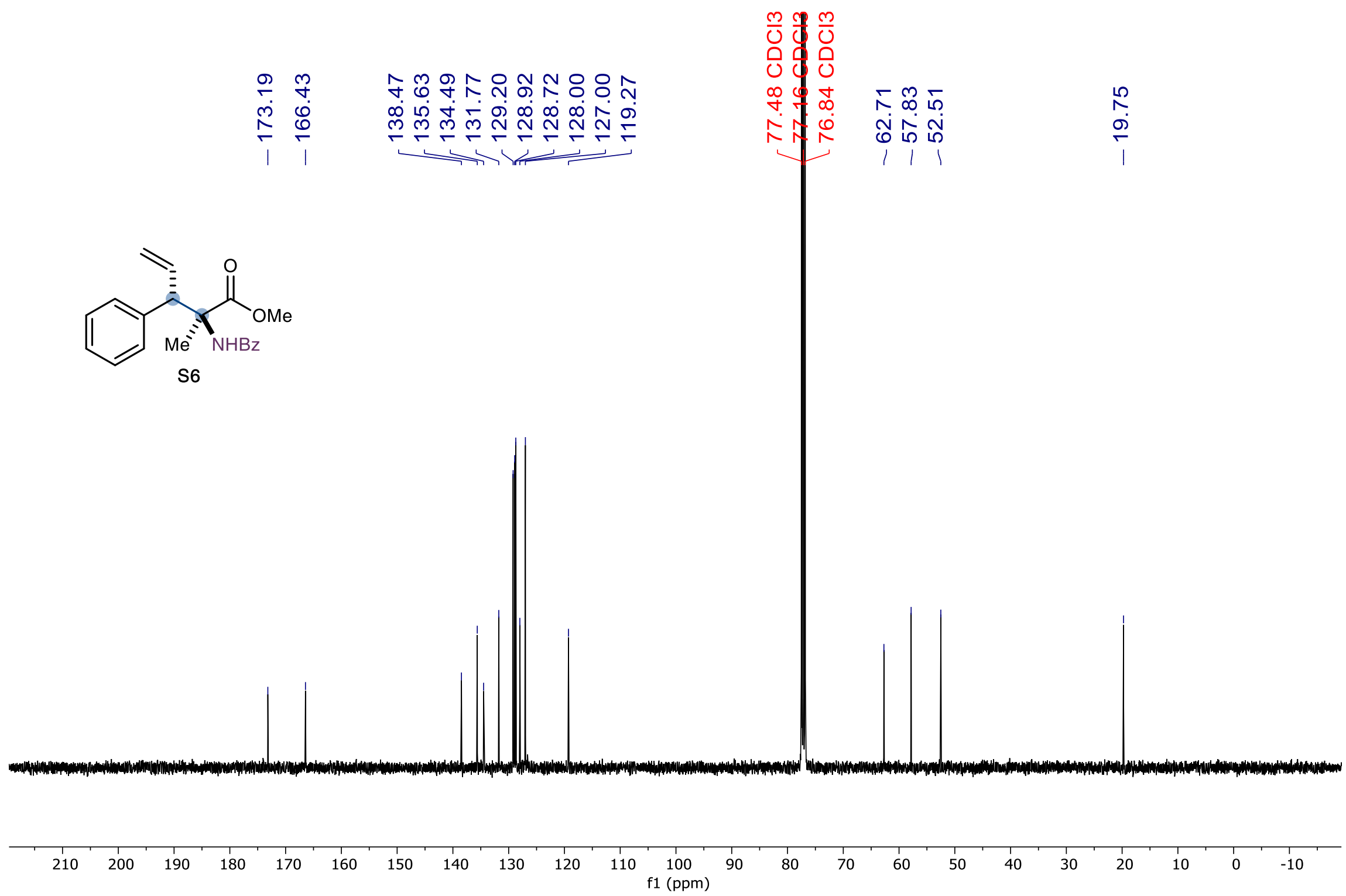

^{1}H NMR (400 MHz, $CDCl_3$) *syn*-Methyl-2-benzamido-2-methyl-3-phenylpent-4-enoate **(S9-*dia*)**

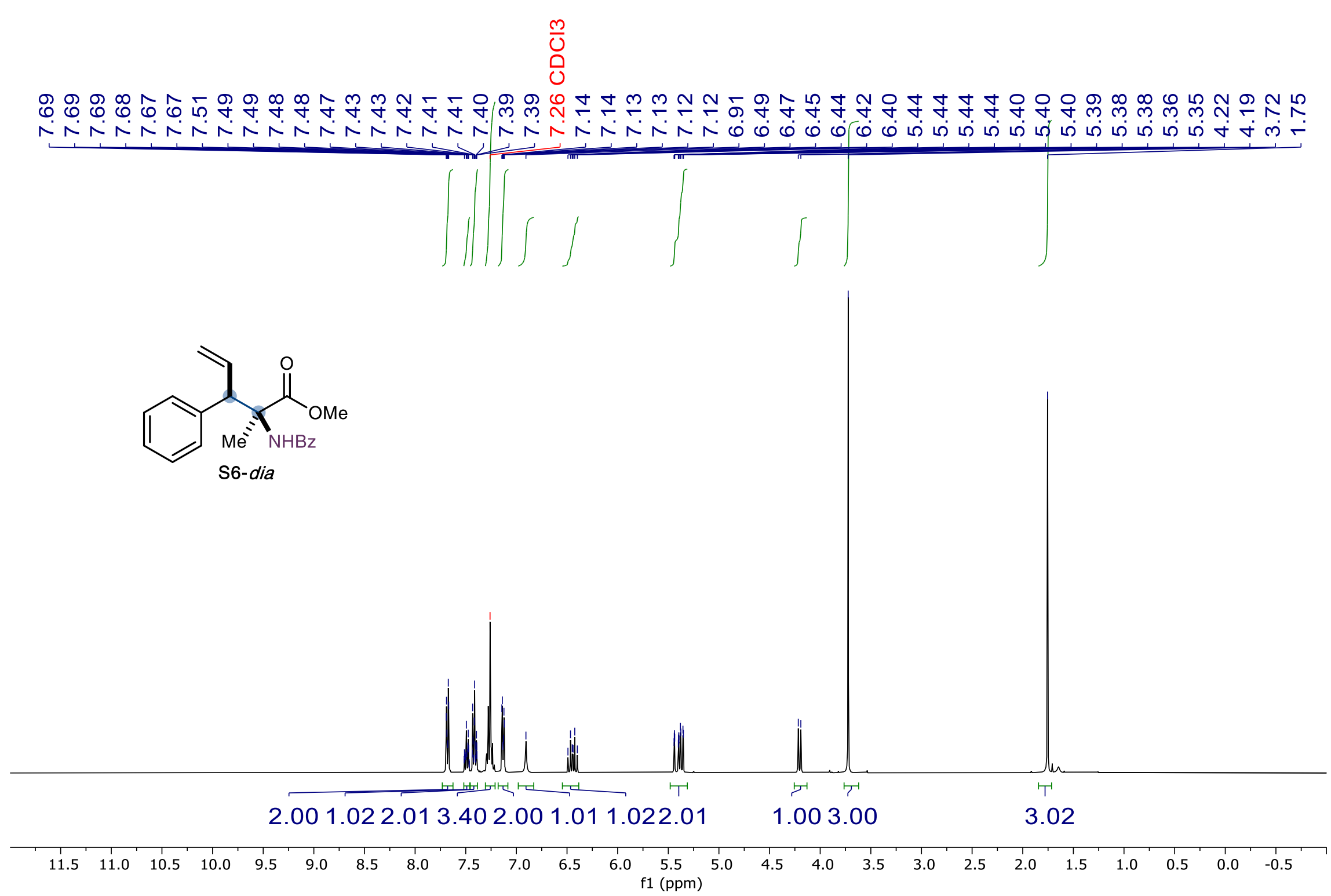

^{13}C NMR (101 MHz, $CDCl_3$) *syn*-Methyl-2-benzamido-2-methyl-3-phenylpent-4-enoate **(S9-*dia*)**

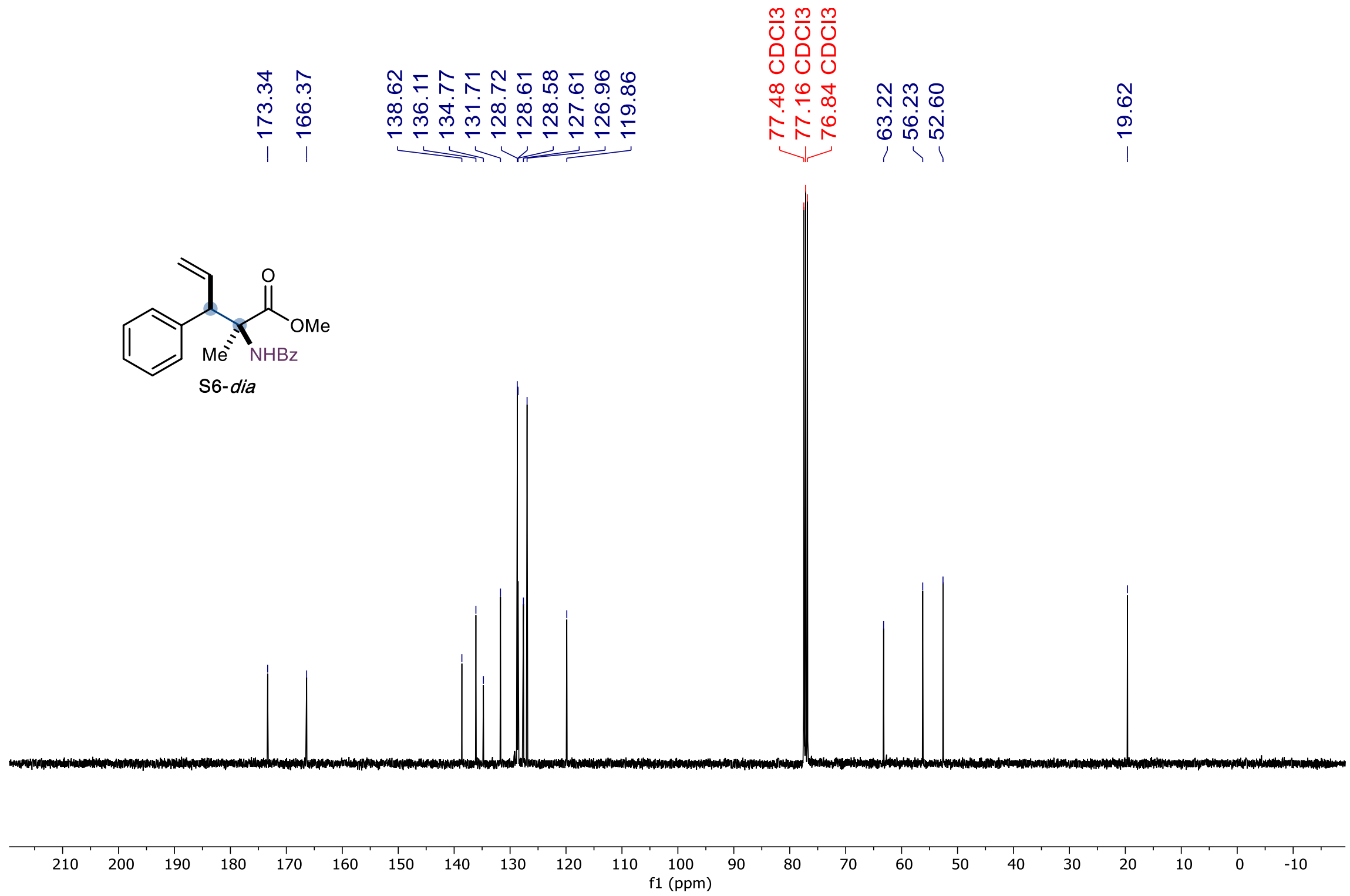

^{1}H NMR (400 MHz, D_2O) of *anti*-2-amino-2-(cyclopropyl(phenyl)methyl)butanoic acid (**7b**)

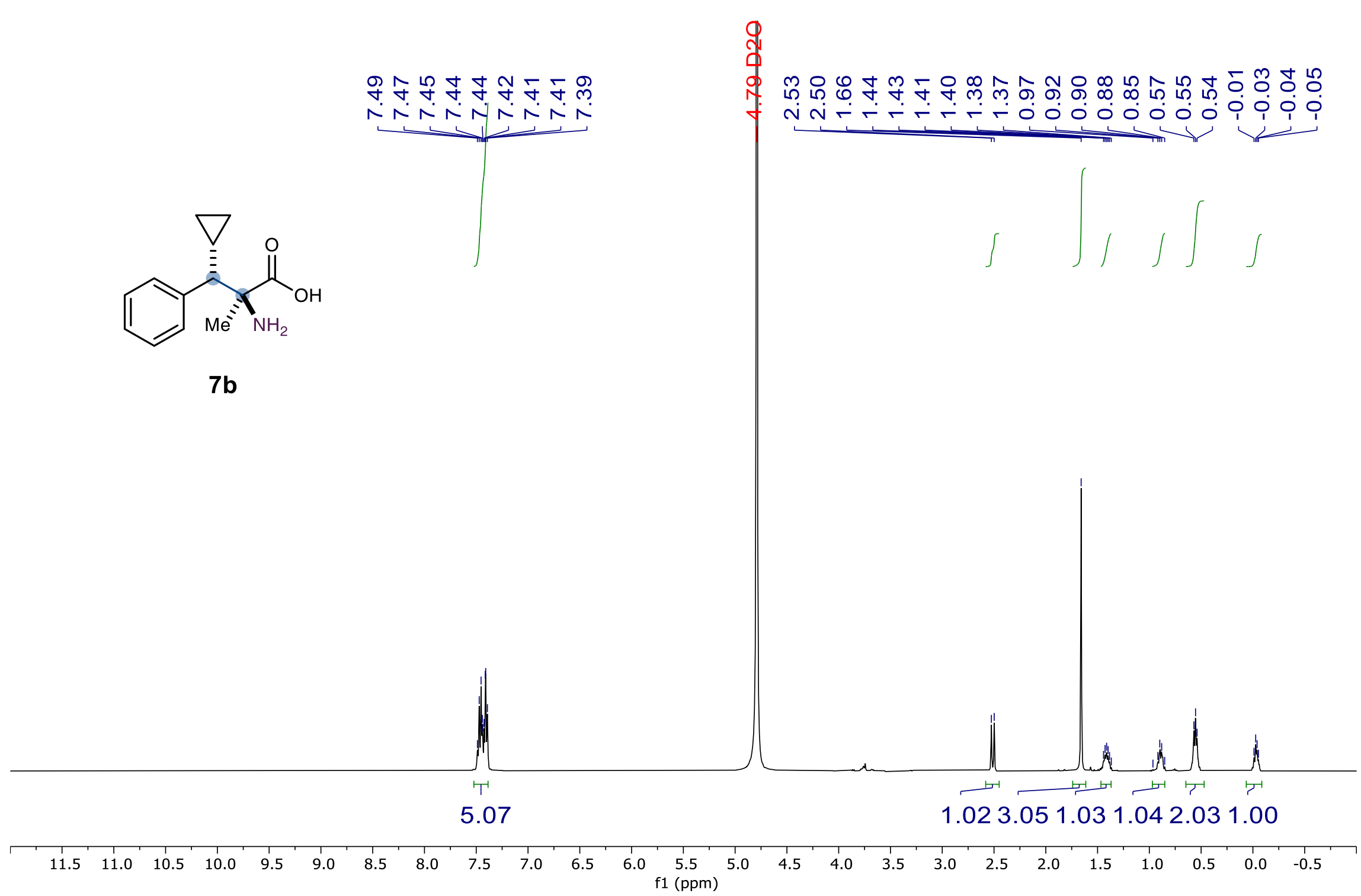

^{13}C NMR (101 MHz, D_2O) of *anti*-2-amino-2-(cyclopropyl(phenyl)methyl)butanoic acid (**7b**)

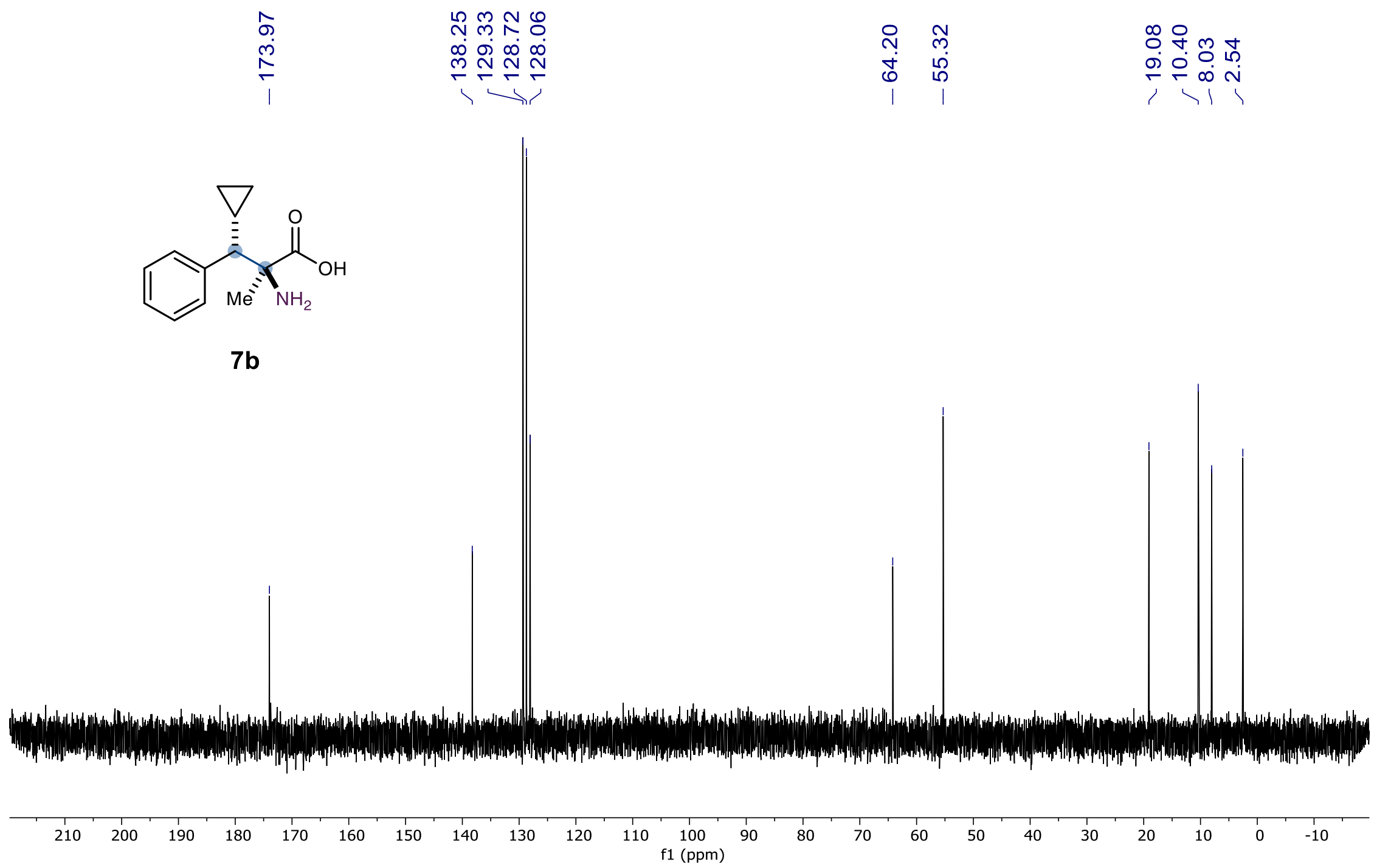

^{1}H NMR (400 MHz, D_2O) of *syn*-2-amino-2-(cyclopropyl(phenyl)methyl)butanoic acid (**7b-*dia***)

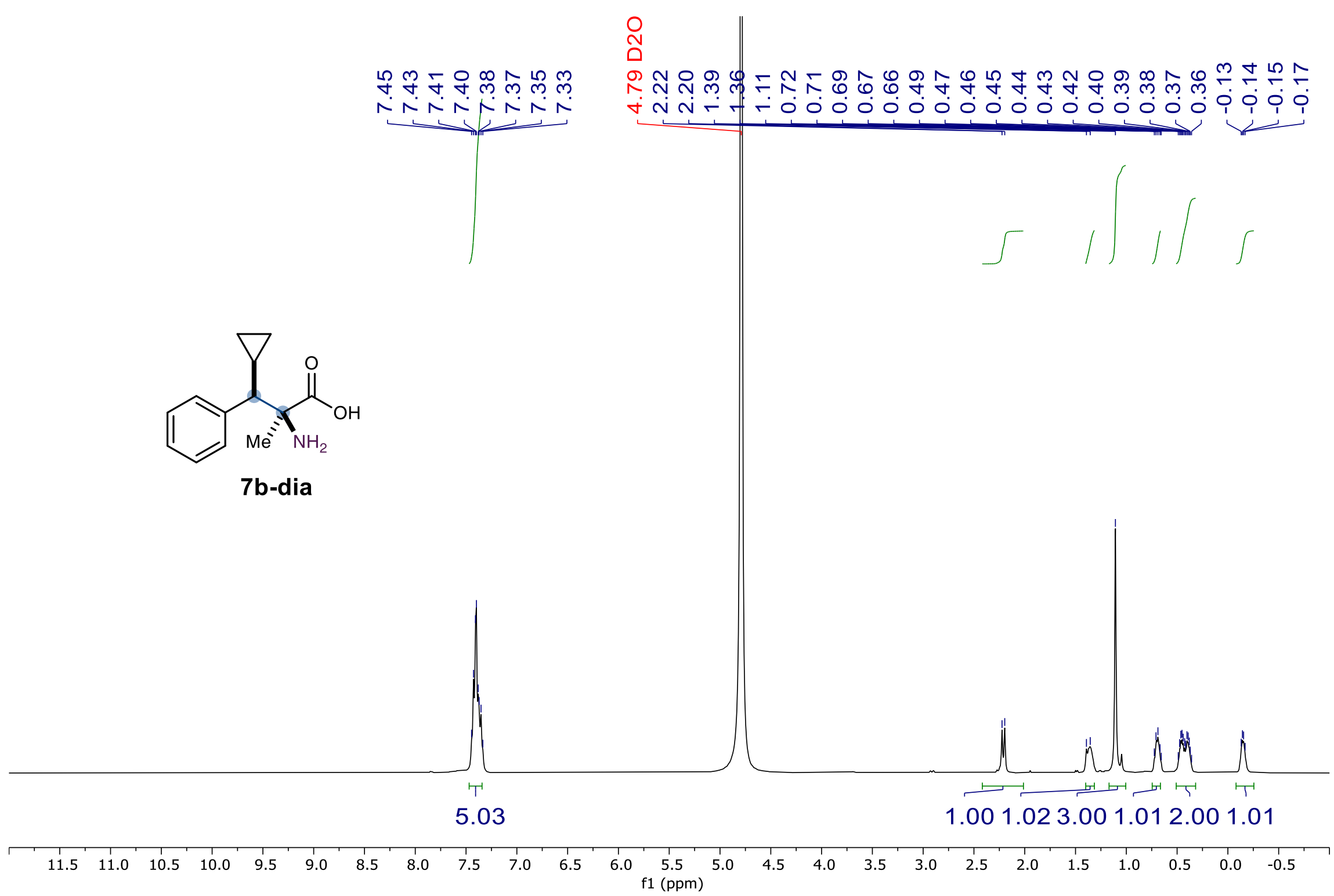

^{13}C NMR (101 MHz, D_2O) of *cis*-2-amino-2-(cyclopropyl(phenyl)methyl)butanoic acid (**7b-*dia***)

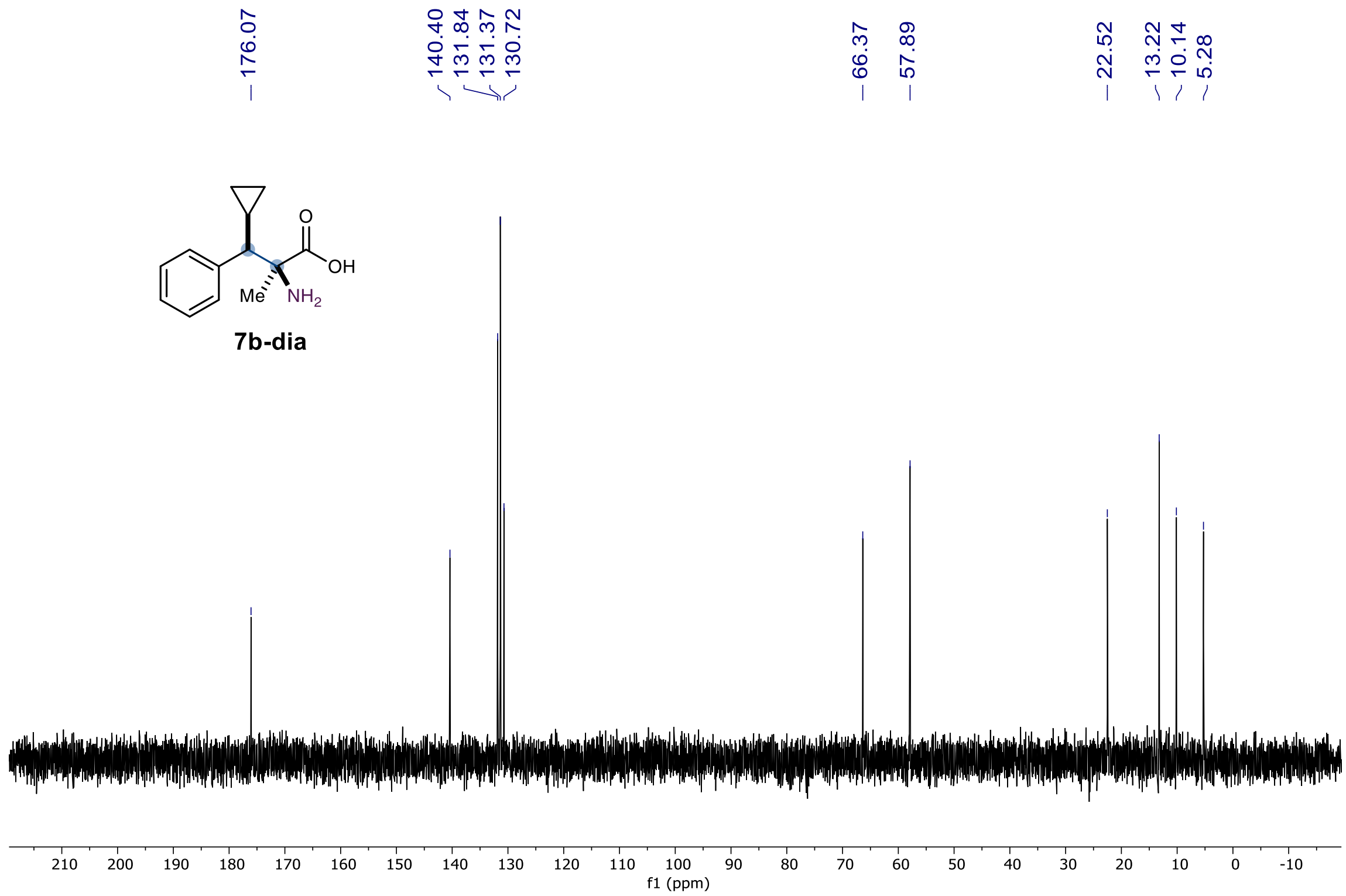

Supplement: Supplementary file 1 [file NIHMS2211252-supplement-SI.pdf]
